# Bayesian refinement of protein functional site matching

**DOI:** 10.1186/1471-2105-8-257

**Published:** 2007-07-17

**Authors:** Kanti V Mardia, Vysaul B Nyirongo, Peter J Green, Nicola D Gold, David R Westhead

**Affiliations:** 1 Department of Statistics, University of Leeds, Leeds, UK; 2 Department of Mathematics, University of Bristol, Bristol, UK; 3 Institute of Molecular and Cellular Biology, University of Leeds, Leeds, UK

## Abstract

**Background:**

Matching functional sites is a key problem for the understanding of protein function and evolution. The commonly used graph theoretic approach, and other related approaches, require adjustment of a matching distance threshold *a priori *according to the noise in atomic positions. This is difficult to pre-determine when matching sites related by varying evolutionary distances and crystallographic precision. Furthermore, sometimes the graph method is unable to identify alternative but important solutions in the neighbourhood of the distance based solution because of strict distance constraints. We consider the Bayesian approach to improve graph based solutions. In principle this approach applies to other methods with strict distance matching constraints. The Bayesian method can flexibly incorporate all types of prior information on specific binding sites (e.g. amino acid types) in contrast to combinatorial formulations.

**Results:**

We present a new meta-algorithm for matching protein functional sites (active sites and ligand binding sites) based on an initial graph matching followed by refinement using a Markov chain Monte Carlo (MCMC) procedure. This procedure is an innovative extension to our recent work. The method accounts for the 3-dimensional structure of the site as well as the physico-chemical properties of the constituent amino acids. The MCMC procedure can lead to a significant increase in the number of significant matches compared to the graph method as measured independently by rigorously derived p-values.

**Conclusion:**

MCMC refinement step is able to significantly improve graph based matches. We apply the method to matching NAD(P)(H) binding sites within single Rossmann fold families, between different families in the same superfamily, and in different folds. Within families sites are often well conserved, but there are examples where significant shape based matches do not retain similar amino acid chemistry, indicating that even within families the same ligand may be bound using substantially different physico-chemistry. We also show that the procedure finds significant matches between binding sites for the same co-factor in different families and different folds.

## Background

Recent advances in high-throughput structural determination techniques and structural genomics initiatives have produced an increase in volume of structural data for proteins prior to knowledge of their functions. With these advances has come the need to rapidly predict functions for proteins based on their structures.

Comparison of the overall folds of proteins by programs such as CE [1] or DALI [2] can be complemented by focusing on local structural comparisons centred on sites of functional importance such as ligand binding sites or catalytic sites [3-10]. These methods have largely arisen in response to the discovery of proteins sharing similar functions without similarity in their overall folds [11,12] or proteins adopting the same superfold [13] without functional similarity. Local structural comparison methods are based on the idea that geometrically similar sites are likely to have similar functions since their amino acids are conserved in precise orientations in order to perform their chemistry or their similar shapes and physico-chemical properties may be selective for similar small molecules such as substrates, inhibitors or co-factors. Hence, finding structural similarity to functional sites of known and characterised proteins may facilitate function prediction for newly determined protein structures even in the absence of overall fold or sequence similarity.

Functional site comparison methods essentially fall into one of two categories. The first category provides known templates of specific motifs of conserved amino acids or atoms often involved in enzyme catalysis [3,7,10]. These are knowledge-based methods which aim to discover new proteins with the same catalytic function. The second category consists of similarity searching algorithms [4,5,8,9,14] where prior knowledge of motifs is not required and site similarity is assessed by how closely the sites align and/or the proportion of overlap. Partial similarity between sites can be detected and hence much larger sites such as ligand binding sites can be compared. Methods addressing this problem generally represent functional sites or functional site surfaces as mathematical graphs for graph-theoretic or geometric hashing comparisons where graph vertex positions are placed using a variety of methods. CavBase [14], SiteEngine [8] and PINTS [9] for example use positions of pseudo-centres whereas eF-site [5] uses electrostatic potentials and surface curvature. SitesBase [15] and pvSoar [4] use *α*-shapes and an all-atom model respectively. Recently, a statistical approach using Bayesian modelling was proposed [16].

The Bayesian method gives a complete distribution of probable matches and hence an opportunity to explore several other solutions near the "optimal" solution. In addition, this approach automatically adapts to the level of noise in functional site atomic positions. The Bayesian method can also flexibly incorporate concomitant information like amino acid types and their different physico-chemistry classifications in matching biding sites. In principle, the Bayesian method could also incorporate all types of prior information on specific binding sites, which is nearly impossible with simple combinatorial approaches like graph matching.

In [16] each "site" consists of a single location, such as a *C*_*α *_atom in an amino acid. But for each *C*_*α *_atom there is also a neighbouring *C*_*β *_atom which can be paired to it. Thus the matching criterion need to be extended to require a close match not only for *C*_*α *_atoms in different configurations but also for the edges connecting *C*_*α *_atoms to their *C*_*β *_atoms.

In this paper, two substantial advances over [16] are matching of pairs of atoms (*C*_*α *_and *C*_*β*_) by a Bayesian method and refining the graph method in matching functional sites. We propose Bayesian modelling of graph matches using *C*_*α *_and *C*_*β *_atoms. This is done by adding a Markov chain Monte Carlo (MCMC) refinement step to the graph matching method [17].

Actually MCMC refinement procedure can be used to refine a match generated by any method. However for illustration purposes, here the graph theoretic method is chosen because it is a well tested method in a variety of geometrical matching problems for protein structure, see for example [3,7,14,18,19].

### The SITESDB database

Throughout this work we compared the local structural and physico-chemical environment of protein functional sites taken from a database of known sites (SITESDB) [17]. SITESDB entries were automatically formed from the PDB [20] by locating the local protein environment (amino acids within 5Å) around bound ligands (identified by PDB HETATM records) and author annotated active sites (identified by PDB SITE records). A protein may contain multiple functional sites so unique identifiers for SITESDB entries were generated from the four letter PDB identifier with an extra integer to distinguish sites from the same protein. For example, the identifiers 1hdx_0 and 1hdx_1 are separate sites from the protein with PDB identifier 1hdx.

The automatic extraction of sites results in multiple and incomplete representations of functional sites containing more than one bound ligand, or sites that are both annotated with SITE records and contain bound ligands. In these cases a better biochemical description of the site was obtained by merging component sites without duplication of their amino acid contents. Sites were merged if ligand atoms occurred within 5Å of atoms in a second ligand. In the absence of bound ligands, sites were merged if they were found to contain common amino acid residues.

### Representation and matching

We denote two functional sites to be matched by *X *and *Y *. Let the number of amino acids be *m *and *n *for *X *and *Y *respectively and we shall simply use *X*_*j*_, *j *= 1, 2, ..., *m *and *Y*_*k*_, *k *= 1, 2, ..., *n *to refer to the *jth *and *kth *amino acids in functional sites *X *and *Y*. Thus *X*_*j *_and *Y*_*k *_are simply the labels for amino acid with no coordinate system. Further, let *x*_1*j *_and *y*_1*k *_denote coordinates for *C*_*α *_atoms for the *jth *and *kth *amino acids in the two functional sites *X *and *Y*, respectively. We denote *C*_*β *_coordinates for the *jth *and *kth *amino acids in the two functional sites by *x*_2*j *_and *y*_2*k*_. Further, let *x*_1 _= {*x*_1*j *_: *j *= 1 ..., *m*}, *x*_2 _= {*x*_2*j *_: *j *= 1 ..., *m*} and *y*_1 _= {*y*_1*k *_: *k *= 1 ..., *n*}, *y*_2 _= {*y*_2*k *_: *k *= 1 ..., *n*}.

We consider matching at the level of amino acid residues. A match between two sites is a mapping between subsets of the residues in each site such that matched residues can be superimposed in three-dimensional space. Thus the problem is finding correspondence between amino acids of the two functional sites and the geometrical transformation to bring the corresponding atoms into registration. Knowing correspondence, solving for transformation is trivial and vice versa, but both are not known before hand.

We denote the matching between amino acids in *X *and *Y *using a matrix *M*:

Mjk={1if the jth amino acid corresponds to the kth amino acid,0otherwise.
 MathType@MTEF@5@5@+=feaafiart1ev1aaatCvAUfKttLearuWrP9MDH5MBPbIqV92AaeXatLxBI9gBaebbnrfifHhDYfgasaacH8akY=wiFfYdH8Gipec8Eeeu0xXdbba9frFj0=OqFfea0dXdd9vqai=hGuQ8kuc9pgc9s8qqaq=dirpe0xb9q8qiLsFr0=vr0=vr0dc8meaabaqaciaacaGaaeqabaqabeGadaaakeaacqWGnbqtdaWgaaWcbaGaemOAaOMaem4AaSgabeaakiabg2da9maaceqabaqbaeaabiGaaaqaaiabigdaXaqaaiabbMgaPjabbAgaMjabbccaGiabbsha0jabbIgaOjabbwgaLjabbccaGiabdQgaQjabdsha0jabdIgaOjabbccaGiabbggaHjabb2gaTjabbMgaPjabb6gaUjabb+gaVjabbccaGiabbggaHjabbogaJjabbMgaPjabbsgaKjabbccaGiabbogaJjabb+gaVjabbkhaYjabbkhaYjabbwgaLjabbohaZjabbchaWjabb+gaVjabb6gaUjabbsgaKjabbohaZjabbccaGiabbsha0jabb+gaVjabbccaGiabbsha0jabbIgaOjabbwgaLjabbccaGiabdUgaRjabdsha0jabdIgaOjabbccaGiabbggaHjabb2gaTjabbMgaPjabb6gaUjabb+gaVjabbccaGiabbggaHjabbogaJjabbMgaPjabbsgaKjabcYcaSaqaaiabicdaWaqaaiabb+gaVjabbsha0jabbIgaOjabbwgaLjabbkhaYjabbEha3jabbMgaPjabbohaZjabbwgaLjabc6caUaaaaiaawUhaaaaa@880D@

We denote the transformation to bring the configurations into alignment to be *x*_*ij *_= *Ay*_*ik *_+ *τ *for *M*_*jk *_= 1, *i *= 1, 2 where *A *is a rotation matrix and *τ *is a translation vector.

Our matching considers *C*_*α *_and *C*_*β *_atoms of each residue (except glycine where only the former is used). Note that since there are several examples of similarities in protein functional sites from evolutionarily unrelated proteins, which do not preserve the amino to carboxy terminal order of the matching residues, methods in this paper take no account of the sequential ordering of residues.

At the least restricted level, any residue was allowed to match any other, thus producing matches considering only the form or shape of the sites, in terms of the spatial arrangement of their constituent residues, irrespective of residues' identities and physico-chemical properties. A more restricted scheme was also considered where residues were only allowed to match within the same physico-chemical class: hydrophobic (A, F, I, L, M, P, V), polar (C, H, N, Q, S, T, W, Y), charged (D, E, K, R) or glycine (G).

It should be emphasised that this scheme was chosen to illustrate the value of the MCMC procedure, and that the procedure would be equally applicable to other possible atom matching schemes (e.g. involving more side chain atoms) or other physico-chemical groupings.

### Graph theoretic approach

Principles of graph theory have been applied to matching biomolecular configurations for some time [3,7,14,18,19,23-26]. These techniques are also used in computer vision research [23,27,28]. In this study each functional site is represented by a mathematical graph where vertices are placed at amino acid positions. Each vertex is connected by an edge to every other vertex in the same graph and each edge is labelled with the inter-residue distance [17]. Finding matching parts of the functional sites is equivalent to searching for maximum similarity between the graphs.

Combinatorial algorithms inherently require exhaustive search of the solution space. For example in graph matching an exhaustive search for solutions with various matching distance thresholds in a suitable range would be required to guarantee globally optimum solutions. In our applications Case 4 requires a different threshold to Cases 1–3 in order to give optimal matching. Case 4 requires a distance threshold of 1.0Å while Cases 1–3 require a distance threshold of 1.5Å. Stochastic approaches are the obvious candidate for consideration to avoid exhaustively searching the solution space.

### The Bayesian model

The Bayesian framework is particularly appealing in specialised problem domains since the "objective" (the posterior joint distribution) is a product of the "prior" and the "likelihood". The likelihood gives an indication of how well the observed data is consistent with parameters of the model and the prior can be used to incorporate expert knowledge of the problem.

The likelihood in [16] can be interpreted on considering that some amino acids in the two functional sites are related (match) under rigid body motion transformation subject to Gaussian, *N*(0, *σ*^2^) errors in the coordinates of the atoms. These errors could be due to crystallographic imprecision or protein phylogeny differences. The prior consists of the distribution on the transformation parameters. The joint posterior distribution (distribution of matching and transformation parameters given the data) is derived. The Bayesian method simultaneously estimates the matching and transformation parameters. Thus the whole posterior distribution is available to extract the statistical information on the parameters. Here we use a loss function to obtain point estimates of the matches being of the main interest. In the methods section we show the connection between the Bayesian method and minimising the RMSD when matches are known. Minimising RMSD is an appropriate criterion for optimising the geometrical transformation, given the matching. However, in no way does that logically imply that RMSD is an appropriate measure of quality of the matching itself – indeed one can trivially make the RMSD equal to 0 by matching just one pair of points, and in general minimised RMSD will increase with the number of points matched. RMSD can only be used as a measure for quality of match given the same number of matched points hence some other measures e.g. p-values [9] are required to assess the quality of a match.

We have also incorporated the expert knowledge on amino acid types through a prior on the matches.

### Assessing the quality of matches

A number of parameters are used to assess pair-wise matching solutions. We first consider the number of matched residues and the root mean square deviation (RMSD) between matched atoms. It is intuitively clear that matches of lower RMSD over larger numbers of matching residues are more statistically significant. A measure of this significance has recently been suggested [9], and was modified in this work to correct for the number of amino acids in functional sites being matched. The null hypothesis considers that we are matching random configurations. However for matching more than two atoms (e.g. *C*_*α *_and *C*_*β*_) in a single amino acid the null hypothesis is still matching random configurations but with some dependency (constraints) for atoms within the same amino acid.

The formula proposed is based on the geometry of a match with a given RMSD level, taking into account the abundance of different amino acid types and the different geometry that applies when various number of atoms are used to represent each amino acid. An extreme value distribution was fitted to the number of matches with an observed RMSD or better. Thus P-values formula is:

*P *= 1 - *e*^-*E*^

where *E *is the expected number of matches with an observed RMSD or better. We calculated E-values with a correction for the number of amino acids in the functional sites. The formula for expected number of matches with an RMSD value *R*_*M *_or better is found to be

E=C(m,n)abqPΦRM2.93q−5.88[yRM2]S[zRM3]T,q≥3
 MathType@MTEF@5@5@+=feaafiart1ev1aaatCvAUfKttLearuWrP9MDH5MBPbIqV92AaeXatLxBI9gBaebbnrfifHhDYfgasaacH8akY=wiFfYdH8Gipec8Eeeu0xXdbba9frFj0=OqFfea0dXdd9vqai=hGuQ8kuc9pgc9s8qqaq=dirpe0xb9q8qiLsFr0=vr0=vr0dc8meaabaqaciaacaGaaeqabaqabeGadaaakeaafaqabeqacaaabaGaemyrauKaeyypa0Jaem4qamKaeiikaGIaemyBa0MaeiilaWIaemOBa4MaeiykaKIaemyyaeMaemOyai2aaWbaaSqabeaacqWGXbqCaaGccqWGqbaucqqHMoGrcqWGsbGudaqhaaWcbaGaemyta0eabaGaeGOmaiJaeiOla4IaeGyoaKJaeG4mamJaemyCaeNaeyOeI0IaeGynauJaeiOla4IaeGioaGJaeGioaGdaaOWaamWaaeaacqWG5bqEcqWGsbGudaqhaaWcbaGaemyta0eabaGaeGOmaidaaaGccaGLBbGaayzxaaWaaWbaaSqabeaacqWGtbWuaaGcdaWadaqaaiabdQha6jabdkfasnaaDaaaleaacqWGnbqtaeaacqaIZaWmaaaakiaawUfacaGLDbaadaahaaWcbeqaaiabdsfaubaakiabcYcaSaqaaiabdghaXjabgwMiZkabiodaZaaaaaa@5E3C@

where *P*, the number of binding sites that were matched corrects for database size [9,31]. Φ is the product of percentage abundances of all matched amino acids, *q *is the number of matched amino acids, *S *is the number of amino acids with two atoms matched, *T *is the number of amino acids with more than two atoms matched. Our application uses dataset similar to previous studies [9,31] hence we use the same empirically derived constants: *a *= 3.704 × 10^6^, *b *= 1.790 × 10^-3^, *y *= 0.196 and *z *= 0.094. In our applications, *T *= 0. C(m,n)=3!(n3)(m3)
 MathType@MTEF@5@5@+=feaafiart1ev1aaatCvAUfKttLearuWrP9MDH5MBPbIqV92AaeXatLxBI9gBaebbnrfifHhDYfgasaacH8akY=wiFfYdH8Gipec8Eeeu0xXdbba9frFj0=OqFfea0dXdd9vqai=hGuQ8kuc9pgc9s8qqaq=dirpe0xb9q8qiLsFr0=vr0=vr0dc8meaabaqaciaacaGaaeqabaqabeGadaaakeaacqWGdbWqcqGGOaakcqWGTbqBcqGGSaalcqWGUbGBcqGGPaqkcqGH9aqpcqaIZaWmcqGGHaqidaqadaqaauaabeqaceaaaeaacqWGUbGBaeaacqaIZaWmaaaacaGLOaGaayzkaaWaaeWaaeaafaqabeGabaaabaGaemyBa0gabaGaeG4mamdaaaGaayjkaiaawMcaaaaa@3DB5@ is a correction factor for the number of amino acids, *m *and *n *in the functional sites. The correction factor is derived by considering that matching 3 points exhausts all degrees of freedom in optimal matching of rigid bodies [25]. Indeed, the first exponent of *R*_*M *_: 2.93*q *- 5.88 ≃ 3*q *- 6 which is expected from the Mardia-Dryden distribution of size-and-shape [32] so there is some theoretical support to the fitted formula, partly because for *q *matches in three-dimensions, the degrees of freedom are 3*q *- 6 since we loose 3 degrees of freedom for translation and 3 for rotation.

## Results

We considered two binding sites, the NAD binding site from an alcohol dehydrogenase structure (1hdx_1 in SITESDB with 60 amino acids), and a larger NADP binding site from a 17 – *β *hydroxysteroid dehydrogenase (1a27_0 in SITESDB with 63 amino acids) which includes both the co-factor and substrate binding regions. For these binding sites we performed the following matching studies

1) A functional site of alcohol dehydrogenase against NAD(P)(H) binding sites from proteins in the same SCOP family as alcohol dehydrogenase (Alcohol dehydrogenase-like, N-terminal domain; SCOP: c.2.1.1).

2) A functional site of 17 – *β *hydroxysteroid dehydrogenase against NAD(P)(H) binding sites from proteins in the same SCOP family as 17 – *β *hydroxysteroid dehydrogenase (Tyrosine-dependent oxidoreductases; SCOP: c.2.1.2).

3) The alcohol dehydrogenase functional site in (1) against NAD(P)(H) binding sites from proteins in the same SCOP superfamily as alcohol dehydrogenase but different families (SCOP: c.2.1.x; for *x *≠ 1).

4) The alcohol dehydrogenase functional site against

FAD/NAD(P)(H) binding sites from proteins in FAD/NAD(P)-binding domain (SCOP: c.3.1.x).

The first of these test cases is the most straightforward, involving matching the NAD binding site against similar sites in closely related proteins. The second is similar, but more challenging in matching, because the larger 1a27_0 site also incorporates the substrate (oestradiol) binding region. The associated family (c.2.1.2) is functionally broad and members catalyse reactions on a variety of diverse substrate molecules leading to variations in functional site shapes. Matching methods therefore need to identify matches in the related co-factor binding region and ignore local site dissimilarities owing to substrate variation. The third test case considers similarities in sites with more distant evolutionary relationships (where sequence similarity between the protein domains concerned is very low, but the structural similarity of the Rossmann fold remains). The fourth test case examines the ability of the method to locate site similarities between different folds that bind the same or related ligands.

### Case 1: Site 1hdx_1 matching against its own SCOP family

**Figure 1:**
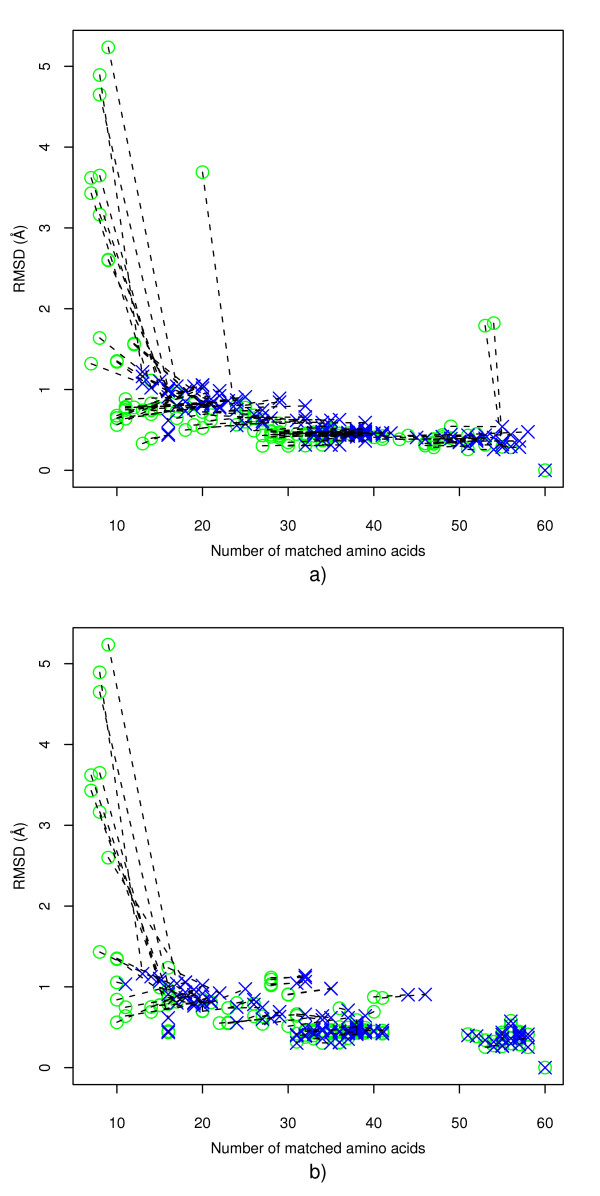
Alcohol dehydrogenase NAD-binding site (1hdx_1) matching against SCOP alcohol dehydrogenase-like family **(Case 1)**. a) Graph matching prior to MCMC refinement step showing results with/without amino acid property information. Each site in the family is represented by a circle (with) and cross (without) connected by a straight line to highlight the difference. b) MCMC refinement step of (a).

Figure 1a shows the results of using graph matching only where matching was performed with and without amino acid physico-chemical property information. First note that in the less restricted matching scenario, without amino acid group information, matches generally involve more residues and similar or lower RMSDs, as would be expected. Thus, in the figure, the lines connecting the restricted matches (circles) with the unrestricted matches (crosses) for each site family member often have a gradient that is negative or close to zero.

In the case of matching without property information, most sites in the family show a match with 1hdx_1 with a low RMSD (< 1.5Å) and a significant number of corresponding residues (> 8). However, this is not the case when amino acid property information is taken account of, and a minority of the matches show relatively high RMSDs (> 1.5Å), over generally lower numbers of matching residues. Thus it appears that lower quality matches can result from the use of amino acid property information, perhaps because these close relatives have conserved the shape of the binding site but not the physico-chemical characteristics. This may happen in binding site regions whose properties are not crucial to ligand binding; it is interesting here because with our use of very broadly defined physico-chemical groups it implies significant changes of physico-chemical properties.

**Figure 2:**
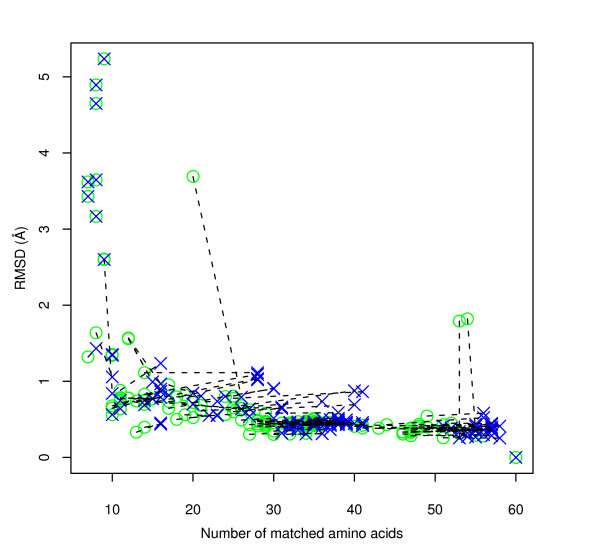
Effect of MCMC refinement on graph matches of 1hdx_1 (Alcohol dehydrogenase) against SCOP alcohol dehydrogenase-like family **(Case 1) **where corresponding amino acids are restricted to others in the same group. Each site in the family is represented by a circle (graph only) and cross (with MCMC refinement) connected by a straight line to highlight the difference.

Figures 1b and 2 show the effect of the MCMC refinement on the graph only matches of Figure 1a. The same basic conclusions can be drawn from Figure 1b as from Figure 1a. However, from Figures 1b and 2 it is clear that in a number of cases when matches with amino acid property information are considered, the MCMC refinement procedure produced significant improvements in the RMSDs (RMSD is improved from > 1.5Å to less than 1Å while also marginally increasing the number of matching residues). Thus the refinement procedure is able to improve some matches, even in this case of closely related sites.

The overall effect of the refinement procedure within this family can be considered in terms of the statistical significance of the matches obtained. This information is summarised in Table 1. When taking physico-chemical properties into account, 125/145 sites produced significant matches before refinement, and this increased to 131/145 after. We use the p-value 0.05 for the significance level. Without taking physico-chemical properties into account, 142/145 sites produced significant matches (p-value < 0.05) before MCMC refinement step; this increased to 143/145 sites after the refinement.

**Table 1:** Number of statistical significant functional sites before (Graph) and after the refinement step (MCMC), without amino acid property information

		Significant	
		
Case	Total	Graph	MCMC
alcohol dehydrogenase and family	145	142	143
17 – *β *hydroxysteroid dehydrogenase and family	326	248	318
alcohol dehydrogenase and superfamily	897	200	324
alcohol dehydrogenase	338	64	76
and FAD/NAD(P)-binding domain			

The improvement was a functional site from 2,4-dienoyl-CoA reductase (1guf_5) which before refinement matched with an RMSD of 1.076Å, a number of corresponding amino acids equal to 13, and a p-value of 0.08; after MCMC refinement step the RMSD was 1.127Å, the number of corresponding amino acids was 14 and p-value was 0.02.

**Figure 3:**
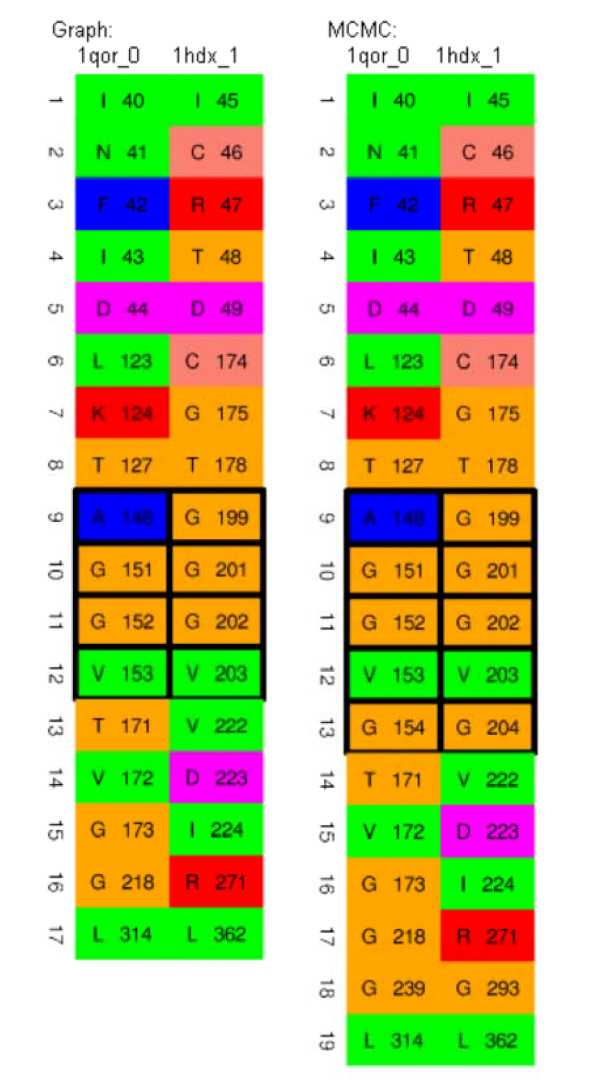
Corresponding amino acids between the NAD-binding site of alcohol dehydrogenase (1hdx_1) and NADP-binding site of quinone oxidoreductase (1qor_0) before and after MCMC refinement with the glycine rich motif highlighted (see main text) **(Case 1)**.

**Figure 4:**
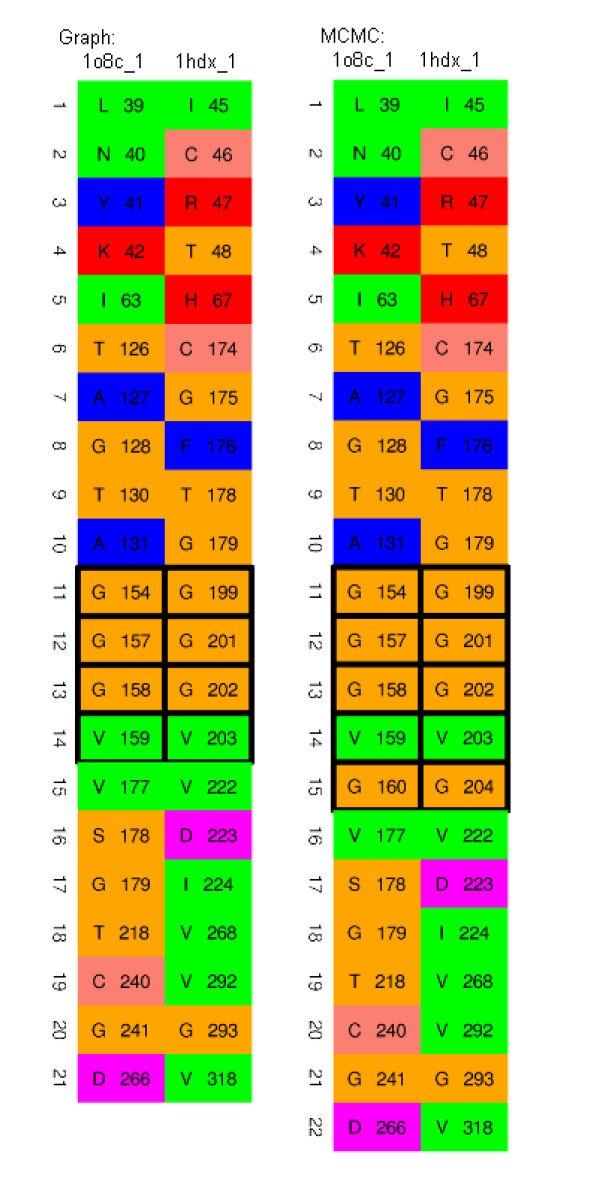
Corresponding amino acids between the NAD-binding site of alcohol dehydrogenase (1hdx_1) and NADP-binding site of hypothetical protein YhdH (1o8c_1) before and after MCMC refinement step with the glycine rich motif highlighted (see main text) **(Case 1)**.

Some examples where the MCMC refinement step produced improvements with obvious biochemical relevance are the sites in quinone oxidoreductase (PDB code 1qor) and hypothetical protein YhdH (PDB code 1o8c). The proteins in this family share a well known glycine rich motif (GXGXXG) in the binding site. For 1qor_0, before MCMC refinement step, 2 glycines in dinucleotide binding motif GLGGVG were matched by graph matching alone and this increased to 3 glycines after MCMC refinement. In the case of 1o8c the motif includes 4 glycines, of which 3 were matched before MCMC refinement step and all 4 after. The full matches with these motifs highlighted are shown in Figures 3 and 4.

### Case 2: 17 – β hydroxysteroid dehydrogenase and family

**Figure 5:**
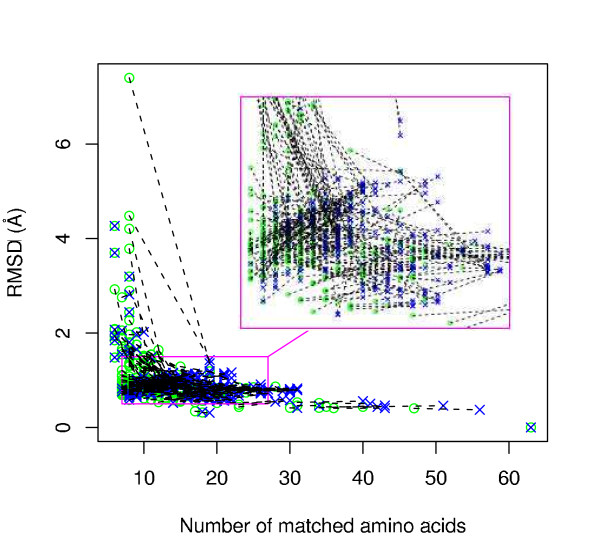
Effect of MCMC refinement on graph matches of 1a27_0 (17 – *β *hydroxysteroid dehydrogenase) against SCOP tyrosine dependent oxidoreductase family **(Case 2) **where corresponding amino acids are not restricted to others in the same group. Each site in the family is represented by a circle (graph only) and cross (with MCMC refinement) connected by a straight line to highlight the difference.

The effect of MCMC refinement on graph matches for the functional site of 17 – *β *hydroxysteroid dehydrogenase (1a27_0) matching against NAD(P)(H) binding sites within the same SCOP family (c.2.1.2) are shown in Figure 5 and Table 1. In the case of this more complex site the effect of MCMC refinement is greater, increasing the number of sites producing statistically significant matches from 248 to 318 of 326 sites. Figure 5 shows that most of these improvements are associated with an increased number of matching residues at a similar RMSD, but in a minority of sites RMSD also improved. Although this family catalyses reactions on a diverse range of substrates, the members have a clear evolutionary relationship, and statistically significant matches at least in the co-factor binding region should be expected. These results show that MCMC refinement of the initial match is often required for these to be detected. When comparing matching with and without amino-acid physico-chemical property information similar results to those of Case 1 were obtained (see additional file 2).

### Case 3: alcohol dehydrogenase and superfamily

**Figure 6:**
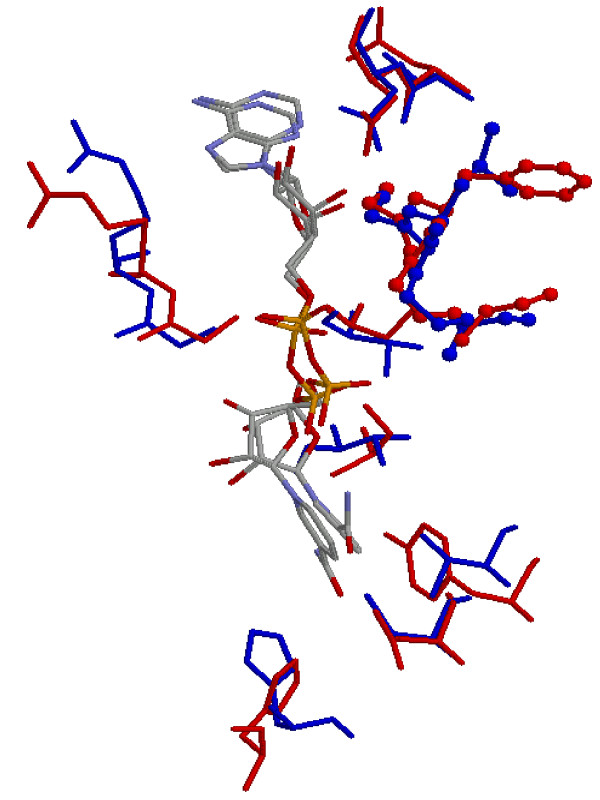
Superposition of matching amino acids **(Case 3) **between alcohol dehydrogenase (1hdx_1; blue) and glyceraldehyde-3-phosphate dehydrogenase (3dbv_3; red) after MCMC refinement (RMSD = 0.672; number of corresponding amino acids = 12; p-value = 3.68e-05). The matched dinucleotide binding motif is shown in ball-and-stick representation. Ligands are coloured in CPK colours.

In the case of the more distant evolutionary relationships probed by matching the site from alcohol dehydrogenase (1hdx_1) against members of the same SCOP superfamily but from different families, MCMC refinement also produced a large increase in the number of statistically significant matches. Of 897 sites, 200 produced significant matches using graph matching alone, and this was increased to 324 by MCMC refinement (see Table 1). As in Case 2 above, it is common with these more distantly related NAD(P) binding sites that significant matches involve the well-known glycine rich motif. An example of a match with the site 3dbv_3 in the glyceraldehyde-3-phosphate dehydrogenase structure is shown in Figure 6, in which this motif is highlighted.

### Case 4: alcohol dehydrogenase and FAD/NAD(P)-binding domain

The FAD/NAD(P)-binding domain is classified as a different fold in SCOP to the cases considered hitherto. Nevertheless, as its name suggests it binds ligands of interest in this work, NAD(P) and more commonly FAD. The latter dinucleotide ligand is related to NAD(P): approximately half of the molecule (an adenosine moiety and two phosphate groups) is identical to NADP while the other half differs substantially, with a flavin group replacing nicotinamide. It is known however that the ligand binding sites of this fold and the Rossmann fold are related, and both include glycine rich motifs associated with binding the shared phosphate groups. Thus some significant matches between these binding sites might be expected on biochemical grounds.

In this case, we consider matching without physico-chemical group information with the initial graph theory match using a matching tolerance of 1Å. As shown in Table 1, 64 of the 338 site matches showed statistical significance with graph matching only, and a further 12 attained this with MCMC refinement.

### Performance of MCMC

In addition to using RMSD as a diagnostic tool for monitoring convergence and using p-values as an objective function to compare the performance of graphical and MCMC methods, we also consider diagnostic plots and quantities for the Bayesian model stability and MCMC convergence.

We can see (in additional files 1, 2, 3, 4) that the number of matches, L=∑jkMjk
 MathType@MTEF@5@5@+=feaafiart1ev1aaatCvAUfKttLearuWrP9MDH5MBPbIqV92AaeXatLxBI9gBaebbnrfifHhDYfgasaacH8akY=wiFfYdH8Gipec8Eeeu0xXdbba9frFj0=OqFfea0dXdd9vqai=hGuQ8kuc9pgc9s8qqaq=dirpe0xb9q8qiLsFr0=vr0=vr0dc8meaabaqaciaacaGaaeqabaqabeGadaaakeaacqWGmbatcqGH9aqpdaaeqbqaaiabd2eannaaBaaaleaacqWGQbGAcqWGRbWAaeqaaaqaaiabdQgaQjabdUgaRbqab0GaeyyeIuoaaaa@37B2@ at each iteration for most cases converged i.e. variance for *L *is very small. There are very few exceptions where convergence was not reached e.g. 1a71_1, 3hud_1 and 1pl8_7 in Case 1 when not using amino acid properties. Here variances for *L *are very big which suggest non-convergence of the MCMC.

**Figure 7:**
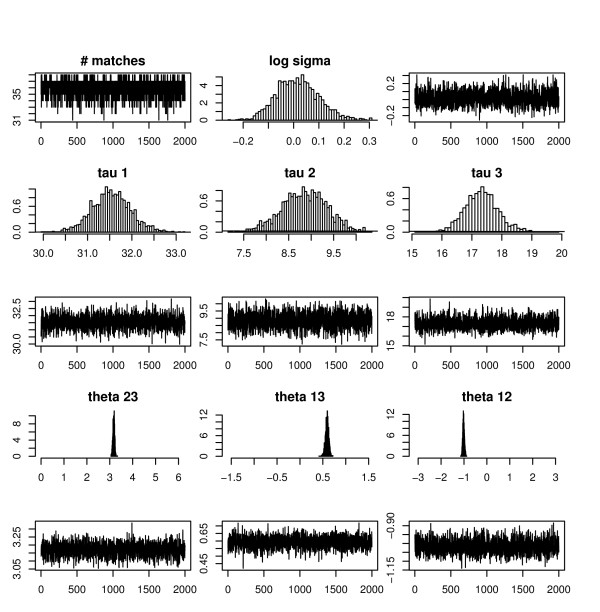
Histograms and traces of parameters when matching 17 – *β *hydroxysteroid dehydrogenase and carbonyl reductase (1cyd_1).

Figure 7 shows histograms and traces for parameters when matching 1a27_0 against 1cyd_1 (**Case 1**). Traces for number of matches (L), log *σ*, *τ *and rotation angles show good mixing, stationarity and that convergence was reached. Histograms for rotation angles are well peaked showing stability of the solution. For a detailed discussion on sensitivity analysis on hyper-parameters for the model, see the methods section under sensitivity of hyper-parameters.

## Discussion

Since the connection between statistical and biological significance is not straightforward example applications above were carefully chosen to be well understood cases where matches can be interpreted relatively easily in biochemical terms. For structural and evolutionary relationships SCOP [33] was used. It has been seen that MCMC refinement step can provide significant improvement over graph-matching techniques.

In this paper the method uses matching schemes that are relatively unrestricted in terms of amino acid identity (either with no restriction or matching in broadly defined physico-chemical groups). As currently formulated it is therefore better suited to the study of larger ligand binding sites, than smaller sites associated, for example, with enzymatic catalysis. The former are more likely to be defined by shape and physico-chemical properties, while the latter depend critically on precise amino acid residue identities. For our example applications we have therefore chosen sites for the binding of some very common biochemical ligands related to NAD(P) (nicotinamide adenine dinucleotide (phosphate)) and FAD (flavin adenine dinucleotide). These ligands are bound as co-factors by a large variety of enzyme domains, many of which come from the Rossmann family of protein folds. Importantly, there are many proteins of known structure that bind these related co-factors ranging from close evolutionary relatives, through very distant relatives to proteins of different fold and likely independent evolutionary origin.

The examples given above make a clear case that MCMC refinement can improve ligand binding site matches generated by graph matching, in terms of both the statistical and biological significance of the match. We attribute this success to the lack of dependence on strict distance matching criteria, which are rigidly enforced in graph matching. Statistical modelling in refinement of matches appears to have been successful in automatically adapting to shape variations in ligand binding sites, which might be due to different noise levels in atomic positions or protein phylogeny differences, among other factors. Refined matches usually retain a similar RMSD, and achieve greater significance through expansion of the number of matching residues from the core graph match. We have noted however that in some cases significant reductions in the match RMSD are also achieved by refinement.

Dependence on a strict matching tolerance is not limited to graph matching, but is also a feature of other matching methods commonly used in the field (e.g. geometric hashing). It is important to note that the MCMC refinement procedure can be applied to a starting match generated by any method; and that the graph procedure chosen here was simply intended as an example. Equally MCMC procedure can be applied to matching with no previously generated starting match, for example by starting from randomly generated matches. That is, the MCMC method provides a stand-alone algorithm for matching. However, we find that obtaining good matches by this method is very expensive in terms of computational time. While methods such as graph matching can be applied to database searching, where a site is matched against all members of a large database of sites, this would be impractical for matching by MCMC alone with modest computing facilities. There is also a need for closely monitoring convergence diagnostics. We suggest therefore that the MCMC procedure would be most advantageous when applied to the best hits from a database search using a faster method, and that in many cases it would increase the number of significant hits. Thus this method is aimed at refining matches and complementing other methods e.g. graph matching as used here. Furthermore, the method provides the full joint posterior distribution so that we have for example, the posterior distribution for the matching matrix (giving probabilities on matches) as well as the parameters of the transformation simultaneously. There is flexibility in Bayesian approach due to the ability to specify distributions on matching coordinates and relative probabilities on the orientation of matching residues as opposed to using matching distance threshold as in combinatorial methods.

From our experiments, matching both *C*_*α *_and *C*_*β *_atoms in our extended model gives solutions with smaller RMSD values (for *C*_*α *_and *C*_*β *_atoms) than when matching using *C*_*α *_atoms only.

Our approach using MCMC with detailed balance update and drawing from the posterior of all parameters in principle help escape local maxima for the model better than alternative methods using for example an annealing schedule.

Furthermore, quantifying the quality of the solution by RMSD in addition to the number of matches ensures that reported improvements over graph matching (e.g. an increased number of matches in examples illustrated in Figures 2, 3, 4, 5) really occur in one single mode for the match matrix *M*. Thus a smaller RMSD guarantees that we have a proper summary for *M *as opposed to having a point estimate of the match matrix invalidly summarising two or several solutions that the Markov chain visited separately, each with a particular probability.

## Conclusion

We have made only a very basic study of the effect of including amino-acid residue physico-chemical property information in matching, contrasting matches obtained without restriction (any residue may match any other) with slightly more restrictive matching (residues only allowed to match within relatively broadly defined groups). It is interesting that even with very broadly defined groups, fewer statistically significant matches are generally obtained than when matching is without restriction. This could suggest that the physico-chemical properties of sites binding the same or similar ligands can change significantly in evolution. It is however most likely to reflect increased flexibility to change in peripheral residues that are less important for binding, and needs further investigation. The main point of this work is that MCMC refinement can improve matches under either matching regime. Indeed in a few cases of matching with/out physico-chemical groups, we showed that some graph matches without statistical significance were converted to significant matches by the MCMC procedure, revealing that using graph matching alone could lead to some erroneous conclusions in this respect.

## Methods

We consider matching two functional sites *X *and *Y *using the graph theoretic method and the Bayesian modelling. First, we give the graph theoretical approach in the next section. We consider the Bayesian method in the following section.

### Graph theoretic approach

Graphs, say *G*_1 _and *G*_2 _are constructed to represent sites *X *and *Y *respectively. Vertices are placed at amino acid positions and edges between vertices represent inter-atomic distances within the functional site. Similar subgraphs for *G*_1 _and *G*_2 _correspond to the matching parts of *X *and *Y*. A search for similar maximal subgraphs for *G*_1 _and *G*_2 _corresponds to finding a clique (a maximal complete subgraph) i.e. the biggest completely connected subgraph in the vertex product graph of *G*_1 _and *G*_2_.

**Definition: **vertex product graph.

Denote sets of vertices for *G*_1 _and *G*_2 _by *V*_1 _and *V*_2_; *V*_1 _= {*X*_*j*_, *j *= 1, 2, ..., *m*} and *V*_2 _= {*Y*_*k*_, *k *= 1, 2, ..., *n*}. A vertex product graph, say *H*_*v *_= *G*_1 _○_*v *_*G*_2 _has a vertex set *V*_*H *_= *V*_1 _× *V*_2 _consisting of vertices defined for each pair (*X*_*j*_, *Y*_*k*_) with *X*_*j *_∈ *V*_1 _and *Y*_*k *_∈ *V*_2 _having the same attribute. An edge between two vertices *v*_*h *_= (*X*_*j*_, *Y*_*k*_), *v*_*h' *_= (*X*_*j'*_, *Y*_*k'*_) ∈ *V*_*H *_exists for *j *≠ *j' *and *k *≠ *k' *when

1. the absolute difference between distances |*x*_1*j *_- *x*_1*j'*_| and |*y*_1*k *_- *y*_1*k'*_| and

2. also the absolute difference between distances |*x*_2*j *_- *x*_2*j'*_| and | *y*_2*k *_- *y*_2*k'*_|

are both less than 1.5Å (matching distance threshold).

In the least restrictive case all amino acids are assumed to have the same attribute and hence matching can occur between any amino acid and is only dependent on inter-residue distances. Alternatively vertices can be labelled with residue physico-chemical properties to restrict matching to amino acids in the same group.

In this work we used the clique detection algorithm of Carraghan and Pardalos [29] to find maximal common subgraphs. Graph matches based on inter-residue distances are not necessarily superimposable (e.g. mirror image sites). Subsequently, a Procrustes algorithm [30] is used to check that matched configurations are geometrically superimposable.

### Bayesian alignment

Recall that the matching between amino acids in *X *and *Y *is represented by a matrix *M*:

Mjk={1if the jth amino acid corresponds to the kth amino acid,0otherwise,
 MathType@MTEF@5@5@+=feaafiart1ev1aaatCvAUfKttLearuWrP9MDH5MBPbIqV92AaeXatLxBI9gBaebbnrfifHhDYfgasaacH8akY=wiFfYdH8Gipec8Eeeu0xXdbba9frFj0=OqFfea0dXdd9vqai=hGuQ8kuc9pgc9s8qqaq=dirpe0xb9q8qiLsFr0=vr0=vr0dc8meaabaqaciaacaGaaeqabaqabeGadaaakeaacqWGnbqtdaWgaaWcbaGaemOAaOMaem4AaSgabeaakiabg2da9maaceqabaqbaeaabiGaaaqaaiabigdaXaqaaiabbMgaPjabbAgaMjabbccaGiabbsha0jabbIgaOjabbwgaLjabbccaGiabdQgaQjabdsha0jabdIgaOjabbccaGiabbggaHjabb2gaTjabbMgaPjabb6gaUjabb+gaVjabbccaGiabbggaHjabbogaJjabbMgaPjabbsgaKjabbccaGiabbogaJjabb+gaVjabbkhaYjabbkhaYjabbwgaLjabbohaZjabbchaWjabb+gaVjabb6gaUjabbsgaKjabbohaZjabbccaGiabbsha0jabb+gaVjabbccaGiabbsha0jabbIgaOjabbwgaLjabbccaGiabdUgaRjabdsha0jabdIgaOjabbccaGiabbggaHjabb2gaTjabbMgaPjabb6gaUjabb+gaVjabbccaGiabbggaHjabbogaJjabbMgaPjabbsgaKjabcYcaSaqaaiabicdaWaqaaiabb+gaVjabbsha0jabbIgaOjabbwgaLjabbkhaYjabbEha3jabbMgaPjabbohaZjabbwgaLjabbYcaSaaaaiaawUhaaaaa@8808@

and the transformation to bring the configurations into alignment is *x*_*ij *_= *Ay*_*ik *_+ *τ *for *M*_*jk *_= 1, *i *= 1, 2 where *A *is a rotation matrix and *τ *is a translation vector.

Given the map *M*, it is straight-forward using least squares estimation to calculate transformation parameters in order to bring the two configurations into registration or optimal alignment [30,32]. However *M*, *A *and *τ *are all unknown.

The model for *C*_*α *_atoms only was developed in [16] and the joint posterior distribution is

p(M,A,τ,σ,x1,y1)∝prior×likelihood=|A|np(A)p(τ)p(σ)×∏j,k:Mjk=1(κφ({x1j−Ay1k−τ}/σ√2)(σ√2)d)
 MathType@MTEF@5@5@+=feaafiart1ev1aaatCvAUfKttLearuWrP9MDH5MBPbIqV92AaeXatLxBI9gBaebbnrfifHhDYfgasaacH8akY=wiFfYdH8Gipec8Eeeu0xXdbba9frFj0=OqFfea0dXdd9vqai=hGuQ8kuc9pgc9s8qqaq=dirpe0xb9q8qiLsFr0=vr0=vr0dc8meaabaqaciaacaGaaeqabaqabeGadaaakeaafaqaaeGacaaabaGaemiCaaNaeiikaGIaemyta0KaeiilaWIaemyqaeKaeiilaWccciGae8hXdqNaeiilaWIae83WdmNaeiilaWIaemiEaG3aaSbaaSqaaiabigdaXaqabaGccqGGSaalcqWG5bqEdaWgaaWcbaGaeGymaedabeaakiabcMcaPaqaaiabg2Hi1kabbchaWjabbkhaYjabbMgaPjabb+gaVjabbkhaYjabgEna0kabbYgaSjabbMgaPjabbUgaRjabbwgaLjabbYgaSjabbMgaPjabbIgaOjabb+gaVjabb+gaVjabbsgaKbqaaaqaaiabg2da9maaemaabaGaemyqaeeacaGLhWUaayjcSdWaaWbaaSqabeaacqWGUbGBaaGccqWGWbaCcqGGOaakcqWGbbqqcqGGPaqkcqWGWbaCcqGGOaakcqWFepaDcqGGPaqkcqWGWbaCcqGGOaakcqWFdpWCcqGGPaqkcqGHxdaTdaqeqbqaamaabmaabaWaaSaaaeaacqWF6oWAcqWFgpGzcqGGOaakcqGG7bWEcqWG4baEdaWgaaWcbaGaeGymaeJaemOAaOgabeaakiabgkHiTiabdgeabjabdMha5naaBaaaleaacqaIXaqmcqWGRbWAaeqaaOGaeyOeI0Iae8hXdqNaeiyFa0Naei4la8Iae83WdmhccaGae4NgIyncbaGae0NmaiJae0xkaKcabaGaeiikaGIae83WdmNae4NgIyTae0NmaiJae0xkaKYaaWbaaSqabeaacqWGKbazaaaaaaGccaGLOaGaayzkaaaaleaacqWGQbGAcqGGSaalcqWGRbWAcqGG6aGocqWGnbqtdaWgaaadbaGaemOAaOMaem4AaSgabeaaliabg2da9iabigdaXaqab0Gaey4dIunaaaaaaa@9DB1@

where *p*(*A*), *p*(*τ*) and *p*(*σ*) denote prior distributions for *A*, *τ *and *σ*. |*A*| is the Jacobian of transformation from the space of *X *into the space of *Y *; *κ *measures the tendency *a priori *for points to be matched and can be a function of concomitant information like amino acid types.

We assume presence of Gaussian noise *N*(0, *σ*^2^) in the atomic positions for *x*_1*j *_and *y*_1*k *_thus *φ*(·) is the standard normal probability density function. Here the dimension, *d *= 3.

### Connection with combinatorial algorithms

We note that there is strong relationship between (combinatorial) algorithms minimising least squares (RMSD) and the Bayesian approach with Gaussian error model. We now give the connection between the joint posterior in Equation 3 and the RMSD. Let us write the RMSD square fully as:

RMSD2=∑j,k:Mjk=1‖x1j−Ay1k−τ‖2L,
 MathType@MTEF@5@5@+=feaafiart1ev1aaatCvAUfKttLearuWrP9MDH5MBPbIqV92AaeXatLxBI9gBaebbnrfifHhDYfgasaacH8akY=wiFfYdH8Gipec8Eeeu0xXdbba9frFj0=OqFfea0dXdd9vqai=hGuQ8kuc9pgc9s8qqaq=dirpe0xb9q8qiLsFr0=vr0=vr0dc8meaabaqaciaacaGaaeqabaqabeGadaaakeaacqqGsbGucqqGnbqtcqqGtbWucqqGebardaahaaWcbeqaaiabikdaYaaakiabg2da9maalaaabaWaaabuaeaadaqbdaqaaiabdIha4naaBaaaleaacqaIXaqmcqWGQbGAaeqaaOGaeyOeI0IaemyqaeKaemyEaK3aaSbaaSqaaiabigdaXiabdUgaRbqabaGccqGHsisliiGacqWFepaDaiaawMa7caGLkWoadaahaaWcbeqaaiabikdaYaaaaeaacqWGQbGAcqGGSaalcqWGRbWAcqGG6aGocqWGnbqtdaWgaaadbaGaemOAaOMaem4AaSgabeaaliabg2da9iabigdaXaqab0GaeyyeIuoaaOqaaiabdYeambaacqGGSaalaaa@532E@

where L=∑j,k:Mjk=1Mjk
 MathType@MTEF@5@5@+=feaafiart1ev1aaatCvAUfKttLearuWrP9MDH5MBPbIqV92AaeXatLxBI9gBaebbnrfifHhDYfgasaacH8akY=wiFfYdH8Gipec8Eeeu0xXdbba9frFj0=OqFfea0dXdd9vqai=hGuQ8kuc9pgc9s8qqaq=dirpe0xb9q8qiLsFr0=vr0=vr0dc8meaabaqaciaacaGaaeqabaqabeGadaaakeaacqWGmbatcqGH9aqpdaaeqaqaaiabd2eannaaBaaaleaacqWGQbGAcqWGRbWAaeqaaaqaaiabdQgaQjabcYcaSiabdUgaRjabcQda6iabd2eannaaBaaameaacqWGQbGAcqWGRbWAaeqaaSGaeyypa0JaeGymaedabeqdcqGHris5aaaa@3F5B@ or *L *denotes the number of matches.

In the Bayesian formulation the log likelihood (with uniform priors) is proportional to

const.−2(ΣMjk)log⁡σ+(ΣMjk)log⁡ρ−12Qσ22
 MathType@MTEF@5@5@+=feaafiart1ev1aaatCvAUfKttLearuWrP9MDH5MBPbIqV92AaeXatLxBI9gBaebbnrfifHhDYfgasaacH8akY=wiFfYdH8Gipec8Eeeu0xXdbba9frFj0=OqFfea0dXdd9vqai=hGuQ8kuc9pgc9s8qqaq=dirpe0xb9q8qiLsFr0=vr0=vr0dc8meaabaqaciaacaGaaeqabaqabeGadaaakeaacqWGJbWycqWGVbWBcqWGUbGBcqWGZbWCcqWG0baDcqGGUaGlcqGHsislcqaIYaGmcqGGOaakcqqHJoWucqWGnbqtdaWgaaWcbaGaemOAaOMaem4AaSgabeaakiabcMcaPiGbcYgaSjabc+gaVjabcEgaNHGaciab=n8aZjabgUcaRiabcIcaOiabfo6atjabd2eannaaBaaaleaacqWGQbGAcqWGRbWAaeqaaOGaeiykaKIagiiBaWMaei4Ba8Maei4zaCMae8xWdiNaeyOeI0YaaSaaaeaacqaIXaqmaeaacqaIYaGmaaWaaSaaaeaacqWGrbquaeaacqWFdpWCdaahaaWcbeqaaiabikdaYaaakmaakaaabaGaeGOmaidaleqaaaaaaaa@59B1@

where

*Q *= Σ*M*_*jk*_||*x*_1*j *_- *Ay*_1*k *_- *τ*||^2^.

Note that for *M*_*jk *_= 1, we have *Q *= Σ||*x*_1*j *_- *Ay*_1_*k *- *τ*||^2^. It can be seen that *RMSD*^2 ^= *Q*/*L *where *L *is the number of matches. Hence RMSD and *Q *are functionally related. In particular, the maximum likelihood estimate of *σ*^2 ^for a given matching matrix *M *is the same as the RMSD which is the least square estimate.

One way to understand this connection is to go back to the ordinary least square estimates for the regression analysis versus the maximum likelihood estimates under normal errors (i.e. least square versus Gauss-Markov formulation). Sometimes purely algorithmic approach is justified but it has the underlying assumption of normality. The situation here is not too different where we are using likelihood estimators versus the RMSD. Thus in the above context of the statistical methodology and in particular the choice to base the combinatorial objective function on squared Euclidean distance is therefore equivalent to the choice to use Gaussian errors in the probability model. Note that with Gaussian errors in the probability model, the Bayesian modelling approach is very closely related to combinatorial and other methods which maximise objective functions of squared Euclidean deviations e.g. RMSD. In the Bayesian approach, the objective function is a probability distribution (the joint distribution of all unknowns given the data). In the combinatorial approach, the objective function is a measure of mismatch and the procedure is to choose the matching to minimise the objective function e.g. RMSD under some constraints (trivially RMSD can be zero by matching a single point). The two objective functions are very closely related mathematically.

### Side chains orientation

So far each "site" in *X *and *Y *has consisted of a single location, such as a *C*_*α *_atom in an amino acid. But for each *C*_*α *_atom there is also a neighbouring *C*_*β *_atom which can be paired to it. Thus the matching criterion can be extended to require a close match not only for *C*_*α *_atoms in different configurations but also for the edges connecting *C*_*α *_atoms to their *C*_*β *_atoms.

The Bayesian model in Equation 3 is extended here whereby we take into account relative orientation (direction information) of side chains by using *C*_*α *_and *C*_*β *_atoms in matching amino acids. Note that positions of *C*_*α *_and *C*_*β *_atoms from the same amino acid are dependent. Thus *x*_1*j *_and *x*_2*j *_are dependent. Similarly, *y*_1*k *_and *y*_2*k *_are dependent.

To motivate the extension of the model to account for side chain orientations, we assume initially that *x*_1*j *_and *x*_2*j *_are a population configuration whereas *y*_1*k *_and *y*_2*k *_are a sample configuration. Now we can interpret Equation 3 above (which corresponds to equation 6 in [16]) and write

*Ay*_1*k *_+ *τ *|*X*_1*j *_~ *N*(*x*_1*j*_, *σ*^2^*I*).

We extend the model by considering how to model *Ay*_2*k *_given *x*_1*j*_, *x*_2*j *_and *Ay*_1*k*_. Taking into account that there is variation only perpendicular to the axis *x*_2*j *_- *x*_1*j*_, it is plausible to take

*Ay*_2*k*_|*x*_1*j*_, *x*_2*j*_, *y*_1*k*_~ *N*(*x*_2*j *_- *x*_1*j *_+ *Ay*_1*k*_, Ψ)

or

*A*(*y*_2*k *_- *y*_1*k*_) |*x*_1*j*_, *x*_2*j *_, *y*_1*k *_~ *N*(*x*_2*j *_- *x*_1*j *_+ *Ay*_1*k*_, Ψ)

where Ψ = *ψ*_1_*I *+ *ψ*_2 _(*x*_2*j *_- *x*_1*j*_)(*x*_2*j *_- *x*_1*j*_)^*T*^, *ψ*_1 _= *σ*^2^, *ψ*_2 _< 0. The second term of the covariance matrix allows variations only in the plane perpendicular to the *C*_*α *_→ *C*_*β *_axis.

The basic likelihood can be reformulated however we favour a spherical distribution for *A*(*y*_2*k *_- *y*_1*k*_)|*x*_1*j*_, *x*_2*j*_, *y*_1*k *_instead of an elliptical (anisotropic) normal distribution because we want to ensure the symmetry in *X *and *Y *. We note that relative (rather than absolute) values of the probabilities for various matches are essentially important and in practice we do not expect much difference by using an elliptical distribution compared to using the spherical distribution.

Using spherical distribution for *A*(*y*_2*k *_- *y*_1*k*_)|*x*_1*j*_, *x*_2*j*_, *y*_1*k *_case, the joint posterior for *y*_1 _and *y*_2 _given *x*_1 _and *x*_2 _becomes

p(M,A,τ,σ,x1,y1,x2,y2)∝prior×likelihood=|A|np(A)p(τ)p(σ)×∏j,k:Mjk=1κφ({x1j−Ay1k−τ}/σ√2)(σ√2)d×φ({x2j−x1j−A(y2k−y1k)}/γ√2)(γ√2)d.
 MathType@MTEF@5@5@+=feaafiart1ev1aaatCvAUfKttLearuWrP9MDH5MBPbIqV92AaeXatLxBI9gBaebbnrfifHhDYfgasaacH8akY=wiFfYdH8Gipec8Eeeu0xXdbba9frFj0=OqFfea0dXdd9vqai=hGuQ8kuc9pgc9s8qqaq=dirpe0xb9q8qiLsFr0=vr0=vr0dc8meaabaqaciaacaGaaeqabaqabeGadaaakeGabaaMeuaabaqaeeaaaaqaaiabdchaWjabcIcaOiabd2eanjabcYcaSiabdgeabjabcYcaSGGaciab=r8a0jabcYcaSiab=n8aZjabcYcaSiabdIha4naaBaaaleaacqaIXaqmaeqaaOGaeiilaWIaemyEaK3aaSbaaSqaaiabigdaXaqabaGccqGGSaalcqWG4baEdaWgaaWcbaGaeGOmaidabeaakiabcYcaSiabdMha5naaBaaaleaacqaIYaGmaeqaaOGaeiykaKcabiqaaGzacaWLjaGaeyyhIuRaeeiCaaNaeeOCaiNaeeyAaKMaee4Ba8MaeeOCaiNaey41aqRaeeiBaWMaeeyAaKMaee4AaSMaeeyzauMaeeiBaWMaeeyAaKMaeeiAaGMaee4Ba8Maee4Ba8MaeeizaqgabiqaaayacaWLjaGaeyypa0ZaaqWaaeaacqWGbbqqaiaawEa7caGLiWoadaahaaWcbeqaaiabd6gaUbaakiabdchaWjabcIcaOiabdgeabjabcMcaPiabdchaWjabcIcaOiab=r8a0jabcMcaPiabdchaWjabcIcaOiab=n8aZjabcMcaPiabgEna0cqaceaa0fGaaCzcamaarafabaGae8NUdS2aaSaaaeaacqWFgpGzcqGGOaakcqGG7bWEcqWG4baEdaWgaaWcbaGaeGymaeJaemOAaOgabeaakiabgkHiTiabdgeabjabdMha5naaBaaaleaacqaIXaqmcqWGRbWAaeqaaOGaeyOeI0Iae8hXdqNaeiyFa0Naei4la8Iae83WdmhccaGae4NgIyncbaGae0NmaiJae0xkaKcabaGae0hkaGIae83WdmNae4NgIyTae0NmaiJae0xkaKYaaWbaaSqabeaacqWGKbazaaaaaOGaey41aq7aaSaaaeaacqWFgpGzcqGGOaakcqGG7bWEcqWG4baEdaWgaaWcbaGaeGOmaiJaemOAaOgabeaakiabgkHiTiabdIha4naaBaaaleaacqaIXaqmcqWGQbGAaeqaaOGaeyOeI0IaemyqaeKaeiikaGIaemyEaK3aaSbaaSqaaiabikdaYiabdUgaRbqabaGccqGHsislcqWG5bqEdaWgaaWcbaGaeGymaeJaem4AaSgabeaakiabcMcaPiabc2ha9jabc+caViab=n7aNjab+PHiwlab9jdaYiab9LcaPaqaaiab9HcaOiab=n7aNjab+PHiwlab9jdaYiab9LcaPmaaCaaaleqabaGaemizaqgaaaaakiabc6caUaWcbiqaaSn=cqWGQbGAcqGGSaalcqWGRbWAcqGG6aGocqWGnbqtdaWgaaadbaGaemOAaOMaem4AaSgabeaaliabg2da9iabigdaXaqab0Gaey4dIunaaaaaaa@D45F@

We now relax the assumption of *x*_1*j *_and *x*_2*j *_being given in Equation 7 and the joint density does not alter. This is the extension to our use of Equation 3.

The joint posterior distribution *p*(*M*, *A*, *τ*, *σ*, *x*_1_, *y*_1_, *x*_2_, *y*_2_) quantifies uncertainty in the unknown quantities *M*, *A*, *τ*, *σ*, after observing the data. Here t and *σ*^-2 ^are taken to have prior Gaussian and gamma distributions respectively. These priors are plausible for *τ *and *σ *in matching functional sites. The match matrix *M *is *a priori *a uniformly distributed random variable over all match matrices and we sample from this using the Metropolis algorithm (see below – updating *M*) with a move set consisting of proposals to add a match, delete a match or switch matches. The rotation matrix *A *is assumed to have prior matrix Fisher distribution [21,22].

Markov chain Monte Carlo (MCMC) methods are used to sample from the full joint distribution in Equation 7. This provides an extremely flexible basis for reporting various aspects of the full joint posterior *p*(*M*, *A*, *τ*, *σ*, *x*_1_, *y*_1_, *x*_2_, *y*_2_) that are of interest. For example the maximum of this function would provide a (naive) point estimate of the unknowns.

Note that Equation 7 takes us back into the framework of sample configurations. We now indicate how the model extends the concept of the RMSD for the paired case. We show that the "objective" in Equation 7 is a function of RMSD (for *C*_*α *_atoms only in Equation 4) and the angle for orientation difference between the matched amino acids. Denote the RMSD for *C*_*α *_atoms only in Equation 4 as *RMSD*_*α*_. Let *v*_1 _= *x*_2*j *_- *x*_1*j *_and *v*_2 _= *y*_2*k *_- *y*_1*k*_. Equation 7 is a function of *RMSD*_*α *_+ ||*v*_1 _- *Av*_2_||^2^. Note that ||*v*_1 _- *Av*_2_||^2 ^= ||*v*_1_||^2 ^+ ||*v*_2_||^2 ^- 2v1Tv2
 MathType@MTEF@5@5@+=feaafiart1ev1aaatCvAUfKttLearuWrP9MDH5MBPbIqV92AaeXatLxBI9gBaebbnrfifHhDYfgasaacH8akY=wiFfYdH8Gipec8Eeeu0xXdbba9frFj0=OqFfea0dXdd9vqai=hGuQ8kuc9pgc9s8qqaq=dirpe0xb9q8qiLsFr0=vr0=vr0dc8meaabaqaciaacaGaaeqabaqabeGadaaakeaacqaIYaGmcqWG2bGDdaqhaaWcbaGaeGymaedabaGaemivaqfaaOGaemODay3aaSbaaSqaaiabikdaYaqabaaaaa@33FE@ and ||*v*_1_|| ≈ ||*v*_2_|| ≈ 1.54Å; v1Tv2
 MathType@MTEF@5@5@+=feaafiart1ev1aaatCvAUfKttLearuWrP9MDH5MBPbIqV92AaeXatLxBI9gBaebbnrfifHhDYfgasaacH8akY=wiFfYdH8Gipec8Eeeu0xXdbba9frFj0=OqFfea0dXdd9vqai=hGuQ8kuc9pgc9s8qqaq=dirpe0xb9q8qiLsFr0=vr0=vr0dc8meaabaqaciaacaGaaeqabaqabeGadaaakeaacqWG2bGDdaqhaaWcbaGaeGymaedabaGaemivaqfaaOGaemODay3aaSbaaSqaaiabikdaYaqabaaaaa@330C@ = ||*v*_1_|| · ||*v*_2_|| cos *θ *≈ 1.54^2^cos *θ *where *θ *is the angle between *v*_1 _= *x*_2*j *_- *x*_1*j *_and *v*_2 _= *y*_2*k *_- *y*_1*k *_i.e. the angle between *C*_*α *_→ *Cβ *directions for matched amino acids. Thus ||*v*_1 _- *Av*_2_||^2 ^≈ 2 × 1.54^2^(1 - cos *θ*) and the "objective" in Equation 7 involves the vector RMSD,

*RMSD*_*αβ *_= *RMSD*_*α *_+ ||*v*_1_||^2 ^+ ||*v*_2_||^2 ^- 2 × ||*v*_1_||·||*v*_2_|| cos *θ ≈ RMSD*_*α *_+ 2 × 1.54^2^(1 - cos *θ*).

Thus the method extends RMSD for *C*_*α *_atoms in the objective function (model) by adding angles in the paired (*C*_*α*_, *C*_*β*_) points.

### Computational implementation

The additional term in the new full likelihood does not involve *τ *hence the posterior and updating of *τ *is unchanged to that in [16].

### Rotation matrix

The full conditional distribution of *A *is

p(A|M,τ,σ,X,Y)∝|A|2np(A)×∏j,k:Mjk=1φ(x1j−Ay1k−τσ2)×∏j,k:Mjk=1φ(x2j−x1j−A(y2k−y1k)γ2).
 MathType@MTEF@5@5@+=feaafiart1ev1aaatCvAUfKttLearuWrP9MDH5MBPbIqV92AaeXatLxBI9gBaebbnrfifHhDYfgasaacH8akY=wiFfYdH8Gipec8Eeeu0xXdbba9frFj0=OqFfea0dXdd9vqai=hGuQ8kuc9pgc9s8qqaq=dirpe0xb9q8qiLsFr0=vr0=vr0dc8meaabaqaciaacaGaaeqabaqabeGadaaakeaafaqaaeWabaaabaGaemiCaaNaeiikaGIaemyqaeKaeiiFaWNaemyta0KaeiilaWccciGae8hXdqNaeiilaWIae83WdmNaeiilaWIaemiwaGLaeiilaWIaemywaKLaeiykaKIaeyyhIu7aaqWaaeaacqWGbbqqaiaawEa7caGLiWoadaahaaWcbeqaaiabikdaYiabd6gaUbaakiabdchaWjabcIcaOiabdgeabjabcMcaPaqaaiabgEna0oaarafabaGae8NXdy2aaeWaaeaadaWcaaqaaiabdIha4naaBaaaleaacqaIXaqmcqWGQbGAaeqaaOGaeyOeI0IaemyqaeKaemyEaK3aaSbaaSqaaiabigdaXiabdUgaRbqabaGccqGHsislcqWFepaDaeaacqWFdpWCdaGcaaqaaiabikdaYaWcbeaaaaaakiaawIcacaGLPaaaaSqaaiabdQgaQjabcYcaSiabdUgaRjabcQda6iabd2eannaaBaaameaacqWGQbGAcqWGRbWAaeqaaSGaeyypa0JaeGymaedabeqdcqGHpis1aaGcbaGaey41aq7aaebuaeaacqWFgpGzdaqadaqaamaalaaabaGaemiEaG3aaSbaaSqaaiabikdaYiabdQgaQbqabaGccqGHsislcqWG4baEdaWgaaWcbaGaeGymaeJaemOAaOgabeaakiabgkHiTiabdgeabjabcIcaOiabdMha5naaBaaaleaacqaIYaGmcqWGRbWAaeqaaOGaeyOeI0IaemyEaK3aaSbaaSqaaiabigdaXiabdUgaRbqabaGccqGGPaqkaeaacqWFZoWzdaGcaaqaaiabikdaYaWcbeaaaaaakiaawIcacaGLPaaacqGGUaGlaSqaaiabdQgaQjabcYcaSiabdUgaRjabcQda6iabd2eannaaBaaameaacqWGQbGAcqWGRbWAaeqaaSGaeyypa0JaeGymaedabeqdcqGHpis1aaaaaaa@9635@

Thus

p(A|M,τ,σ,X,Y)∝p(A)×exp⁡(tr{12σ2∑y1k(x1j−τ)TA})×exp⁡(12γ2∑(x2j−x1j)TA(y2k−y1k))     ∝exp⁡(tr{12σ2∑y1k(x1j−τ)TA})×exp⁡(tr{12γ2∑(y2k−y1k)(x2j−x1j)TA})∝exp⁡(tr{HA})
 MathType@MTEF@5@5@+=feaafiart1ev1aaatCvAUfKttLearuWrP9MDH5MBPbIqV92AaeXatLxBI9gBaebbnrfifHhDYfgasaacH8akY=wiFfYdH8Gipec8Eeeu0xXdbba9frFj0=OqFfea0dXdd9vqai=hGuQ8kuc9pgc9s8qqaq=dirpe0xb9q8qiLsFr0=vr0=vr0dc8meaabaqaciaacaGaaeqabaqabeGadaaakeaafaqaaeGbbaaaaeaacqWGWbaCcqGGOaakcqWGbbqqcqGG8baFcqWGnbqtcqGGSaaliiGacqWFepaDcqGGSaalcqWFdpWCcqGGSaalcqWGybawcqGGSaalcqWGzbqwcqGGPaqkaeGacaaaaaaNaiaaxMaacqGHDisTcqWGWbaCcqGGOaakcqWGbbqqcqGGPaqkcqGHxdaTcyGGLbqzcqGG4baEcqGGWbaCdaqadaqaaiabbsha0jabbkhaYnaacmqabaWaaSaaaeaacqaIXaqmaeaacqaIYaGmcqWFdpWCdaahaaWcbeqaaiabikdaYaaaaaGcdaaeabqaaiabdMha5naaBaaaleaacqaIXaqmcqWGRbWAaeqaaOGaeiikaGIaemiEaG3aaSbaaSqaaiabigdaXiabdQgaQbqabaGccqGHsislcqWFepaDcqGGPaqkdaahaaWcbeqaaiabdsfaubaakiabdgeabbWcbeqab0GaeyyeIuoaaOGaay5Eaiaaw2haaaGaayjkaiaawMcaaaqaciaaefaaCfGaaCzcaiabgEna0kGbcwgaLjabcIha4jabcchaWnaabmaabaWaaSaaaeaacqaIXaqmaeaacqaIYaGmcqWFZoWzdaahaaWcbeqaaiabikdaYaaaaaGcdaaeabqaaiabcIcaOiabdIha4naaBaaaleaacqaIYaGmcqWGQbGAaeqaaOGaeyOeI0IaemiEaG3aaSbaaSqaaiabigdaXiabdQgaQbqabaGccqGGPaqkdaahaaWcbeqaaiabdsfaubaakiabdgeabjabcIcaOiabdMha5naaBaaaleaacqaIYaGmcqWGRbWAaeqaaOGaeyOeI0IaemyEaK3aaSbaaSqaaiabigdaXiabdUgaRbqabaGccqGGPaqkaSqabeqaniabggHiLdaakiaawIcacaGLPaaaaeGacaavaaaEaiaaxMaacaWLjaGaeyyhIuRagiyzauMaeiiEaGNaeiiCaa3aaeWaaeaacqqG0baDcqqGYbGCdaGadeqaamaalaaabaGaeGymaedabaGaeGOmaiJae83Wdm3aaWbaaSqabeaacqaIYaGmaaaaaOWaaabqaeaacqWG5bqEdaWgaaWcbaGaeGymaeJaem4AaSgabeaakiabcIcaOiabdIha4naaBaaaleaacqaIXaqmcqWGQbGAaeqaaOGaeyOeI0Iae8hXdqNaeiykaKYaaWbaaSqabeaacqWGubavaaGccqWGbbqqaSqabeqaniabggHiLdaakiaawUhacaGL9baaaiaawIcacaGLPaaaaeGabaarbiaaxMaacqGHxdaTcyGGLbqzcqGG4baEcqGGWbaCdaqadaqaaiabbsha0jabbkhaYnaacmqabaWaaSaaaeaacqaIXaqmaeaacqaIYaGmcqWFZoWzdaahaaWcbeqaaiabikdaYaaaaaGcdaaeabqaaiabcIcaOiabdMha5naaBaaaleaacqaIYaGmcqWGRbWAaeqaaOGaeyOeI0IaemyEaK3aaSbaaSqaaiabigdaXiabdUgaRbqabaGccqGGPaqkcqGGOaakcqWG4baEdaWgaaWcbaGaeGOmaiJaemOAaOgabeaakiabgkHiTiabdIha4naaBaaaleaacqaIXaqmcqWGQbGAaeqaaOGaeiykaKYaaWbaaSqabeaacqWGubavaaGccqWGbbqqaSqabeqaniabggHiLdaakiaawUhacaGL9baaaiaawIcacaGLPaaaaeGacaayaaaNaiaaxMaacqGHDisTcyGGLbqzcqGG4baEcqGGWbaCcqGGOaakcqqG0baDcqqGYbGCcqGG7bWEcqWGibascqWGbbqqcqGG9bqFcqGGPaqkaaaaaa@EFB2@

where the summation is over *j*, *k*:*M*_*jk *_= 1 and

H=12σ2∑y1k(x1j−τ)T+12γ2∑(y2k−y1k)(x2j−x1j)T.
 MathType@MTEF@5@5@+=feaafiart1ev1aaatCvAUfKttLearuWrP9MDH5MBPbIqV92AaeXatLxBI9gBaebbnrfifHhDYfgasaacH8akY=wiFfYdH8Gipec8Eeeu0xXdbba9frFj0=OqFfea0dXdd9vqai=hGuQ8kuc9pgc9s8qqaq=dirpe0xb9q8qiLsFr0=vr0=vr0dc8meaabaqaciaacaGaaeqabaqabeGadaaakeaacqWGibascqGH9aqpdaWcaaqaaiabigdaXaqaaiabikdaYGGaciab=n8aZnaaCaaaleqabaGaeGOmaidaaaaakmaaqaeabaGaemyEaK3aaSbaaSqaaiabigdaXiabdUgaRbqabaGccqGGOaakcqWG4baEdaWgaaWcbaGaeGymaeJaemOAaOgabeaakiabgkHiTiab=r8a0jabcMcaPmaaCaaaleqabaGaemivaqfaaaqabeqaniabggHiLdGccqGHRaWkdaWcaaqaaiabigdaXaqaaiabikdaYiab=n7aNnaaCaaaleqabaGaeGOmaidaaaaakmaaqaeabaGaeiikaGIaemyEaK3aaSbaaSqaaiabikdaYiabdUgaRbqabaGccqGHsislcqWG5bqEdaWgaaWcbaGaeGymaeJaem4AaSgabeaakiabcMcaPiabcIcaOiabdIha4naaBaaaleaacqaIYaGmcqWGQbGAaeqaaOGaeyOeI0IaemiEaG3aaSbaaSqaaiabigdaXiabdQgaQbqabaGccqGGPaqkdaahaaWcbeqaaiabdsfaubaaaeqabeqdcqGHris5aOGaeiOla4caaa@62AA@

Thus with *p*(*A*) ∝ exp(trF0T
 MathType@MTEF@5@5@+=feaafiart1ev1aaatCvAUfKttLearuWrP9MDH5MBPbIqV92AaeXatLxBI9gBaebbnrfifHhDYfgasaacH8akY=wiFfYdH8Gipec8Eeeu0xXdbba9frFj0=OqFfea0dXdd9vqai=hGuQ8kuc9pgc9s8qqaq=dirpe0xb9q8qiLsFr0=vr0=vr0dc8meaabaqaciaacaGaaeqabaqabeGadaaakeaacqWGgbGrdaqhaaWcbaGaeGimaadabaGaemivaqfaaaaa@300D@*A*) for some matrix *F*_0_, then the full conditional distribution of *A *given data and values for all other parameters has the same form with *F*_0 _replaced by

F=F0+(1/2σ2)∑j,k:Mjk=1(x1j−τ)y1kT+(1/2γ2)∑j,k:Mjk=1(x2j−x1j)(y2k−y1k)T.
 MathType@MTEF@5@5@+=feaafiart1ev1aaatCvAUfKttLearuWrP9MDH5MBPbIqV92AaeXatLxBI9gBaebbnrfifHhDYfgasaacH8akY=wiFfYdH8Gipec8Eeeu0xXdbba9frFj0=OqFfea0dXdd9vqai=hGuQ8kuc9pgc9s8qqaq=dirpe0xb9q8qiLsFr0=vr0=vr0dc8meaabaqaciaacaGaaeqabaqabeGadaaakeaafaqadeGabaaabaGaemOrayKaeyypa0JaemOray0aaSbaaSqaaiabicdaWaqabaGccqGHRaWkcqGGOaakcqaIXaqmcqGGVaWlcqaIYaGmiiGacqWFdpWCdaahaaWcbeqaaiabikdaYaaakiabcMcaPmaaqafabaGaeiikaGIaemiEaG3aaSbaaSqaaiabigdaXiabdQgaQbqabaGccqGHsislcqWFepaDcqGGPaqkcqWG5bqEdaqhaaWcbaGaeGymaeJaem4AaSgabaGaemivaqfaaaqaaiabdQgaQjabcYcaSiabdUgaRjabcQda6iabd2eannaaBaaameaacqWGQbGAcqWGRbWAaeqaaSGaeyypa0JaeGymaedabeqdcqGHris5aaGcbaGaey4kaSIaeiikaGIaeGymaeJaei4la8IaeGOmaiJae83SdC2aaWbaaSqabeaacqaIYaGmaaGccqGGPaqkdaaeqbqaaiabcIcaOiabdIha4naaBaaaleaacqaIYaGmcqWGQbGAaeqaaOGaeyOeI0IaemiEaG3aaSbaaSqaaiabigdaXiabdQgaQbqabaGccqGGPaqkcqGGOaakcqWG5bqEdaWgaaWcbaGaeGOmaiJaem4AaSgabeaakiabgkHiTiabdMha5naaBaaaleaacqaIXaqmcqWGRbWAaeqaaOGaeiykaKYaaWbaaSqabeaacqWGubavaaaabaGaemOAaOMaeiilaWIaem4AaSMaeiOoaOJaemyta00aaSbaaWqaaiabdQgaQjabdUgaRbqabaWccqGH9aqpcqaIXaqmaeqaniabggHiLdGccqGGUaGlaaaaaa@8012@

#### Updating M

It can be shown (equation 8 in [16]) that acceptance probability for adding a match (*j*, *k*) is min(1, *a*_*r*_),

ar=κφ({x1j−Ay1k−τ}/σ2)p∗nu(σ2)d×φ({x2j−x1j−A(y2k−y1k}/γ2)(γ2)d.
 MathType@MTEF@5@5@+=feaafiart1ev1aaatCvAUfKttLearuWrP9MDH5MBPbIqV92AaeXatLxBI9gBaebbnrfifHhDYfgasaacH8akY=wiFfYdH8Gipec8Eeeu0xXdbba9frFj0=OqFfea0dXdd9vqai=hGuQ8kuc9pgc9s8qqaq=dirpe0xb9q8qiLsFr0=vr0=vr0dc8meaabaqaciaacaGaaeqabaqabeGadaaakeaacqWGHbqydaWgaaWcbaGaemOCaihabeaakiabg2da9maalaaabaacciGae8NUdSMae8NXdyMaeiikaGIaei4EaSNaemiEaG3aaSbaaSqaaiabigdaXiabdQgaQbqabaGccqGHsislcqWGbbqqcqWG5bqEdaWgaaWcbaGaeGymaeJaem4AaSgabeaakiabgkHiTiab=r8a0jabc2ha9jabc+caViab=n8aZnaakaaabaGaeGOmaidaleqaaOGaeiykaKIaemiCaa3aaWbaaSqabeaacqGHxiIkaaGccqWGUbGBdaWgaaWcbaGaemyDauhabeaaaOqaaiabcIcaOiab=n8aZnaakaaabaGaeGOmaidaleqaaOGaeiykaKYaaWbaaSqabeaacqWGKbazaaaaaOGaey41aq7aaSaaaeaacqWFgpGzcqGGOaakcqGG7bWEcqWG4baEdaWgaaWcbaGaeGOmaiJaemOAaOgabeaakiabgkHiTiabdIha4naaBaaaleaacqaIXaqmcqWGQbGAaeqaaOGaeyOeI0IaemyqaeKaeiikaGIaemyEaK3aaSbaaSqaaiabikdaYiabdUgaRbqabaGccqGHsislcqWG5bqEdaWgaaWcbaGaeGymaeJaem4AaSgabeaakiabc2ha9jabc+caViab=n7aNnaakaaabaGaeGOmaidaleqaaOGaeiykaKcabaGaeiikaGIae83SdC2aaOaaaeaacqaIYaGmaSqabaGccqGGPaqkdaahaaWcbeqaaiabdsgaKbaaaaGccqGGUaGlaaa@7C80@

Similarly, the acceptance probability for switching the match of *X*_*j *_from *Y*_*k *_to *Y*_*k' *_(equation 9 in [16]) is min(1, *a*_*r*_),

ar=φ({x1j−Ay1k′−τ}/σ2)φ({x1j−Ay1k−τ}/σ2)×φ({x2j−x1j−A(y2k′−y1k′}/γ2)φ({x2j−x1j−A(y2k−y1k}/γ2)
 MathType@MTEF@5@5@+=feaafiart1ev1aaatCvAUfKttLearuWrP9MDH5MBPbIqV92AaeXatLxBI9gBaebbnrfifHhDYfgasaacH8akY=wiFfYdH8Gipec8Eeeu0xXdbba9frFj0=OqFfea0dXdd9vqai=hGuQ8kuc9pgc9s8qqaq=dirpe0xb9q8qiLsFr0=vr0=vr0dc8meaabaqaciaacaGaaeqabaqabeGadaaakeaacqWGHbqydaWgaaWcbaGaemOCaihabeaakiabg2da9maalaaabaacciGae8NXdyMaeiikaGIaei4EaSNaemiEaG3aaSbaaSqaaiabigdaXiabdQgaQbqabaGccqGHsislcqWGbbqqcqWG5bqEdaWgaaWcbaGaeGymaeJafm4AaSMbauaaaeqaaOGaeyOeI0Iae8hXdqNaeiyFa0Naei4la8Iae83Wdm3aaOaaaeaacqaIYaGmaSqabaGccqGGPaqkaeaacqWFgpGzcqGGOaakcqGG7bWEcqWG4baEdaWgaaWcbaGaeGymaeJaemOAaOgabeaakiabgkHiTiabdgeabjabdMha5naaBaaaleaacqaIXaqmcqWGRbWAaeqaaOGaeyOeI0Iae8hXdqNaeiyFa0Naei4la8Iae83Wdm3aaOaaaeaacqaIYaGmaSqabaGccqGGPaqkaaGaey41aq7aaSaaaeaacqWFgpGzcqGGOaakcqGG7bWEcqWG4baEdaWgaaWcbaGaeGOmaiJaemOAaOgabeaakiabgkHiTiabdIha4naaBaaaleaacqaIXaqmcqWGQbGAaeqaaOGaeyOeI0IaemyqaeKaeiikaGIaemyEaK3aaSbaaSqaaiabikdaYiqbdUgaRzaafaaabeaakiabgkHiTiabdMha5naaBaaaleaacqaIXaqmcuWGRbWAgaqbaaqabaGccqGG9bqFcqGGVaWlcqWFZoWzdaGcaaqaaiabikdaYaWcbeaakiabcMcaPaqaaiab=z8aMjabcIcaOiabcUha7jabdIha4naaBaaaleaacqaIYaGmcqWGQbGAaeqaaOGaeyOeI0IaemiEaG3aaSbaaSqaaiabigdaXiabdQgaQbqabaGccqGHsislcqWGbbqqcqGGOaakcqWG5bqEdaWgaaWcbaGaeGOmaiJaem4AaSgabeaakiabgkHiTiabdMha5naaBaaaleaacqaIXaqmcqWGRbWAaeqaaOGaeiyFa0Naei4la8Iae83SdC2aaOaaaeaacqaIYaGmaSqabaGccqGGPaqkaaaaaa@9DE1@

and for deleting the match (*j*, *k*) is min(1, *a*_*r*_) where

ar=(σ2)dκφ({x1j−Ay1k−τ}/σ2)p∗nu×(γ2)dφ({x2j−x1j−A(y2k−y1k}/γ2.
 MathType@MTEF@5@5@+=feaafiart1ev1aaatCvAUfKttLearuWrP9MDH5MBPbIqV92AaeXatLxBI9gBaebbnrfifHhDYfgasaacH8akY=wiFfYdH8Gipec8Eeeu0xXdbba9frFj0=OqFfea0dXdd9vqai=hGuQ8kuc9pgc9s8qqaq=dirpe0xb9q8qiLsFr0=vr0=vr0dc8meaabaqaciaacaGaaeqabaqabeGadaaakeaacqWGHbqydaWgaaWcbaGaemOCaihabeaakiabg2da9maalaaabaGaeiikaGccciGae83Wdm3aaOaaaeaacqaIYaGmaSqabaGccqGGPaqkdaahaaWcbeqaaiabdsgaKbaaaOqaaiab=P7aRjab=z8aMjabcIcaOiabcUha7jabdIha4naaBaaaleaacqaIXaqmcqWGQbGAaeqaaOGaeyOeI0IaemyqaeKaemyEaK3aaSbaaSqaaiabigdaXiabdUgaRbqabaGccqGHsislcqWFepaDcqGG9bqFcqGGVaWlcqWFdpWCdaGcaaqaaiabikdaYaWcbeaakiabcMcaPiabdchaWnaaCaaaleqabaGaey4fIOcaaOGaemOBa42aaSbaaSqaaiabdwha1bqabaaaaOGaey41aq7aaSaaaeaacqGGOaakcqWFZoWzdaGcaaqaaiabikdaYaWcbeaakiabcMcaPmaaCaaaleqabaGaemizaqgaaaGcbaGae8NXdyMaeiikaGIaei4EaSNaemiEaG3aaSbaaSqaaiabikdaYiabdQgaQbqabaGccqGHsislcqWG4baEdaWgaaWcbaGaeGymaeJaemOAaOgabeaakiabgkHiTiabdgeabjabcIcaOiabdMha5naaBaaaleaacqaIYaGmcqWGRbWAaeqaaOGaeyOeI0IaemyEaK3aaSbaaSqaaiabigdaXiabdUgaRbqabaGccqGG9bqFcqGGVaWlcqWFZoWzdaGcaaqaaiabikdaYaWcbeaaaaGccqGGUaGlaaa@7BA6@

#### Point estimates for A, τ and M

Posterior means are taken as estimates for transformation parameters A and *τ *[16].

#### Point estimate for the match matrix, M

Assignments are made to minimise error rates: *P*(M^jk
 MathType@MTEF@5@5@+=feaafiart1ev1aaatCvAUfKttLearuWrP9MDH5MBPbIqV92AaeXatLxBI9gBaebbnrfifHhDYfgasaacH8akY=wiFfYdH8Gipec8Eeeu0xXdbba9frFj0=OqFfea0dXdd9vqai=hGuQ8kuc9pgc9s8qqaq=dirpe0xb9q8qiLsFr0=vr0=vr0dc8meaabaqaciaacaGaaeqabaqabeGadaaakeaacuWGnbqtgaqcamaaBaaaleaacqWGQbGAcqWGRbWAaeqaaaaa@30C7@ = 1|*M*_*jk *_= 0) and *P*(M^jk
 MathType@MTEF@5@5@+=feaafiart1ev1aaatCvAUfKttLearuWrP9MDH5MBPbIqV92AaeXatLxBI9gBaebbnrfifHhDYfgasaacH8akY=wiFfYdH8Gipec8Eeeu0xXdbba9frFj0=OqFfea0dXdd9vqai=hGuQ8kuc9pgc9s8qqaq=dirpe0xb9q8qiLsFr0=vr0=vr0dc8meaabaqaciaacaGaaeqabaqabeGadaaakeaacuWGnbqtgaqcamaaBaaaleaacqWGQbGAcqWGRbWAaeqaaaaa@30C7@ = 0|*M*_*jk *_= 1).

Suppose that the loss is ℓ_*ab *_for declaring M^jk
 MathType@MTEF@5@5@+=feaafiart1ev1aaatCvAUfKttLearuWrP9MDH5MBPbIqV92AaeXatLxBI9gBaebbnrfifHhDYfgasaacH8akY=wiFfYdH8Gipec8Eeeu0xXdbba9frFj0=OqFfea0dXdd9vqai=hGuQ8kuc9pgc9s8qqaq=dirpe0xb9q8qiLsFr0=vr0=vr0dc8meaabaqaciaacaGaaeqabaqabeGadaaakeaadaqiaaqaaiabd2eanbGaayPadaWaaSbaaSqaaiabdQgaQjabdUgaRbqabaaaaa@3179@ = *b *for *b *= 0, 1 when *p*_*jk *_= *P*(*M*_*jk *_= *a*), *a *= 0, 1; for example, ℓ_01 _is the loss associated with declaring a match between *X*_*j *_and *Y*_*k *_when there is really none, that is, a "false positive". A false negative is ℓ_10_. An optimal Bayesian point estimate of *M *is given using a loss function which penalises false positive (ℓ_01_) and false negative (ℓ_10_) matches. This optimum is controlled by a cost ratio *K *= ℓ_01_/(ℓ_01 _+ ℓ_10_). Note that for *K *= 0.5 false positive and false negative matches are equally undesirable whereas for *K *= 0.75 i.e. ℓ_01 _= 3 × ℓ_10 _used in this paper, accepting matches when there are no "true" matches is heavily penalised.

We minimise the expected sum of losses at each entry of *M *with respect to the MCMC chain target distribution (the posterior distribution) by using empirical estimates of matching probabilities *p*_*jk *_= ∑*M*_*jk *_where the sum is taken over the number of iterations after burn-in. That is we are averaging over *M *and we minimise

E[L(M,M^)|x,y]=∑j,kM^jkℓ11pjk+∑j,kM^jkℓ01(1−pjk)+∑j,k(1−M^jk)ℓ10pjk+∑j,k(1−M^jk)ℓ00(1−pjk)=∑j,kM^jk(ℓ11pjk−ℓ01pjk−ℓ10pjk+ℓ00pjk+ℓ01−ℓ00)+∑j,k(ℓ00+ℓ10pjk−ℓ00pjk)=∑j,kM^jk((ℓ11−ℓ01−ℓ10+ℓ00)pjk+ℓ01−ℓ00)+∑j,k(ℓ00+ℓ10pjk−ℓ00pjk).
 MathType@MTEF@5@5@+=feaafiart1ev1aaatCvAUfKttLearuWrP9MDH5MBPbIqV92AaeXatLxBI9gBaebbnrfifHhDYfgasaacH8akY=wiFfYdH8Gipec8Eeeu0xXdbba9frFj0=OqFfea0dXdd9vqai=hGuQ8kuc9pgc9s8qqaq=dirpe0xb9q8qiLsFr0=vr0=vr0dc8meaabaqaciaacaGaaeqabaqabeGadaaakeGabaaX=xaabaqageaaaaqaaiabdweafjabcUfaBjabdYeamjabcIcaOiabd2eanjabcYcaSmaaHaaabaGaemyta0eacaGLcmaacqGGPaqkcqGG8baFcqWG4baEcqGGSaalcqWG5bqEcqGGDbqxaeGacaa2aaalbiaaxMaacqGH9aqpdaaeqbqaamaaHaaabaGaemyta0eacaGLcmaadaWgaaWcbaGaemOAaOMaem4AaSgabeaakiabloriSnaaBaaaleaacqaIXaqmcqaIXaqmaeqaaOGaemiCaa3aaSbaaSqaaiabdQgaQjabdUgaRbqabaaabaGaemOAaOMaeiilaWIaem4AaSgabeqdcqGHris5aOGaey4kaSYaaabuaeaadaqiaaqaaiabd2eanbGaayPadaWaaSbaaSqaaiabdQgaQjabdUgaRbqabaGccqWItecBdaWgaaWcbaGaeGimaaJaeGymaedabeaakiabcIcaOiabigdaXiabgkHiTiabdchaWnaaBaaaleaacqWGQbGAcqWGRbWAaeqaaOGaeiykaKcaleaacqWGQbGAcqGGSaalcqWGRbWAaeqaniabggHiLdGccqGHRaWkdaaeqbqaaiabcIcaOiabigdaXiabgkHiTmaaHaaabaGaemyta0eacaGLcmaadaWgaaWcbaGaemOAaOMaem4AaSgabeaakiabcMcaPiabloriSnaaBaaaleaacqaIXaqmcqaIWaamaeqaaOGaemiCaa3aaSbaaSqaaiabdQgaQjabdUgaRbqabaaabaGaemOAaOMaeiilaWIaem4AaSgabeqdcqGHris5aaGcbiqaaqfacaWLjaGaey4kaSYaaabuaeaacqGGOaakcqaIXaqmcqGHsisldaqiaaqaaiabd2eanbGaayPadaWaaSbaaSqaaiabdQgaQjabdUgaRbqabaGccqGGPaqkcqWItecBdaWgaaWcbaGaeGimaaJaeGimaadabeaakiabcIcaOiabigdaXiabgkHiTiabdchaWnaaBaaaleaacqWGQbGAcqWGRbWAaeqaaOGaeiykaKcaleaacqWGQbGAcqGGSaalcqWGRbWAaeqaniabggHiLdaakeGabaaEaiaaxMaacqGH9aqpdaaeqbqaamaaHaaabaGaemyta0eacaGLcmaadaWgaaWcbaGaemOAaOMaem4AaSgabeaakiabcIcaOiabloriSnaaBaaaleaacqaIXaqmcqaIXaqmaeqaaOGaemiCaa3aaSbaaSqaaiabdQgaQjabdUgaRbqabaGccqGHsislcqWItecBdaWgaaWcbaGaeGimaaJaeGymaedabeaakiabdchaWnaaBaaaleaacqWGQbGAcqWGRbWAaeqaaOGaeyOeI0IaeS4eHW2aaSbaaSqaaiabigdaXiabicdaWaqabaGccqWGWbaCdaWgaaWcbaGaemOAaOMaem4AaSgabeaakiabgUcaRiabloriSnaaBaaaleaacqaIWaamcqaIWaamaeqaaOGaemiCaa3aaSbaaSqaaiabdQgaQjabdUgaRbqabaGccqGHRaWkcqWItecBdaWgaaWcbaGaeGimaaJaeGymaedabeaakiabgkHiTiabloriSnaaBaaaleaacqaIWaamcqaIWaamaeqaaOGaeiykaKcaleaacqWGQbGAcqGGSaalcqWGRbWAaeqaniabggHiLdaakeGabaaKaiaaxMaacqGHRaWkdaaeqbqaaiabcIcaOiabloriSnaaBaaaleaacqaIWaamcqaIWaamaeqaaOGaey4kaSIaeS4eHW2aaSbaaSqaaiabigdaXiabicdaWaqabaGccqWGWbaCdaWgaaWcbaGaemOAaOMaem4AaSgabeaakiabgkHiTiabloriSnaaBaaaleaacqaIWaamcqaIWaamaeqaaOGaemiCaa3aaSbaaSqaaiabdQgaQjabdUgaRbqabaGccqGGPaqkaSqaaiabdQgaQjabcYcaSiabdUgaRbqab0GaeyyeIuoaaOqaceaaGbGaaCzcaiabg2da9maaqafabaWaaecaaeaacqWGnbqtaiaawkWaamaaBaaaleaacqWGQbGAcqWGRbWAaeqaaOGaeiikaGIaeiikaGIaeS4eHW2aaSbaaSqaaiabigdaXiabigdaXaqabaGccqGHsislcqWItecBdaWgaaWcbaGaeGimaaJaeGymaedabeaakiabgkHiTiabloriSnaaBaaaleaacqaIXaqmcqaIWaamaeqaaOGaey4kaSIaeS4eHW2aaSbaaSqaaiabicdaWiabicdaWaqabaGccqGGPaqkcqWGWbaCdaWgaaWcbaGaemOAaOMaem4AaSgabeaakiabgUcaRiabloriSnaaBaaaleaacqaIWaamcqaIXaqmaeqaaOGaeyOeI0IaeS4eHW2aaSbaaSqaaiabicdaWiabicdaWaqabaGccqGGPaqkaSqaaiabdQgaQjabcYcaSiabdUgaRbqab0GaeyyeIuoakiabgUcaRmaaqafabaGaeiikaGIaeS4eHW2aaSbaaSqaaiabicdaWiabicdaWaqabaGccqGHRaWkcqWItecBdaWgaaWcbaGaeGymaeJaeGimaadabeaakiabdchaWnaaBaaaleaacqWGQbGAcqWGRbWAaeqaaOGaeyOeI0IaeS4eHW2aaSbaaSqaaiabicdaWiabicdaWaqabaGccqWGWbaCdaWgaaWcbaGaemOAaOMaem4AaSgabeaakiabcMcaPaWcbaGaemOAaOMaeiilaWIaem4AaSgabeqdcqGHris5aOGaeiOla4caaaaa@3B96@

The last sum does not depend on M^jk
 MathType@MTEF@5@5@+=feaafiart1ev1aaatCvAUfKttLearuWrP9MDH5MBPbIqV92AaeXatLxBI9gBaebbnrfifHhDYfgasaacH8akY=wiFfYdH8Gipec8Eeeu0xXdbba9frFj0=OqFfea0dXdd9vqai=hGuQ8kuc9pgc9s8qqaq=dirpe0xb9q8qiLsFr0=vr0=vr0dc8meaabaqaciaacaGaaeqabaqabeGadaaakeaadaqiaaqaaiabd2eanbGaayPadaWaaSbaaSqaaiabdQgaQjabdUgaRbqabaaaaa@3179@, hence interested in minimising the first part:

−(ℓ01+ℓ10−ℓ11−ℓ00)∑j,k:M^jk=1(pjk−K)
 MathType@MTEF@5@5@+=feaafiart1ev1aaatCvAUfKttLearuWrP9MDH5MBPbIqV92AaeXatLxBI9gBaebbnrfifHhDYfgasaacH8akY=wiFfYdH8Gipec8Eeeu0xXdbba9frFj0=OqFfea0dXdd9vqai=hGuQ8kuc9pgc9s8qqaq=dirpe0xb9q8qiLsFr0=vr0=vr0dc8meaabaqaciaacaGaaeqabaqabeGadaaakeaacqGHsislcqGGOaakcqWItecBdaWgaaWcbaGaeGimaaJaeGymaedabeaakiabgUcaRiabloriSnaaBaaaleaacqaIXaqmcqaIWaamaeqaaOGaeyOeI0IaeS4eHW2aaSbaaSqaaiabigdaXiabigdaXaqabaGccqGHsislcqWItecBdaWgaaWcbaGaeGimaaJaeGimaadabeaakiabcMcaPmaaqafabaGaeiikaGIaemiCaa3aaSbaaSqaaiabdQgaQjabdUgaRbqabaGccqGHsislcqWGlbWscqGGPaqkaSqaaiabdQgaQjabcYcaSiabdUgaRjabcQda6maaHaaabaGaemyta0eacaGLcmaadaWgaaadbaGaemOAaOMaem4AaSgabeaaliabg2da9iabigdaXaqab0GaeyyeIuoaaaa@54BE@

where

*K *= (*ℓ*_01 _- *ℓ*_00_)/(*ℓ*_10 _- *ℓ*_11 _- *ℓ*_00_)

and *p*_*jk *_= *P*(*M*_*jk *_= 1|*x*, *y*) is the posterior probability that (*j*, *k*) is a match, which is estimated by the empirical frequency of this match from an MCMC run.

Thus M^
 MathType@MTEF@5@5@+=feaafiart1ev1aaatCvAUfKttLearuWrP9MDH5MBPbIqV92AaeXatLxBI9gBaebbnrfifHhDYfgasaacH8akY=wiFfYdH8Gipec8Eeeu0xXdbba9frFj0=OqFfea0dXdd9vqai=hGuQ8kuc9pgc9s8qqaq=dirpe0xb9q8qiLsFr0=vr0=vr0dc8meaabaqaciaacaGaaeqabaqabeGadaaakeaadaqiaaqaaiabd2eanbGaayPadaaaaa@2E91@ is a solution to a "linear assignment" problem with cost matrix (*p*_*jk *_- *K*). A standard linear assignment program (lpsolve [34]) is used to find M^
 MathType@MTEF@5@5@+=feaafiart1ev1aaatCvAUfKttLearuWrP9MDH5MBPbIqV92AaeXatLxBI9gBaebbnrfifHhDYfgasaacH8akY=wiFfYdH8Gipec8Eeeu0xXdbba9frFj0=OqFfea0dXdd9vqai=hGuQ8kuc9pgc9s8qqaq=dirpe0xb9q8qiLsFr0=vr0=vr0dc8meaabaqaciaacaGaaeqabaqabeGadaaakeaadaqiaaqaaiabd2eanbGaayPadaaaaa@2E91@ with the cost matrix (*p*_*jk *_- *K*)_+ _and we take ℓ_11 _= ℓ_00 _= 0 (there is no cost for declaring true matches and also identifying true non-matching amino acids). We penalise (avoid) false positives more than false negatives i.e. we take K=ℓ01ℓ01+ℓ10=0.75
 MathType@MTEF@5@5@+=feaafiart1ev1aaatCvAUfKttLearuWrP9MDH5MBPbIqV92AaeXatLxBI9gBaebbnrfifHhDYfgasaacH8akY=wiFfYdH8Gipec8Eeeu0xXdbba9frFj0=OqFfea0dXdd9vqai=hGuQ8kuc9pgc9s8qqaq=dirpe0xb9q8qiLsFr0=vr0=vr0dc8meaabaqaciaacaGaaeqabaqabeGadaaakeaacqWGlbWscqGH9aqpdaWcaaqaaiabloriSnaaBaaaleaacqaIWaamcqaIXaqmaeqaaaGcbaGaeS4eHW2aaSbaaSqaaiabicdaWiabigdaXaqabaGccqGHRaWkcqWItecBdaWgaaWcbaGaeGymaeJaeGimaadabeaaaaGccqGH9aqpcqaIWaamcqGGUaGlcqaI3aWncqaI1aqnaaa@3E5E@

We note that although arguably point estimates are most important to a molecular biologist, by maintaining detailed balance and drawing from the posterior of all parameters MCMC provides a way to escape local maxima for the model. Furthermore, it provides an easy framework for quantifying uncertainty in parameters and alternative solutions. Besides the MCMC approach goes further to give statistical understanding of the distribution of matches, transformation, correspondence parameters and the loss function.

### Accounting for amino acid groups

The matching indicator *M*_*jk *_is constrained to be zero, in the probability model and all algorithmic steps, for amino acids *j*, *k *in different amino acid groups. This strictly matches amino acids in the same group only. This way, unlike in [16] and similar to graph theoretic method, we do not utilise a probability distribution for *κ *in Equation 7.

### MCMC refinement step

**Figure 8:**
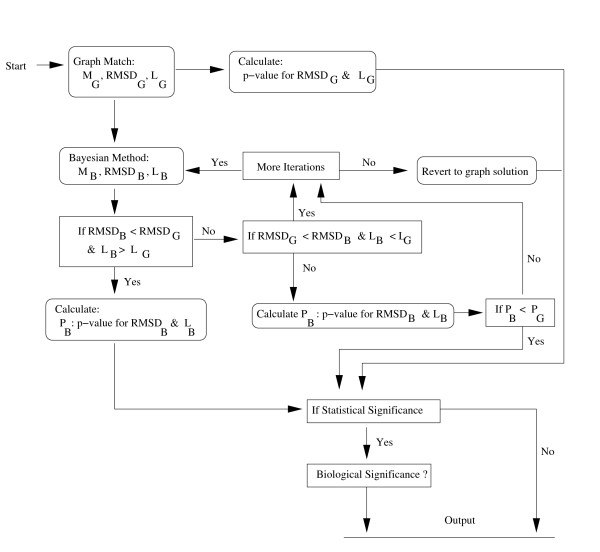
Decision tree for refining the graph solution by the MCMC method. Boxes with curved corners show processes and their output while boxes with sharp corners are for branching conditions. The procedure starts with graph solution *M*_*G*_. The graph solution's RMSD and number of matches are denoted by *RMSD*_*G *_and *L*_*G *_respectively. MCMC is re-iterated until the MCMC solution: *M*_*B *_is better. The RMSD and number of matches for *M*_*B *_are denoted by *RMSD*_*B *_and *L*_*B *_respectively. *M*_*B *_and *M*_*G *_are compared using 1) RMSDs and the number of matches or 2) P-values for *M*_*G *_and *M*_*G*_, denoted by *P*_*G *_and *P*_*B *_respectively.

We now describe a hierarchical decision rule to assess the improvement from graph method. Figure 8 shows the refinement decision tree. Arrows show flow directions, boxes with curved corners show processes and their output while boxes with sharp corners are for branching conditions. The procedure starts with graph matching to obtain the solution, *M*_*G *_and the corresponding RMSD and the number of matches: *RMSD*_*G *_and *L*_*G *_respectively. We use MCMC sampling in the Bayesian modelling starting from the rotation and translation obtained from the graph solution *M*_*G*_. We iteratively start from *β *= 1.5, 0.9, 0.5 (for the noise parameter *σ*^-2 ^~ Γ(*α*, *β*)) and *κ *= 0.00005, 0.0001, 0.0005, 0.005. We find the MCMC solution *M*_*B *_with the RMSD value say, *RMSD*_*B *_and the number of matches *L*_*B*_. We monitor convergence and quality of the solution in terms of RMSD, statistical significance and the number of matched amino acids. The MCMC solution *M*_*B *_is primarily assessed against the graph solution *M*_*G *_using plugged-in RMSD (defined below) and the number of matches. We stop if MCMC solution is better than the graph method or we have exhausted all the combinations of *β *and *κ *values.

Note that *M*_*B *_is clearly better than *M*_*G *_if *RMSD*_*B *_<*RMSD*_*G *_and *L*_*B *_> *L*_*G *_while *M*_*G *_is clearly better than *M*_*B *_if *RMSD*_*B *_> *RMSD*_*G *_and *L*_*B *_<*L*_*G*_. If these conditions do not hold (when the plugged-in RMSD for MCMC is smaller than the graph method but on the other hand the graph solution has the bigger number of matches or vice versa), we use p-values for *M*_*B *_and *M*_*G *_using Equations 1 and 2. We denote the p-values for *M*_*B *_and *M*_*G *_by *P*_*B *_and *P*_*G *_respectively. *M*_*B *_is better that *M*_*G *_if *P*_*B *_<*P*_*G*_.

In rare cases after all the steps, if the MCMC solution is not better then we re-start the whole decision tree. Note that this situation is very rare, for example, in all the significant matches reported in Table 1, only one match by MCMC drifted into a poor solution than the graph method. A second MCMC run on this particular matching converged to the same solution as the graph method. Most likely the first run for this matching might not have converged. We observe that a re-run of the MCMC might be necessary in those rare occurrences where the MCMC gives a poor solution. From our experiments, we find that in most situations, a second run would suffice for the non-converging cases and thus our MCMC procedure is robust.

Once we have an improved solution from MCMC and find that its p-value is statistically significant (whereas the graph solution was not significant), we examine the match for chemical properties.

By plugged-in RMSD we mean using point estimates of the transformation parameters *A *and *τ *obtained from the Bayesian model to calculate the RMSD for the aligned matching amino acids. In the decision tree we use the average plugged-in RMSD for *C*_*α *_and *C*_*β *_atoms:

RMSD2=(∑j,k:Mjk=1||x1j−Ay1k−τ||2+∑j′,k′:Mj′k′=1||x2j′−Ay2k′−τ||2)/(L+L0)
 MathType@MTEF@5@5@+=feaafiart1ev1aaatCvAUfKttLearuWrP9MDH5MBPbIqV92AaeXatLxBI9gBaebbnrfifHhDYfgasaacH8akY=wiFfYdH8Gipec8Eeeu0xXdbba9frFj0=OqFfea0dXdd9vqai=hGuQ8kuc9pgc9s8qqaq=dirpe0xb9q8qiLsFr0=vr0=vr0dc8meaabaqaciaacaGaaeqabaqabeGadaaakeaacqWGsbGucqWGnbqtcqWGtbWucqWGebardaahaaWcbeqaaiabikdaYaaakiabg2da9iabcIcaOmaaqafabaGaeiiFaWNaeiiFaWNaemiEaG3aaSbaaSqaaiabigdaXiabdQgaQbqabaGccqGHsislcqWGbbqqcqWG5bqEdaWgaaWcbaGaeGymaeJaem4AaSgabeaakiabgkHiTGGaciab=r8a0jabcYha8jabcYha8naaCaaaleqabaGaeGOmaidaaaqaaiabdQgaQjabcYcaSiabdUgaRjabcQda6iabd2eannaaBaaameaacqWGQbGAcqWGRbWAaeqaaSGaeyypa0JaeGymaedabeqdcqGHris5aOGaey4kaSYaaabuaeaacqGG8baFcqGG8baFcqWG4baEdaWgaaWcbaGaeGOmaiJafmOAaOMbauaaaeqaaOGaeyOeI0IaemyqaeKaemyEaK3aaSbaaSqaaiabikdaYiqbdUgaRzaafaaabeaakiabgkHiTiab=r8a0jabcYha8jabcYha8naaCaaaleqabaGaeGOmaidaaOGaeiykaKIaei4la8IaeiikaGIaemitaWKaey4kaSIaemitaW0aaSbaaSqaaiabicdaWaqabaGccqGGPaqkaSqaaiqbdQgaQzaafaGaeiilaWIafm4AaSMbauaacqGG6aGocqWGnbqtdaWgaaadbaGafmOAaOMbauaacuWGRbWAgaqbaaqabaWccqGH9aqpcqaIXaqmaeqaniabggHiLdaaaa@7E50@

where *C*_*β *_atoms are matched as well for the *j'th *and *k'th *amino acids. *L *= ∑_*j*, *k *_*M*_*jk *_is the number of matches and *L*_0 _= ∑_*j'*, *k' *_*M*_*j' k' *_is the number of matches with two atoms matched for both the *j'th *and *k'th *amino acids.

#### Hyper-parameters

We now want to give values of the hyper-parameters and fixed parameters used in this paper. For prior distributions for *σ*^-2 ^~ Γ(*α*, *β*), *τ *~ *N*(*μ*_*τ*_, στ2
 MathType@MTEF@5@5@+=feaafiart1ev1aaatCvAUfKttLearuWrP9MDH5MBPbIqV92AaeXatLxBI9gBaebbnrfifHhDYfgasaacH8akY=wiFfYdH8Gipec8Eeeu0xXdbba9frFj0=OqFfea0dXdd9vqai=hGuQ8kuc9pgc9s8qqaq=dirpe0xb9q8qiLsFr0=vr0=vr0dc8meaabaqaciaacaGaaeqabaqabeGadaaakeaaiiGacqWFdpWCdaqhaaWcbaGae8hXdqhabaGaeGOmaidaaaaa@3155@ we used *α *= 1, *β *iteratively took values 1.5,0.9,0.5 (see above: MCMC refinement step),μτ=∑jx1j/m−∑ky1k/n, στ=15
 MathType@MTEF@5@5@+=feaafiart1ev1aaatCvAUfKttLearuWrP9MDH5MBPbIqV92AaeXatLxBI9gBaebbnrfifHhDYfgasaacH8akY=wiFfYdH8Gipec8Eeeu0xXdbba9frFj0=OqFfea0dXdd9vqai=hGuQ8kuc9pgc9s8qqaq=dirpe0xb9q8qiLsFr0=vr0=vr0dc8meaabaqaciaacaGaaeqabaqabeGadaaakeaaiiGacqWF8oqBdaWgaaWcbaGae8hXdqhabeaakiabg2da9maaqafabaGaemiEaG3aaSbaaSqaaiabigdaXiabdQgaQbqabaGccqGGVaWlcqWGTbqBaSqaaiabdQgaQbqab0GaeyyeIuoakiabgkHiTmaaqafabaGaemyEaK3aaSbaaSqaaiabigdaXiabdUgaRbqabaGccqGGVaWlcqWGUbGBcqGGSaalcqqGGaaicqWFdpWCdaWgaaWcbaGae8hXdqhabeaakiabg2da9iabigdaXiabiwda1aWcbaGaem4AaSgabeqdcqGHris5aaaa@4E37@. We used *γ *= 0.5 and this value is found to work well in case of all considered functional sites in SITESDB. The cost ratio for declaring "false" matches is taken to be K=ℓ01ℓ01+ℓ10=0.75
 MathType@MTEF@5@5@+=feaafiart1ev1aaatCvAUfKttLearuWrP9MDH5MBPbIqV92AaeXatLxBI9gBaebbnrfifHhDYfgasaacH8akY=wiFfYdH8Gipec8Eeeu0xXdbba9frFj0=OqFfea0dXdd9vqai=hGuQ8kuc9pgc9s8qqaq=dirpe0xb9q8qiLsFr0=vr0=vr0dc8meaabaqaciaacaGaaeqabaqabeGadaaakeaacqWGlbWscqGH9aqpdaWcaaqaaiabloriSnaaBaaaleaacqaIWaamcqaIXaqmaeqaaaGcbaGaeS4eHW2aaSbaaSqaaiabicdaWiabigdaXaqabaGccqGHRaWkcqWItecBdaWgaaWcbaGaeGymaeJaeGimaadabeaaaaGccqGH9aqpcqaIWaamcqGGUaGlcqaI3aWncqaI1aqnaaa@3E5E@.

### Sensitivity of hyper-parameters in MCMC

The method is not observed to be sensitive to hyper-parameters for *τ *and *σ *(also see [16]). Mean values for *τ *ranging from 0 to the difference in centres of mass for the sites have worked in many cases of matching functional sites in SITESDB. Weak priors e.g. standard deviation values for *τ *in the range of 5 to 50Å have worked very well. With shape parameter for the prior Gamma distribution for *σ*^-2 ^set to 1, scale parameter values in the range of 0.5–50 have given satisfactory results in many cases. For *κ*, values in the range of 0.0001 – 0.003 have been observed to give optimal results in many examples. In our implementation we track all parameters for MCMC. Histogram and trace plots for parameter values can be used to check MCMC convergence and model stability. In addition we have used RMSD as a complimentary diagnostic tool, namely if RMSD is relatively large then we continue iterations.

## Authors' contributions

KVM, PJG contributed to the formulation of the model and methodology. KVM and VBN contributed to the formulation to account for side chains. VBN, DRW, KVM contributed to the design (refinement step). PJG, NDG and VBN contributed to the design, development and implementation of software. DRW and NDG provided data. VBN performed the computations and statistical analysis. NDG performed the biological analysis. VBN, NDG, DRW and KVM drafted the manuscript. DRW conceived of the study. KVM, VBN and PJG revised the manuscript. All authors read and approved the final manuscript.

## Supplementary Material

Additional file 2 **Case 2 Results**. Results for 17 – *β *hydroxysteroid dehydrogenase and family. Tables 1–5: Without amino acid property. Tables 6–10: With amino acid property. Click here for file

Additional file 1 **Case 1 Results**. Results for alcohol dehydrogenase (1hdx_1) matching against its own SCOP family. Tables 1–2: Without amino acid property. Tables 3–4: With amino acid property Click here for file

Additional file 3 **Case 3 Results**. Results for alcohol dehydrogenase (1hdx_1) and superfamily. Tables 1–14: Without physico-chemistry. Tables 14–28: With physico-chemistry. Click here for file

Additional file 4 **Case 4 Results**. Results for alcohol dehydrogenase and FAD/NAD(P)-binding domain. Tables 1–5: Without physico-chemistry. Tables 5–10: With physico-chemistry. Click here for file
