# Supplementary material for: Bayesian refinement of protein functional site matching
Source: BMC Bioinformatics. 2007 Jul 17;8:257. doi: 10.1186/1471-2105-8-257 (PMC1940029; doi:10.1186/1471-2105-8-257)
Supplement: Additional file 2 — Case 2 Results. Results for 17 – β hydroxysteroid dehydrogenase and family. Tables 1–5: Without amino acid property. Tables 6–10: With amino acid property. [file 1471-2105-8-257-S2.pdf]

Results for $17 - \beta$ hydroxysteroid dehydrogenase and family.

Table 1: Results for $17 - \beta$ hydroxysteroid dehydrogenase matching against its own SCOP family without amino acid property.

Site	N	RMSD	q	Pvalue	Evalue	RMSD	q	Pvalue	Evalue	CG	Mean L	Var L	SCOP
1udb_0	38	0.872	10	9.89E-01	4.54E+00	0.893	13	2.35E-05	2.35E-05	10	15	1.4	c.2.1.2
1udc_0	58	0.878	10	1.00E+00	2.20E+01	1.118	17	1.05E-04	1.05E-04	8	18	2.2	c.2.1.2
1xel_0	57	0.867	11	1.80E-01	1.98E-01	0.949	16	3.03E-08	3.03E-08	9	19	4.7	c.2.1.2
1lrk_2	34	0.828	11	7.43E-03	7.46E-03	1.030	17	5.60E-08	5.60E-08	9	19	2.0	c.2.1.2
1nah_0	39	0.886	12	5.33E-03	5.35E-03	0.922	16	1.37E-09	1.37E-09	10	17	4.0	c.2.1.2
2udp_0	54	0.751	12	1.33E-06	1.33E-06	1.062	18	9.10E-08	9.10E-08	9	19	2.0	c.2.1.2
2udp_1	52	1.088	14	8.26E-01	1.75E+00	1.083	18	3.65E-07	3.65E-07	11	19	2.0	c.2.1.2
1uda_0	37	0.881	10	9.98E-01	6.39E+00	1.018	16	8.87E-07	8.87E-07	8	17	1.4	c.2.1.2
1a9y_0	53	1.058	17	3.83E-06	3.83E-06	1.084	18	4.16E-07	4.16E-07	11	19	2.1	c.2.1.2
1lrl_2	33	1.171	12	1.00E+00	9.39E+02	0.979	15	1.56E-06	1.56E-06	9	17	2.0	c.2.1.2
1kvu_0	58	0.828	10	9.51E-01	3.02E+00	1.094	18	1.11E-06	1.11E-06	7	19	2.0	c.2.1.2
1kvr_0	38	0.851	11	2.38E-02	2.41E-02	1.018	16	9.63E-07	9.63E-07	9	17	1.4	c.2.1.2
1a9z_0	33	0.842	11	9.48E-03	9.52E-03	1.029	16	1.28E-06	1.28E-06	9	17	1.4	c.2.1.2
1lrj_0	53	1.008	12	9.97E-01	5.75E+00	1.131	19	6.43E-07	6.43E-07	9	19	1.9	c.2.1.2
1nai_0	37	0.885	11	2.15E-01	2.42E-01	1.074	16	3.21E-05	3.21E-05	9	17	1.7	c.2.1.2
1kvt_0	52	0.879	10	1.00E+00	2.90E+01	1.031	17	2.22E-07	2.22E-07	9	19	6.0	c.2.1.2
1kvq_0	52	1.081	18	8.39E-07	8.39E-07	0.943	16	1.50E-08	1.50E-08	12	18	10.4	c.2.1.2
1kvs_0	52	0.839	10	9.78E-01	3.84E+00	1.117	18	3.93E-06	3.93E-06	7	19	2.2	c.2.1.2
1i3k_0	52	1.024	12	1.00E+00	2.89E+01	0.994	18	5.01E-10	5.01E-10	10	19	2.4	c.2.1.2
1i3k_1	52	1.375	10	1.00E+00	1.85E+09	0.989	18	3.40E-10	3.40E-10	8	19	1.9	c.2.1.2
1i3l_6	32	0.885	11	1.43E-01	1.55E-01	0.969	16	2.08E-08	2.08E-08	10	18	2.1	c.2.1.2
1i3l_7	33	0.865	10	9.72E-01	3.56E+00	0.968	16	2.13E-08	2.13E-08	7	17	6.5	c.2.1.2
1i3n_0	54	1.278	10	1.00E+00	3.71E+08	0.990	17	1.34E-08	1.34E-08	8	19	3.1	c.2.1.2
1i3n_6	33	0.752	10	8.13E-03	8.17E-03	0.971	16	2.62E-08	2.62E-08	7	18	2.1	c.2.1.2
1i3m_0	54	1.432	10	1.00E+00	1.12E+10	0.997	17	2.23E-08	2.23E-08	8	19	2.9	c.2.1.2
1i3m_6	32	0.747	10	5.54E-03	5.56E-03	1.012	17	1.30E-08	1.30E-08	9	18	2.2	c.2.1.2
1hzj_0	54	0.848	11	9.34E-02	9.80E-02	1.013	17	7.01E-08	7.01E-08	11	19	2.5	c.2.1.2
1hzj_1	55	1.035	11	1.00E+00	1.59E+03	0.979	19	5.38E-12	5.38E-12	10	20	1.8	c.2.1.2
1ek5_0	36	1.010	15	3.27E-05	3.27E-05	1.038	16	3.00E-06	3.00E-06	11	18	2.1	c.2.1.2
1gy8_0	120	0.795	14	5.54E-08	5.54E-08	0.980	26	0.00E+00	2.48E-21	13	28	3.3	c.2.1.2
1bxk_0	33	1.066	12	1.00E+00	6.08E+01	1.147	15	8.30E-02	8.66E-02	9	17	2.7	c.2.1.2
1bxk_1	36	0.871	13	9.30E-06	9.30E-06	0.871	13	9.30E-06	9.30E-06	13	17	2.0	c.2.1.2
1g1a_0	38	0.940	12	1.09E-01	1.15E-01	1.204	18	1.57E-03	1.57E-03	9	17	1.7	c.2.1.2
1g1a_1	37	0.985	12	7.23E-01	1.28E+00	1.136	17	2.44E-04	2.44E-04	9	17	1.6	c.2.1.2
1g1a_2	37	1.067	14	1.65E-01	1.80E-01	1.203	18	1.35E-03	1.36E-03	12	17	1.6	c.2.1.2
1g1a_3	37	0.991	12	8.31E-01	1.78E+00	1.193	18	7.01E-04	7.02E-04	10	17	1.6	c.2.1.2
1kep_0	45	0.907	12	2.85E-02	2.89E-02	0.836	11	1.12E-02	1.12E-02	8	17	3.9	c.2.1.2
1kep_1	58	1.156	16	5.19E-02	5.33E-02	1.110	17	1.75E-04	1.75E-04	12	17	1.5	c.2.1.2
1ket_2	38	1.090	11	1.00E+00	6.23E+03	1.126	16	2.37E-03	2.38E-03	9	16	1.6	c.2.1.2
1ket_3	39	1.190	15	7.84E-01	1.53E+00	1.123	16	2.14E-03	2.14E-03	11	16	1.4	c.2.1.2
1kew_2	39	1.090	13	1.00E+00	1.65E+01	1.080	17	6.83E-06	6.83E-06	10	17	1.6	c.2.1.2
1kew_3	38	1.074	13	9.98E-01	6.43E+00	0.775	10	2.49E-02	2.52E-02	7	16	9.6	c.2.1.2
1ker_0	59	1.040	14	2.45E-02	2.48E-02	1.005	16	3.59E-06	3.59E-06	12	18	2.0	c.2.1.2
1ker_1	61	1.299	18	9.31E-01	2.67E+00	1.086	16	8.35E-04	8.36E-04	12	17	2.7	c.2.1.2
1keu_0	58	0.953	12	5.84E-01	8.78E-01	1.150	18	1.54E-04	1.54E-04	11	17	1.8	c.2.1.2
1keu_1	57	0.952	12	5.45E-01	7.87E-01	1.157	18	2.36E-04	2.36E-04	11	17	1.6	c.2.1.2
1r6d_0	57	1.034	13	9.13E-01	2.45E+00	1.067	18	3.97E-07	3.97E-07	11	18	1.5	c.2.1.2
1r66_1	38	1.092	11	1.00E+00	6.81E+03	1.063	13	4.16E-01	5.38E-01	11	16	1.6	c.2.1.2
1kc1_2	27	1.048	12	1.00E+00	1.32E+01	1.108	18	7.77E-07	7.77E-07	9	17	3.4	c.2.1.2
1kc3_0	39	1.099	14	7.45E-01	1.37E+00	1.050	17	8.52E-07	8.52E-07	11	18	2.0	c.2.1.2
1e6u_0	120	0.850	17	7.04E-12	7.04E-12	0.850	17	7.04E-12	7.04E-12	17	30	5.0	c.2.1.2
1e7s_0	120	0.892	18	5.04E-12	5.04E-12	1.068	24	1.27E-14	1.26E-14	16	26	3.9	c.2.1.2
1e7q_0	120	0.964	16	1.75E-06	1.75E-06	1.175	26	1.67E-12	1.67E-12	14	28	4.7	c.2.1.2
1e7r_0	120	0.988	16	9.56E-06	9.56E-06	1.234	27	4.54E-11	4.54E-11	14	28	4.5	c.2.1.2
1bsv_0	29	0.935	10	1.00E+00	7.00E+01	0.993	15	2.53E-06	2.53E-06	8	18	2.5	c.2.1.2
1fxs_0	29	1.006	11	1.00E+00	5.59E+01	1.046	16	2.57E-06	2.57E-06	10	18	2.0	c.2.1.2
1bws_0	29	0.809	11	9.99E-04	9.99E-04	0.809	11	9.99E-04	9.99E-04	11	17	2.0	c.2.1.2
1rpn_0	40	0.893	14	1.66E-06	1.66E-06	1.029	18	7.69E-09	7.69E-09	11	19	1.8	c.2.1.2
1rpn_1	40	0.820	13	1.13E-06	1.13E-06	1.016	18	2.82E-09	2.82E-09	11	18	1.8	c.2.1.2
1rpn_2	39	0.821	12	9.23E-05	9.23E-05	1.029	18	7.11E-09	7.11E-09	10	18	9.7	c.2.1.2
1rpn_3	39	0.894	13	1.60E-04	1.60E-04	1.026	18	5.65E-09	5.65E-09	10	18	1.7	c.2.1.2
1t2a_0	40	0.919	13	8.63E-04	8.64E-04	1.063	17	2.29E-06	2.29E-06	10	17	3.0	c.2.1.2
1t2a_2	39	0.923	17	3.20E-11	3.20E-11	0.923	17	3.20E-11	3.20E-11	17	18	1.9	c.2.1.2
1t2a_4	39	0.930	18	1.24E-12	1.24E-12	0.930	18	1.24E-12	1.24E-12	18	18	1.6	c.2.1.2
1t2a_6	39	0.849	12	3.37E-04	3.37E-04	1.060	18	7.39E-08	7.39E-08	10	18	1.5	c.2.1.2
1n7h_0	35	0.853	13	7.47E-06	7.47E-06	1.064	20	5.33E-11	5.33E-11	10	20	1.8	c.2.1.2

Table 2: Results for $17-\beta$ hydroxysteroid dehydrogenase matching against its own SCOP family without amino acid property.

Site	N	RMSD	q	Pvalue	Evalue	RMSD	q	Pvalue	Evalue	CG	Mean L	Var L	SCOP
1n7h_2	35	0.865	14	1.57E-07	1.57E-07	1.065	20	5.79E-11	5.79E-11	11	20	1.9	c.2.1.2
1n7g_0	58	0.858	15	1.14E-08	1.14E-08	1.031	19	4.36E-10	4.36E-10	11	20	2.3	c.2.1.2
1n7g_1	57	0.876	14	1.52E-06	1.52E-06	1.065	20	2.59E-10	2.59E-10	11	21	2.7	c.2.1.2
1n7g_2	58	0.875	13	1.55E-04	1.55E-04	1.075	20	6.13E-10	6.13E-10	10	21	2.4	c.2.1.2
1n7g_3	37	0.792	13	1.18E-07	1.18E-07	1.043	20	1.12E-11	1.12E-11	11	20	1.9	c.2.1.2
1eq2_0	36	0.936	10	1.00E+00	1.43E+02	1.031	15	1.22E-04	1.22E-04	9	17	2.1	c.2.1.2
1eq2_1	54	1.108	10	1.00E+00	7.56E+05	1.032	15	4.52E-04	4.52E-04	8	15	1.2	c.2.1.2
1eq2_2	36	0.766	15	6.62E-13	6.62E-13	0.766	15	6.62E-13	6.62E-13	15	17	2.0	c.2.1.2
1eq2_3	54	1.161	19	5.79E-06	5.79E-06	1.161	19	5.79E-06	5.79E-06	19	17	2.5	c.2.1.2
1eq2_4	36	0.994	18	1.62E-10	1.62E-10	0.994	18	1.62E-10	1.62E-10	18	16	1.7	c.2.1.2
1eq2_5	36	1.109	10	1.00E+00	2.26E+05	0.874	14	1.86E-07	1.86E-07	8	15	3.4	c.2.1.2
1eq2_6	36	0.885	12	1.25E-03	1.25E-03	1.018	15	5.43E-05	5.43E-05	11	17	2.2	c.2.1.2
1eq2_7	36	1.100	10	1.00E+00	1.59E+05	1.020	15	6.16E-05	6.16E-05	8	16	1.7	c.2.1.2
1eq2_8	36	1.076	10	1.00E+00	6.09E+04	1.019	15	5.78E-05	5.78E-05	8	17	2.1	c.2.1.2
1eq2_9	36	1.098	11	1.00E+00	2.75E+03	1.026	15	8.96E-05	8.96E-05	8	16	1.8	c.2.1.2
1orr_0	35	0.774	12	1.55E-06	1.55E-06	0.774	12	1.55E-06	1.55E-06	12	17	3.6	c.2.1.2
1orr_2	34	1.026	11	1.00E+00	2.37E+02	1.315	19	3.64E-02	3.71E-02	8	17	1.5	c.2.1.2
1orr_4	34	1.016	11	1.00E+00	1.48E+02	1.320	19	4.94E-02	5.06E-02	8	18	1.6	c.2.1.2
1orr_6	35	0.879	10	1.00E+00	8.58E+00	1.304	18	1.57E-01	1.71E-01	7	17	1.7	c.2.1.2
1i24_0	58	1.160	10	1.00E+00	2.26E+06	1.317	21	2.21E-04	2.21E-04	7	20	2.7	c.2.1.2
1qrr_0	58	1.159	10	1.00E+00	2.18E+06	1.213	19	2.33E-05	2.33E-05	7	20	3.4	c.2.1.2
1i2c_0	58	1.143	10	1.00E+00	1.22E+06	1.238	20	1.06E-05	1.06E-05	7	21	2.9	c.2.1.2
1i2b_0	59	1.132	10	1.00E+00	8.65E+05	1.248	20	2.16E-05	2.16E-05	7	21	3.3	c.2.1.2
1k6x_0	27	1.964	10	1.00E+00	5.55E+15	2.021	11	1.00E+00	2.15E+15	10	11	0.3	c.2.1.2
1ti7_0	33	0.694	12	7.12E-09	7.12E-09	0.710	13	1.25E-10	1.25E-10	12	15	1.2	c.2.1.2
1cyd_0	40	0.784	16	7.66E-14	7.66E-14	0.849	24	0.00E+00	1.09E-26	16	24	1.6	c.2.1.2
1cyd_1	40	0.737	19	0.00E+00	2.52E-22	0.831	25	0.00E+00	1.45E-29	18	25	1.6	c.2.1.2
1cyd_2	40	0.771	17	2.22E-16	2.02E-16	0.849	24	0.00E+00	1.09E-26	17	24	1.5	c.2.1.2
1cyd_3	40	0.885	26	0.00E+00	1.60E-28	0.854	24	0.00E+00	2.04E-26	22	24	1.7	c.2.1.2
1pr9_0	41	0.698	21	0.00E+00	8.51E-29	0.775	26	0.00E+00	6.06E-35	21	25	1.1	c.2.1.2
1pr9_1	42	0.808	30	0.00E+00	9.15E-41	0.745	27	0.00E+00	5.01E-39	25	26	0.8	c.2.1.2
1oaa_0	39	0.914	23	0.00E+00	1.25E-21	0.914	23	0.00E+00	1.25E-21	23	25	2.5	c.2.1.2
1sep_0	43	0.884	25	0.00E+00	1.77E-26	0.884	25	0.00E+00	1.77E-26	25	28	2.5	c.2.1.2
1nas_0	46	0.946	24	0.00E+00	1.68E-21	0.946	24	0.00E+00	1.68E-21	24	27	38.2	c.2.1.2
1dhr_0	31	0.890	9	1.00E+00	5.57E+02	1.234	16	5.73E-02	5.91E-02	8	17	2.4	c.2.1.2
1dir_0	33	0.891	10	1.00E+00	1.29E+01	1.231	17	5.49E-03	5.50E-03	10	17	2.0	c.2.1.2
1dir_1	36	0.930	9	1.00E+00	4.80E+03	1.009	14	1.71E-04	1.71E-04	8	17	7.4	c.2.1.2
1dir_2	33	0.947	9	1.00E+00	7.37E+03	1.162	15	2.10E-02	2.13E-02	9	17	2.1	c.2.1.2
1dir_3	32	1.622	11	1.00E+00	3.84E+09	1.012	12	1.12E-01	1.19E-01	0	17	5.8	c.2.1.2
1hdr_0	30	2.803	8	1.00E+00	4.58E+18	1.187	14	5.31E-01	7.56E-01	0	16	2.3	c.2.1.2
1e7w_0	44	0.979	13	4.49E-02	4.60E-02	0.953	15	7.14E-07	7.14E-07	8	21	3.2	c.2.1.2
1e7w_1	43	1.278	17	1.00E+00	8.68E+00	1.092	17	7.73E-06	7.73E-06	11	21	3.2	c.2.1.2
1e92_0	38	1.040	17	3.87E-07	3.87E-07	0.737	14	7.47E-12	7.47E-12	12	19	3.0	c.2.1.2
1e92_1	38	0.781	14	2.05E-10	2.05E-10	0.733	14	5.47E-12	5.47E-12	12	19	3.0	c.2.1.2
1e92_2	37	0.930	12	5.84E-02	6.01E-02	0.738	14	7.44E-12	7.44E-12	9	19	2.9	c.2.1.2
1e92_3	38	1.041	13	6.48E-01	1.04E+00	0.950	17	2.35E-10	2.35E-10	10	21	3.1	c.2.1.2
1p33_0	42	1.125	14	9.27E-01	2.62E+00	0.875	15	3.09E-09	3.09E-09	11	21	3.5	c.2.1.2
1p33_1	42	1.130	13	1.00E+00	1.69E+02	0.798	14	9.56E-10	9.56E-10	9	21	3.3	c.2.1.2
1p33_2	42	0.767	13	2.70E-08	2.70E-08	0.894	16	2.18E-10	2.18E-10	7	21	4.0	c.2.1.2
1p33_3	42	0.713	11	1.04E-05	1.04E-05	0.876	14	1.97E-07	1.97E-07	6	20	4.2	c.2.1.2
1mxh_0	42	0.934	12	5.49E-02	5.65E-02	1.031	17	1.15E-07	1.15E-07	9	21	3.5	c.2.1.2
1mxh_1	42	1.007	12	9.98E-01	6.16E+00	1.107	19	5.45E-08	5.45E-08	7	21	2.9	c.2.1.2
1mxh_2	42	1.102	13	1.00E+00	1.42E+01	0.989	16	1.89E-07	1.89E-07	9	21	3.5	c.2.1.2
1mxh_3	42	1.198	17	1.82E-02	1.83E-02	0.896	15	1.35E-08	1.35E-08	12	21	3.1	c.2.1.2
1mxf_0	47	1.111	12	1.00E+00	1.64E+03	0.968	17	1.74E-09	1.74E-09	7	19	3.1	c.2.1.2
1mxf_1	47	1.035	12	1.00E+00	3.75E+01	1.051	18	2.67E-08	2.67E-08	9	21	3.7	c.2.1.2
1mxf_2	46	0.840	11	3.72E-02	3.79E-02	0.604	10	2.96E-06	2.96E-06	6	19	13.4	c.2.1.2
1mxf_3	46	0.860	12	1.08E-03	1.08E-03	0.953	17	5.31E-10	5.31E-10	9	20	3.6	c.2.1.2
1qyw_2	20	0.310	19	0.00E+00	3.32E-54	0.310	19	0.00E+00	3.32E-54	19	19	0.1	c.2.1.2
1qyx_2	19	0.334	18	0.00E+00	8.67E-48	0.334	18	0.00E+00	8.67E-48	18	18	0.0	c.2.1.2
1qyv_0	33	0.412	31	0.00E+00	7.52E-83	0.412	31	0.00E+00	7.52E-83	31	31	0.4	c.2.1.2
1a27_0	63	0.000	63	0.00E+00	0.00E+00	0.000	63	0.00E+00	0.00E+00	63	63	0.0	c.2.1.2
1fdt_0	50	0.517	34	0.00E+00	5.25E-79	0.504	41	0.00E+00	8.03E-100	34	41	0.3	c.2.1.2
1fdu_0	60	0.405	49	0.00E+00	2.63E-147	0.380	57	0.00E+00	5.79E-178	48	58	0.4	c.2.1.2
1fdu_1	49	0.446	35	0.00E+00	1.25E-92	0.485	44	0.00E+00	5.19E-112	35	44	0.5	c.2.1.2
1fdu_2	45	0.403	37	0.00E+00	5.91E-107	0.406	44	0.00E+00	2.44E-127	37	44	0.5	c.2.1.2

Table 3: Results for $17-\beta$ hydroxysteroid dehydrogenase matching against its own SCOP family without amino acid property.

Site	N	RMSD	q	Pvalue	Evalue	RMSD	q	Pvalue	Evalue	CG	Mean L	Var L	SCOP
1fdu_3	47	0.437	35	0.00E+00	3.68E-94	0.434	42	0.00E+00	5.11E-115	35	42	0.3	c.2.1.2
1equ_0	43	0.531	31	0.00E+00	8.60E-69	0.558	40	0.00E+00	3.16E-89	31	40	0.3	c.2.1.2
1equ_1	33	0.517	20	0.00E+00	6.49E-39	0.545	28	0.00E+00	4.40E-58	20	29	0.4	c.2.1.2
1fdv_0	52	0.438	41	0.00E+00	2.07E-113	0.461	51	0.00E+00	1.11E-137	41	51	0.3	c.2.1.2
1fdv_1	34	0.463	31	0.00E+00	6.54E-77	0.464	34	0.00E+00	3.00E-85	31	34	0.2	c.2.1.2
1fdv_3	32	0.434	23	0.00E+00	7.76E-56	0.575	30	0.00E+00	8.35E-61	23	30	0.3	c.2.1.2
1fdv_5	30	0.482	23	0.00E+00	5.02E-51	0.593	29	0.00E+00	1.63E-56	23	29	0.4	c.2.1.2
1fmc_0	47	0.707	17	0.00E+00	3.42E-19	1.014	26	0.00E+00	2.75E-22	13	26	1.7	c.2.1.2
1fmc_1	47	0.768	19	0.00E+00	1.89E-20	0.989	26	0.00E+00	1.75E-23	15	27	1.7	c.2.1.2
1ahi_0	48	0.651	15	0.00E+00	4.97E-17	1.075	26	0.00E+00	1.84E-19	13	26	1.5	c.2.1.2
1ahi_1	46	0.854	16	6.05E-11	6.05E-11	1.084	27	0.00E+00	1.95E-20	12	27	1.9	c.2.1.2
1ahh_0	32	0.718	15	6.22E-15	6.23E-15	0.718	15	6.22E-15	6.23E-15	15	20	1.0	c.2.1.2
1ahh_1	29	0.706	14	4.00E-13	4.00E-13	0.706	14	4.00E-13	4.00E-13	14	19	1.1	c.2.1.2
2hsd_0	35	0.796	17	1.11E-15	1.12E-15	1.046	23	1.11E-16	1.29E-16	13	22	1.3	c.2.1.2
2hsd_1	32	0.801	16	1.84E-13	1.84E-13	1.027	21	1.21E-14	1.21E-14	12	21	1.5	c.2.1.2
2hsd_2	29	0.742	16	5.55E-16	5.09E-16	0.943	20	2.22E-16	2.08E-16	12	19	0.9	c.2.1.2
2hsd_3	32	0.814	17	3.11E-15	3.10E-15	0.814	17	3.11E-15	3.10E-15	17	19	1.2	c.2.1.2
1fk8_0	30	1.006	24	0.00E+00	5.96E-20	0.726	21	0.00E+00	6.21E-28	21	22	0.8	c.2.1.2
1fk8_1	28	1.538	14	1.00E+00	8.05E+08	0.688	18	0.00E+00	3.37E-23	11	18	0.7	c.2.1.2
1nff_0	35	0.803	23	0.00E+00	3.76E-27	0.601	22	0.00E+00	1.68E-37	17	23	1.1	c.2.1.2
1nff_1	35	0.764	26	0.00E+00	4.04E-36	0.729	24	0.00E+00	4.51E-34	22	24	0.8	c.2.1.2
1nfr_0	43	0.716	18	0.00E+00	4.92E-21	0.727	24	0.00E+00	6.42E-34	18	24	0.3	c.2.1.2
1nfr_1	44	0.742	18	0.00E+00	1.01E-19	0.752	23	0.00E+00	2.78E-30	16	23	0.9	c.2.1.2
1nfr_2	43	0.710	18	0.00E+00	2.45E-21	0.733	23	0.00E+00	2.13E-31	16	23	0.7	c.2.1.2
1nfr_3	43	0.629	19	0.00E+00	6.07E-28	0.805	24	0.00E+00	2.20E-29	18	23	0.6	c.2.1.2
1bdb_0	54	0.727	25	0.00E+00	2.13E-35	0.780	32	0.00E+00	8.29E-47	21	32	1.2	c.2.1.2
1b14_0	50	0.755	27	0.00E+00	9.28E-38	0.870	32	0.00E+00	4.83E-40	23	33	2.6	c.2.1.2
1b14_1	35	0.673	18	0.00E+00	1.54E-23	0.815	21	0.00E+00	3.80E-23	14	22	1.2	c.2.1.2
1gee_0	32	0.719	17	0.00E+00	4.40E-19	0.711	22	0.00E+00	7.31E-31	16	22	1.1	c.2.1.2
1gee_1	32	0.720	19	0.00E+00	1.24E-23	0.732	21	0.00E+00	1.56E-27	18	21	1.4	c.2.1.2
1gee_2	31	0.754	15	2.52E-13	2.52E-13	0.774	21	0.00E+00	1.88E-25	13	21	1.4	c.2.1.2
1gee_3	33	0.852	25	0.00E+00	2.89E-29	0.704	21	0.00E+00	5.62E-29	20	23	1.8	c.2.1.2
1gco_0	33	0.729	18	0.00E+00	9.66E-21	0.727	23	0.00E+00	4.23E-32	16	23	1.2	c.2.1.2
1gco_1	33	0.726	21	0.00E+00	1.19E-27	0.738	23	0.00E+00	1.83E-31	19	23	1.1	c.2.1.2
1gco_2	33	0.811	18	1.11E-16	6.62E-17	0.757	23	0.00E+00	2.18E-30	14	23	1.3	c.2.1.2
1gco_3	34	0.857	26	0.00E+00	1.48E-30	0.873	26	0.00E+00	1.18E-29	25	25	1.3	c.2.1.2
1g6k_0	31	0.814	15	4.61E-11	4.61E-11	0.745	21	0.00E+00	6.61E-27	13	21	1.0	c.2.1.2
1g6k_1	31	0.790	18	0.00E+00	1.55E-18	0.768	21	0.00E+00	9.50E-26	17	21	1.1	c.2.1.2
1g6k_2	30	0.880	14	4.89E-07	4.89E-07	0.801	20	0.00E+00	3.17E-22	12	21	1.1	c.2.1.2
1g6k_3	32	0.971	24	0.00E+00	1.93E-21	0.831	22	0.00E+00	1.36E-24	20	23	1.4	c.2.1.2
1rwb_0	34	0.666	17	0.00E+00	1.36E-21	0.737	24	0.00E+00	1.26E-33	16	24	0.8	c.2.1.2
1rwb_1	32	0.756	21	0.00E+00	4.42E-26	0.889	24	0.00E+00	2.30E-25	19	24	1.4	c.2.1.2
1rwb_2	32	0.729	15	2.80E-14	2.80E-14	0.762	22	0.00E+00	4.46E-28	14	22	1.0	c.2.1.2
1rwb_3	33	0.785	18	0.00E+00	3.19E-18	0.757	22	0.00E+00	2.67E-28	17	23	1.6	c.2.1.2
1geg_0	38	0.651	19	0.00E+00	4.92E-27	0.757	25	0.00E+00	2.26E-34	17	26	1.1	c.2.1.2
1geg_1	40	0.590	21	0.00E+00	5.86E-36	0.714	27	0.00E+00	1.87E-41	18	28	0.9	c.2.1.2
1geg_2	38	0.732	29	0.00E+00	1.42E-44	0.732	29	0.00E+00	1.42E-44	29	27	1.0	c.2.1.2
1geg_3	39	0.819	30	0.00E+00	2.93E-40	0.819	30	0.00E+00	2.93E-40	30	27	1.0	c.2.1.2
1geg_4	40	0.741	30	0.00E+00	5.80E-46	0.729	27	0.00E+00	2.14E-40	25	27	1.1	c.2.1.2
1geg_5	39	0.748	31	0.00E+00	1.13E-47	0.748	31	0.00E+00	1.13E-47	31	27	1.1	c.2.1.2
1geg_6	40	0.640	22	0.00E+00	5.91E-35	0.726	27	0.00E+00	1.32E-40	19	28	1.2	c.2.1.2
1geg_7	39	0.641	21	0.00E+00	1.78E-32	0.728	26	0.00E+00	2.49E-38	18	27	0.9	c.2.1.2
1iy8_0	41	0.815	16	1.40E-12	1.40E-12	0.989	26	0.00E+00	1.15E-23	12	25	1.7	c.2.1.2
1iy8_1	40	0.774	25	0.00E+00	2.12E-33	0.774	25	0.00E+00	2.12E-33	25	24	1.8	c.2.1.2
1iy8_2	40	0.719	16	1.11E-16	1.38E-16	0.954	24	0.00E+00	6.31E-22	12	25	1.7	c.2.1.2
1iy8_3	40	0.781	25	0.00E+00	5.46E-33	1.004	25	0.00E+00	3.89E-21	20	25	1.8	c.2.1.2
1iy8_4	41	0.725	17	0.00E+00	1.81E-18	0.952	25	0.00E+00	6.77E-24	14	25	1.8	c.2.1.2
1iy8_5	40	0.860	18	1.54E-14	1.55E-14	1.025	25	0.00E+00	3.59E-20	13	24	1.7	c.2.1.2
1iy8_6	40	0.680	16	0.00E+00	2.36E-18	0.964	24	0.00E+00	1.84E-21	13	24	1.8	c.2.1.2
1iy8_7	40	1.017	19	5.88E-10	5.88E-10	1.005	25	0.00E+00	4.33E-21	13	24	1.7	c.2.1.2
1h5q_0	88	0.907	21	1.11E-16	1.12E-16	0.911	33	0.00E+00	3.80E-38	20	34	3.2	c.2.1.2
1h5q_2	95	0.701	22	0.00E+00	9.41E-30	0.956	35	0.00E+00	3.80E-38	20	36	2.7	c.2.1.2
1h5q_4	89	1.251	29	7.27E-14	7.27E-14	0.927	35	0.00E+00	2.50E-40	26	34	2.2	c.2.1.2
1h5q_5	91	0.964	31	0.00E+00	4.91E-31	0.829	33	0.00E+00	4.07E-44	27	34	2.4	c.2.1.2
1h5q_6	116	1.009	22	2.90E-13	2.90E-13	0.893	35	0.00E+00	1.60E-42	21	36	3.3	c.2.1.2
1h5q_7	117	1.050	22	1.77E-11	1.77E-11	0.842	34	0.00E+00	1.18E-44	21	36	2.8	c.2.1.2

Table 4: Results for $17-\beta$ hydroxysteroid dehydrogenase matching against its own SCOP family without amino acid property.

Site	N	RMSD	q	Pvalue	Evalue	RMSD	q	Pvalue	Evalue	CG	Mean L	Var L	SCOP
1h5q_9	91	1.050	22	8.26E-12	8.26E-12	0.905	35	0.00E+00	6.21E-42	20	35	2.3	c.2.1.2
1h5q_10	91	1.007	23	3.77E-15	3.79E-15	0.859	33	0.00E+00	7.52E-42	22	34	2.3	c.2.1.2
1h5q_11	92	1.020	34	0.00E+00	1.05E-32	0.867	33	0.00E+00	3.03E-41	27	34	2.3	c.2.1.2
1h5q_13	90	0.899	34	0.00E+00	3.30E-41	0.825	33	0.00E+00	1.94E-44	29	34	2.7	c.2.1.2
1edo_0	35	0.780	25	0.00E+00	4.36E-33	0.767	23	0.00E+00	9.41E-30	23	23	1.2	c.2.1.2
1q7c_0	19	1.249	15	9.73E-01	3.61E+00	1.030	15	1.56E-05	1.56E-05	11	14	1.3	c.2.1.2
1q7c_1	20	0.733	9	7.73E-02	8.04E-02	0.413	7	8.12E-04	8.12E-04	4	13	12.7	c.2.1.2
1q7b_8	30	0.856	22	0.00E+00	7.94E-23	0.909	24	0.00E+00	3.44E-24	20	24	1.1	c.2.1.2
1q7b_9	30	1.098	23	6.79E-14	6.79E-14	0.966	24	0.00E+00	1.97E-21	19	23	1.1	c.2.1.2
1q7b_10	30	0.757	16	2.44E-15	2.44E-15	0.925	23	0.00E+00	9.68E-22	13	23	1.3	c.2.1.2
1q7b_11	29	0.803	16	1.63E-13	1.63E-13	0.970	24	0.00E+00	2.73E-21	13	23	1.0	c.2.1.2
1o5i_0	29	0.970	13	7.38E-03	7.41E-03	1.152	19	4.51E-07	4.51E-07	11	20	1.6	c.2.1.2
1eno_0	30	0.894	19	2.22E-16	2.73E-16	0.789	17	1.11E-16	1.27E-16	15	18	10.8	c.2.1.2
1d7o_0	32	0.776	17	0.00E+00	4.85E-17	0.776	17	0.00E+00	4.85E-17	17	20	1.2	c.2.1.2
1cwu_0	40	0.870	20	0.00E+00	6.21E-18	1.071	25	0.00E+00	4.01E-18	19	25	1.8	c.2.1.2
1cwu_1	40	0.774	17	2.22E-16	2.02E-16	1.092	25	0.00E+00	3.22E-17	15	25	1.6	c.2.1.2
1nhd_0	34	0.774	16	1.82E-14	1.82E-14	0.866	20	0.00E+00	5.05E-19	14	21	1.8	c.2.1.2
1nhd_1	34	0.780	15	3.37E-12	3.37E-12	0.907	20	0.00E+00	2.54E-17	13	21	2.0	c.2.1.2
1nhg_0	38	0.752	15	3.94E-13	3.94E-13	0.883	20	0.00E+00	3.69E-18	13	22	7.8	c.2.1.2
1nhg_1	39	0.779	15	4.71E-12	4.71E-12	1.079	22	2.10E-13	2.10E-13	13	23	2.3	c.2.1.2
1nhw_0	37	0.807	15	3.00E-11	3.00E-11	0.892	20	0.00E+00	8.03E-18	13	22	2.5	c.2.1.2
1nhw_1	37	0.789	14	9.51E-10	9.51E-10	0.902	20	0.00E+00	2.06E-17	12	22	2.3	c.2.1.2
1nnu_0	38	0.809	16	4.36E-13	4.36E-13	0.968	21	2.22E-16	2.45E-16	14	22	13.9	c.2.1.2
1nnu_1	41	0.771	15	2.72E-12	2.72E-12	0.964	21	2.22E-16	2.13E-16	13	23	2.7	c.2.1.2
1uh5_0	38	0.782	15	5.65E-12	5.65E-12	0.910	20	0.00E+00	4.74E-17	13	21	1.7	c.2.1.2
1uh5_1	38	0.792	15	1.34E-11	1.34E-11	0.909	20	0.00E+00	4.32E-17	13	22	2.5	c.2.1.2
1eny_0	28	1.006	18	9.83E-10	9.83E-10	0.879	16	2.00E-11	2.00E-11	12	16	9.3	c.2.1.2
1p44_0	39	0.828	12	1.45E-04	1.45E-04	0.935	18	1.88E-12	1.88E-12	8	19	1.6	c.2.1.2
1p44_1	43	0.880	13	8.64E-05	8.64E-05	0.994	18	2.80E-10	2.80E-10	10	20	2.1	c.2.1.2
1p44_2	41	0.881	12	4.60E-03	4.61E-03	1.029	18	3.46E-09	3.46E-09	9	19	1.6	c.2.1.2
1p44_3	44	0.869	14	7.21E-07	7.21E-07	0.892	17	3.97E-12	3.97E-12	10	18	1.5	c.2.1.2
1p44_4	29	0.820	14	5.09E-09	5.09E-09	0.890	17	9.33E-13	9.33E-13	11	18	0.9	c.2.1.2
1p44_5	28	0.896	11	1.69E-01	1.85E-01	0.983	16	3.59E-08	3.59E-08	8	17	1.2	c.2.1.2
1enz_0	28	0.780	16	9.21E-15	9.20E-15	0.780	16	9.21E-15	9.20E-15	16	18	1.1	c.2.1.2
1p45_0	43	0.906	15	9.97E-08	9.97E-08	1.020	18	2.04E-09	2.04E-09	11	19	1.9	c.2.1.2
1p45_1	34	0.842	15	6.14E-10	6.14E-10	1.049	20	1.42E-11	1.42E-11	10	20	1.3	c.2.1.2
1bvr_0	39	0.817	12	7.11E-05	7.11E-05	0.936	17	8.75E-11	8.75E-11	9	18	1.2	c.2.1.2
1bvr_1	39	0.820	12	8.65E-05	8.65E-05	0.941	17	1.28E-10	1.28E-10	9	18	1.1	c.2.1.2
1bvr_2	39	0.826	12	1.28E-04	1.28E-04	0.939	17	1.10E-10	1.10E-10	9	18	1.2	c.2.1.2
1bvr_3	39	0.823	12	1.05E-04	1.05E-04	0.938	17	1.02E-10	1.02E-10	9	18	1.1	c.2.1.2
1bvr_4	28	0.807	11	1.18E-03	1.18E-03	0.916	16	3.17E-10	3.17E-10	9	17	7.5	c.2.1.2
1bvr_5	30	0.799	12	3.18E-06	3.18E-06	1.128	18	1.53E-06	1.53E-06	11	18	1.3	c.2.1.2
1qsg_0	37	0.812	13	5.04E-07	5.04E-07	1.181	21	3.94E-08	3.94E-08	11	22	2.2	c.2.1.2
1qsg_1	36	0.810	12	3.51E-05	3.51E-05	1.147	21	2.49E-09	2.49E-09	10	23	2.0	c.2.1.2
1qsg_2	36	1.137	18	1.45E-05	1.45E-05	1.263	23	1.77E-07	1.77E-07	13	23	2.2	c.2.1.2
1qsg_3	36	0.964	18	3.23E-11	3.23E-11	0.964	18	3.23E-11	3.23E-11	18	23	2.0	c.2.1.2
1qsg_4	37	0.799	11	1.73E-03	1.73E-03	1.126	20	8.60E-09	8.60E-09	9	23	2.5	c.2.1.2
1qsg_5	36	0.803	12	2.21E-05	2.21E-05	1.174	21	2.10E-08	2.10E-08	10	23	2.2	c.2.1.2
1qsg_6	36	0.855	13	5.64E-06	5.64E-06	1.140	21	1.42E-09	1.42E-09	11	22	2.1	c.2.1.2
1qsg_7	36	0.788	11	8.12E-04	8.12E-04	1.149	21	2.92E-09	2.92E-09	9	23	2.0	c.2.1.2
1qg6_0	37	0.848	14	5.54E-08	5.54E-08	1.131	21	7.48E-10	7.48E-10	12	23	2.1	c.2.1.2
1qg6_1	37	0.920	16	3.70E-09	3.70E-09	1.131	21	7.48E-10	7.48E-10	12	23	2.2	c.2.1.2
1qg6_2	37	0.780	13	4.86E-08	4.86E-08	1.131	21	7.48E-10	7.48E-10	11	23	2.1	c.2.1.2
1qg6_3	37	0.780	13	4.86E-08	4.86E-08	1.131	21	7.48E-10	7.48E-10	11	23	2.2	c.2.1.2
1dfi_0	28	0.860	12	1.40E-04	1.40E-04	1.011	17	8.03E-09	8.03E-09	11	18	1.6	c.2.1.2
1dfi_1	27	1.078	15	2.29E-03	2.30E-03	1.022	17	1.56E-08	1.56E-08	10	18	1.4	c.2.1.2
1dfi_2	26	0.903	10	9.97E-01	5.94E+00	1.064	17	2.51E-07	2.51E-07	8	17	1.3	c.2.1.2
1dfi_3	29	0.837	11	7.65E-03	7.68E-03	0.923	16	5.88E-10	5.88E-10	8	17	15.9	c.2.1.2
1c14_0	38	0.797	15	1.43E-11	1.43E-11	0.797	15	1.43E-11	1.43E-11	15	22	2.1	c.2.1.2
1c14_1	38	0.953	20	4.88E-15	4.87E-15	0.953	20	4.88E-15	4.87E-15	20	22	2.5	c.2.1.2
1dfh_0	34	0.847	14	2.44E-08	2.44E-08	0.896	18	4.65E-14	4.66E-14	12	18	15.5	c.2.1.2
1dfh_1	33	0.900	19	2.11E-15	2.11E-15	0.900	19	2.11E-15	2.11E-15	19	19	1.1	c.2.1.2
1dfg_0	34	0.866	14	9.06E-08	9.06E-08	0.977	19	1.04E-12	1.04E-12	12	19	1.2	c.2.1.2
1dfg_1	38	0.898	20	0.00E+00	2.81E-17	0.898	20	0.00E+00	2.81E-17	20	20	1.4	c.2.1.2
1mfp_0	43	0.815	15	9.15E-11	9.15E-11	0.815	15	9.15E-11	9.15E-11	15	23	2.1	c.2.1.2
1mfp_1	42	0.951	22	0.00E+00	3.51E-18	0.951	22	0.00E+00	3.51E-18	22	23	1.8	c.2.1.2

Table 5: Results for $17-\beta$ hydroxysteroid dehydrogenase matching against its own SCOP family without amino acid property.

Site	N	RMSD	q	Pvalue	Evalue	RMSD	q	Pvalue	Evalue	CG	Mean L	Var L	SCOP
1d8a_0	35	0.828	12	6.69E-05	6.69E-05	1.126	21	4.20E-10	4.20E-10	11	21	11.2	c.2.1.2
1d8a_1	36	0.918	20	3.33E-16	3.07E-16	0.918	20	3.33E-16	3.07E-16	20	23	1.9	c.2.1.2
1lx6_0	32	0.916	12	1.70E-02	1.71E-02	1.170	20	1.52E-07	1.52E-07	10	20	1.4	c.2.1.2
1lx6_1	33	0.917	19	1.01E-14	1.01E-14	0.917	19	1.01E-14	1.01E-14	19	19	1.8	c.2.1.2
1lxc_0	39	0.976	16	2.89E-07	2.89E-07	0.917	18	4.22E-13	4.22E-13	11	20	19.8	c.2.1.2
1lxc_1	38	1.076	19	3.92E-09	3.92E-09	1.093	20	7.08E-10	7.08E-10	12	21	2.2	c.2.1.2
1i30_0	31	0.697	14	1.99E-13	1.99E-13	0.697	14	1.99E-13	1.99E-13	14	21	1.5	c.2.1.2
1i30_1	31	0.938	21	0.00E+00	1.54E-17	0.938	21	0.00E+00	1.54E-17	21	20	1.6	c.2.1.2
1i2z_0	39	0.884	16	2.56E-10	2.56E-10	0.944	20	2.33E-15	2.32E-15	14	22	1.8	c.2.1.2
1i2z_1	38	0.932	20	6.66E-16	7.06E-16	0.932	20	6.66E-16	7.06E-16	20	22	1.7	c.2.1.2
1jw7_0	36	0.816	15	5.74E-11	5.74E-11	0.816	15	5.74E-11	5.74E-11	15	21	2.5	c.2.1.2
1jw7_1	37	0.785	13	7.04E-08	7.04E-08	1.115	20	1.03E-08	1.03E-08	12	21	2.5	c.2.1.2
1jw7_2	37	0.783	13	6.07E-08	6.07E-08	1.018	19	9.13E-11	9.13E-11	12	21	2.6	c.2.1.2
1jw7_3	37	0.784	13	6.54E-08	6.54E-08	1.019	19	9.91E-11	9.91E-11	12	20	2.3	c.2.1.2
1jvf_0	35	0.860	13	7.17E-06	7.17E-06	1.007	19	3.10E-11	3.10E-11	13	21	2.6	c.2.1.2
1jvf_1	36	0.818	12	5.93E-05	5.93E-05	1.008	19	3.67E-11	3.67E-11	12	21	2.7	c.2.1.2
1jvf_2	36	0.819	12	6.33E-05	6.33E-05	1.008	19	3.67E-11	3.67E-11	12	21	2.6	c.2.1.2
1jvf_3	35	0.816	12	4.77E-05	4.77E-05	0.782	14	2.38E-10	2.38E-10	10	19	23.3	c.2.1.2
1ae1_0	33	0.847	17	1.70E-13	1.70E-13	0.978	23	0.00E+00	1.54E-19	14	24	2.2	c.2.1.2
1ae1_1	35	0.943	18	2.12E-11	2.12E-11	1.066	27	0.00E+00	3.14E-21	17	27	2.9	c.2.1.2
2ae2_0	43	0.918	19	9.14E-14	9.14E-14	1.144	26	3.33E-16	3.73E-16	14	26	2.9	c.2.1.2
2ae2_1	44	0.805	17	5.33E-15	5.34E-15	1.068	25	0.00E+00	3.97E-18	11	26	2.7	c.2.1.2
1ipe_0	35	0.871	18	2.94E-14	2.94E-14	1.049	23	2.22E-16	1.71E-16	13	24	2.0	c.2.1.2
1ipe_1	37	0.939	19	4.19E-13	4.19E-13	1.011	25	0.00E+00	6.46E-21	14	25	1.7	c.2.1.2
1ipf_0	45	0.878	20	0.00E+00	3.58E-17	1.026	26	0.00E+00	2.10E-21	15	28	3.8	c.2.1.2
1ipf_1	45	0.937	19	6.35E-13	6.35E-13	1.113	27	0.00E+00	1.07E-18	13	27	2.7	c.2.1.2
1g0o_0	42	0.675	19	0.00E+00	1.61E-25	0.836	25	0.00E+00	8.26E-30	17	24	0.8	c.2.1.2
1g0o_1	42	0.753	22	0.00E+00	9.36E-28	0.834	25	0.00E+00	6.42E-30	18	24	0.8	c.2.1.2
1g0o_2	43	0.728	31	0.00E+00	3.27E-49	0.728	31	0.00E+00	3.27E-49	31	25	0.8	c.2.1.2
1g0o_3	43	0.667	20	0.00E+00	2.66E-28	0.830	26	0.00E+00	5.36E-32	18	25	0.8	c.2.1.2
1g0n_0	43	0.632	20	0.00E+00	1.80E-30	0.771	25	0.00E+00	1.76E-33	17	25	0.9	c.2.1.2
1g0n_1	35	0.658	20	0.00E+00	4.00E-29	0.756	25	0.00E+00	1.17E-34	18	25	1.0	c.2.1.2
1doh_0	43	0.685	21	0.00E+00	1.57E-29	0.790	25	0.00E+00	2.28E-32	18	25	0.8	c.2.1.2
1doh_1	42	0.680	20	0.00E+00	1.48E-27	0.786	24	0.00E+00	1.26E-30	17	24	0.8	c.2.1.2
1ybv_0	113	0.693	28	0.00E+00	1.08E-43	0.865	35	0.00E+00	3.49E-45	25	36	2.0	c.2.1.2
1ybv_1	113	0.669	32	0.00E+00	3.75E-55	0.865	35	0.00E+00	3.49E-45	29	36	2.6	c.2.1.2
1ja9_0	43	0.707	17	0.00E+00	2.95E-19	0.792	23	0.00E+00	4.05E-28	15	24	1.3	c.2.1.2
1hdo_0	109	1.031	13	1.00E+00	1.48E+01	1.347	29	2.74E-08	2.74E-08	10	29	2.9	c.2.1.2
1he2_0	120	1.097	12	1.00E+00	1.44E+04	1.097	12	1.00E+00	1.44E+04	12	14	1.6	c.2.1.2
1he3_0	120	1.137	11	1.00E+00	1.60E+06	1.377	30	1.03E-07	1.03E-07	8	31	3.6	c.2.1.2
1he4_0	115	1.080	12	1.00E+00	5.51E+03	1.372	28	2.34E-06	2.34E-06	7	29	3.9	c.2.1.2
1he5_0	114	1.078	12	1.00E+00	4.86E+03	1.386	29	1.32E-06	1.32E-06	7	29	3.7	c.2.1.2
1e6w_0	111	0.898	39	0.00E+00	1.31E-49	0.898	39	0.00E+00	1.31E-49	39	40	3.5	c.2.1.2
1e6w_1	49	0.840	25	0.00E+00	3.67E-28	0.897	34	0.00E+00	1.24E-41	21	36	2.4	c.2.1.2
1e6w_2	51	0.932	29	0.00E+00	5.75E-30	0.973	36	0.00E+00	2.55E-39	25	37	2.4	c.2.1.2
1e6w_3	111	0.966	33	0.00E+00	1.88E-33	0.882	35	0.00E+00	2.01E-43	26	37	4.1	c.2.1.2
1e3s_0	47	0.792	24	0.00E+00	3.16E-29	0.777	30	0.00E+00	4.99E-43	24	33	2.2	c.2.1.2
1e3s_1	47	0.788	24	0.00E+00	1.79E-29	0.937	34	0.00E+00	8.18E-39	21	34	29.4	c.2.1.2
1e3s_2	46	0.789	23	0.00E+00	1.92E-27	0.999	36	0.00E+00	1.32E-37	20	35	2.2	c.2.1.2
1e3s_3	47	0.820	25	0.00E+00	1.91E-29	0.967	35	0.00E+00	2.67E-38	22	35	2.0	c.2.1.2
1e3w_0	49	0.646	23	0.00E+00	1.07E-36	0.913	34	0.00E+00	1.81E-40	21	36	2.4	c.2.1.2
1e3w_1	49	0.730	25	0.00E+00	2.57E-35	0.887	34	0.00E+00	2.26E-42	23	36	2.3	c.2.1.2
1e3w_2	53	0.741	25	0.00E+00	1.89E-34	0.897	34	0.00E+00	1.58E-41	23	36	2.5	c.2.1.2
1e3w_3	55	0.726	24	0.00E+00	2.88E-33	0.871	34	0.00E+00	2.03E-43	22	37	2.2	c.2.1.2
1n5d_1	33	0.719	18	0.00E+00	3.08E-21	0.901	24	0.00E+00	3.38E-24	15	24	1.7	c.2.1.2
1sb8_0	55	0.962	16	6.24E-07	6.24E-07	0.952	18	4.43E-11	4.43E-11	14	19	1.9	c.2.1.2
1sb9_0	53	0.932	17	1.26E-09	1.26E-09	0.930	18	6.25E-12	6.25E-12	15	19	1.9	c.2.1.2
1vl8_2	37	1.018	15	3.26E-04	3.26E-04	1.011	21	2.11E-15	2.07E-15	15	24	2.1	c.2.1.2
1vl8_3	37	1.005	15	1.36E-04	1.36E-04	1.171	28	0.00E+00	5.12E-18	13	27	2.1	c.2.1.2
1sny_1	40	0.796	17	2.44E-15	2.43E-15	0.874	21	0.00E+00	3.02E-20	14	22	2.4	c.2.1.2
1xhl_0	43	0.773	18	0.00E+00	2.81E-18	0.915	27	0.00E+00	9.92E-29	16	27	1.4	c.2.1.2
1xhl_1	43	0.726	18	0.00E+00	1.55E-20	0.833	26	0.00E+00	1.25E-31	16	27	1.5	c.2.1.2

Table 6: Results for alcohol dehydrogenase (1hdx_1) matching against its own SCOP family with amino acid property.

Site	N	RMSD	q	Pvalue	Evalue	RMSD	q	Pvalue	Evalue	CG	Mean L	Var L	SCOP
1udb_0	38	0.955	8	1.00E+00	1.34E+02	1.018	12	5.88E-05	5.88E-05	8	14	1.1	c.2.1.2
1udc_0	58	0.944	8	1.00E+00	3.33E+02	0.865	13	3.75E-10	3.75E-10	6	14	4.8	c.2.1.2
1xel_0	57	0.933	9	8.78E-01	2.10E+00	0.919	15	3.99E-13	3.99E-13	7	15	3.4	c.2.1.2
1lrk_2	34	0.897	10	7.69E-04	7.69E-04	0.856	14	2.03E-13	2.03E-13	8	16	1.7	c.2.1.2
1nah_0	39	0.790	10	3.53E-06	3.53E-06	0.890	15	1.71E-14	1.70E-14	10	15	0.9	c.2.1.2
2udp_0	54	0.911	10	6.22E-03	6.24E-03	0.876	15	1.73E-14	1.73E-14	8	16	0.9	c.2.1.2
2udp_1	52	0.936	11	1.37E-04	1.37E-04	0.889	15	3.84E-14	3.84E-14	10	15	0.8	c.2.1.2
1uda_0	37	1.619	8	1.00E+00	8.06E+09	0.927	14	1.90E-11	1.90E-11	7	15	1.2	c.2.1.2
1a9y_0	53	1.000	11	3.59E-03	3.60E-03	0.895	13	1.67E-09	1.67E-09	10	13	1.1	c.2.1.2
1lrl_2	33	0.904	9	1.10E-01	1.16E-01	0.834	13	7.38E-12	7.38E-12	7	14	1.8	c.2.1.2
1kvu_0	58	2.267	8	1.00E+00	4.55E+15	0.894	14	1.25E-11	1.25E-11	6	14	1.0	c.2.1.2
1kvr_0	38	0.973	8	1.00E+00	3.32E+02	0.912	13	1.21E-09	1.21E-09	6	13	0.9	c.2.1.2
1a9z_0	33	1.005	7	1.00E+00	7.87E+04	0.890	12	3.70E-08	3.70E-08	5	12	1.9	c.2.1.2
1lrj_0	53	0.926	10	1.19E-02	1.20E-02	0.680	11	5.38E-10	5.38E-10	7	14	6.6	c.2.1.2
1nai_0	37	0.761	9	3.83E-04	3.83E-04	1.004	14	1.81E-09	1.81E-09	9	15	1.5	c.2.1.2
1kvt_0	52	0.871	8	1.00E+00	2.11E+01	0.922	14	5.23E-11	5.23E-11	7	14	1.1	c.2.1.2
1kvq_0	52	0.985	12	1.65E-05	1.65E-05	0.929	14	8.06E-11	8.06E-11	11	15	1.4	c.2.1.2
1kvs_0	52	1.565	8	1.00E+00	1.30E+10	0.963	15	5.50E-12	5.50E-12	7	15	1.0	c.2.1.2
1i3k_0	52	1.361	10	1.00E+00	2.73E+05	0.893	15	8.97E-14	8.97E-14	9	15	1.2	c.2.1.2
1i3k_1	52	1.479	10	1.00E+00	5.71E+06	0.890	15	7.27E-14	7.28E-14	9	15	0.9	c.2.1.2
1i3l_6	32	1.473	10	1.00E+00	1.07E+06	0.882	14	1.25E-12	1.25E-12	9	14	1.1	c.2.1.2
1i3l_7	33	1.535	9	1.00E+00	6.16E+07	0.881	14	1.28E-12	1.28E-12	8	14	0.9	c.2.1.2
1i3n_0	54	1.545	9	1.00E+00	3.60E+08	0.888	14	9.18E-12	9.18E-12	8	14	1.5	c.2.1.2
1i3n_6	33	1.408	9	1.00E+00	3.92E+06	0.888	14	2.02E-12	2.02E-12	8	14	1.2	c.2.1.2
1i3m_0	54	1.532	9	1.00E+00	2.60E+08	0.886	14	8.07E-12	8.07E-12	8	14	0.9	c.2.1.2
1i3m_6	32	1.409	9	1.00E+00	3.66E+06	0.905	14	5.43E-12	5.43E-12	8	14	1.3	c.2.1.2
1hzj_0	54	1.457	11	1.00E+00	2.25E+05	0.907	14	3.08E-11	3.08E-11	10	14	1.3	c.2.1.2
1hzj_1	55	1.456	11	1.00E+00	3.04E+05	0.863	15	1.28E-14	1.28E-14	10	16	0.9	c.2.1.2
1ek5_0	36	1.442	11	1.00E+00	2.56E+04	0.841	12	4.46E-09	4.46E-09	10	13	1.7	c.2.1.2
1gy8_0	120	1.046	9	1.00E+00	5.28E+02	1.089	21	0.00E+00	4.86E-18	8	21	2.0	c.2.1.2
1bxk_0	33	0.760	7	1.00E+00	1.12E+01	0.760	7	1.00E+00	1.12E+01	7	10	4.6	c.2.1.2
1bxk_1	36	0.787	8	2.45E-01	2.81E-01	0.897	10	8.33E-04	8.33E-04	8	11	1.1	c.2.1.2
1g1a_0	38	0.831	7	1.00E+00	2.23E+02	0.928	10	3.75E-03	3.76E-03	7	11	2.4	c.2.1.2
1g1a_1	37	0.820	7	1.00E+00	1.40E+02	0.919	10	2.35E-03	2.36E-03	7	11	1.4	c.2.1.2
1g1a_2	37	0.904	8	1.00E+00	1.10E+01	0.921	10	2.56E-03	2.57E-03	8	10	1.0	c.2.1.2
1g1a_3	37	0.831	7	1.00E+00	2.06E+02	0.771	8	3.05E-01	3.64E-01	6	10	3.5	c.2.1.2
1kep_0	45	0.732	8	5.52E-02	5.68E-02	0.948	10	1.45E-02	1.46E-02	8	11	1.3	c.2.1.2
1kep_1	58	1.423	11	1.00E+00	3.43E+04	0.777	9	5.50E-03	5.52E-03	8	11	1.8	c.2.1.2
1ket_2	38	0.890	8	1.00E+00	2.15E+01	0.676	8	1.08E-02	1.08E-02	7	10	2.9	c.2.1.2
1ket_3	39	1.174	10	1.00E+00	5.88E+01	0.923	10	4.39E-03	4.40E-03	9	11	1.7	c.2.1.2
1kew_2	39	1.573	8	1.00E+00	1.53E+09	0.959	10	1.48E-02	1.49E-02	3	11	1.6	c.2.1.2
1kew_3	38	1.683	8	1.00E+00	1.54E+10	0.721	8	5.30E-02	5.45E-02	5	10	2.8	c.2.1.2
1ker_0	59	1.079	8	1.00E+00	3.11E+04	1.066	10	9.98E-01	6.12E+00	8	11	0.5	c.2.1.2
1ker_1	61	1.510	10	1.00E+00	1.04E+07	1.485	11	1.00E+00	2.37E+05	10	12	0.5	c.2.1.2
1keu_0	58	1.559	8	1.00E+00	3.82E+09	1.657	9	1.00E+00	1.85E+09	8	9	0.4	c.2.1.2
1keu_1	57	0.854	7	1.00E+00	1.69E+03	0.937	10	1.89E-02	1.91E-02	7	11	1.9	c.2.1.2
1r6d_0	57	2.053	7	1.00E+00	1.82E+14	2.053	7	1.00E+00	1.82E+14	7	8	0.7	c.2.1.2
1r66_1	38	1.844	6	1.00E+00	2.58E+12	1.844	6	1.00E+00	2.58E+12	6	8	0.6	c.2.1.2
1kc1_2	27	0.918	9	1.07E-01	1.13E-01	1.038	14	6.44E-09	6.44E-09	9	13	0.8	c.2.1.2
1kc3_0	39	0.946	9	6.73E-01	1.12E+00	1.013	14	4.76E-09	4.76E-09	9	13	0.8	c.2.1.2
1e6u_0	120	1.494	12	1.00E+00	2.96E+05	1.088	17	6.04E-11	6.04E-11	11	19	3.3	c.2.1.2
1e7s_0	120	1.519	10	1.00E+00	1.81E+08	1.112	17	2.20E-10	2.20E-10	9	19	3.2	c.2.1.2
1e7q_0	120	1.506	12	1.00E+00	5.89E+05	1.003	17	1.74E-13	1.74E-13	11	18	2.3	c.2.1.2
1e7r_0	120	1.207	10	1.00E+00	1.75E+04	1.192	19	4.68E-11	4.68E-11	9	19	3.0	c.2.1.2
1bsv_0	29	1.208	7	1.00E+00	4.33E+06	0.869	10	2.93E-04	2.93E-04	6	11	1.5	c.2.1.2
1fxs_0	29	0.804	7	1.00E+00	3.76E+01	0.849	10	1.23E-04	1.23E-04	7	11	1.5	c.2.1.2
1bws_0	29	0.759	7	9.99E-01	7.22E+00	0.846	10	1.07E-04	1.07E-04	7	11	1.5	c.2.1.2
1rpn_0	40	0.849	7	1.00E+00	1.21E+03	0.941	10	1.01E-02	1.02E-02	6	10	1.3	c.2.1.2
1rpn_1	40	4.268	6	1.00E+00	1.01E+22	4.268	6	1.00E+00	1.01E+22	6	8	1.1	c.2.1.2
1rpn_2	39	1.934	6	1.00E+00	6.68E+13	1.934	6	1.00E+00	6.68E+13	6	7	0.5	c.2.1.2
1rpn_3	39	1.937	6	1.00E+00	6.93E+13	0.943	10	1.02E-02	1.02E-02	0	10	2.0	c.2.1.2
1t2a_0	40	1.482	6	1.00E+00	8.10E+10	1.482	6	1.00E+00	8.10E+10	6	6	0.4	c.2.1.2
1t2a_2	39	3.194	8	1.00E+00	3.27E+18	3.194	8	1.00E+00	3.27E+18	8	9	0.5	c.2.1.2
1t2a_4	39	2.440	8	1.00E+00	2.74E+15	2.440	8	1.00E+00	2.74E+15	8	8	0.4	c.2.1.2
1t2a_6	39	2.070	6	1.00E+00	3.34E+14	2.070	6	1.00E+00	3.34E+14	6	7	0.6	c.2.1.2
1n7h_0	35	0.821	7	1.00E+00	2.16E+02	0.901	10	3.58E-03	3.58E-03	7	12	1.2	c.2.1.2

Table 7: Results for alcohol dehydrogenase (1hdx_1) matching against its own SCOP family with amino acid property.

Site	N	RMSD	q	Pvalue	Evalue	RMSD	q	Pvalue	Evalue	CG	Mean L	Var L	SCOP
1n7h_2	35	1.082	7	1.00E+00	2.51E+05	0.986	12	7.54E-06	7.54E-06	5	13	1.3	c.2.1.2
1n7g_0	58	4.208	8	1.00E+00	2.64E+24	1.043	13	4.91E-06	4.91E-06	1	13	1.8	c.2.1.2
1n7g_1	57	0.689	7	1.00E+00	1.38E+01	1.038	13	3.62E-06	3.62E-06	6	14	1.9	c.2.1.2
1n7g_2	58	0.828	7	1.00E+00	7.36E+02	1.044	13	5.16E-06	5.16E-06	7	14	1.9	c.2.1.2
1n7g_3	37	0.686	7	9.62E-01	3.26E+00	1.014	13	2.84E-07	2.84E-07	6	13	1.0	c.2.1.2
1eq2_0	36	0.701	8	3.53E-03	3.54E-03	0.973	11	2.53E-04	2.53E-04	8	11	2.0	c.2.1.2
1eq2_1	54	0.710	8	1.87E-02	1.89E-02	0.981	11	1.26E-03	1.26E-03	8	11	0.7	c.2.1.2
1eq2_2	36	1.287	10	1.00E+00	3.65E+03	0.968	11	2.01E-04	2.01E-04	9	11	1.6	c.2.1.2
1eq2_3	54	1.546	11	1.00E+00	1.70E+06	0.585	8	2.55E-04	2.55E-04	7	11	4.1	c.2.1.2
1eq2_4	36	1.540	11	1.00E+00	4.08E+05	0.646	9	2.22E-06	2.22E-06	8	10	4.9	c.2.1.2
1eq2_5	36	0.706	8	4.48E-03	4.49E-03	0.981	11	3.64E-04	3.64E-04	8	11	0.9	c.2.1.2
1eq2_6	36	0.713	9	3.31E-05	3.31E-05	0.713	9	3.31E-05	3.31E-05	9	11	0.8	c.2.1.2
1eq2_7	36	0.699	8	3.21E-03	3.22E-03	0.723	10	3.03E-07	3.03E-07	8	10	3.9	c.2.1.2
1eq2_8	36	0.693	8	2.41E-03	2.41E-03	0.709	10	1.40E-07	1.40E-07	8	11	3.5	c.2.1.2
1eq2_9	36	0.702	8	3.71E-03	3.71E-03	0.975	11	2.77E-04	2.77E-04	8	11	0.5	c.2.1.2
1orr_0	35	0.693	7	8.19E-01	1.71E+00	0.985	14	6.86E-10	6.86E-10	7	14	3.7	c.2.1.2
1orr_2	34	0.804	8	4.06E-01	5.21E-01	1.005	14	1.98E-09	1.98E-09	7	15	1.2	c.2.1.2
1orr_4	34	0.719	7	9.89E-01	4.49E+00	0.991	14	8.88E-10	8.88E-10	7	15	1.2	c.2.1.2
1orr_6	35	0.725	7	9.98E-01	6.23E+00	1.093	15	3.20E-09	3.20E-09	7	15	1.4	c.2.1.2
1i24_0	58	0.823	7	1.00E+00	4.69E+02	1.150	14	3.16E-05	3.16E-05	6	16	3.5	c.2.1.2
1qrr_0	58	1.888	7	1.00E+00	2.55E+12	1.083	13	6.32E-05	6.32E-05	0	15	3.1	c.2.1.2
1i2c_0	58	1.896	7	1.00E+00	2.83E+12	1.020	12	3.03E-04	3.03E-04	0	14	3.4	c.2.1.2
1i2b_0	59	1.902	7	1.00E+00	3.22E+12	1.043	12	8.37E-04	8.37E-04	0	14	3.5	c.2.1.2
1k6x_0	27	1.609	7	1.00E+00	2.27E+10	1.633	8	1.00E+00	2.08E+09	7	8	0.3	c.2.1.2
1ti7_0	33	1.805	8	1.00E+00	1.21E+11	1.805	8	1.00E+00	1.21E+11	8	8	0.1	c.2.1.2
1cyd_0	40	0.591	13	0.00E+00	1.09E-20	0.650	17	0.00E+00	3.03E-28	13	17	0.6	c.2.1.2
1cyd_1	40	0.578	14	0.00E+00	2.79E-24	0.629	18	0.00E+00	4.04E-32	14	18	0.5	c.2.1.2
1cyd_2	40	0.595	13	0.00E+00	1.62E-20	0.641	17	0.00E+00	1.11E-28	13	17	0.7	c.2.1.2
1cyd_3	40	0.809	17	0.00E+00	7.52E-22	0.653	17	0.00E+00	4.23E-28	16	17	0.6	c.2.1.2
1pr9_0	41	0.665	16	0.00E+00	5.52E-26	0.593	18	0.00E+00	3.56E-34	15	18	0.6	c.2.1.2
1pr9_1	42	0.771	20	0.00E+00	8.20E-31	0.571	19	0.00E+00	1.39E-38	18	20	0.6	c.2.1.2
1oaa_0	39	0.799	14	6.77E-15	6.74E-15	1.145	21	0.00E+00	2.47E-17	14	21	1.7	c.2.1.2
1sep_0	43	0.922	15	1.42E-13	1.42E-13	1.167	22	0.00E+00	4.14E-18	15	21	1.8	c.2.1.2
1nas_0	46	1.290	15	5.72E-04	5.72E-04	1.123	21	0.00E+00	7.20E-18	13	21	1.7	c.2.1.2
1dhr_0	31	2.923	6	1.00E+00	5.86E+17	0.974	9	9.82E-01	4.02E+00	0	11	2.8	c.2.1.2
1dir_0	33	2.086	6	1.00E+00	2.32E+13	1.024	9	1.00E+00	2.25E+01	3	12	2.0	c.2.1.2
1dir_1	36	1.109	7	1.00E+00	3.31E+06	0.991	9	1.00E+00	1.08E+01	0	11	2.2	c.2.1.2
1dir_2	33	1.664	7	1.00E+00	2.65E+10	0.957	10	3.14E-02	3.19E-02	5	11	2.1	c.2.1.2
1dir_3	32	2.013	9	1.00E+00	6.62E+11	2.021	10	1.00E+00	8.50E+10	9	10	0.1	c.2.1.2
1hdr_0	30	3.698	6	1.00E+00	1.37E+18	3.698	6	1.00E+00	1.37E+18	6	7	0.8	c.2.1.2
1e7w_0	44	0.935	8	1.00E+00	1.04E+02	0.657	9	3.05E-05	3.05E-05	5	12	2.7	c.2.1.2
1e7w_1	43	1.059	9	1.00E+00	1.28E+02	1.047	12	1.82E-04	1.82E-04	6	13	2.0	c.2.1.2
1e92_0	38	3.785	8	1.00E+00	1.13E+21	0.947	11	1.79E-04	1.79E-04	1	13	1.8	c.2.1.2
1e92_1	38	1.022	8	1.00E+00	1.69E+03	0.947	11	1.79E-04	1.79E-04	6	12	1.9	c.2.1.2
1e92_2	37	0.964	7	1.00E+00	1.93E+04	0.949	11	1.80E-04	1.80E-04	5	12	1.8	c.2.1.2
1e92_3	38	0.990	9	1.00E+00	8.10E+00	1.003	13	2.30E-07	2.30E-07	8	13	2.8	c.2.1.2
1p33_0	42	0.917	8	1.00E+00	7.43E+01	1.160	13	6.20E-04	6.20E-04	8	13	1.7	c.2.1.2
1p33_1	42	0.866	8	9.99E-01	6.76E+00	1.008	12	2.81E-05	2.81E-05	8	13	1.4	c.2.1.2
1p33_2	42	0.972	9	9.87E-01	4.33E+00	0.689	9	1.24E-04	1.24E-04	6	12	7.1	c.2.1.2
1p33_3	42	2.756	7	1.00E+00	5.71E+17	2.817	8	1.00E+00	3.17E+17	7	9	0.8	c.2.1.2
1mxh_0	42	1.026	8	1.00E+00	3.35E+03	0.960	12	3.74E-06	3.74E-06	7	12	2.2	c.2.1.2
1mxh_1	42	1.330	8	1.00E+00	1.21E+07	0.965	12	4.78E-06	4.78E-06	6	12	2.1	c.2.1.2
1mxh_2	42	1.079	10	9.26E-01	2.61E+00	0.957	12	3.22E-06	3.22E-06	9	13	2.0	c.2.1.2
1mxh_3	42	0.941	10	1.02E-02	1.02E-02	0.739	11	5.07E-09	5.07E-09	9	12	2.2	c.2.1.2
1mxf_0	47	0.843	8	9.94E-01	5.18E+00	0.974	12	1.05E-05	1.05E-05	7	12	2.0	c.2.1.2
1mxf_1	47	1.367	8	1.00E+00	4.30E+07	0.981	12	1.47E-05	1.47E-05	6	13	2.2	c.2.1.2
1mxf_2	46	0.780	8	3.01E-01	3.58E-01	0.724	11	2.81E-09	2.81E-09	7	12	2.0	c.2.1.2
1mxf_3	46	0.796	8	5.07E-01	7.07E-01	0.951	12	3.17E-06	3.17E-06	7	13	2.0	c.2.1.2
1qyw_2	20	0.317	18	0.00E+00	1.52E-56	0.310	19	0.00E+00	1.18E-61	18	19	0.1	c.2.1.2
1qyx_2	19	0.342	17	0.00E+00	1.11E-49	0.334	18	0.00E+00	1.26E-54	17	18	0.1	c.2.1.2
1qyv_0	33	0.414	30	0.00E+00	4.51E-91	0.412	31	0.00E+00	2.52E-94	30	31	0.2	c.2.1.2
1a27_0	63	0.000	63	0.00E+00	0.00E+00	0.000	63	0.00E+00	0.00E+00	63	63	0.0	c.2.1.2
1fdt_0	50	0.517	34	0.00E+00	2.19E-94	0.504	41	0.00E+00	3.36E-115	34	41	0.3	c.2.1.2
1fdu_0	60	0.404	47	0.00E+00	8.20E-162	0.374	56	0.00E+00	2.93E-197	46	56	0.4	c.2.1.2
1fdu_1	49	0.417	34	0.00E+00	1.24E-109	0.465	43	0.00E+00	5.80E-129	34	43	0.3	c.2.1.2
1fdu_2	45	0.407	36	0.00E+00	6.81E-119	0.409	43	0.00E+00	2.05E-139	36	43	0.2	c.2.1.2

Table 8: Results for alcohol dehydrogenase (1hdx_1) matching against its own SCOP family with amino acid property.

Site	N	RMSD	q	Pvalue	Evalue	RMSD	q	Pvalue	Evalue	CG	Mean L	Var L	SCOP
1fdu_3	47	0.437	35	0.00E+00	6.62E-110	0.434	42	0.00E+00	9.19E-131	35	42	0.2	c.2.1.2
1equ_0	43	0.531	31	0.00E+00	6.04E-83	0.558	40	0.00E+00	4.11E-104	31	40	0.3	c.2.1.2
1equ_1	33	0.517	20	0.00E+00	1.48E-48	0.545	28	0.00E+00	1.41E-68	20	29	0.4	c.2.1.2
1fdv_0	52	0.436	40	0.00E+00	7.63E-129	0.461	51	0.00E+00	5.68E-157	40	51	0.3	c.2.1.2
1fdv_1	34	0.463	31	0.00E+00	7.50E-90	0.464	34	0.00E+00	3.44E-98	31	34	0.2	c.2.1.2
1fdv_3	32	0.434	23	0.00E+00	1.86E-66	0.575	30	0.00E+00	2.80E-72	23	30	0.2	c.2.1.2
1fdv_5	30	0.482	23	0.00E+00	1.61E-61	0.593	29	0.00E+00	1.70E-67	23	29	0.5	c.2.1.2
1fmc_0	47	0.628	13	0.00E+00	4.90E-17	0.881	21	0.00E+00	8.42E-27	13	21	0.9	c.2.1.2
1fmc_1	47	0.768	12	1.99E-11	1.99E-11	0.898	21	0.00E+00	4.33E-26	12	21	0.8	c.2.1.2
1ahi_0	48	0.665	12	2.15E-13	2.15E-13	0.780	17	0.00E+00	9.88E-22	11	19	14.0	c.2.1.2
1ahi_1	46	0.729	10	1.79E-07	1.79E-07	1.047	22	0.00E+00	3.62E-22	10	21	0.9	c.2.1.2
1ahh_0	32	0.780	12	1.96E-10	1.96E-10	0.910	18	0.00E+00	6.61E-20	12	18	0.8	c.2.1.2
1ahh_1	29	0.580	8	2.35E-06	2.35E-06	0.864	14	6.76E-13	6.76E-13	7	15	0.7	c.2.1.2
2hsd_0	35	0.587	11	8.44E-15	8.42E-15	0.602	16	0.00E+00	9.88E-27	11	16	18.6	c.2.1.2
2hsd_1	32	0.615	10	4.80E-11	4.80E-11	0.791	18	0.00E+00	3.21E-24	10	18	0.7	c.2.1.2
2hsd_2	29	0.594	11	8.33E-15	8.34E-15	0.630	16	0.00E+00	9.67E-26	11	17	0.4	c.2.1.2
2hsd_3	32	0.534	12	0.00E+00	1.93E-18	0.651	16	0.00E+00	7.85E-25	12	16	0.2	c.2.1.2
1fk8_0	30	1.010	19	0.00E+00	8.98E-19	0.702	18	0.00E+00	3.37E-28	17	18	0.6	c.2.1.2
1fk8_1	28	1.640	12	1.00E+00	1.93E+06	0.665	16	0.00E+00	1.52E-24	10	16	0.4	c.2.1.2
1nff_0	35	0.563	18	0.00E+00	5.34E-36	0.578	22	0.00E+00	2.16E-46	18	22	0.2	c.2.1.2
1nff_1	35	0.597	21	0.00E+00	1.37E-42	0.595	22	0.00E+00	3.17E-45	21	22	0.2	c.2.1.2
1nfr_0	43	0.559	15	0.00E+00	2.53E-28	0.563	20	0.00E+00	5.34E-41	15	20	0.2	c.2.1.2
1nfr_1	44	0.567	15	0.00E+00	7.14E-28	0.579	20	0.00E+00	5.82E-40	15	20	0.2	c.2.1.2
1nfr_2	43	0.547	15	0.00E+00	5.78E-29	0.561	20	0.00E+00	3.98E-41	15	20	0.2	c.2.1.2
1nfr_3	43	0.535	17	0.00E+00	8.51E-34	0.587	20	0.00E+00	1.69E-39	17	20	0.2	c.2.1.2
1bdb_0	54	0.825	21	0.00E+00	9.66E-31	0.745	30	0.00E+00	3.24E-56	19	31	1.0	c.2.1.2
1b14_0	50	0.790	20	0.00E+00	5.93E-30	0.804	27	0.00E+00	1.24E-44	19	27	0.8	c.2.1.2
1b14_1	35	0.811	15	0.00E+00	2.73E-17	0.818	20	0.00E+00	3.64E-28	15	20	0.8	c.2.1.2
1gee_0	32	0.695	13	0.00E+00	2.96E-17	0.729	20	0.00E+00	1.77E-32	13	21	1.4	c.2.1.2
1gee_1	32	0.845	15	3.33E-16	2.80E-16	0.714	18	0.00E+00	7.91E-28	13	20	1.5	c.2.1.2
1gee_2	31	0.802	12	1.29E-10	1.29E-10	0.693	17	0.00E+00	6.31E-26	10	19	11.0	c.2.1.2
1gee_3	33	0.847	18	0.00E+00	1.17E-22	0.650	18	0.00E+00	1.64E-30	15	20	9.7	c.2.1.2
1gco_0	33	0.723	14	0.00E+00	2.12E-18	0.791	22	0.00E+00	3.18E-34	13	22	1.3	c.2.1.2
1gco_1	33	0.767	17	0.00E+00	1.88E-23	0.800	22	0.00E+00	9.07E-34	15	22	1.2	c.2.1.2
1gco_2	33	0.762	14	1.11E-16	1.03E-16	0.815	22	0.00E+00	5.07E-33	13	22	1.3	c.2.1.2
1gco_3	34	0.844	19	0.00E+00	4.50E-25	0.795	22	0.00E+00	5.57E-34	18	22	1.4	c.2.1.2
1g6k_0	31	1.011	13	1.41E-07	1.41E-07	0.888	20	0.00E+00	3.42E-25	12	20	1.1	c.2.1.2
1g6k_1	31	0.984	16	7.32E-14	7.32E-14	0.525	14	0.00E+00	4.86E-24	12	18	25.4	c.2.1.2
1g6k_2	30	1.085	12	3.74E-04	3.74E-04	0.936	19	0.00E+00	2.40E-21	11	19	1.3	c.2.1.2
1g6k_3	32	1.013	18	2.22E-16	2.19E-16	0.898	20	0.00E+00	9.52E-25	17	20	1.0	c.2.1.2
1rwb_0	34	0.699	13	1.11E-16	8.79E-17	0.740	21	0.00E+00	2.32E-34	13	21	0.7	c.2.1.2
1rwb_1	32	0.813	16	0.00E+00	2.25E-19	0.744	20	0.00E+00	1.26E-31	15	21	1.1	c.2.1.2
1rwb_2	32	0.813	12	9.60E-11	9.60E-11	0.761	20	0.00E+00	8.15E-31	11	21	1.0	c.2.1.2
1rwb_3	33	0.734	14	0.00E+00	1.05E-17	0.729	20	0.00E+00	2.57E-32	14	21	1.3	c.2.1.2
1geg_0	38	0.737	16	0.00E+00	7.94E-23	0.632	21	0.00E+00	7.52E-40	15	21	0.5	c.2.1.2
1geg_1	40	0.701	16	0.00E+00	2.40E-24	0.586	22	0.00E+00	8.84E-44	15	22	0.5	c.2.1.2
1geg_2	38	0.693	19	0.00E+00	2.88E-31	0.616	22	0.00E+00	1.01E-43	18	22	0.5	c.2.1.2
1geg_3	39	0.690	20	0.00E+00	3.73E-34	0.684	22	0.00E+00	1.78E-39	20	22	0.4	c.2.1.2
1geg_4	40	0.696	19	0.00E+00	4.76E-31	0.567	21	0.00E+00	6.51E-44	17	22	0.8	c.2.1.2
1geg_5	39	0.696	20	0.00E+00	7.77E-34	0.625	21	0.00E+00	3.07E-40	19	21	0.7	c.2.1.2
1geg_6	40	0.720	16	0.00E+00	1.69E-23	0.672	23	0.00E+00	5.54E-43	16	23	0.4	c.2.1.2
1geg_7	39	0.702	16	0.00E+00	2.47E-24	0.612	21	0.00E+00	4.86E-41	15	21	0.7	c.2.1.2
1iy8_0	41	1.058	13	1.91E-05	1.91E-05	0.831	26	0.00E+00	1.54E-40	11	26	1.4	c.2.1.2
1iy8_1	40	0.873	18	0.00E+00	3.53E-21	0.911	26	0.00E+00	1.65E-36	17	25	1.4	c.2.1.2
1iy8_2	40	0.884	14	2.91E-12	2.91E-12	0.751	23	0.00E+00	3.78E-38	13	25	1.7	c.2.1.2
1iy8_3	40	0.872	18	0.00E+00	3.24E-21	0.866	25	0.00E+00	1.08E-36	17	25	1.4	c.2.1.2
1iy8_4	41	0.890	14	4.81E-12	4.81E-12	0.842	26	0.00E+00	6.56E-40	13	26	1.4	c.2.1.2
1iy8_5	40	0.902	15	3.73E-14	3.73E-14	0.822	24	0.00E+00	1.34E-36	13	25	1.4	c.2.1.2
1iy8_6	40	0.898	14	7.85E-12	7.85E-12	0.823	24	0.00E+00	8.57E-37	13	25	1.6	c.2.1.2
1iy8_7	40	1.054	15	1.98E-09	1.98E-09	0.812	24	0.00E+00	3.81E-37	12	25	1.5	c.2.1.2
1h5q_0	88	0.695	19	0.00E+00	1.78E-31	0.780	26	0.00E+00	3.68E-43	19	25	0.8	c.2.1.2
1h5q_2	95	0.705	20	0.00E+00	6.27E-34	0.734	27	0.00E+00	1.16E-48	20	26	0.6	c.2.1.2
1h5q_4	89	0.848	23	0.00E+00	2.25E-32	0.748	26	0.00E+00	3.45E-45	22	26	0.8	c.2.1.2
1h5q_5	91	0.739	24	0.00E+00	2.75E-41	0.738	26	0.00E+00	8.15E-46	24	25	0.7	c.2.1.2
1h5q_6	116	0.845	19	0.00E+00	8.81E-24	0.740	26	0.00E+00	2.30E-45	18	26	1.1	c.2.1.2
1h5q_7	117	1.188	19	6.59E-11	6.59E-11	0.737	26	0.00E+00	1.50E-45	18	26	1.3	c.2.1.2

Table 9: Results for alcohol dehydrogenase (1hdx_1) matching against its own SCOP family with amino acid property.

Site	N	RMSD	q	Pvalue	Evalue	RMSD	q	Pvalue	Evalue	CG	Mean L	Var L	SCOP
1h5q_9	91	1.190	19	3.57E-11	3.57E-11	0.717	26	0.00E+00	3.18E-47	18	25	0.8	c.2.1.2
1h5q_10	91	0.854	19	0.00E+00	1.07E-23	0.740	26	0.00E+00	1.10E-45	18	25	0.8	c.2.1.2
1h5q_11	92	0.831	24	0.00E+00	2.64E-35	0.756	26	0.00E+00	1.26E-44	23	25	0.8	c.2.1.2
1h5q_13	90	0.807	24	0.00E+00	1.16E-36	0.723	26	0.00E+00	7.85E-47	23	25	0.6	c.2.1.2
1edo_0	35	0.847	16	0.00E+00	6.53E-18	0.728	19	0.00E+00	2.20E-29	15	19	0.5	c.2.1.2
1q7c_0	19	0.805	9	1.83E-03	1.83E-03	0.805	9	1.83E-03	1.83E-03	9	8	0.5	c.2.1.2
1q7c_1	20	0.846	7	1.00E+00	1.57E+02	0.647	8	9.33E-04	9.34E-04	6	9	1.5	c.2.1.2
1q7b_8	30	0.828	17	0.00E+00	4.06E-21	0.856	19	0.00E+00	3.87E-24	17	19	0.7	c.2.1.2
1q7b_9	30	0.800	17	0.00E+00	5.11E-22	0.835	18	0.00E+00	9.34E-23	17	17	0.8	c.2.1.2
1q7b_10	30	0.838	13	4.05E-12	4.05E-12	0.833	18	0.00E+00	7.81E-23	13	18	0.8	c.2.1.2
1q7b_11	29	0.831	13	2.24E-12	2.24E-12	0.830	18	0.00E+00	5.36E-23	13	18	0.6	c.2.1.2
1o5i_0	29	1.353	9	1.00E+00	7.61E+04	1.159	14	3.12E-06	3.12E-06	8	13	0.8	c.2.1.2
1eno_0	30	0.843	12	3.74E-09	3.74E-09	0.816	13	8.32E-12	8.32E-12	11	14	0.8	c.2.1.2
1d7o_0	32	0.861	12	1.24E-08	1.24E-08	0.910	14	1.60E-11	1.60E-11	12	14	0.9	c.2.1.2
1cwu_0	40	0.911	15	1.12E-13	1.12E-13	0.900	17	0.00E+00	7.13E-18	14	18	1.2	c.2.1.2
1cwu_1	40	0.743	11	2.95E-09	2.95E-09	0.979	18	0.00E+00	2.71E-17	11	18	1.1	c.2.1.2
1nhd_0	34	0.817	11	2.44E-07	2.44E-07	0.819	14	4.40E-14	4.39E-14	11	15	1.1	c.2.1.2
1nhd_1	34	0.773	10	9.00E-07	9.00E-07	0.730	13	3.46E-14	3.46E-14	9	14	1.0	c.2.1.2
1nhg_0	38	0.787	10	2.92E-05	2.92E-05	0.828	14	1.13E-13	1.13E-13	10	16	1.9	c.2.1.2
1nhg_1	39	0.784	9	6.06E-04	6.06E-04	0.735	13	7.45E-14	7.45E-14	8	15	2.0	c.2.1.2
1nhw_0	37	0.824	10	1.50E-04	1.50E-04	0.845	14	3.20E-13	3.20E-13	10	16	1.6	c.2.1.2
1nhw_1	37	2.894	8	1.00E+00	2.32E+18	0.863	14	1.02E-12	1.02E-12	7	15	1.7	c.2.1.2
1nnu_0	38	0.841	11	1.17E-06	1.17E-06	0.840	14	2.50E-13	2.50E-13	11	16	1.8	c.2.1.2
1nnu_1	41	0.755	9	1.66E-04	1.66E-04	0.822	14	9.58E-14	9.58E-14	9	16	1.7	c.2.1.2
1uh5_0	38	0.820	9	9.62E-03	9.67E-03	0.734	13	6.43E-14	6.42E-14	8	15	8.5	c.2.1.2
1uh5_1	38	0.781	9	4.83E-04	4.83E-04	0.853	14	5.84E-13	5.84E-13	9	16	1.7	c.2.1.2
1eny_0	28	0.997	11	7.62E-04	7.62E-04	0.784	14	1.67E-15	1.71E-15	10	14	6.3	c.2.1.2
1p44_0	39	0.815	9	2.57E-02	2.61E-02	0.855	15	4.33E-15	4.34E-15	9	16	1.4	c.2.1.2
1p44_1	43	1.752	9	1.00E+00	4.10E+10	0.965	15	1.07E-11	1.07E-11	8	16	1.9	c.2.1.2
1p44_2	41	0.811	9	9.41E-03	9.45E-03	1.008	15	1.39E-10	1.39E-10	9	15	5.1	c.2.1.2
1p44_3	44	0.839	9	5.21E-02	5.35E-02	0.893	15	1.23E-13	1.23E-13	9	16	1.6	c.2.1.2
1p44_4	29	0.761	9	1.34E-04	1.34E-04	0.802	14	6.99E-15	6.99E-15	9	15	0.9	c.2.1.2
1p44_5	28	1.547	7	1.00E+00	6.17E+09	1.547	7	1.00E+00	6.17E+09	7	8	0.4	c.2.1.2
1enz_0	28	1.499	8	1.00E+00	1.52E+08	0.843	13	2.38E-11	2.38E-11	6	14	1.0	c.2.1.2
1p45_0	43	0.781	10	8.49E-06	8.49E-06	0.937	15	1.73E-12	1.73E-12	10	16	1.9	c.2.1.2
1p45_1	34	0.818	9	1.26E-02	1.26E-02	0.941	16	1.12E-14	1.12E-14	9	16	9.7	c.2.1.2
1bvr_0	39	1.588	8	1.00E+00	8.17E+09	0.870	14	1.83E-12	1.83E-12	7	15	1.4	c.2.1.2
1bvr_1	39	0.786	9	9.94E-03	9.99E-03	0.874	14	2.38E-12	2.38E-12	9	15	1.5	c.2.1.2
1bvr_2	39	1.629	8	1.00E+00	3.58E+10	0.875	14	2.54E-12	2.54E-12	7	15	1.2	c.2.1.2
1bvr_3	39	1.625	8	1.00E+00	3.30E+10	0.874	14	2.38E-12	2.38E-12	7	15	1.4	c.2.1.2
1bvr_4	28	0.814	8	4.30E-01	5.63E-01	0.849	13	3.45E-11	3.45E-11	8	13	11.0	c.2.1.2
1bvr_5	30	0.884	10	4.70E-04	4.70E-04	1.087	15	5.71E-09	5.71E-09	10	15	1.4	c.2.1.2
1qsg_0	37	0.871	9	3.83E-02	3.91E-02	1.117	16	1.08E-09	1.08E-09	9	17	1.9	c.2.1.2
1qsg_1	36	0.883	8	1.00E+00	1.43E+01	1.125	16	1.60E-09	1.60E-09	8	18	1.9	c.2.1.2
1qsg_2	36	1.299	8	1.00E+00	3.42E+06	1.130	16	2.15E-09	2.15E-09	5	17	1.7	c.2.1.2
1qsg_3	36	0.875	9	9.63E-02	1.01E-01	1.102	16	4.00E-10	4.00E-10	9	17	2.1	c.2.1.2
1qsg_4	37	0.893	8	1.00E+00	2.26E+01	1.139	16	3.98E-09	3.98E-09	8	17	3.3	c.2.1.2
1qsg_5	36	0.844	8	9.56E-01	3.13E+00	1.126	16	1.69E-09	1.69E-09	8	17	1.8	c.2.1.2
1qsg_6	36	0.907	8	1.00E+00	4.37E+01	1.098	16	3.13E-10	3.13E-10	8	17	1.9	c.2.1.2
1qsg_7	36	0.854	8	9.90E-01	4.65E+00	1.123	16	1.42E-09	1.42E-09	8	17	1.6	c.2.1.2
1qg6_0	37	0.859	10	2.09E-04	2.09E-04	1.011	15	9.22E-11	9.22E-11	10	17	1.9	c.2.1.2
1qg6_1	37	1.754	9	1.00E+00	1.54E+10	1.093	16	2.51E-10	2.51E-10	0	17	2.0	c.2.1.2
1qg6_2	37	0.847	9	3.31E-02	3.36E-02	1.093	16	2.51E-10	2.51E-10	9	17	1.8	c.2.1.2
1qg6_3	37	0.836	9	8.02E-03	8.06E-03	1.093	16	2.51E-10	2.51E-10	9	16	8.4	c.2.1.2
1dfi_0	28	0.881	9	7.57E-02	7.87E-02	0.938	12	4.70E-07	4.70E-07	9	13	1.1	c.2.1.2
1dfi_1	27	0.850	8	9.62E-01	3.28E+00	0.987	12	4.66E-06	4.66E-06	8	12	0.9	c.2.1.2
1dfi_2	26	0.909	7	1.00E+00	1.97E+03	0.824	7	1.00E+00	4.33E+02	5	11	8.4	c.2.1.2
1dfi_3	29	0.873	7	1.00E+00	1.66E+03	0.937	12	4.99E-07	4.99E-07	7	13	1.2	c.2.1.2
1c14_0	38	0.844	11	6.59E-07	6.59E-07	1.108	17	1.57E-11	1.57E-11	11	17	2.1	c.2.1.2
1c14_1	38	0.850	11	1.74E-06	1.74E-06	1.046	16	1.43E-11	1.43E-11	11	17	2.0	c.2.1.2
1dfh_0	34	0.813	10	3.42E-05	3.42E-05	0.937	14	6.29E-11	6.29E-11	10	14	1.2	c.2.1.2
1dfh_1	33	0.805	11	3.60E-08	3.60E-08	0.828	13	8.93E-12	8.93E-12	11	14	1.0	c.2.1.2
1dfg_0	34	0.838	10	1.13E-04	1.13E-04	0.884	13	2.98E-10	2.98E-10	10	14	1.1	c.2.1.2
1dfg_1	38	0.797	11	5.66E-08	5.66E-08	0.932	14	6.53E-11	6.53E-11	11	14	1.1	c.2.1.2
1mfp_0	43	0.873	11	1.10E-05	1.10E-05	1.127	18	1.95E-12	1.95E-12	11	19	1.7	c.2.1.2
1mfp_1	42	1.103	12	7.30E-03	7.33E-03	1.144	19	1.55E-13	1.55E-13	12	19	2.2	c.2.1.2

Table 10: Results for alcohol dehydrogenase (1hdx_1) matching against its own SCOP family with amino acid property.

Site	N	RMSD	q	Pvalue	Evalue	RMSD	q	Pvalue	Evalue	CG	Mean L	Var L	SCOP
1d8a_0	35	0.924	10	7.63E-03	7.66E-03	1.138	17	8.30E-11	8.30E-11	10	17	1.3	c.2.1.2
1d8a_1	36	1.007	11	3.56E-03	3.57E-03	0.997	16	4.88E-13	4.88E-13	11	17	1.5	c.2.1.2
1lx6_0	32	0.909	9	2.46E-01	2.83E-01	1.039	14	2.52E-08	2.52E-08	9	14	1.2	c.2.1.2
1lx6_1	33	1.014	11	3.70E-03	3.71E-03	1.019	14	9.14E-09	9.14E-09	11	14	1.2	c.2.1.2
1lxc_0	39	0.893	11	2.21E-05	2.21E-05	1.006	16	1.14E-12	1.14E-12	11	17	1.5	c.2.1.2
1lxc_1	38	1.006	12	5.77E-05	5.77E-05	0.995	16	5.04E-13	5.04E-13	12	17	1.4	c.2.1.2
1i30_0	31	0.823	11	2.21E-07	2.21E-07	0.843	14	1.12E-13	1.12E-13	11	15	1.4	c.2.1.2
1i30_1	31	0.963	12	2.71E-06	2.71E-06	0.961	15	2.29E-12	2.29E-12	12	15	0.8	c.2.1.2
1i2z_0	39	0.949	12	3.53E-06	3.53E-06	0.919	16	2.66E-15	2.66E-15	12	17	1.4	c.2.1.2
1i2z_1	38	0.850	12	1.08E-08	1.08E-08	1.015	17	2.85E-14	2.86E-14	12	17	1.1	c.2.1.2
1jw7_0	36	0.915	11	3.81E-05	3.81E-05	1.034	15	3.57E-10	3.57E-10	11	16	1.9	c.2.1.2
1jw7_1	37	0.824	10	2.47E-05	2.47E-05	1.032	15	3.43E-10	3.43E-10	10	16	1.8	c.2.1.2
1jw7_2	37	0.807	11	3.66E-08	3.66E-08	1.035	15	4.13E-10	4.13E-10	11	16	2.6	c.2.1.2
1jw7_3	37	0.824	10	2.47E-05	2.47E-05	1.028	15	2.67E-10	2.67E-10	10	16	1.8	c.2.1.2
1jvf_0	35	0.936	11	9.55E-05	9.55E-05	0.915	14	1.44E-11	1.44E-11	11	15	6.4	c.2.1.2
1jvf_1	36	0.865	10	1.42E-04	1.42E-04	0.916	14	1.68E-11	1.68E-11	10	16	1.6	c.2.1.2
1jvf_2	36	0.869	10	1.73E-04	1.73E-04	1.072	15	3.60E-09	3.60E-09	10	16	1.7	c.2.1.2
1jvf_3	35	0.865	10	1.30E-04	1.30E-04	1.066	15	2.30E-09	2.30E-09	10	16	1.6	c.2.1.2
1ae1_0	33	0.901	12	1.95E-07	1.95E-07	0.758	16	0.00E+00	2.22E-20	11	17	1.2	c.2.1.2
1ae1_1	35	0.944	13	3.79E-09	3.79E-09	0.520	14	0.00E+00	7.52E-24	8	17	17.3	c.2.1.2
2ae2_0	43	1.022	12	8.18E-05	8.18E-05	0.745	19	0.00E+00	4.42E-28	10	20	1.4	c.2.1.2
2ae2_1	44	0.875	12	2.22E-07	2.22E-07	0.807	19	0.00E+00	1.80E-25	10	20	1.3	c.2.1.2
1ipe_0	35	0.935	12	2.87E-07	2.87E-07	0.679	18	0.00E+00	8.35E-29	10	20	1.0	c.2.1.2
1ipe_1	37	0.917	13	8.31E-10	8.31E-10	0.916	22	0.00E+00	3.59E-28	13	21	0.9	c.2.1.2
1ipf_0	45	0.976	13	7.55E-08	7.55E-08	0.741	20	0.00E+00	1.07E-30	10	22	1.6	c.2.1.2
1ipf_1	45	1.016	14	1.18E-08	1.18E-08	0.843	21	0.00E+00	2.06E-28	13	21	1.6	c.2.1.2
1g0o_0	42	0.766	10	3.57E-06	3.57E-06	0.760	19	0.00E+00	3.44E-27	10	19	0.4	c.2.1.2
1g0o_1	42	0.698	11	4.19E-10	4.19E-10	0.745	19	0.00E+00	7.59E-28	11	19	0.4	c.2.1.2
1g0o_2	43	0.701	16	0.00E+00	1.75E-22	0.748	20	0.00E+00	2.78E-30	16	19	9.4	c.2.1.2
1g0o_3	43	0.772	10	5.39E-06	5.39E-06	0.639	19	0.00E+00	5.49E-33	9	19	0.6	c.2.1.2
1g0n_0	43	0.755	11	5.40E-09	5.40E-09	0.609	19	0.00E+00	1.43E-34	10	19	0.5	c.2.1.2
1g0n_1	35	0.780	10	3.39E-06	3.39E-06	0.746	19	0.00E+00	3.63E-28	10	19	0.4	c.2.1.2
1doh_0	43	0.779	11	2.45E-08	2.45E-08	0.632	19	0.00E+00	2.38E-33	10	20	0.5	c.2.1.2
1doh_1	42	0.765	11	9.49E-09	9.49E-09	0.630	18	0.00E+00	1.61E-30	10	18	3.4	c.2.1.2
1ybv_0	113	0.584	17	0.00E+00	6.87E-32	0.725	27	0.00E+00	3.09E-48	17	27	1.4	c.2.1.2
1ybv_1	113	0.590	21	0.00E+00	1.29E-41	0.725	27	0.00E+00	3.09E-48	21	27	1.1	c.2.1.2
1ja9_0	43	0.692	13	1.11E-16	1.33E-16	0.747	19	0.00E+00	3.08E-28	13	19	0.5	c.2.1.2
1hdo_0	109	7.403	8	1.00E+00	7.78E+31	1.359	19	2.81E-06	2.81E-06	0	19	2.0	c.2.1.2
1he2_0	120	1.965	8	1.00E+00	1.10E+14	1.963	9	1.00E+00	1.09E+13	8	10	0.8	c.2.1.2
1he3_0	120	1.562	8	1.00E+00	2.69E+11	1.562	8	1.00E+00	2.69E+11	8	9	1.1	c.2.1.2
1he4_0	115	1.564	8	1.00E+00	2.47E+11	1.564	8	1.00E+00	2.47E+11	8	9	1.0	c.2.1.2
1he5_0	114	4.483	8	1.00E+00	2.30E+26	1.426	19	1.65E-04	1.65E-04	0	19	2.8	c.2.1.2
1e6w_0	111	0.865	28	0.00E+00	4.97E-42	0.770	29	0.00E+00	1.76E-50	26	29	26.0	c.2.1.2
1e6w_1	49	0.758	21	0.00E+00	7.64E-34	0.799	31	0.00E+00	2.56E-54	20	31	1.3	c.2.1.2
1e6w_2	51	0.801	22	0.00E+00	1.29E-33	0.791	30	0.00E+00	2.20E-52	21	30	1.2	c.2.1.2
1e6w_3	111	0.851	25	0.00E+00	3.51E-36	0.793	31	0.00E+00	1.10E-53	24	31	1.5	c.2.1.2
1e3s_0	47	0.786	20	0.00E+00	4.15E-30	0.812	30	0.00E+00	4.13E-51	19	30	1.3	c.2.1.2
1e3s_1	47	0.819	21	0.00E+00	1.49E-30	0.814	30	0.00E+00	5.72E-51	20	30	1.2	c.2.1.2
1e3s_2	46	0.778	20	0.00E+00	1.51E-30	0.811	30	0.00E+00	3.29E-51	19	30	1.2	c.2.1.2
1e3s_3	47	0.824	21	0.00E+00	1.77E-30	0.815	30	0.00E+00	6.73E-51	20	30	1.2	c.2.1.2
1e3w_0	49	0.833	20	0.00E+00	1.03E-27	0.827	31	0.00E+00	2.87E-52	19	31	1.5	c.2.1.2
1e3w_1	49	0.795	21	0.00E+00	6.08E-32	0.790	31	0.00E+00	5.43E-55	20	31	1.3	c.2.1.2
1e3w_2	53	0.821	20	0.00E+00	3.41E-28	0.772	30	0.00E+00	7.58E-54	18	31	1.6	c.2.1.2
1e3w_3	55	0.782	20	0.00E+00	4.19E-30	0.789	31	0.00E+00	6.51E-55	19	31	1.4	c.2.1.2
1n5d_1	33	0.636	13	0.00E+00	3.24E-19	0.686	18	0.00E+00	5.63E-30	13	18	0.9	c.2.1.2
1sb8_0	55	0.946	11	2.03E-04	2.03E-04	0.786	13	7.33E-13	7.33E-13	10	13	1.9	c.2.1.2
1sb9_0	53	0.906	11	2.25E-05	2.25E-05	0.726	13	8.88E-15	8.84E-15	10	13	0.5	c.2.1.2
1vl8_2	37	0.748	12	2.34E-12	2.34E-12	0.827	19	0.00E+00	7.96E-25	12	20	0.7	c.2.1.2
1vl8_3	37	0.671	12	2.89E-14	2.88E-14	0.826	19	0.00E+00	7.27E-25	12	20	0.7	c.2.1.2
1sny_1	40	0.739	14	0.00E+00	8.78E-18	0.729	20	0.00E+00	1.38E-32	13	21	1.7	c.2.1.2
1xhl_0	43	0.795	16	0.00E+00	1.68E-20	0.832	24	0.00E+00	4.36E-36	15	24	2.8	c.2.1.2
1xhl_1	43	0.748	16	0.00E+00	1.97E-22	0.740	23	0.00E+00	8.48E-39	15	24	1.2	c.2.1.2
