# Supplementary material for: Bayesian refinement of protein functional site matching
Source: BMC Bioinformatics. 2007 Jul 17;8:257. doi: 10.1186/1471-2105-8-257 (PMC1940029; doi:10.1186/1471-2105-8-257)
Supplement: Additional file 1 — Case 1 Results. Results for alcohol dehydrogenase (1hdx_1) matching against its own SCOP family. Tables 1–2: Without amino acid property. Tables 3–4: With amino acid property [file 1471-2105-8-257-S1.pdf]

Results for 1hdx_1 matching against its own SCOP family.

Table 1: Results for alcohol dehydrogenase (1hdx_1) matching against its own SCOP family without amino acid property.

Site	N	RMSD	q	Pvalue	Evalue	RMSD	q	Pvalue	Evalue	CG	Mean L	Var L	SCOP
1het_0	109	0.476	58	0.00E+00	6.11E-155	0.448	57	0.00E+00	1.59E-158	56	57	0.6	c.2.1.1
1het_2	118	0.396	53	0.00E+00	2.47E-160	0.413	58	0.00E+00	9.54E-171	52	58	0.4	c.2.1.1
1heu_0	118	0.44	56	0.00E+00	3.75E-157	0.436	55	0.00E+00	7.21E-155	54	55	0.8	c.2.1.1
1heu_2	112	0.434	53	0.00E+00	1.03E-150	0.431	57	0.00E+00	9.66E-163	52	57	0.5	c.2.1.1
1mgo_0	37	0.436	33	0.00E+00	2.48E-86	0.433	33	0.00E+00	2.15E-86	32	33	0.1	c.2.1.1
1mgo_1	43	0.493	38	0.00E+00	3.60E-93	0.493	38	0.00E+00	3.60E-93	38	37	0.4	c.2.1.1
1ee2_0	48	0.441	37	0.00E+00	3.05E-98	0.439	36	0.00E+00	2.59E-95	36	36	0.2	c.2.1.1
1ee2_1	48	0.453	37	0.00E+00	2.53E-96	0.451	36	0.00E+00	1.91E-93	36	36	0.2	c.2.1.1
1ju9_4	20	0.453	16	0.00E+00	2.95E-31	0.453	16	0.00E+00	2.95E-31	16	16	0.3	c.2.1.1
1ju9_5	18	0.436	16	0.00E+00	1.76E-32	0.436	16	0.00E+00	1.76E-32	16	16	0.4	c.2.1.1
1qv6_0	44	0.458	40	0.00E+00	8.04E-105	0.458	40	0.00E+00	8.04E-105	40	39	0.4	c.2.1.1
1qv6_1	43	0.431	39	0.00E+00	1.30E-106	0.431	39	0.00E+00	3.08E-106	38	39	0.3	c.2.1.1
3bto_0	52	0.436	41	0.00E+00	5.04E-112	0.429	41	0.00E+00	6.13E-113	40	41	0.4	c.2.1.1
3bto_1	43	0.441	38	0.00E+00	1.24E-101	0.435	38	0.00E+00	2.84E-102	37	38	0.4	c.2.1.1
3bto_2	43	0.456	39	0.00E+00	2.42E-102	0.451	39	0.00E+00	7.66E-103	38	39	0.2	c.2.1.1
3bto_3	43	0.468	39	0.00E+00	2.24E-100	0.464	39	0.00E+00	1.03E-100	38	39	0.3	c.2.1.1
1qv7_0	42	0.461	39	0.00E+00	3.13E-101	0.425	37	0.00E+00	1.13E-100	37	38	0.4	c.2.1.1
1qv7_1	43	0.448	38	0.00E+00	1.79E-100	0.447	38	0.00E+00	2.71E-100	37	38	0.3	c.2.1.1
1mg0_0	42	0.436	39	0.00E+00	9.01E-106	0.436	38	0.00E+00	1.67E-102	38	38	0.3	c.2.1.1
1mg0_1	41	0.441	38	0.00E+00	1.07E-101	0.441	38	0.00E+00	1.07E-101	38	37	0.3	c.2.1.1
1mg0_2	42	0.428	39	0.00E+00	3.57E-107	0.428	38	0.00E+00	7.25E-104	38	38	0.2	c.2.1.1
1mg0_3	43	0.449	39	0.00E+00	1.63E-103	0.449	39	0.00E+00	3.56E-103	38	39	0.4	c.2.1.1
2ohx_0	117	0.365	57	0.00E+00	5.89E-181	0.361	56	0.00E+00	1.70E-178	55	56	0.7	c.2.1.1
2ohx_1	119	0.362	52	0.00E+00	3.81E-166	0.374	57	0.00E+00	2.95E-178	51	57	0.3	c.2.1.1
1hf3_0	120	0.394	52	0.00E+00	2.32E-157	0.367	55	0.00E+00	4.77E-173	50	56	0.4	c.2.1.1
1hf3_2	64	0.405	54	0.00E+00	3.44E-162	0.378	57	0.00E+00	6.64E-178	52	57	0.4	c.2.1.1
2oxi_0	116	0.378	52	0.00E+00	1.06E-161	0.398	57	0.00E+00	1.88E-171	51	57	0.3	c.2.1.1
2oxi_1	119	0.378	52	0.00E+00	1.15E-161	0.348	55	0.00E+00	1.12E-178	50	56	0.7	c.2.1.1
1axe_0	40	0.423	37	0.00E+00	1.83E-101	0.423	37	0.00E+00	1.83E-101	37	36	0.2	c.2.1.1
1axe_1	41	0.413	38	0.00E+00	4.12E-106	0.413	38	0.00E+00	4.12E-106	38	37	0.2	c.2.1.1
1a71_0	42	0.467	37	0.00E+00	5.07E-94	0.467	37	0.00E+00	5.07E-94	37	36	0.4	c.2.1.1
1a71_1	42	0.504	38	0.00E+00	1.35E-91	0.504	38	0.00E+00	1.35E-91	38	35	31	c.2.1.1
1bto_0	52	0.464	41	0.00E+00	4.82E-107	0.46	41	0.00E+00	2.04E-107	40	41	0.5	c.2.1.1
1bto_1	43	0.465	38	0.00E+00	9.86E-98	0.462	38	0.00E+00	6.80E-98	37	38	0.7	c.2.1.1
1bto_2	43	0.502	39	0.00E+00	4.59E-95	0.463	38	0.00E+00	9.77E-98	37	38	0.5	c.2.1.1
1bto_3	43	0.504	39	0.00E+00	9.19E-95	0.462	38	0.00E+00	6.80E-98	37	38	0.8	c.2.1.1
1hld_0	120	0.384	46	0.00E+00	8.79E-139	0.371	58	0.00E+00	9.59E-183	45	57	0.5	c.2.1.1
1hld_1	120	0.406	45	0.00E+00	2.62E-130	0.357	57	0.00E+00	2.33E-183	43	57	0.4	c.2.1.1
1qlh_0	45	0.555	24	0.00E+00	1.35E-46	0.782	26	0.00E+00	8.80E-35	22	27	4.5	c.2.1.1
1lde_0	51	0.452	40	0.00E+00	5.65E-106	0.447	40	0.00E+00	1.70E-106	39	40	0.4	c.2.1.1
1lde_1	41	0.459	35	0.00E+00	2.81E-89	0.457	35	0.00E+00	3.02E-89	34	35	0.4	c.2.1.1
1lde_2	39	0.435	36	0.00E+00	7.40E-96	0.433	35	0.00E+00	6.87E-93	35	35	0.5	c.2.1.1
1lde_3	40	0.445	36	0.00E+00	2.87E-94	0.449	36	0.00E+00	1.18E-93	35	36	0.4	c.2.1.1
1axg_0	44	0.48	39	0.00E+00	3.81E-98	0.473	38	0.00E+00	3.76E-96	38	38	0.5	c.2.1.1
1axg_1	42	0.464	38	0.00E+00	1.30E-97	0.494	38	0.00E+00	4.70E-93	37	38	0.3	c.2.1.1
1axg_2	44	0.51	39	0.00E+00	1.32E-93	0.51	38	0.00E+00	1.13E-90	38	38	0.5	c.2.1.1
1axg_3	42	0.436	38	0.00E+00	3.88E-102	0.435	37	0.00E+00	4.95E-99	37	37	0.4	c.2.1.1
1ldy_0	52	0.461	42	0.00E+00	1.04E-110	0.421	41	0.00E+00	1.98E-114	40	41	0.4	c.2.1.1
1ldy_1	42	0.468	38	0.00E+00	2.73E-97	0.423	37	0.00E+00	5.25E-101	36	37	0.4	c.2.1.1
1ldy_2	42	0.48	38	0.00E+00	1.99E-95	0.444	37	0.00E+00	1.38E-97	36	38	0.4	c.2.1.1
1ldy_3	42	0.465	38	0.00E+00	1.87E-97	0.436	37	0.00E+00	7.20E-99	36	37	0.4	c.2.1.1
6adh_0	120	0.442	49	0.00E+00	9.74E-136	0.516	56	0.00E+00	5.90E-140	48	56	0.7	c.2.1.1
6adh_1	120	0.54	55	0.00E+00	3.03E-132	0.574	56	0.00E+00	1.79E-128	54	55	0.6	c.2.1.1
1ht0_0	38	0.312	35	0.00E+00	2.60E-115	0.306	35	0.00E+00	6.09E-116	34	35	0.1	c.2.1.1
1ht0_1	38	0.367	34	0.00E+00	9.09E-101	0.36	34	0.00E+00	1.73E-101	33	34	0.1	c.2.1.1
1deh_1	60	0.286	57	0.00E+00	1.19E-208	0.286	57	0.00E+00	1.19E-208	57	57	0.2	c.2.1.1
1deh_3	59	0.302	51	0.00E+00	8.74E-182	0.299	56	0.00E+00	1.04E-199	51	56	0.2	c.2.1.1
1mp0_9	36	0.574	25	0.00E+00	1.22E-47	0.6	27	0.00E+00	1.08E-50	25	27	1	c.2.1.1
1mp0_10	36	0.607	27	0.00E+00	3.36E-50	0.632	29	0.00E+00	4.39E-53	27	30	1.1	c.2.1.1
1hsz_0	39	0.312	35	0.00E+00	2.82E-115	0.311	36	0.00E+00	7.98E-119	35	36	0.1	c.2.1.1
1hsz_1	38	0.314	36	0.00E+00	3.35E-118	0.314	36	0.00E+00	3.35E-118	36	36	0.1	c.2.1.1
1htb_1	60	0.295	56	0.00E+00	3.86E-201	0.266	55	0.00E+00	5.74E-208	55	56	0.3	c.2.1.1
1htb_3	58	0.356	50	0.00E+00	2.03E-161	0.357	55	0.00E+00	6.32E-177	50	55	0.2	c.2.1.1
1ma0_0	47	0.673	27	0.00E+00	1.15E-44	0.66	32	0.00E+00	1.49E-57	25	33	1.5	c.2.1.1
1ma0_1	47	0.618	32	0.00E+00	1.50E-61	0.64	34	0.00E+00	2.75E-64	32	34	0.8	c.2.1.1
1hdy_1	59	0.287	55	0.00E+00	1.09E-200	0.255	54	0.00E+00	7.24E-209	54	55	0.3	c.2.1.1
1hdy_3	58	0.302	51	0.00E+00	8.30E-182	0.273	55	0.00E+00	2.87E-205	50	55	0.3	c.2.1.1
1hdx_1	60	0	60	0.00E+00	0.00E+00	0	60	0.00E+00	0.00E+00	60	60	0	c.2.1.1
1hdx_3	59	0.257	54	0.00E+00	1.59E-210	0.254	58	0.00E+00	3.84E-226	54	58	0.1	c.2.1.1

Table 2: Results for alcohol dehydrogenase (1hdx_1) matching against its own SCOP family without amino acid property.

Site	N	RMSD	q	Pvalue	Evalue	RMSD	q	Pvalue	Evalue	CG	Mean L	Var L	SCOP
1hdz_1	58	0.32	55	0.00E+00	7.50E-188	0.32	54	0.00E+00	1.48E-184	54	54	0.1	c.2.1.1
1hdz_3	56	0.345	50	0.00E+00	5.16E-164	0.34	53	0.00E+00	4.16E-175	49	53	0.1	c.2.1.1
1d1t_0	38	0.473	34	0.00E+00	2.88E-84	0.467	33	0.00E+00	1.14E-81	32	33	0.2	c.2.1.1
1d1t_1	39	0.462	34	0.00E+00	9.20E-86	0.404	32	0.00E+00	3.43E-87	31	32	0.5	c.2.1.1
1d1t_2	39	0.479	34	0.00E+00	2.06E-83	0.479	34	0.00E+00	2.06E-83	34	33	0.3	c.2.1.1
1d1t_3	38	0.429	33	0.00E+00	2.59E-87	0.397	31	0.00E+00	8.36E-85	30	32	0.3	c.2.1.1
1mc5_0	51	0.55	35	0.00E+00	7.75E-77	0.616	37	0.00E+00	3.24E-74	35	37	0.6	c.2.1.1
1mc5_8	35	0.581	27	0.00E+00	2.59E-52	0.609	28	0.00E+00	1.50E-52	27	29	1.3	c.2.1.1
1d1s_0	38	0.465	33	0.00E+00	3.02E-82	0.465	33	0.00E+00	3.02E-82	33	32	0.2	c.2.1.1
1d1s_1	40	0.456	34	0.00E+00	1.40E-86	0.456	34	0.00E+00	1.40E-86	34	33	0.4	c.2.1.1
1d1s_2	40	0.442	34	0.00E+00	1.32E-88	0.391	32	0.00E+00	4.07E-89	31	33	0.4	c.2.1.1
1d1s_3	40	0.532	34	0.00E+00	1.49E-76	0.493	32	0.00E+00	3.09E-75	31	33	0.7	c.2.1.1
1teh_1	58	0.797	32	0.00E+00	1.16E-45	0.906	46	0.00E+00	1.13E-62	30	45	1.5	c.2.1.1
1teh_3	56	0.617	31	0.00E+00	7.91E-59	0.902	44	0.00E+00	1.10E-59	29	44	1.6	c.2.1.1
1hso_0	41	0.363	39	0.00E+00	3.71E-119	0.353	37	0.00E+00	2.34E-114	37	37	0.2	c.2.1.1
1hso_1	35	0.314	32	0.00E+00	8.90E-102	0.304	31	0.00E+00	2.52E-100	30	31	0.8	c.2.1.1
3hud_1	59	0.352	55	0.00E+00	2.15E-178	0.352	55	0.00E+00	2.15E-178	55	47	239.2	c.2.1.1
3hud_3	56	0.435	51	0.00E+00	7.69E-145	0.409	55	0.00E+00	1.32E-162	50	56	0.4	c.2.1.1
1agn_1	58	0.417	48	0.00E+00	5.46E-139	0.399	51	0.00E+00	2.42E-151	46	51	0.2	c.2.1.1
1agn_3	59	0.392	49	0.00E+00	2.45E-148	0.391	52	0.00E+00	8.69E-157	47	52	0.2	c.2.1.1
1agn_5	40	0.455	35	0.00E+00	6.73E-90	0.412	33	0.00E+00	2.26E-89	32	34	0.4	c.2.1.1
1agn_6	39	0.464	34	0.00E+00	1.76E-85	0.41	31	0.00E+00	2.50E-83	31	32	0.4	c.2.1.1
1e3i_0	120	0.627	36	0.00E+00	1.90E-69	0.641	39	0.00E+00	3.65E-75	33	39	1.7	c.2.1.1
1e3i_1	115	0.734	18	0.00E+00	2.95E-19	0.555	24	0.00E+00	4.81E-45	13	24	2.3	c.2.1.1
1e3i_2	44	0.644	28	0.00E+00	1.75E-49	0.692	29	0.00E+00	6.77E-48	25	30	1.3	c.2.1.1
1e3e_0	120	0.66	32	0.00E+00	2.49E-56	0.711	37	0.00E+00	5.81E-63	29	37	1.9	c.2.1.1
1e3l_0	118	0.591	39	0.00E+00	1.81E-81	0.555	38	0.00E+00	3.21E-83	34	38	1	c.2.1.1
1p0f_0	41	0.592	34	0.00E+00	2.26E-69	0.592	34	0.00E+00	2.26E-69	34	32	0.6	c.2.1.1
1p0f_1	41	0.618	35	0.00E+00	4.14E-69	0.618	35	0.00E+00	4.14E-69	35	32	0.7	c.2.1.1
1cdo_0	60	0.459	41	0.00E+00	4.84E-108	0.459	41	0.00E+00	4.84E-108	41	40	0.3	c.2.1.1
1r37_0	66	0.844	29	0.00E+00	2.13E-36	0.98	35	0.00E+00	4.95E-38	26	35	1.8	c.2.1.1
1r37_2	66	0.891	29	0.00E+00	3.76E-33	0.977	35	0.00E+00	3.10E-38	24	35	1.9	c.2.1.1
1llu_0	42	0.69	26	0.00E+00	2.92E-41	0.841	26	0.00E+00	1.31E-31	23	27	1.2	c.2.1.1
1llu_1	41	0.859	23	0.00E+00	3.68E-25	0.974	25	0.00E+00	6.20E-23	20	25	1.3	c.2.1.1
1llu_2	42	0.69	26	0.00E+00	2.92E-41	0.841	26	0.00E+00	1.31E-31	23	27	1.1	c.2.1.1
1llu_3	41	0.859	23	0.00E+00	3.68E-25	0.974	25	0.00E+00	6.20E-23	20	25	1.4	c.2.1.1
1llu_4	42	0.69	26	0.00E+00	2.92E-41	0.841	26	0.00E+00	1.31E-31	23	27	1.3	c.2.1.1
1llu_5	41	0.909	25	0.00E+00	3.73E-26	0.974	25	0.00E+00	6.20E-23	22	25	1.4	c.2.1.1
1llu_6	42	0.745	27	0.00E+00	1.21E-39	0.745	27	0.00E+00	1.21E-39	27	27	1.4	c.2.1.1
1llu_7	41	0.87	24	0.00E+00	2.07E-26	0.974	25	0.00E+00	6.20E-23	21	25	1.6	c.2.1.1
1o8c_0	120	0.923	22	0.00E+00	2.55E-19	0.923	22	0.00E+00	2.55E-19	22	27	3.9	c.2.1.1
1o8c_1	117	0.81	21	0.00E+00	1.51E-22	0.81	21	0.00E+00	1.51E-22	21	27	4.8	c.2.1.1
1kev_0	60	0.827	21	0.00E+00	4.37E-22	1.065	32	0.00E+00	9.36E-28	16	32	1.9	c.2.1.1
1kev_1	58	0.772	22	0.00E+00	6.48E-27	1.116	32	0.00E+00	6.44E-25	18	32	2.2	c.2.1.1
1kev_2	59	0.984	22	0.00E+00	4.68E-17	1.14	32	0.00E+00	1.39E-23	16	31	2.2	c.2.1.1
1kev_3	61	0.789	22	0.00E+00	3.11E-26	1.046	31	0.00E+00	1.90E-27	17	31	2	c.2.1.1
1ykf_0	36	1.057	20	4.97E-12	4.97E-12	0.863	18	1.22E-15	1.19E-15	14	20	1.5	c.2.1.1
1ykf_1	37	0.765	19	0.00E+00	1.06E-21	0.765	19	0.00E+00	1.06E-21	19	20	1.6	c.2.1.1
1ykf_2	35	1.051	20	2.81E-12	2.81E-12	0.932	18	4.03E-13	4.03E-13	14	19	1.4	c.2.1.1
1ykf_3	36	1.041	19	3.47E-11	3.47E-11	0.966	19	1.88E-13	1.88E-13	13	20	1.5	c.2.1.1
1pl8_4	26	0.825	20	0.00E+00	8.95E-22	0.792	19	0.00E+00	2.99E-21	16	19	0.6	c.2.1.1
1pl8_5	26	0.82	21	0.00E+00	6.57E-24	0.82	21	0.00E+00	6.57E-24	21	19	0.7	c.2.1.1
1pl8_6	27	0.807	20	0.00E+00	1.62E-22	0.807	20	0.00E+00	1.62E-22	20	19	7	c.2.1.1
1pl8_7	26	0.838	20	0.00E+00	3.26E-21	0.807	19	0.00E+00	1.29E-20	16	18	14.8	c.2.1.1
1pl6_2	36	0.823	20	0.00E+00	2.01E-21	0.915	20	0.00E+00	1.29E-17	16	20	0.7	c.2.1.1
1pl6_3	36	0.816	20	0.00E+00	9.92E-22	0.816	20	0.00E+00	9.92E-22	20	20	0.8	c.2.1.1
1pl6_4	37	0.812	20	0.00E+00	7.19E-22	0.812	20	0.00E+00	7.19E-22	20	20	0.9	c.2.1.1
1pl6_5	37	0.812	21	0.00E+00	8.31E-24	0.812	21	0.00E+00	8.31E-24	21	20	0.7	c.2.1.1
1kol_0	42	0.832	24	0.00E+00	9.34E-29	0.771	20	0.00E+00	1.46E-23	18	22	2.3	c.2.1.1
1kol_1	42	0.75	24	0.00E+00	3.42E-33	0.75	24	0.00E+00	3.42E-33	24	23	2.1	c.2.1.1
1qor_0	101	0.802	17	6.22E-15	6.26E-15	0.802	17	6.22E-15	6.26E-15	17	25	6.7	c.2.1.1
1qor_1	100	0.849	18	2.89E-15	2.87E-15	0.903	19	2.66E-15	2.71E-15	18	21	1.9	c.2.1.1
1iyz_0	37	1.219	13	1.00E+00	1.07E+03	1.033	11	1.00E+00	1.16E+01	9	16	3.4	c.2.1.1
1n9g_0	27	1.162	13	9.46E-01	2.92E+00	1.162	13	9.46E-01	2.92E+00	13	12	3.4	c.2.1.1
1n9g_1	35	1.103	15	3.24E-03	3.25E-03	1.103	15	3.24E-03	3.25E-03	15	17	3.1	c.2.1.1
1n9g_2	31	1.021	14	4.42E-04	4.42E-04	1.064	15	8.67E-05	8.67E-05	14	16	0.4	c.2.1.1
1guf_0	120	1.041	17	3.58E-07	3.58E-07	1.041	17	3.58E-07	3.58E-07	17	23	4.9	c.2.1.1
1guf_3	99	1.008	16	1.26E-07	1.26E-07	1.008	16	1.26E-07	1.26E-07	16	17	4.5	c.2.1.1
1guf_5	31	1.076	13	7.78E-02	8.10E-02	1.127	14	1.82E-02	1.84E-02	13	14	0.4	c.2.1.1
1piw_0	40	0.881	21	0.00E+00	4.16E-20	0.881	21	0.00E+00	4.16E-20	21	21	1.7	c.2.1.1
1piw_1	34	0.979	18	7.44E-12	7.44E-12	0.889	17	1.68E-13	1.68E-13	14	19	1.8	c.2.1.1
1ps0_0	38	1.017	20	2.23E-13	2.23E-13	1.017	20	2.23E-13	2.23E-13	20	19	1.7	c.2.1.1
1v3v_0	42	0.616	16	0.00E+00	1.21E-21	0.616	16	0.00E+00	1.21E-21	16	15	2	c.2.1.1
1v3v_1	44	0.999	16	3.71E-07	3.71E-07	1.059	18	5.89E-09	5.89E-09	16	20	1.3	c.2.1.1
1v3t_0	37	0.909	16	3.23E-10	3.23E-10	0.909	16	3.23E-10	3.23E-10	16	16	2.1	c.2.1.1
1v3t_1	38	0.984	16	8.32E-08	8.32E-08	1.054	18	2.63E-09	2.63E-09	16	20	1.2	c.2.1.1

Table 3: Results for alcohol dehydrogenase (1hdx_1) matching against its own SCOP family with amino acid property.

Site	N	RMSD	q	Pvalue	Evalue	RMSD	q	Pvalue	Evalue	CG	Mean L	Var L	SCOP
1het_0	109	0.449	52	0.00E+00	1.28E-137	0.447	57	0.00E+00	2.50E-151	51	57	0.6	c.2.1.1
1het_2	118	0.388	48	0.00E+00	2.64E-144	0.411	58	0.00E+00	7.53E-164	48	58	0.4	c.2.1.1
1heu_0	118	0.431	51	0.00E+00	2.37E-138	0.436	55	0.00E+00	1.99E-147	50	55	0.6	c.2.1.1
1heu_2	112	0.411	48	0.00E+00	7.49E-139	0.431	57	0.00E+00	2.67E-155	48	57	0.4	c.2.1.1
1mgo_0	37	0.425	29	0.00E+00	6.57E-73	0.437	33	0.00E+00	1.43E-81	29	33	0.2	c.2.1.1
1mgo_1	43	0.461	34	0.00E+00	1.39E-82	0.497	37	0.00E+00	2.42E-85	34	37	0.3	c.2.1.1
1ee2_0	48	0.428	33	0.00E+00	2.10E-85	0.442	36	0.00E+00	1.19E-91	33	36	0.1	c.2.1.1
1ee2_1	48	0.45	34	0.00E+00	2.08E-85	0.453	36	0.00E+00	6.00E-90	34	36	0.1	c.2.1.1
1ju9_4	20	0.331	13	0.00E+00	2.06E-26	0.453	16	0.00E+00	3.94E-29	13	16	0.4	c.2.1.1
1ju9_5	18	0.396	14	0.00E+00	4.01E-26	0.436	16	0.00E+00	2.35E-30	14	16	0.4	c.2.1.1
1qv6_0	44	0.456	36	0.00E+00	1.32E-89	0.459	39	0.00E+00	3.05E-97	36	39	0.3	c.2.1.1
1qv6_1	43	0.438	35	0.00E+00	1.35E-90	0.43	39	0.00E+00	3.69E-102	35	39	0.2	c.2.1.1
3bto_0	52	0.426	36	0.00E+00	1.30E-96	0.431	41	0.00E+00	2.21E-109	36	41	0.4	c.2.1.1
3bto_1	43	0.432	34	0.00E+00	3.02E-88	0.438	38	0.00E+00	1.60E-97	34	38	0.3	c.2.1.1
3bto_2	43	0.448	35	0.00E+00	4.65E-89	0.453	39	0.00E+00	2.94E-98	35	39	0.5	c.2.1.1
3bto_3	43	0.462	35	0.00E+00	5.77E-87	0.464	39	0.00E+00	1.84E-96	35	39	0.3	c.2.1.1
1qv7_0	42	0.458	35	0.00E+00	3.34E-86	0.423	37	0.00E+00	9.39E-97	34	38	0.3	c.2.1.1
1qv7_1	43	0.418	34	0.00E+00	2.04E-90	0.448	38	0.00E+00	7.03E-96	34	38	0.3	c.2.1.1
1mg0_0	42	0.428	35	0.00E+00	3.37E-92	0.435	38	0.00E+00	1.75E-99	35	38	0.2	c.2.1.1
1mg0_1	41	0.423	34	0.00E+00	1.07E-89	0.441	37	0.00E+00	2.91E-95	34	37	0.2	c.2.1.1
1mg0_2	42	0.419	35	0.00E+00	1.21E-93	0.429	38	0.00E+00	1.66E-100	35	38	0.2	c.2.1.1
1mg0_3	43	0.453	35	0.00E+00	2.64E-88	0.448	39	0.00E+00	4.34E-99	35	39	0.3	c.2.1.1
2ohx_0	117	0.36	51	0.00E+00	3.76E-156	0.361	56	0.00E+00	4.71E-171	50	56	0.6	c.2.1.1
2ohx_1	119	0.329	47	0.00E+00	3.09E-156	0.373	57	0.00E+00	4.14E-171	47	57	0.3	c.2.1.1
1hf3_0	120	0.386	47	0.00E+00	3.00E-141	0.367	55	0.00E+00	1.32E-165	46	56	0.3	c.2.1.1
1hf3_2	64	0.4	49	0.00E+00	1.21E-145	0.376	57	0.00E+00	4.79E-171	48	58	0.4	c.2.1.1
2oxi_0	116	0.376	47	0.00E+00	9.39E-144	0.398	57	0.00E+00	5.20E-164	47	57	0.3	c.2.1.1
2oxi_1	119	0.347	47	0.00E+00	3.04E-151	0.346	55	0.00E+00	7.65E-172	47	56	0.7	c.2.1.1
1axe_0	40	0.423	32	0.00E+00	4.60E-83	0.421	35	0.00E+00	6.31E-92	32	35	0.2	c.2.1.1
1axe_1	41	0.38	33	0.00E+00	1.19E-91	0.405	36	0.00E+00	1.98E-96	33	36	0.1	c.2.1.1
1a71_0	42	0.454	32	0.00E+00	3.02E-77	0.466	35	0.00E+00	1.14E-83	32	35	0.4	c.2.1.1
1a71_1	42	0.47	33	0.00E+00	2.99E-78	0.501	36	0.00E+00	7.74E-82	33	36	0.3	c.2.1.1
1bto_0	52	0.452	36	0.00E+00	1.87E-92	0.458	41	0.00E+00	1.43E-104	36	41	0.5	c.2.1.1
1bto_1	43	0.459	34	0.00E+00	2.99E-84	0.462	38	0.00E+00	1.22E-93	34	38	0.5	c.2.1.1
1bto_2	43	0.5	35	0.00E+00	1.38E-81	0.464	38	0.00E+00	2.51E-93	34	38	0.4	c.2.1.1
1bto_3	43	0.5	35	0.00E+00	1.38E-81	0.461	38	0.00E+00	8.46E-94	34	38	0.5	c.2.1.1
1hld_0	120	0.387	41	0.00E+00	7.26E-120	0.348	57	0.00E+00	1.00E-178	40	57	0.5	c.2.1.1
1hld_1	120	0.409	40	0.00E+00	5.48E-112	0.36	57	0.00E+00	5.35E-175	39	57	0.4	c.2.1.1
1qlh_0	45	3.69	20	1.00E+00	3.04E+36	0.791	26	0.00E+00	2.88E-35	19	27	4.2	c.2.1.1
1lde_0	51	0.443	35	0.00E+00	1.17E-90	0.445	40	0.00E+00	1.18E-103	35	40	0.4	c.2.1.1
1lde_1	41	0.449	31	0.00E+00	3.77E-76	0.452	35	0.00E+00	1.01E-85	31	35	0.3	c.2.1.1
1lde_2	39	0.43	32	0.00E+00	1.20E-80	0.433	35	0.00E+00	1.23E-88	32	35	0.4	c.2.1.1
1lde_3	40	0.439	32	0.00E+00	2.36E-79	0.448	36	0.00E+00	1.48E-89	32	36	0.4	c.2.1.1
1axg_0	44	0.471	35	0.00E+00	2.92E-84	0.48	38	0.00E+00	7.86E-91	35	38	0.5	c.2.1.1
1axg_1	42	0.467	34	0.00E+00	8.96E-82	0.494	38	0.00E+00	8.40E-89	34	38	0.4	c.2.1.1
1axg_2	44	0.514	35	0.00E+00	2.16E-78	0.513	38	0.00E+00	5.38E-86	35	38	0.5	c.2.1.1
1axg_3	42	0.434	34	0.00E+00	1.54E-86	0.43	37	0.00E+00	1.35E-95	34	37	0.4	c.2.1.1
1ldy_0	52	0.458	37	0.00E+00	1.08E-94	0.42	41	0.00E+00	1.99E-111	36	41	0.4	c.2.1.1
1ldy_1	42	0.463	34	0.00E+00	1.04E-83	0.421	37	0.00E+00	4.35E-97	33	37	0.3	c.2.1.1
1ldy_2	42	0.476	34	0.00E+00	6.93E-82	0.438	37	0.00E+00	2.71E-94	33	37	0.4	c.2.1.1
1ldy_3	42	0.46	34	0.00E+00	9.33E-83	0.431	37	0.00E+00	1.97E-95	33	37	0.3	c.2.1.1
6adh_0	120	0.431	44	0.00E+00	4.93E-121	0.523	56	0.00E+00	4.62E-131	44	56	0.7	c.2.1.1
6adh_1	120	0.544	49	0.00E+00	9.91E-110	0.581	56	0.00E+00	1.00E-119	49	55	1	c.2.1.1
1ht0_0	38	0.302	30	0.00E+00	8.73E-96	0.307	34	0.00E+00	1.87E-107	30	34	0.1	c.2.1.1
1ht0_1	38	0.346	30	0.00E+00	2.34E-86	0.358	33	0.00E+00	6.66E-94	30	33	0.1	c.2.1.1
1deh_1	60	0.283	56	0.00E+00	3.56E-198	0.286	57	0.00E+00	3.29E-201	56	57	0.2	c.2.1.1
1deh_3	59	0.285	47	0.00E+00	1.28E-170	0.299	56	0.00E+00	2.87E-192	47	56	0.2	c.2.1.1
1mp0_9	36	0.499	18	0.00E+00	2.99E-30	0.551	22	0.00E+00	9.89E-36	18	22	0.5	c.2.1.1
1mp0_10	36	0.56	19	0.00E+00	2.47E-29	0.549	23	0.00E+00	1.20E-38	18	23	0.7	c.2.1.1
1hsz_0	39	0.306	32	0.00E+00	5.51E-102	0.311	36	0.00E+00	1.65E-113	32	36	0.1	c.2.1.1
1hsz_1	38	0.316	34	0.00E+00	3.03E-106	0.314	36	0.00E+00	6.92E-113	34	36	0.1	c.2.1.1
1htb_1	60	0.291	55	0.00E+00	4.91E-191	0.266	55	0.00E+00	1.59E-200	54	56	0.3	c.2.1.1
1htb_3	58	0.332	46	0.00E+00	1.75E-152	0.357	55	0.00E+00	1.75E-169	46	55	0.2	c.2.1.1
1ma0_0	47	0.616	21	0.00E+00	6.08E-32	0.635	27	0.00E+00	3.12E-42	20	27	0.7	c.2.1.1
1ma0_1	47	0.564	24	0.00E+00	2.54E-42	0.541	27	0.00E+00	4.13E-50	23	27	0.5	c.2.1.1
1hdy_1	59	1.791	53	1.00E+00	8.76E+01	0.255	53	0.00E+00	4.52E-198	51	54	0.3	c.2.1.1
1hdy_3	58	0.307	46	0.00E+00	1.18E-159	0.274	54	0.00E+00	3.54E-193	45	54	0.3	c.2.1.1
1hdx_1	60	0	60	0.00E+00	0.00E+00	0	60	0.00E+00	0.00E+00	60	60	0	c.2.1.1
1hdx_3	59	0.258	51	0.00E+00	1.25E-195	0.254	58	0.00E+00	1.06E-218	51	58	0	c.2.1.1

Table 4: Results for alcohol dehydrogenase (1hdx_1) matching against its own SCOP family with amino acid property.

Site	N	RMSD	q	Pvalue	Evalue	RMSD	q	Pvalue	Evalue	CG	Mean L	Var L	SCOP
1hdz_1	58	0.314	53	0.00E+00	4.21E-175	0.32	54	0.00E+00	4.10E-177	53	54	0.1	c.2.1.1
1hdz_3	56	0.333	46	0.00E+00	2.97E-152	0.34	53	0.00E+00	9.94E-169	46	53	0.1	c.2.1.1
1d1t_0	38	0.475	29	0.00E+00	1.14E-65	0.466	33	0.00E+00	1.73E-76	29	33	0.2	c.2.1.1
1d1t_1	39	0.46	29	0.00E+00	5.03E-66	0.406	32	0.00E+00	1.40E-81	28	33	0.4	c.2.1.1
1d1t_2	39	0.441	28	0.00E+00	1.72E-66	0.463	33	0.00E+00	7.46E-77	28	33	0.2	c.2.1.1
1d1t_3	38	0.414	28	0.00E+00	7.05E-70	0.406	32	0.00E+00	1.29E-81	28	32	0.2	c.2.1.1
1mc5_0	51	0.491	26	0.00E+00	2.08E-54	0.513	30	0.00E+00	2.24E-61	26	30	0.2	c.2.1.1
1mc5_8	35	0.524	20	0.00E+00	3.20E-33	0.558	23	0.00E+00	5.05E-38	20	23	0.5	c.2.1.1
1d1s_0	38	0.431	27	0.00E+00	1.89E-64	0.448	32	0.00E+00	1.01E-75	27	32	0.2	c.2.1.1
1d1s_1	40	0.457	29	0.00E+00	9.82E-68	0.446	33	0.00E+00	3.86E-79	29	33	0.3	c.2.1.1
1d1s_2	40	0.435	29	0.00E+00	5.01E-69	0.379	32	0.00E+00	1.15E-85	28	33	0.3	c.2.1.1
1d1s_3	40	0.479	28	0.00E+00	4.51E-62	0.462	32	0.00E+00	8.26E-74	28	32	0.4	c.2.1.1
1teh_1	58	0.776	25	0.00E+00	7.01E-30	0.863	41	0.00E+00	1.51E-51	25	40	0.9	c.2.1.1
1teh_3	56	0.68	26	0.00E+00	1.83E-39	0.876	40	0.00E+00	1.16E-48	26	39	1.2	c.2.1.1
1hso_0	41	0.367	34	0.00E+00	4.06E-100	0.365	37	0.00E+00	7.67E-110	34	37	0.1	c.2.1.1
1hso_1	35	0.304	27	0.00E+00	1.18E-81	0.32	31	0.00E+00	4.12E-93	27	31	0.1	c.2.1.1
3hud_1	59	1.82	54	1.00E+00	3.66E+03	0.352	55	0.00E+00	5.95E-171	53	55	0.1	c.2.1.1
3hud_3	56	0.436	48	0.00E+00	4.18E-134	0.409	55	0.00E+00	3.65E-155	47	56	0.4	c.2.1.1
1agn_1	58	0.424	41	0.00E+00	1.94E-113	0.415	51	0.00E+00	4.39E-140	41	51	0.2	c.2.1.1
1agn_3	59	0.383	43	0.00E+00	9.83E-129	0.386	52	0.00E+00	1.27E-150	43	52	0.2	c.2.1.1
1agn_5	40	0.403	29	0.00E+00	1.13E-74	0.398	33	0.00E+00	3.35E-86	29	33	0.3	c.2.1.1
1agn_6	39	0.474	28	0.00E+00	1.16E-62	0.421	31	0.00E+00	1.59E-77	27	32	0.8	c.2.1.1
1e3i_0	120	0.626	27	0.00E+00	4.20E-46	0.692	40	0.00E+00	1.60E-68	27	39	1	c.2.1.1
1e3i_1	115	0.741	14	1.90E-11	1.90E-11	0.635	26	0.00E+00	8.62E-41	13	26	3.7	c.2.1.1
1e3i_2	44	0.681	21	0.00E+00	3.46E-29	0.637	27	0.00E+00	1.99E-45	20	27	0.7	c.2.1.1
1e3e_0	120	0.652	25	0.00E+00	3.37E-39	0.735	36	0.00E+00	3.24E-54	25	36	0.9	c.2.1.1
1e3l_0	118	0.58	29	0.00E+00	2.16E-55	0.588	38	0.00E+00	9.10E-75	29	38	2.1	c.2.1.1
1p0f_0	41	0.601	25	0.00E+00	3.93E-40	0.643	31	0.00E+00	1.76E-50	25	31	0.6	c.2.1.1
1p0f_1	41	0.645	26	0.00E+00	2.88E-39	0.662	31	0.00E+00	8.44E-49	26	31	0.6	c.2.1.1
1cdo_0	60	0.487	28	0.00E+00	6.35E-61	0.494	39	0.00E+00	1.46E-86	28	39	0.5	c.2.1.1
1r37_0	66	0.806	25	0.00E+00	6.11E-28	0.908	30	0.00E+00	2.37E-30	25	29	1	c.2.1.1
1r37_2	66	0.801	24	0.00E+00	3.64E-27	0.902	30	0.00E+00	1.01E-30	24	29	0.9	c.2.1.1
1llu_0	42	0.722	20	0.00E+00	7.55E-26	0.801	24	0.00E+00	5.43E-29	20	24	0.7	c.2.1.1
1llu_1	41	0.796	18	0.00E+00	2.48E-18	0.806	21	0.00E+00	6.88E-23	18	21	1	c.2.1.1
1llu_2	42	0.723	20	0.00E+00	8.49E-26	0.738	23	0.00E+00	1.71E-30	20	24	0.8	c.2.1.1
1llu_3	41	0.796	18	0.00E+00	2.48E-18	0.806	21	0.00E+00	6.88E-23	18	21	0.9	c.2.1.1
1llu_4	42	0.722	20	0.00E+00	7.55E-26	0.801	24	0.00E+00	5.43E-29	20	24	0.8	c.2.1.1
1llu_5	41	0.82	19	0.00E+00	2.78E-19	0.806	21	0.00E+00	6.88E-23	19	21	1.1	c.2.1.1
1llu_6	42	0.722	20	0.00E+00	7.55E-26	0.738	23	0.00E+00	1.71E-30	20	24	0.9	c.2.1.1
1llu_7	41	0.82	19	0.00E+00	2.78E-19	0.806	21	0.00E+00	6.88E-23	19	21	1	c.2.1.1
1o8c_0	120	0.881	11	1.00E+00	4.35E+02	1.236	16	1.00E+00	2.71E+04	11	21	4.9	c.2.1.1
1o8c_1	117	0.64	11	1.25E-02	1.26E-02	0.64	11	1.25E-02	1.26E-02	11	21	5.4	c.2.1.1
1kev_0	60	0.696	14	4.84E-13	4.84E-13	1.019	28	0.00E+00	1.51E-22	14	28	1.5	c.2.1.1
1kev_1	58	1.113	14	1.04E-02	1.05E-02	1.114	28	0.00E+00	7.30E-18	13	29	2.1	c.2.1.1
1kev_2	59	0.83	14	1.79E-10	1.79E-10	1.089	28	0.00E+00	4.81E-19	14	28	2.2	c.2.1.1
1kev_3	61	0.767	15	1.85E-12	1.85E-12	1.035	28	0.00E+00	1.43E-19	15	28	1.9	c.2.1.1
1ykf_0	36	0.677	10	3.70E-06	3.70E-06	0.866	16	8.23E-10	8.23E-10	10	17	1	c.2.1.1
1ykf_1	37	0.646	10	6.43E-06	6.43E-06	0.898	16	1.02E-08	1.02E-08	10	17	1	c.2.1.1
1ykf_2	35	0.562	10	2.73E-07	2.73E-07	0.562	10	2.73E-07	2.73E-07	10	16	0.8	c.2.1.1
1ykf_3	36	0.647	10	6.30E-06	6.30E-06	0.75	14	8.80E-10	8.80E-10	10	16	7	c.2.1.1
1pl8_4	26	0.771	11	2.16E-03	2.16E-03	0.773	15	1.19E-09	1.19E-09	11	15	0.5	c.2.1.1
1pl8_5	26	0.779	12	3.15E-05	3.15E-05	0.77	15	9.50E-10	9.50E-10	12	15	2.1	c.2.1.1
1pl8_6	27	0.78	12	3.77E-05	3.77E-05	0.774	15	1.44E-09	1.44E-09	12	15	0.6	c.2.1.1
1pl8_7	26	0.786	11	4.87E-03	4.88E-03	0.69	14	3.14E-10	3.14E-10	10	14	11.4	c.2.1.1
1pl6_2	36	0.748	11	1.56E-05	1.56E-05	0.748	11	1.56E-05	1.56E-05	11	16	1.4	c.2.1.1
1pl6_3	36	1.57	12	1.00E+00	2.13E+10	0.992	15	6.37E-03	6.39E-03	10	17	3.6	c.2.1.1
1pl6_4	37	1.558	12	1.00E+00	1.61E+10	0.993	15	7.35E-03	7.37E-03	10	17	3.8	c.2.1.1
1pl6_5	37	1.569	12	1.00E+00	2.24E+10	0.967	16	4.32E-05	4.32E-05	11	16	0.9	c.2.1.1
1kol_0	42	0.736	17	1.90E-14	1.90E-14	0.703	20	0.00E+00	1.46E-21	16	21	1.1	c.2.1.1
1kol_1	42	0.645	17	0.00E+00	1.86E-18	0.698	20	0.00E+00	8.06E-22	17	21	1.1	c.2.1.1
1qor_0	101	1.341	10	1.00E+00	1.77E+11	1.341	10	1.00E+00	1.77E+11	10	11	1	c.2.1.1
1qor_1	100	1.353	10	1.00E+00	2.39E+11	1.353	10	1.00E+00	2.39E+11	10	11	1	c.2.1.1
1iyz_0	37	1.638	8	1.00E+00	1.58E+13	1.057	10	1.00E+00	9.66E+04	5	11	1.9	c.2.1.1
1n9g_0	27	4.893	8	1.00E+00	9.75E+26	4.893	8	1.00E+00	9.75E+26	8	11	0.7	c.2.1.1
1n9g_1	35	3.619	7	1.00E+00	3.94E+22	3.619	7	1.00E+00	3.94E+22	7	10	2.8	c.2.1.1
1n9g_2	31	3.429	7	1.00E+00	3.81E+22	3.429	7	1.00E+00	3.81E+22	7	8	0.8	c.2.1.1
1guf_0	120	5.236	9	1.00E+00	8.99E+31	5.236	9	1.00E+00	8.99E+31	9	12	1.7	c.2.1.1
1guf_3	99	4.649	8	1.00E+00	5.10E+29	4.649	8	1.00E+00	5.10E+29	8	10	1.3	c.2.1.1
1guf_5	31	1.319	7	1.00E+00	4.32E+11	1.431	8	1.00E+00	1.52E+12	7	8	0.3	c.2.1.1
1piw_0	40	0.953	17	1.32E-10	1.32E-10	0.844	20	0.00E+00	2.99E-18	16	20	1	c.2.1.1
1piw_1	34	0.742	13	5.03E-10	5.03E-10	0.789	16	7.82E-12	7.82E-12	13	19	2.9	c.2.1.1
1ps0_0	38	0.751	14	1.12E-09	1.12E-09	0.817	17	1.34E-14	1.34E-14	14	18	0.9	c.2.1.1
1v3v_0	42	3.647	8	1.00E+00	6.10E+23	3.647	8	1.00E+00	6.10E+23	8	9	0.8	c.2.1.1
1v3v_1	44	2.6	9	1.00E+00	3.95E+20	2.6	9	1.00E+00	3.95E+20	9	11	1	c.2.1.1
1v3t_0	37	3.167	8	1.00E+00	7.30E+20	3.167	8	1.00E+00	7.30E+20	8	9	0.5	c.2.1.1
1v3t_1	38	2.607	9	1.00E+00	2.77E+20	0.84	10	8.12E-01	1.67E+00	0	12	1.8	c.2.1.1
