# Supplementary material for: Bayesian refinement of protein functional site matching
Source: BMC Bioinformatics. 2007 Jul 17;8:257. doi: 10.1186/1471-2105-8-257 (PMC1940029; doi:10.1186/1471-2105-8-257)
Supplement: Additional file 3 — Case 3 Results. Results for alcohol dehydrogenase (1hdx_1) and superfamily. Tables 1–14: Without physico-chemistry. Tables 14–28: With physico-chemistry. [file 1471-2105-8-257-S3.pdf]

Results for alcohol dehydrogenase (1hdx_1) and superfamily.

Table 1: Results for alcohol dehydrogenase matching against its own SCOP superfamily (but not family) without amino acid property.

Site	N	RMSD	q	Pvalue	Evalue	RMSD	q	Pvalue	Evalue	CG	Mean L	Var L	SCOP
1udb_0	38	1.924	12	1.00E+00	1.74E+13	1.889	13	1.00E+00	6.71E+11	12	14	1.0	c.2.1.2
1udc_0	58	2.204	12	1.00E+00	4.32E+17	2.204	12	1.00E+00	4.32E+17	12	13	0.6	c.2.1.2
1xel_0	57	1.454	12	1.00E+00	5.13E+08	1.447	13	1.00E+00	1.70E+07	12	14	0.5	c.2.1.2
1lrk_2	34	4.726	11	1.00E+00	5.12E+29	4.726	11	1.00E+00	5.12E+29	11	13	0.9	c.2.1.2
1nah_0	39	1.912	12	1.00E+00	1.40E+13	1.896	13	1.00E+00	8.75E+11	12	14	0.8	c.2.1.2
2udp_0	54	6.254	12	1.00E+00	7.27E+39	6.254	12	1.00E+00	7.27E+39	12	13	1.0	c.2.1.2
2udp_1	52	1.858	12	1.00E+00	6.84E+13	1.884	13	1.00E+00	1.24E+13	12	13	0.4	c.2.1.2
1uda_0	37	1.926	12	1.00E+00	1.68E+13	1.890	13	1.00E+00	6.34E+11	12	14	1.0	c.2.1.2
1a9y_0	53	3.570	13	1.00E+00	4.08E+27	3.570	13	1.00E+00	4.08E+27	13	14	0.9	c.2.1.2
1lrl_2	33	5.759	11	1.00E+00	1.52E+35	5.759	11	1.00E+00	1.52E+35	11	13	0.8	c.2.1.2
1kvu_0	58	3.810	12	1.00E+00	7.36E+30	3.810	12	1.00E+00	7.36E+30	12	15	1.2	c.2.1.2
1kvr_0	38	1.916	12	1.00E+00	1.43E+13	1.897	13	1.00E+00	8.29E+11	12	14	0.9	c.2.1.2
1a9z_0	33	1.945	12	1.00E+00	1.88E+13	1.907	13	1.00E+00	6.98E+11	12	14	0.8	c.2.1.2
1lrj_0	53	1.468	12	1.00E+00	6.58E+08	1.452	13	1.00E+00	1.63E+07	12	13	0.2	c.2.1.2
1nai_0	37	6.564	11	1.00E+00	7.16E+37	6.564	11	1.00E+00	7.16E+37	11	12	1.0	c.2.1.2
1kvt_0	52	1.946	12	1.00E+00	7.80E+13	1.908	13	1.00E+00	2.90E+12	12	14	0.9	c.2.1.2
1kvq_0	52	1.771	12	1.00E+00	6.44E+12	1.805	13	1.00E+00	1.32E+12	12	13	0.4	c.2.1.2
1kvs_0	52	1.774	12	1.00E+00	9.81E+11	1.774	12	1.00E+00	9.81E+11	12	12	0.3	c.2.1.2
1i3k_0	52	1.361	12	1.00E+00	3.55E+06	1.361	12	1.00E+00	3.55E+06	12	12	0.4	c.2.1.2
1i3k_1	52	4.509	12	1.00E+00	2.97E+34	4.509	12	1.00E+00	2.97E+34	12	14	1.1	c.2.1.2
1i3l_6	32	4.476	11	1.00E+00	1.93E+30	4.476	11	1.00E+00	1.93E+30	11	13	0.8	c.2.1.2
1i3l_7	33	5.942	11	1.00E+00	6.08E+35	5.942	11	1.00E+00	6.08E+35	11	12	0.8	c.2.1.2
1i3n_0	54	5.469	12	1.00E+00	6.64E+38	5.469	12	1.00E+00	6.64E+38	12	14	1.2	c.2.1.2
1i3n_6	33	4.472	11	1.00E+00	2.04E+30	4.472	11	1.00E+00	2.04E+30	11	13	0.8	c.2.1.2
1i3m_0	54	5.575	12	1.00E+00	1.78E+39	5.575	12	1.00E+00	1.78E+39	12	14	1.1	c.2.1.2
1i3m_6	32	1.471	11	1.00E+00	7.12E+08	1.471	11	1.00E+00	7.12E+08	11	11	0.2	c.2.1.2
1hzj_0	54	6.208	12	1.00E+00	5.05E+39	6.208	12	1.00E+00	5.05E+39	12	14	1.1	c.2.1.2
1hzj_1	55	1.470	12	1.00E+00	1.61E+08	1.470	12	1.00E+00	1.61E+08	12	13	0.8	c.2.1.2
1ek5_0	36	1.167	12	1.00E+00	7.98E+02	1.167	12	1.00E+00	7.98E+02	12	13	0.8	c.2.1.2
1gy8_0	120	1.962	14	1.00E+00	1.34E+14	1.962	14	1.00E+00	1.34E+14	14	14	0.3	c.2.1.2
1bxk_0	33	1.669	11	1.00E+00	2.12E+11	1.669	11	1.00E+00	2.12E+11	11	11	0.2	c.2.1.2
1bxk_1	36	6.262	12	1.00E+00	2.23E+39	6.262	12	1.00E+00	2.23E+39	12	13	0.8	c.2.1.2
1g1a_0	38	4.487	12	1.00E+00	1.94E+32	4.487	12	1.00E+00	1.94E+32	12	13	0.7	c.2.1.2
1g1a_1	37	4.480	12	1.00E+00	1.65E+32	4.480	12	1.00E+00	1.65E+32	12	13	0.7	c.2.1.2
1g1a_2	37	1.587	13	1.00E+00	1.83E+10	1.587	13	1.00E+00	1.83E+10	13	13	0.0	c.2.1.2
1g1a_3	37	4.465	12	1.00E+00	1.40E+32	4.465	12	1.00E+00	1.40E+32	12	13	0.7	c.2.1.2
1kep_0	45	5.402	12	1.00E+00	1.33E+40	5.402	12	1.00E+00	1.33E+40	12	13	1.2	c.2.1.2
1kep_1	58	4.634	14	1.00E+00	1.33E+37	4.634	14	1.00E+00	1.33E+37	14	16	1.3	c.2.1.2
1ket_2	38	5.616	12	1.00E+00	1.72E+35	5.616	12	1.00E+00	1.72E+35	12	14	1.0	c.2.1.2
1ket_3	39	7.687	13	1.00E+00	3.91E+44	7.687	13	1.00E+00	3.91E+44	13	14	1.0	c.2.1.2
1kew_2	39	6.153	12	1.00E+00	1.03E+41	6.153	12	1.00E+00	1.03E+41	12	13	0.7	c.2.1.2
1kew_3	38	6.155	12	1.00E+00	9.67E+40	6.155	12	1.00E+00	9.67E+40	12	13	0.7	c.2.1.2
1ker_0	59	3.896	12	1.00E+00	2.06E+28	3.831	13	1.00E+00	6.77E+27	12	14	1.3	c.2.1.2
1ker_1	61	5.729	13	1.00E+00	4.47E+36	5.729	13	1.00E+00	4.47E+36	13	14	0.9	c.2.1.2
1keu_0	58	1.672	12	1.00E+00	5.28E+11	1.662	13	1.00E+00	2.48E+10	12	14	1.0	c.2.1.2
1keu_1	57	1.682	12	1.00E+00	6.72E+11	1.852	13	1.00E+00	6.70E+12	12	14	0.8	c.2.1.2
1r6d_0	57	1.571	13	1.00E+00	6.96E+09	1.568	14	1.00E+00	3.34E+08	13	14	0.3	c.2.1.2
1r66_1	38	5.539	12	1.00E+00	8.96E+34	5.539	12	1.00E+00	8.96E+34	12	14	1.0	c.2.1.2
1kc1_2	27	4.673	10	1.00E+00	5.89E+30	4.673	10	1.00E+00	5.89E+30	10	11	0.6	c.2.1.2
1kc3_0	39	5.480	11	1.00E+00	2.81E+34	5.480	11	1.00E+00	2.81E+34	11	13	1.0	c.2.1.2
1e6u_0	120	6.485	14	1.00E+00	5.45E+44	6.485	14	1.00E+00	5.45E+44	14	17	1.9	c.2.1.2
1e7s_0	120	5.538	15	1.00E+00	1.41E+41	5.538	15	1.00E+00	1.41E+41	15	17	1.1	c.2.1.2
1e7q_0	120	2.282	14	1.00E+00	5.57E+17	2.282	14	1.00E+00	5.57E+17	14	16	1.6	c.2.1.2
1e7r_0	120	1.879	14	1.00E+00	1.24E+13	1.899	15	1.00E+00	2.07E+12	14	17	1.5	c.2.1.2
1bsv_0	29	5.089	11	1.00E+00	7.19E+30	5.089	11	1.00E+00	7.19E+30	11	12	0.8	c.2.1.2
1fxs_0	29	1.248	11	1.00E+00	1.01E+05	1.248	11	1.00E+00	1.01E+05	11	11	0.1	c.2.1.2
1bws_0	29	1.518	11	1.00E+00	5.77E+10	1.518	11	1.00E+00	5.77E+10	11	11	0.1	c.2.1.2
1rpn_0	40	1.536	12	1.00E+00	2.58E+09	1.536	12	1.00E+00	2.58E+09	12	13	0.6	c.2.1.2
1rpn_1	40	1.575	12	1.00E+00	1.58E+09	1.575	12	1.00E+00	1.58E+09	12	13	1.0	c.2.1.2
1rpn_2	39	1.478	12	1.00E+00	1.78E+09	1.478	12	1.00E+00	1.78E+09	12	12	0.0	c.2.1.2
1rpn_3	39	1.558	12	1.00E+00	4.82E+09	1.558	12	1.00E+00	4.82E+09	12	12	0.4	c.2.1.2
1t2a_0	40	1.543	11	1.00E+00	1.18E+10	1.543	11	1.00E+00	1.18E+10	11	11	0.4	c.2.1.2
1t2a_2	39	1.680	12	1.00E+00	3.09E+10	1.708	13	1.00E+00	4.62E+09	12	14	0.6	c.2.1.2
1t2a_4	39	1.683	12	1.00E+00	3.37E+10	1.702	13	1.00E+00	3.87E+09	12	14	0.5	c.2.1.2
1t2a_6	39	1.526	11	1.00E+00	1.27E+09	1.526	11	1.00E+00	1.27E+09	11	12	0.6	c.2.1.2

Table 2: Results for alcohol dehydrogenase matching against its own SCOP superfamily (but not family) without amino acid property.

Site	N	RMSD	q	Pvalue	Evalue	RMSD	q	Pvalue	Evalue	CG	Mean L	Var L	SCOP
1n7h_0	35	1.657	11	1.00E+00	1.85E+11	1.657	11	1.00E+00	1.85E+11	11	11	0.4	c.2.1.2
1n7h_2	35	1.652	11	1.00E+00	1.61E+11	1.652	11	1.00E+00	1.61E+11	11	11	0.3	c.2.1.2
1n7g_0	58	8.502	13	1.00E+00	6.83E+51	8.502	13	1.00E+00	6.83E+51	13	15	1.5	c.2.1.2
1n7g_1	57	2.499	12	1.00E+00	2.83E+21	2.499	12	1.00E+00	2.83E+21	12	14	1.6	c.2.1.2
1n7g_2	58	1.628	12	1.00E+00	8.52E+11	1.628	12	1.00E+00	8.52E+11	12	12	0.1	c.2.1.2
1n7g_3	37	2.484	12	1.00E+00	3.94E+19	2.484	12	1.00E+00	3.94E+19	12	13	1.0	c.2.1.2
1eq2_0	36	2.823	11	1.00E+00	6.65E+22	2.823	11	1.00E+00	6.65E+22	11	13	0.9	c.2.1.2
1eq2_1	54	1.182	12	1.00E+00	1.60E+04	1.245	13	1.00E+00	5.62E+03	12	13	0.3	c.2.1.2
1eq2_2	36	1.892	11	1.00E+00	5.85E+14	1.892	11	1.00E+00	5.85E+14	11	11	0.3	c.2.1.2
1eq2_3	54	7.485	13	1.00E+00	3.36E+46	7.485	13	1.00E+00	3.36E+46	13	15	1.2	c.2.1.2
1eq2_4	36	3.138	12	1.00E+00	8.12E+25	3.138	12	1.00E+00	8.12E+25	12	14	0.9	c.2.1.2
1eq2_5	36	2.824	11	1.00E+00	6.76E+22	2.824	11	1.00E+00	6.76E+22	11	13	0.8	c.2.1.2
1eq2_6	36	1.740	11	1.00E+00	2.56E+11	1.740	11	1.00E+00	2.56E+11	11	11	0.0	c.2.1.2
1eq2_7	36	2.832	11	1.00E+00	7.70E+22	2.832	11	1.00E+00	7.70E+22	11	13	0.9	c.2.1.2
1eq2_8	36	1.663	11	1.00E+00	9.28E+12	1.640	12	1.00E+00	2.87E+11	11	12	0.3	c.2.1.2
1eq2_9	36	1.749	11	1.00E+00	3.19E+11	1.749	11	1.00E+00	3.19E+11	11	11	0.0	c.2.1.2
1orr_0	35	1.876	11	1.00E+00	5.69E+12	1.843	12	1.00E+00	2.29E+11	11	13	0.5	c.2.1.2
1orr_2	34	2.795	11	1.00E+00	1.98E+21	2.795	11	1.00E+00	1.98E+21	11	13	0.8	c.2.1.2
1orr_4	34	2.798	11	1.00E+00	2.08E+21	2.798	11	1.00E+00	2.08E+21	11	13	0.7	c.2.1.2
1orr_6	35	4.987	11	1.00E+00	3.08E+32	4.987	11	1.00E+00	3.08E+32	11	13	0.9	c.2.1.2
1i24_0	58	1.799	12	1.00E+00	1.43E+14	1.799	12	1.00E+00	1.43E+14	12	13	0.5	c.2.1.2
1qrr_0	58	0.866	12	1.52E-03	1.52E-03	0.866	12	1.52E-03	1.52E-03	12	13	0.7	c.2.1.2
1i2c_0	58	0.886	12	4.26E-03	4.27E-03	0.886	12	4.26E-03	4.27E-03	12	13	0.7	c.2.1.2
1i2b_0	59	1.301	12	1.00E+00	1.61E+05	1.301	12	1.00E+00	1.61E+05	12	13	0.4	c.2.1.2
1k6x_0	27	1.885	11	1.00E+00	2.02E+14	1.908	12	1.00E+00	3.35E+13	11	12	0.4	c.2.1.2
1ti7_0	33	4.684	11	1.00E+00	7.92E+32	4.684	11	1.00E+00	7.92E+32	11	12	0.6	c.2.1.2
1cyd_0	40	1.464	12	1.00E+00	2.43E+08	0.861	11	3.99E-02	4.07E-02	2	12	3.6	c.2.1.2
1cyd_1	40	1.464	12	1.00E+00	2.43E+08	0.852	11	2.58E-02	2.61E-02	2	12	2.9	c.2.1.2
1cyd_2	40	1.453	12	1.00E+00	1.67E+08	0.677	8	1.00E+00	1.47E+01	1	12	4.5	c.2.1.2
1cyd_3	40	7.970	12	1.00E+00	4.48E+44	7.970	12	1.00E+00	4.48E+44	12	14	1.2	c.2.1.2
1pr9_0	41	2.457	12	1.00E+00	2.31E+18	2.457	12	1.00E+00	2.31E+18	12	12	0.4	c.2.1.2
1pr9_1	42	5.641	13	1.00E+00	3.41E+39	5.641	13	1.00E+00	3.41E+39	13	15	1.2	c.2.1.2
1oaa_0	39	3.284	12	1.00E+00	4.38E+25	3.284	12	1.00E+00	4.38E+25	12	13	0.9	c.2.1.2
1sep_0	43	6.756	12	1.00E+00	1.68E+43	6.756	12	1.00E+00	1.68E+43	12	15	1.1	c.2.1.2
1nas_0	46	1.527	13	1.00E+00	4.09E+09	1.571	14	1.00E+00	1.08E+09	13	14	0.5	c.2.1.2
1dhr_0	31	2.686	10	1.00E+00	3.70E+18	2.686	10	1.00E+00	3.70E+18	10	11	0.7	c.2.1.2
1dir_0	33	3.256	11	1.00E+00	1.58E+24	3.256	11	1.00E+00	1.58E+24	11	12	0.9	c.2.1.2
1dir_1	36	1.387	13	1.00E+00	4.55E+05	1.410	15	1.00E+00	1.63E+03	13	15	0.2	c.2.1.2
1dir_2	33	1.406	13	1.00E+00	7.07E+05	0.650	8	9.29E-01	2.64E+00	5	10	2.5	c.2.1.2
1dir_3	32	3.443	11	1.00E+00	2.36E+22	3.443	11	1.00E+00	2.36E+22	11	12	0.8	c.2.1.2
1hdr_0	30	2.291	10	1.00E+00	7.31E+14	2.291	10	1.00E+00	7.31E+14	10	11	0.8	c.2.1.2
1e7w_0	44	1.474	11	1.00E+00	1.02E+10	1.474	11	1.00E+00	1.02E+10	11	11	0.1	c.2.1.2
1e7w_1	43	7.409	12	1.00E+00	1.91E+45	7.409	12	1.00E+00	1.91E+45	12	14	1.1	c.2.1.2
1e92_0	38	1.811	12	1.00E+00	7.39E+12	1.811	12	1.00E+00	7.39E+12	12	12	0.2	c.2.1.2
1e92_1	38	1.962	11	1.00E+00	3.73E+15	1.962	11	1.00E+00	3.73E+15	11	11	0.1	c.2.1.2
1e92_2	37	5.026	11	1.00E+00	2.96E+34	5.026	11	1.00E+00	2.96E+34	11	13	1.0	c.2.1.2
1e92_3	38	4.197	12	1.00E+00	7.19E+30	4.197	12	1.00E+00	7.19E+30	12	13	0.8	c.2.1.2
1p33_0	42	1.324	11	1.00E+00	6.14E+07	1.324	11	1.00E+00	6.14E+07	11	11	0.3	c.2.1.2
1p33_1	42	4.575	11	1.00E+00	2.65E+34	4.575	11	1.00E+00	2.65E+34	11	14	1.3	c.2.1.2
1p33_2	42	6.311	11	1.00E+00	1.67E+39	6.311	11	1.00E+00	1.67E+39	11	13	1.1	c.2.1.2
1p33_3	42	1.939	12	1.00E+00	2.11E+16	1.939	12	1.00E+00	2.11E+16	12	12	0.0	c.2.1.2
1mxh_0	42	1.509	12	1.00E+00	1.25E+09	1.509	12	1.00E+00	1.25E+09	12	12	0.3	c.2.1.2
1mxh_1	42	1.528	13	1.00E+00	6.06E+08	1.528	13	1.00E+00	6.06E+08	13	13	0.2	c.2.1.2
1mxh_2	42	6.491	13	1.00E+00	6.87E+42	6.491	13	1.00E+00	6.87E+42	13	14	1.0	c.2.1.2
1mxh_3	42	6.563	13	1.00E+00	1.19E+47	6.563	13	1.00E+00	1.19E+47	13	15	1.3	c.2.1.2
1mxf_0	47	1.510	12	1.00E+00	1.83E+09	1.510	12	1.00E+00	1.83E+09	12	12	0.3	c.2.1.2
1mxf_1	47	1.506	12	1.00E+00	1.60E+09	1.506	12	1.00E+00	1.60E+09	12	12	0.3	c.2.1.2
1mxf_2	46	1.506	12	1.00E+00	1.50E+09	1.506	12	1.00E+00	1.50E+09	12	12	0.3	c.2.1.2
1mxf_3	46	1.525	12	1.00E+00	2.79E+09	1.525	12	1.00E+00	2.79E+09	12	12	0.3	c.2.1.2
1qyw_2	20	1.383	10	1.00E+00	6.64E+07	0.999	6	1.00E+00	1.68E+08	6	9	2.1	c.2.1.2
1qyx_2	19	1.409	10	1.00E+00	1.18E+08	1.307	7	1.00E+00	8.90E+08	6	9	2.0	c.2.1.2
1qyv_0	33	1.600	12	1.00E+00	1.84E+09	1.604	13	1.00E+00	1.18E+08	12	13	0.4	c.2.1.2
1a27_0	63	2.258	13	1.00E+00	2.84E+17	2.258	13	1.00E+00	2.84E+17	13	15	1.3	c.2.1.2
1fdt_0	50	1.281	13	1.00E+00	1.97E+04	0.992	8	1.00E+00	1.09E+06	8	15	3.5	c.2.1.2
1fdu_0	60	9.202	13	1.00E+00	9.15E+46	9.202	13	1.00E+00	9.15E+46	13	16	1.8	c.2.1.2

Table 3: Results for alcohol dehydrogenase matching against its own SCOP superfamily (but not family) without amino acid property.

Site	N	RMSD	q	Pvalue	Evalue	RMSD	q	Pvalue	Evalue	CG	Mean L	Var L	SCOP
1fdu_1	49	1.988	12	1.00E+00	1.60E+15	1.982	13	1.00E+00	1.46E+14	12	14	0.6	c.2.1.2
1fdu_2	45	1.538	13	1.00E+00	1.07E+09	1.562	15	1.00E+00	6.84E+06	13	16	1.2	c.2.1.2
1fdu_3	47	5.389	12	1.00E+00	3.10E+36	5.389	12	1.00E+00	3.10E+36	12	14	1.4	c.2.1.2
1equ_0	43	1.543	13	1.00E+00	1.11E+09	1.549	15	1.00E+00	3.60E+06	13	15	0.6	c.2.1.2
1equ_1	33	1.709	13	1.00E+00	1.88E+10	1.693	14	1.00E+00	7.67E+08	13	15	0.5	c.2.1.2
1fdv_0	52	4.480	13	1.00E+00	1.19E+31	4.480	13	1.00E+00	1.19E+31	13	16	1.4	c.2.1.2
1fdv_1	34	1.590	11	1.00E+00	2.70E+10	1.590	11	1.00E+00	2.70E+10	11	12	0.9	c.2.1.2
1fdv_3	32	1.568	11	1.00E+00	1.21E+10	1.044	8	1.00E+00	1.13E+06	7	11	2.1	c.2.1.2
1fdv_5	30	2.397	11	1.00E+00	8.72E+15	2.397	11	1.00E+00	8.72E+15	11	11	0.3	c.2.1.2
1fmc_0	47	3.943	12	1.00E+00	2.25E+31	3.943	12	1.00E+00	2.25E+31	12	15	1.5	c.2.1.2
1fmc_1	47	1.566	12	1.00E+00	1.15E+07	1.566	12	1.00E+00	1.15E+07	12	14	1.0	c.2.1.2
1ahi_0	48	5.718	13	1.00E+00	1.95E+36	5.718	13	1.00E+00	1.95E+36	13	15	1.8	c.2.1.2
1ahi_1	46	1.630	12	1.00E+00	1.23E+10	1.622	13	1.00E+00	5.74E+08	12	14	0.8	c.2.1.2
1ahh_0	32	1.748	11	1.00E+00	1.50E+12	1.748	11	1.00E+00	1.50E+12	11	12	0.6	c.2.1.2
1ahh_1	29	1.802	11	1.00E+00	4.25E+12	1.802	11	1.00E+00	4.25E+12	11	11	0.3	c.2.1.2
2hsd_0	35	1.353	12	1.00E+00	7.95E+05	1.353	12	1.00E+00	7.95E+05	12	12	0.1	c.2.1.2
2hsd_1	32	3.213	11	1.00E+00	7.95E+23	3.213	11	1.00E+00	7.95E+23	11	14	1.0	c.2.1.2
2hsd_2	29	2.518	11	1.00E+00	1.18E+19	2.518	11	1.00E+00	1.18E+19	11	11	0.1	c.2.1.2
2hsd_3	32	1.433	10	1.00E+00	1.17E+09	1.433	10	1.00E+00	1.17E+09	10	10	0.0	c.2.1.2
1fk8_0	30	1.704	12	1.00E+00	1.77E+11	1.704	12	1.00E+00	1.77E+11	12	13	0.4	c.2.1.2
1fk8_1	28	1.659	11	1.00E+00	9.74E+10	1.659	11	1.00E+00	9.74E+10	11	11	0.2	c.2.1.2
1nff_0	35	1.508	12	1.00E+00	1.34E+08	1.508	12	1.00E+00	1.34E+08	12	14	0.5	c.2.1.2
1nff_1	35	1.643	12	1.00E+00	7.73E+09	1.643	12	1.00E+00	7.73E+09	12	13	0.7	c.2.1.2
1nfr_0	43	4.390	12	1.00E+00	4.21E+33	4.390	12	1.00E+00	4.21E+33	12	13	1.0	c.2.1.2
1nfr_1	44	8.185	12	1.00E+00	5.14E+49	8.185	12	1.00E+00	5.14E+49	12	15	1.6	c.2.1.2
1nfr_2	43	4.555	12	1.00E+00	2.69E+29	4.454	13	1.00E+00	1.10E+29	12	14	0.7	c.2.1.2
1nfr_3	43	4.544	12	1.00E+00	2.47E+34	4.544	12	1.00E+00	2.47E+34	12	14	1.2	c.2.1.2
1bdb_0	54	1.390	14	1.00E+00	6.61E+04	1.390	14	1.00E+00	6.61E+04	14	16	0.8	c.2.1.2
1b14_0	50	1.636	13	1.00E+00	4.20E+10	1.618	14	1.00E+00	1.34E+09	13	14	0.4	c.2.1.2
1b14_1	35	1.196	11	1.00E+00	9.22E+03	1.196	11	1.00E+00	9.22E+03	11	11	0.2	c.2.1.2
1gee_0	32	1.433	11	1.00E+00	2.23E+08	1.468	12	1.00E+00	2.85E+07	11	12	0.2	c.2.1.2
1gee_1	32	3.421	11	1.00E+00	1.28E+25	1.148	10	1.00E+00	2.10E+04	6	12	2.7	c.2.1.2
1gee_2	31	1.618	11	1.00E+00	7.43E+09	1.110	10	1.00E+00	5.77E+03	7	11	2.3	c.2.1.2
1gee_3	33	3.420	11	1.00E+00	1.39E+25	1.091	8	1.00E+00	1.55E+06	6	12	3.3	c.2.1.2
1gco_0	33	3.415	11	1.00E+00	1.31E+25	1.113	10	1.00E+00	7.71E+03	7	12	2.4	c.2.1.2
1gco_1	33	1.432	11	1.00E+00	2.38E+08	1.467	12	1.00E+00	3.04E+07	11	12	0.2	c.2.1.2
1gco_2	33	3.412	11	1.00E+00	1.26E+25	3.348	12	1.00E+00	2.66E+24	11	14	0.9	c.2.1.2
1gco_3	34	3.717	11	1.00E+00	6.14E+26	3.717	11	1.00E+00	6.14E+26	11	13	1.0	c.2.1.2
1g6k_0	31	1.392	11	1.00E+00	5.58E+07	1.531	12	1.00E+00	1.89E+08	11	12	0.3	c.2.1.2
1g6k_1	31	1.393	11	1.00E+00	5.76E+07	1.393	11	1.00E+00	5.76E+07	11	12	0.3	c.2.1.2
1g6k_2	30	1.393	11	1.00E+00	5.20E+07	1.393	11	1.00E+00	5.20E+07	11	12	0.3	c.2.1.2
1g6k_3	32	1.624	12	1.00E+00	3.38E+09	1.013	7	1.00E+00	1.43E+07	7	12	3.1	c.2.1.2
1rwb_0	34	2.132	12	1.00E+00	1.63E+16	1.095	12	1.00E+00	1.21E+01	7	13	1.9	c.2.1.2
1rwb_1	32	1.608	11	1.00E+00	3.69E+10	1.608	11	1.00E+00	3.69E+10	11	12	0.5	c.2.1.2
1rwb_2	32	1.677	11	1.00E+00	2.38E+11	1.677	11	1.00E+00	2.38E+11	11	13	0.5	c.2.1.2
1rwb_3	33	1.617	11	1.00E+00	5.20E+10	1.617	11	1.00E+00	5.20E+10	11	12	0.5	c.2.1.2
1geg_0	38	4.868	11	1.00E+00	7.30E+33	4.868	11	1.00E+00	7.30E+33	11	14	1.5	c.2.1.2
1geg_1	40	7.317	12	1.00E+00	5.46E+40	7.317	12	1.00E+00	5.46E+40	12	14	1.1	c.2.1.2
1geg_2	38	3.708	12	1.00E+00	5.00E+29	3.708	12	1.00E+00	5.00E+29	12	15	1.4	c.2.1.2
1geg_3	39	5.479	12	1.00E+00	2.69E+38	5.479	12	1.00E+00	2.69E+38	12	14	1.4	c.2.1.2
1geg_4	40	6.261	13	1.00E+00	8.36E+41	6.261	13	1.00E+00	8.36E+41	13	15	1.4	c.2.1.2
1geg_5	39	6.198	12	1.00E+00	1.72E+39	6.198	12	1.00E+00	1.72E+39	12	15	1.5	c.2.1.2
1geg_6	40	7.578	12	1.00E+00	3.73E+43	7.578	12	1.00E+00	3.73E+43	12	14	1.3	c.2.1.2
1geg_7	39	1.563	11	1.00E+00	1.93E+10	1.636	12	1.00E+00	8.82E+09	11	12	0.2	c.2.1.2
1iy8_0	41	5.031	11	1.00E+00	7.40E+32	5.031	11	1.00E+00	7.40E+32	11	12	0.9	c.2.1.2
1iy8_1	40	5.431	12	1.00E+00	4.14E+34	5.431	12	1.00E+00	4.14E+34	12	14	1.1	c.2.1.2
1iy8_2	40	1.788	11	1.00E+00	1.12E+12	1.714	12	1.00E+00	1.30E+10	11	13	0.8	c.2.1.2
1iy8_3	40	5.440	12	1.00E+00	4.48E+34	5.440	12	1.00E+00	4.48E+34	12	14	1.2	c.2.1.2
1iy8_4	41	5.440	11	1.00E+00	2.37E+34	5.440	11	1.00E+00	2.37E+34	11	13	1.5	c.2.1.2
1iy8_5	40	1.775	11	1.00E+00	8.24E+11	1.702	12	1.00E+00	9.42E+09	11	13	0.8	c.2.1.2
1iy8_6	40	1.737	12	1.00E+00	2.37E+10	1.737	12	1.00E+00	2.37E+10	12	13	0.8	c.2.1.2
1iy8_7	40	1.689	12	1.00E+00	6.66E+09	1.689	12	1.00E+00	6.66E+09	12	13	0.7	c.2.1.2
1h5q_0	88	7.477	14	1.00E+00	7.25E+47	7.477	14	1.00E+00	7.25E+47	14	17	1.8	c.2.1.2
1h5q_2	95	1.926	14	1.00E+00	2.00E+14	1.926	14	1.00E+00	2.00E+14	14	15	0.5	c.2.1.2
1h5q_4	89	7.914	15	1.00E+00	1.67E+52	7.914	15	1.00E+00	1.67E+52	15	17	1.3	c.2.1.2

Table 4: Results for alcohol dehydrogenase matching against its own SCOP superfamily (but not family) without amino acid property.

Site	N	RMSD	q	Pvalue	Evalue	RMSD	q	Pvalue	Evalue	CG	Mean L	Var L	SCOP
1h5q_5	91	7.799	15	1.00E+00	7.20E+51	7.799	15	1.00E+00	7.20E+51	15	17	1.3	c.2.1.2
1h5q_6	116	1.514	14	1.00E+00	7.51E+07	1.514	14	1.00E+00	7.51E+07	14	14	0.3	c.2.1.2
1h5q_7	117	3.537	14	1.00E+00	4.56E+29	3.537	14	1.00E+00	4.56E+29	14	16	1.4	c.2.1.2
1h5q_9	91	7.646	14	1.00E+00	2.88E+48	7.646	14	1.00E+00	2.88E+48	14	17	2.0	c.2.1.2
1h5q_10	91	7.452	14	1.00E+00	4.18E+43	7.452	14	1.00E+00	4.18E+43	14	17	2.1	c.2.1.2
1h5q_11	92	2.689	16	1.00E+00	2.32E+21	2.689	16	1.00E+00	2.32E+21	16	18	1.4	c.2.1.2
1h5q_13	90	2.692	16	1.00E+00	2.33E+21	2.692	16	1.00E+00	2.33E+21	16	17	1.4	c.2.1.2
1edo_0	35	6.527	12	1.00E+00	1.47E+44	6.527	12	1.00E+00	1.47E+44	12	14	1.3	c.2.1.2
1q7c_0	19	1.823	10	1.00E+00	3.02E+12	1.823	10	1.00E+00	3.02E+12	10	10	0.0	c.2.1.2
1q7c_1	20	2.096	9	1.00E+00	7.00E+15	2.088	10	1.00E+00	7.50E+14	9	10	0.4	c.2.1.2
1q7b_8	30	1.463	12	1.00E+00	1.99E+07	1.463	12	1.00E+00	1.99E+07	12	12	0.1	c.2.1.2
1q7b_9	30	1.673	12	1.00E+00	1.13E+10	1.673	12	1.00E+00	1.13E+10	12	12	0.3	c.2.1.2
1q7b_10	30	5.086	11	1.00E+00	1.33E+29	5.086	11	1.00E+00	1.33E+29	11	11	0.5	c.2.1.2
1q7b_11	29	5.122	11	1.00E+00	1.59E+29	5.122	11	1.00E+00	1.59E+29	11	11	0.5	c.2.1.2
1o5i_0	29	5.466	11	1.00E+00	1.00E+34	5.466	11	1.00E+00	1.00E+34	11	12	0.6	c.2.1.2
1eno_0	30	1.545	10	1.00E+00	3.44E+09	1.545	10	1.00E+00	3.44E+09	10	10	0.2	c.2.1.2
1d7o_0	32	1.653	11	1.00E+00	1.26E+11	1.696	12	1.00E+00	2.63E+10	11	12	0.6	c.2.1.2
1cwu_0	40	1.649	12	1.00E+00	1.39E+10	1.694	14	1.00E+00	2.21E+08	12	15	0.9	c.2.1.2
1cwu_1	40	1.782	12	1.00E+00	5.43E+11	1.769	13	1.00E+00	2.91E+10	12	14	0.8	c.2.1.2
1nhd_0	34	3.077	11	1.00E+00	1.41E+23	3.077	11	1.00E+00	1.41E+23	11	13	1.1	c.2.1.2
1nhd_1	34	3.072	11	1.00E+00	1.31E+23	3.072	11	1.00E+00	1.31E+23	11	13	1.2	c.2.1.2
1nhg_0	38	1.447	11	1.00E+00	1.23E+08	1.447	11	1.00E+00	1.23E+08	11	12	0.8	c.2.1.2
1nhg_1	39	7.018	11	1.00E+00	1.83E+41	7.018	11	1.00E+00	1.83E+41	11	14	1.5	c.2.1.2
1nhw_0	37	1.284	11	1.00E+00	2.68E+06	1.314	12	1.00E+00	2.37E+05	11	12	0.6	c.2.1.2
1nhw_1	37	1.598	11	1.00E+00	4.39E+10	1.598	11	1.00E+00	4.39E+10	11	11	0.2	c.2.1.2
1nnu_0	38	2.213	12	1.00E+00	1.44E+17	2.213	12	1.00E+00	1.44E+17	12	13	0.8	c.2.1.2
1nnu_1	41	2.211	12	1.00E+00	1.74E+17	2.211	12	1.00E+00	1.74E+17	12	13	1.0	c.2.1.2
1uh5_0	38	1.552	11	1.00E+00	1.30E+10	1.634	12	1.00E+00	7.69E+09	11	13	1.2	c.2.1.2
1uh5_1	38	1.565	11	1.00E+00	1.89E+10	1.637	12	1.00E+00	8.38E+09	11	13	1.2	c.2.1.2
1eny_0	28	1.172	11	1.00E+00	1.97E+04	1.172	11	1.00E+00	1.97E+04	11	11	0.3	c.2.1.2
1p44_0	39	4.945	11	1.00E+00	2.95E+32	4.945	11	1.00E+00	2.95E+32	11	13	1.4	c.2.1.2
1p44_1	43	1.768	11	1.00E+00	4.37E+13	1.768	11	1.00E+00	4.37E+13	11	11	0.3	c.2.1.2
1p44_2	41	7.411	12	1.00E+00	1.34E+43	7.411	12	1.00E+00	1.34E+43	12	14	0.9	c.2.1.2
1p44_3	44	7.939	11	1.00E+00	8.03E+43	7.939	11	1.00E+00	8.03E+43	11	13	1.1	c.2.1.2
1p44_4	29	2.988	11	1.00E+00	4.73E+23	2.988	11	1.00E+00	4.73E+23	11	12	0.5	c.2.1.2
1p44_5	28	2.163	11	1.00E+00	1.25E+16	2.163	11	1.00E+00	1.25E+16	11	12	0.4	c.2.1.2
1enz_0	28	1.454	11	1.00E+00	2.81E+08	1.454	11	1.00E+00	2.81E+08	11	11	0.0	c.2.1.2
1p45_0	43	1.931	12	1.00E+00	2.55E+14	1.931	12	1.00E+00	2.55E+14	12	12	0.2	c.2.1.2
1p45_1	34	1.923	12	1.00E+00	8.45E+14	1.979	13	1.00E+00	3.88E+14	12	14	0.9	c.2.1.2
1bvr_0	39	2.223	11	1.00E+00	1.32E+18	2.231	12	1.00E+00	2.33E+17	11	13	0.9	c.2.1.2
1bvr_1	39	3.772	11	1.00E+00	1.80E+27	3.772	11	1.00E+00	1.80E+27	11	12	0.9	c.2.1.2
1bvr_2	39	3.779	11	1.00E+00	1.95E+27	3.779	11	1.00E+00	1.95E+27	11	12	0.9	c.2.1.2
1bvr_3	39	3.773	11	1.00E+00	1.82E+27	3.773	11	1.00E+00	1.82E+27	11	12	0.9	c.2.1.2
1bvr_4	28	2.522	10	1.00E+00	3.68E+18	2.522	10	1.00E+00	3.68E+18	10	11	0.5	c.2.1.2
1bvr_5	30	1.522	11	1.00E+00	2.64E+09	1.522	11	1.00E+00	2.64E+09	11	11	0.0	c.2.1.2
1qsg_0	37	1.705	11	1.00E+00	5.12E+12	1.705	11	1.00E+00	5.12E+12	11	12	0.8	c.2.1.2
1qsg_1	36	4.331	11	1.00E+00	6.45E+29	4.331	11	1.00E+00	6.45E+29	11	12	0.8	c.2.1.2
1qsg_2	36	1.317	12	1.00E+00	9.53E+05	1.317	12	1.00E+00	9.53E+05	12	12	0.2	c.2.1.2
1qsg_3	36	1.346	12	1.00E+00	2.79E+06	1.346	12	1.00E+00	2.79E+06	12	12	0.1	c.2.1.2
1qsg_4	37	4.739	11	1.00E+00	9.86E+34	4.739	11	1.00E+00	9.86E+34	11	13	1.1	c.2.1.2
1qsg_5	36	4.347	11	1.00E+00	7.60E+29	4.347	11	1.00E+00	7.60E+29	11	12	0.8	c.2.1.2
1qsg_6	36	3.015	11	1.00E+00	1.40E+24	3.015	11	1.00E+00	1.40E+24	11	12	0.9	c.2.1.2
1qsg_7	36	3.036	11	1.00E+00	1.94E+24	3.036	11	1.00E+00	1.94E+24	11	12	0.8	c.2.1.2
1qg6_0	37	3.274	11	1.00E+00	6.96E+25	3.274	11	1.00E+00	6.96E+25	11	12	0.9	c.2.1.2
1qg6_1	37	3.274	11	1.00E+00	6.96E+25	3.274	11	1.00E+00	6.96E+25	11	12	0.9	c.2.1.2
1qg6_2	37	1.999	11	1.00E+00	8.16E+15	2.149	12	1.00E+00	3.13E+16	11	12	0.6	c.2.1.2
1qg6_3	37	1.999	11	1.00E+00	8.16E+15	2.149	12	1.00E+00	3.13E+16	11	12	0.5	c.2.1.2
1dfi_0	28	1.333	11	1.00E+00	5.95E+06	1.333	11	1.00E+00	5.95E+06	11	11	0.0	c.2.1.2
1dfi_1	27	1.315	11	1.00E+00	2.91E+06	1.315	11	1.00E+00	2.91E+06	11	11	0.4	c.2.1.2
1dfi_2	26	2.896	11	1.00E+00	7.90E+22	2.896	11	1.00E+00	7.90E+22	11	11	0.3	c.2.1.2
1dfi_3	29	4.241	11	1.00E+00	3.19E+27	4.241	11	1.00E+00	3.19E+27	11	12	0.6	c.2.1.2
1c14_0	38	1.575	11	1.00E+00	1.41E+11	1.575	11	1.00E+00	1.41E+11	11	11	0.1	c.2.1.2
1c14_1	38	6.395	12	1.00E+00	6.37E+43	6.395	12	1.00E+00	6.37E+43	12	14	1.2	c.2.1.2
1dfh_0	34	1.358	12	1.00E+00	3.62E+06	1.358	12	1.00E+00	3.62E+06	12	12	0.0	c.2.1.2
1dfh_1	33	1.362	12	1.00E+00	3.81E+06	1.362	12	1.00E+00	3.81E+06	12	12	0.0	c.2.1.2

Table 5: Results for alcohol dehydrogenase matching against its own SCOP superfamily (but not family) without amino acid property.

Site	N	RMSD	q	Pvalue	Evalue	RMSD	q	Pvalue	Evalue	CG	Mean L	Var L	SCOP
1dfg_0	34	1.350	14	1.00E+00	1.32E+04	1.350	14	1.00E+00	1.32E+04	14	14	0.0	c.2.1.2
1dfg_1	38	1.361	13	1.00E+00	2.00E+05	1.361	13	1.00E+00	2.00E+05	13	13	0.0	c.2.1.2
1mfp_0	43	1.360	13	1.00E+00	2.81E+05	1.360	13	1.00E+00	2.81E+05	13	13	0.2	c.2.1.2
1mfp_1	42	1.369	12	1.00E+00	2.43E+06	1.369	12	1.00E+00	2.43E+06	12	13	0.4	c.2.1.2
1d8a_0	35	7.858	11	1.00E+00	2.47E+43	7.858	11	1.00E+00	2.47E+43	11	12	0.8	c.2.1.2
1d8a_1	36	1.824	12	1.00E+00	6.71E+13	1.824	12	1.00E+00	6.71E+13	12	12	0.4	c.2.1.2
1lx6_0	32	1.226	11	1.00E+00	2.20E+05	1.226	11	1.00E+00	2.20E+05	11	11	0.3	c.2.1.2
1lx6_1	33	6.699	11	1.00E+00	1.26E+40	6.699	11	1.00E+00	1.26E+40	11	12	0.8	c.2.1.2
1lxc_0	39	2.330	12	1.00E+00	2.44E+19	2.330	12	1.00E+00	2.44E+19	12	13	0.4	c.2.1.2
1lxc_1	38	1.712	12	1.00E+00	3.07E+12	1.712	12	1.00E+00	3.07E+12	12	13	0.6	c.2.1.2
1i30_0	31	1.350	11	1.00E+00	1.43E+07	1.350	11	1.00E+00	1.43E+07	11	11	0.2	c.2.1.2
1i30_1	31	1.727	12	1.00E+00	2.56E+12	1.727	12	1.00E+00	2.56E+12	12	12	0.2	c.2.1.2
1i2z_0	39	1.366	13	1.00E+00	2.62E+05	1.366	13	1.00E+00	2.62E+05	13	13	0.2	c.2.1.2
1i2z_1	38	1.889	12	1.00E+00	5.90E+13	1.889	12	1.00E+00	5.90E+13	12	12	0.3	c.2.1.2
1jw7_0	36	1.754	11	1.00E+00	2.51E+12	1.754	11	1.00E+00	2.51E+12	11	11	0.0	c.2.1.2
1jw7_1	37	2.157	11	1.00E+00	2.92E+18	2.157	11	1.00E+00	2.92E+18	11	12	0.8	c.2.1.2
1jw7_2	37	2.943	10	1.00E+00	7.54E+22	2.943	10	1.00E+00	7.54E+22	10	11	0.7	c.2.1.2
1jw7_3	37	2.150	11	1.00E+00	2.38E+17	2.210	12	1.00E+00	1.24E+17	11	12	0.5	c.2.1.2
1jvf_0	35	2.189	11	1.00E+00	5.02E+18	2.189	11	1.00E+00	5.02E+18	11	12	0.7	c.2.1.2
1jvf_1	36	2.188	11	1.00E+00	5.36E+18	2.188	11	1.00E+00	5.36E+18	11	12	0.8	c.2.1.2
1jvf_2	36	2.190	11	1.00E+00	5.60E+18	2.190	11	1.00E+00	5.60E+18	11	12	0.8	c.2.1.2
1jvf_3	35	5.666	11	1.00E+00	6.44E+36	5.666	11	1.00E+00	6.44E+36	11	12	1.0	c.2.1.2
1ae1_0	33	1.054	12	8.59E-01	1.96E+00	1.137	13	7.16E-01	1.26E+00	12	13	0.3	c.2.1.2
1ae1_1	35	5.849	12	1.00E+00	9.13E+35	5.849	12	1.00E+00	9.13E+35	12	15	1.1	c.2.1.2
2ae2_0	43	1.551	12	1.00E+00	9.56E+08	1.609	13	1.00E+00	3.11E+08	12	13	0.4	c.2.1.2
2ae2_1	44	3.032	12	1.00E+00	5.96E+22	3.032	12	1.00E+00	5.96E+22	12	14	1.5	c.2.1.2
1ipe_0	35	1.348	11	1.00E+00	1.95E+07	1.348	11	1.00E+00	1.95E+07	11	11	0.2	c.2.1.2
1ipe_1	37	3.082	12	1.00E+00	7.58E+22	3.082	12	1.00E+00	7.58E+22	12	14	1.2	c.2.1.2
1ipf_0	45	1.563	12	1.00E+00	1.58E+09	1.615	13	1.00E+00	4.32E+08	12	13	0.5	c.2.1.2
1ipf_1	45	1.526	13	1.00E+00	3.68E+09	1.574	14	1.00E+00	1.13E+09	13	15	2.0	c.2.1.2
1g0o_0	42	1.462	13	1.00E+00	1.14E+07	1.462	13	1.00E+00	1.14E+07	13	14	0.7	c.2.1.2
1g0o_1	42	1.470	13	1.00E+00	1.52E+07	1.470	13	1.00E+00	1.52E+07	13	14	0.7	c.2.1.2
1g0o_2	43	2.287	13	1.00E+00	1.72E+17	2.263	14	1.00E+00	1.54E+16	13	14	0.5	c.2.1.2
1g0o_3	43	1.466	13	1.00E+00	1.42E+07	1.466	13	1.00E+00	1.42E+07	13	14	0.7	c.2.1.2
1g0n_0	43	1.467	13	1.00E+00	1.47E+07	1.467	13	1.00E+00	1.47E+07	13	14	0.7	c.2.1.2
1g0n_1	35	1.453	12	1.00E+00	2.32E+07	1.453	12	1.00E+00	2.32E+07	12	13	0.5	c.2.1.2
1doh_0	43	1.549	14	1.00E+00	7.01E+07	1.549	14	1.00E+00	7.01E+07	14	15	0.5	c.2.1.2
1doh_1	42	1.689	13	1.00E+00	2.14E+10	1.711	14	1.00E+00	2.89E+09	13	15	0.9	c.2.1.2
1ybv_0	113	2.107	14	1.00E+00	5.74E+16	2.155	16	1.00E+00	3.80E+15	14	19	2.9	c.2.1.2
1ybv_1	113	1.704	14	1.00E+00	4.70E+10	1.715	15	1.00E+00	4.64E+09	14	17	2.3	c.2.1.2
1ja9_0	43	1.931	12	1.00E+00	3.57E+12	1.931	12	1.00E+00	3.57E+12	12	14	1.7	c.2.1.2
1hdo_0	109	1.751	14	1.00E+00	1.89E+11	1.751	14	1.00E+00	1.89E+11	14	16	0.7	c.2.1.2
1he2_0	120	1.737	14	1.00E+00	7.60E+12	1.737	14	1.00E+00	7.60E+12	14	15	1.0	c.2.1.2
1he3_0	120	1.461	14	1.00E+00	2.73E+08	1.461	14	1.00E+00	2.73E+08	14	15	0.6	c.2.1.2
1he4_0	115	1.460	14	1.00E+00	2.31E+08	1.460	14	1.00E+00	2.31E+08	14	15	0.7	c.2.1.2
1he5_0	114	2.135	13	1.00E+00	8.64E+14	2.135	13	1.00E+00	8.64E+14	13	15	1.5	c.2.1.2
1e6w_0	111	9.837	18	1.00E+00	1.77E+66	9.837	18	1.00E+00	1.77E+66	18	22	2.5	c.2.1.2
1e6w_1	49	2.105	12	1.00E+00	2.68E+16	2.105	12	1.00E+00	2.68E+16	12	13	0.7	c.2.1.2
1e6w_2	51	7.072	13	1.00E+00	1.67E+49	7.072	13	1.00E+00	1.67E+49	13	16	1.9	c.2.1.2
1e6w_3	111	5.608	14	1.00E+00	1.50E+39	5.608	14	1.00E+00	1.50E+39	14	18	2.3	c.2.1.2
1e3s_0	47	6.527	12	1.00E+00	3.64E+44	6.527	12	1.00E+00	3.64E+44	12	15	1.7	c.2.1.2
1e3s_1	47	5.473	12	1.00E+00	9.78E+34	5.473	12	1.00E+00	9.78E+34	12	14	1.5	c.2.1.2
1e3s_2	46	1.812	12	1.00E+00	1.37E+13	1.812	12	1.00E+00	1.37E+13	12	13	0.7	c.2.1.2
1e3s_3	47	1.790	12	1.00E+00	7.99E+12	1.790	12	1.00E+00	7.99E+12	12	13	0.6	c.2.1.2
1e3w_0	49	2.114	12	1.00E+00	3.30E+16	2.114	12	1.00E+00	3.30E+16	12	12	0.3	c.2.1.2
1e3w_1	49	2.053	13	1.00E+00	8.74E+15	2.053	13	1.00E+00	8.74E+15	13	14	0.5	c.2.1.2
1e3w_2	53	2.136	12	1.00E+00	6.99E+16	2.136	12	1.00E+00	6.99E+16	12	13	0.8	c.2.1.2
1e3w_3	55	3.853	13	1.00E+00	2.45E+29	3.853	13	1.00E+00	2.45E+29	13	16	1.7	c.2.1.2
1n5d_1	33	1.654	11	1.00E+00	1.42E+11	1.654	11	1.00E+00	1.42E+11	11	12	0.4	c.2.1.2
1sb8_0	55	1.561	12	1.00E+00	1.52E+10	1.579	13	1.00E+00	1.45E+09	12	14	0.5	c.2.1.2
1sb9_0	53	8.076	13	1.00E+00	1.95E+48	8.076	13	1.00E+00	1.95E+48	13	15	1.4	c.2.1.2
1vl8_2	37	1.476	11	1.00E+00	5.32E+07	1.476	11	1.00E+00	5.32E+07	11	12	0.9	c.2.1.2
1vl8_3	37	4.561	11	1.00E+00	6.97E+30	4.561	11	1.00E+00	6.97E+30	11	13	1.2	c.2.1.2
1sny_1	40	1.531	12	1.00E+00	4.14E+08	1.540	13	1.00E+00	2.76E+07	12	14	0.6	c.2.1.2
1xhl_0	43	3.207	12	1.00E+00	1.84E+25	3.207	12	1.00E+00	1.84E+25	12	13	1.1	c.2.1.2

Table 6: Results for alcohol dehydrogenase matching against its own SCOP superfamily (but not family) without amino acid property.

Site	N	RMSD	q	Pvalue	Evalue	RMSD	q	Pvalue	Evalue	CG	Mean L	Var L	SCOP
1xhl_1	43	1.285	12	1.00E+00	4.90E+05	1.376	13	1.00E+00	5.18E+05	12	14	0.8	c.2.1.2
1gad_0	30	1.646	15	1.00E+00	2.80E+08	0.952	13	3.69E-04	3.69E-04	9	14	2.7	c.2.1.3
1gad_1	29	1.247	15	1.00E+00	8.27E+00	0.912	13	3.84E-05	3.84E-05	9	14	2.1	c.2.1.3
1dc6_0	29	1.216	14	1.00E+00	2.05E+01	0.858	13	1.79E-06	1.79E-06	9	14	2.2	c.2.1.3
1dc6_1	28	1.241	14	1.00E+00	5.87E+01	0.666	11	2.54E-07	2.54E-07	9	13	2.9	c.2.1.3
1gae_0	28	1.371	15	1.00E+00	2.66E+03	0.776	12	1.12E-06	1.12E-06	8	14	3.3	c.2.1.3
1gae_1	29	1.350	16	1.00E+00	1.62E+02	0.747	12	2.23E-07	2.23E-07	9	15	3.6	c.2.1.3
1gd1_0	69	1.854	17	1.00E+00	3.43E+11	1.282	19	6.92E-03	6.95E-03	8	21	3.1	c.2.1.3
1gd1_1	70	1.230	16	1.11E-01	1.17E-01	1.077	15	2.01E-03	2.01E-03	9	21	3.5	c.2.1.3
1gd1_2	69	1.616	16	1.00E+00	1.91E+06	1.294	19	1.42E-02	1.43E-02	9	21	3.6	c.2.1.3
1gd1_3	70	1.649	16	1.00E+00	6.86E+06	1.234	18	4.09E-03	4.10E-03	9	21	3.8	c.2.1.3
1nqo_0	34	1.541	15	1.00E+00	6.89E+06	0.781	13	2.62E-08	2.62E-08	9	15	2.6	c.2.1.3
1nqo_1	34	1.191	14	1.00E+00	1.02E+01	0.786	13	3.60E-08	3.60E-08	9	15	2.6	c.2.1.3
1nqo_2	33	1.567	15	1.00E+00	1.77E+07	0.823	13	3.31E-07	3.31E-07	9	15	2.8	c.2.1.3
1nqo_3	34	1.566	15	1.00E+00	1.87E+07	0.768	13	1.13E-08	1.13E-08	9	14	2.9	c.2.1.3
1nq5_0	32	1.542	15	1.00E+00	5.94E+06	0.887	14	2.31E-07	2.31E-07	9	15	2.8	c.2.1.3
1nq5_1	32	1.175	14	9.80E-01	3.91E+00	0.778	13	1.79E-08	1.79E-08	9	14	2.4	c.2.1.3
1nq5_2	31	1.585	15	1.00E+00	2.97E+07	0.823	13	2.73E-07	2.73E-07	9	15	2.4	c.2.1.3
1nq5_3	32	1.545	15	1.00E+00	6.71E+06	0.757	13	4.52E-09	4.52E-09	9	14	2.7	c.2.1.3
1npt_0	35	1.187	14	1.00E+00	9.23E+00	0.784	12	3.57E-06	3.57E-06	9	16	3.9	c.2.1.3
1npt_1	34	1.192	14	1.00E+00	1.07E+01	0.776	13	1.90E-08	1.90E-08	9	15	3.1	c.2.1.3
1npt_2	34	1.184	14	9.99E-01	7.30E+00	0.772	13	1.46E-08	1.46E-08	9	14	2.7	c.2.1.3
1npt_3	34	1.257	15	1.00E+00	2.22E+01	0.766	13	9.88E-09	9.88E-09	10	15	3.2	c.2.1.3
1nqa_0	36	1.597	15	1.00E+00	7.53E+07	0.794	12	6.91E-06	6.91E-06	9	15	3.5	c.2.1.3
1nqa_1	37	1.559	15	1.00E+00	1.84E+07	0.762	13	9.87E-09	9.87E-09	9	14	2.6	c.2.1.3
1nqa_2	36	1.573	15	1.00E+00	2.94E+07	0.748	13	3.57E-09	3.57E-09	9	14	2.4	c.2.1.3
1nqa_3	36	1.559	15	1.00E+00	1.69E+07	0.766	13	1.18E-08	1.18E-08	9	15	3.2	c.2.1.3
2dbv_0	33	1.220	15	6.10E-01	9.41E-01	0.695	10	4.40E-04	4.40E-04	8	14	3.6	c.2.1.3
2dbv_1	35	1.496	14	1.00E+00	5.09E+06	0.700	9	1.15E-01	1.22E-01	6	15	3.3	c.2.1.3
2dbv_2	33	1.224	14	1.00E+00	1.31E+01	0.803	12	8.79E-06	8.79E-06	8	14	2.1	c.2.1.3
2dbv_3	34	1.625	15	1.00E+00	3.10E+07	0.866	12	2.95E-04	2.95E-04	8	14	2.7	c.2.1.3
1dbv_0	31	1.568	15	1.00E+00	2.73E+06	0.789	12	3.27E-06	3.27E-06	8	13	1.9	c.2.1.3
1dbv_1	33	1.570	15	1.00E+00	3.58E+06	0.815	11	1.46E-03	1.46E-03	8	14	2.7	c.2.1.3
1dbv_2	39	1.207	15	5.63E-01	8.28E-01	1.207	15	5.63E-01	8.28E-01	15	17	4.1	c.2.1.3
1dbv_3	29	1.590	15	1.00E+00	5.12E+06	0.818	12	1.36E-05	1.36E-05	7	13	1.8	c.2.1.3
4dbv_0	32	1.417	13	1.00E+00	9.65E+05	0.772	13	1.21E-08	1.21E-08	6	14	1.8	c.2.1.3
4dbv_1	33	1.175	14	9.86E-01	4.31E+00	0.770	12	1.31E-06	1.31E-06	9	15	3.6	c.2.1.3
4dbv_2	32	1.428	13	1.00E+00	1.45E+06	0.741	13	1.55E-09	1.55E-09	6	14	1.5	c.2.1.3
4dbv_3	30	1.587	14	1.00E+00	9.22E+07	0.708	13	1.29E-10	1.29E-10	6	14	1.5	c.2.1.3
3dbv_0	32	1.204	14	9.92E-01	4.80E+00	0.756	13	4.23E-09	4.23E-09	10	14	1.6	c.2.1.3
3dbv_1	33	1.409	14	1.00E+00	1.38E+05	0.721	11	1.04E-05	1.04E-05	6	16	3.7	c.2.1.3
3dbv_2	30	1.623	15	1.00E+00	1.17E+08	0.768	13	7.64E-09	7.64E-09	9	14	1.7	c.2.1.3
3dbv_3	30	1.632	15	1.00E+00	1.65E+08	0.788	13	2.78E-08	2.78E-08	9	14	1.7	c.2.1.3
1cer_0	32	1.580	13	1.00E+00	2.84E+08	0.811	12	1.25E-05	1.25E-05	6	14	2.7	c.2.1.3
1cer_1	30	1.082	14	2.84E-02	2.88E-02	0.979	13	1.50E-03	1.50E-03	9	13	1.9	c.2.1.3
1cer_2	30	1.143	14	4.84E-01	6.61E-01	0.927	13	9.69E-05	9.69E-05	9	14	2.2	c.2.1.3
1cer_3	29	1.538	13	1.00E+00	5.13E+07	0.773	11	1.16E-04	1.16E-04	7	13	2.2	c.2.1.3
1vc2_0	29	1.746	12	1.00E+00	7.65E+10	1.035	13	2.18E-02	2.21E-02	7	14	2.8	c.2.1.3
1hdg_0	40	1.237	15	1.00E+00	1.36E+01	0.931	13	2.93E-04	2.93E-04	10	16	3.0	c.2.1.3
1hdg_1	41	0.854	14	1.01E-07	1.01E-07	0.854	14	1.01E-07	1.01E-07	14	16	3.0	c.2.1.3
1cf2_4	31	2.183	12	1.00E+00	3.36E+14	2.183	12	1.00E+00	3.36E+14	12	13	0.8	c.2.1.3
1cf2_5	31	1.705	12	1.00E+00	4.64E+09	1.705	12	1.00E+00	4.64E+09	12	13	0.6	c.2.1.3
1cf2_6	31	1.196	13	1.00E+00	1.25E+02	0.972	9	1.00E+00	4.00E+03	7	11	2.0	c.2.1.3
1cf2_7	31	1.745	12	1.00E+00	1.33E+10	1.745	12	1.00E+00	1.33E+10	12	13	0.6	c.2.1.3
1gga_0	32	1.497	14	1.00E+00	4.01E+06	0.695	10	4.00E-04	4.00E-04	8	14	3.5	c.2.1.3
1gga_1	32	2.111	14	1.00E+00	1.35E+15	0.866	11	1.53E-02	1.54E-02	7	14	3.7	c.2.1.3
1gga_2	32	2.108	14	1.00E+00	1.25E+15	0.701	10	5.42E-04	5.42E-04	7	13	3.3	c.2.1.3
1gga_3	32	2.114	14	1.00E+00	1.47E+15	0.700	10	5.15E-04	5.16E-04	7	14	3.6	c.2.1.3
1gga_4	31	2.108	14	1.00E+00	1.13E+15	0.863	11	1.20E-02	1.21E-02	7	14	4.2	c.2.1.3
1gga_5	31	2.115	14	1.00E+00	1.37E+15	0.875	11	2.09E-02	2.12E-02	7	14	3.8	c.2.1.3
1ml3_0	39	1.140	13	1.00E+00	2.08E+01	0.923	11	3.10E-01	3.71E-01	7	17	3.7	c.2.1.3
1ml3_1	34	0.962	14	5.14E-05	5.14E-05	0.962	14	5.14E-05	5.14E-05	14	15	3.7	c.2.1.3
1ml3_2	36	4.841	13	1.00E+00	1.00E+34	0.806	9	1.00E+00	1.45E+01	2	14	5.6	c.2.1.3
1qxs_0	40	1.272	13	1.00E+00	6.86E+03	0.961	11	8.70E-01	2.04E+00	6	17	3.7	c.2.1.3
1qxs_1	33	1.087	13	6.44E-01	1.03E+00	1.087	13	6.44E-01	1.03E+00	13	16	3.8	c.2.1.3

Table 7: Results for alcohol dehydrogenase matching against its own SCOP superfamily (but not family) without amino acid property.

Site	N	RMSD	q	Pvalue	Evalue	RMSD	q	Pvalue	Evalue	CG	Mean L	Var L	SCOP
1qxs_2	32	1.337	13	1.00E+00	4.64E+04	0.850	11	7.22E-03	7.25E-03	6	15	3.4	c.2.1.3
1qxs_3	31	1.577	15	1.00E+00	2.17E+07	0.798	9	9.90E-01	4.56E+00	5	14	3.1	c.2.1.3
1gyp_0	42	0.930	15	3.22E-07	3.22E-07	0.778	13	4.14E-08	4.14E-08	10	16	4.1	c.2.1.3
1gyp_1	44	0.958	14	1.86E-04	1.86E-04	0.785	12	7.64E-06	7.64E-06	9	17	3.9	c.2.1.3
1gyp_2	42	1.097	15	9.07E-03	9.12E-03	0.861	13	6.72E-06	6.72E-06	10	17	4.2	c.2.1.3
1gyp_3	42	1.213	15	9.91E-01	4.67E+00	0.927	14	6.09E-06	6.09E-06	10	16	3.4	c.2.1.3
1a7k_0	33	0.813	14	3.12E-09	3.12E-09	0.813	14	3.12E-09	3.12E-09	14	15	3.6	c.2.1.3
1a7k_1	34	1.729	14	1.00E+00	1.82E+10	0.767	11	1.38E-04	1.38E-04	8	16	4.3	c.2.1.3
1a7k_2	31	0.827	14	6.83E-09	6.83E-09	0.827	14	6.83E-09	6.83E-09	14	15	3.2	c.2.1.3
1a7k_3	39	0.979	15	6.21E-06	6.21E-06	0.734	12	2.52E-07	2.52E-07	10	15	4.0	c.2.1.3
1gpd_0	73	1.492	19	1.00E+00	1.10E+03	0.995	14	1.63E-03	1.64E-03	11	17	2.4	c.2.1.3
1gpd_1	78	1.623	15	1.00E+00	1.31E+10	1.048	12	1.00E+00	8.49E+00	8	19	4.5	c.2.1.3
1dss_0	45	1.061	15	1.42E-03	1.42E-03	0.908	14	2.40E-06	2.40E-06	10	16	3.3	c.2.1.3
1dss_1	46	1.997	15	1.00E+00	1.70E+14	1.018	15	4.96E-05	4.96E-05	8	17	3.1	c.2.1.3
1szj_0	44	1.617	16	1.00E+00	1.04E+08	1.239	15	8.10E-01	1.66E+00	10	19	3.9	c.2.1.3
1szj_1	33	1.017	14	1.12E-03	1.12E-03	0.693	11	2.10E-06	2.10E-06	9	13	3.1	c.2.1.3
3gpd_0	91	1.339	17	1.00E+00	1.04E+02	0.980	15	1.86E-05	1.86E-05	8	20	4.7	c.2.1.3
3gpd_1	35	1.513	17	1.00E+00	1.49E+05	0.828	11	3.31E-03	3.32E-03	10	14	1.9	c.2.1.3
1j0x_0	38	1.791	15	1.00E+00	1.10E+11	0.708	11	7.72E-06	7.72E-06	7	14	2.9	c.2.1.3
1j0x_1	39	1.740	15	1.00E+00	1.98E+10	0.966	13	1.73E-03	1.73E-03	8	15	3.0	c.2.1.3
1rm4_0	38	1.190	14	9.86E-01	4.28E+00	0.857	10	4.95E-01	6.83E-01	7	17	3.0	c.2.1.3
1rm4_1	30	1.511	13	1.00E+00	4.37E+06	1.511	13	1.00E+00	4.37E+06	13	15	0.9	c.2.1.3
1rm4_2	32	1.324	14	1.00E+00	2.28E+02	0.627	8	6.31E-01	9.98E-01	5	13	3.2	c.2.1.3
1rm5_0	27	0.756	12	3.07E-07	3.07E-07	0.756	12	3.07E-07	3.07E-07	12	12	2.4	c.2.1.3
1rm5_1	31	0.803	12	7.24E-06	7.24E-06	0.803	12	7.24E-06	7.24E-06	12	13	3.1	c.2.1.3
1rm5_2	27	1.868	12	1.00E+00	1.49E+12	0.736	9	2.66E-01	3.10E-01	6	10	2.1	c.2.1.3
1rm3_0	33	0.694	14	3.10E-13	3.10E-13	0.694	14	3.10E-13	3.10E-13	14	13	2.1	c.2.1.3
1rm3_1	36	0.796	14	5.93E-10	5.93E-10	0.796	14	5.93E-10	5.93E-10	14	14	2.8	c.2.1.3
1rm3_2	39	0.917	14	2.67E-06	2.67E-06	0.916	16	3.05E-10	3.05E-10	14	18	0.8	c.2.1.3
1nbo_0	40	1.207	15	5.91E-01	8.95E-01	1.207	15	5.91E-01	8.95E-01	15	17	3.8	c.2.1.3
1nbo_1	40	1.726	15	1.00E+00	1.92E+09	0.935	11	2.89E-01	3.41E-01	8	17	4.8	c.2.1.3
1nbo_2	41	1.300	15	1.00E+00	8.34E+01	0.901	11	8.50E-02	8.89E-02	8	17	4.5	c.2.1.3
1jn0_0	35	0.859	14	5.20E-08	5.20E-08	0.859	14	5.20E-08	5.20E-08	14	13	2.6	c.2.1.3
1jn0_1	45	0.931	15	2.17E-07	2.17E-07	0.931	15	2.17E-07	2.17E-07	15	17	4.4	c.2.1.3
1jn0_2	35	0.788	13	4.48E-08	4.48E-08	0.788	13	4.48E-08	4.48E-08	13	13	3.6	c.2.1.3
1gl3_0	120	1.466	15	1.00E+00	7.12E+07	1.455	16	1.00E+00	1.88E+06	15	18	1.3	c.2.1.3
1gl3_1	102	6.267	14	1.00E+00	4.72E+43	6.267	14	1.00E+00	4.72E+43	14	18	2.6	c.2.1.3
1mb4_0	37	1.505	12	1.00E+00	5.50E+06	1.505	12	1.00E+00	5.50E+06	12	14	1.2	c.2.1.3
1mb4_1	36	2.019	12	1.00E+00	1.68E+12	2.019	12	1.00E+00	1.68E+12	12	14	1.5	c.2.1.3
1pqu_0	40	1.348	13	1.00E+00	8.37E+03	1.348	13	1.00E+00	8.37E+03	13	14	0.8	c.2.1.3
1pqu_1	40	1.369	14	1.00E+00	2.68E+03	1.369	14	1.00E+00	2.68E+03	14	15	0.6	c.2.1.3
1pqu_3	42	1.291	14	1.00E+00	1.38E+02	1.291	14	1.00E+00	1.38E+02	14	15	0.9	c.2.1.3
1nvm_16	36	0.824	16	8.85E-13	8.85E-13	0.824	16	8.85E-13	8.85E-13	16	16	0.4	c.2.1.3
1nvm_17	36	0.991	15	4.64E-06	4.64E-06	0.963	16	2.23E-08	2.23E-08	15	17	0.5	c.2.1.3
1nvm_18	36	1.098	16	1.12E-04	1.12E-04	0.512	10	6.81E-09	6.81E-09	8	14	3.9	c.2.1.3
1ebf_2	26	1.184	13	1.00E+00	1.36E+02	0.911	11	1.10E-01	1.17E-01	9	12	1.3	c.2.1.3
1e5q_0	120	1.007	17	1.23E-06	1.23E-06	1.007	17	1.23E-06	1.23E-06	17	22	4.1	c.2.1.3
1e5q_1	120	1.169	16	5.53E-01	8.04E-01	0.881	14	2.78E-05	2.78E-05	11	21	5.9	c.2.1.3
1e5q_2	116	0.999	15	6.04E-04	6.04E-04	0.998	16	4.10E-05	4.10E-05	11	21	5.6	c.2.1.3
1e5q_3	120	1.009	15	1.24E-03	1.24E-03	1.019	16	8.12E-05	8.12E-05	15	19	2.2	c.2.1.3
1e5q_4	120	1.003	15	8.58E-04	8.58E-04	1.017	16	7.12E-05	7.12E-05	15	19	2.1	c.2.1.3
1e5q_5	119	0.995	15	5.09E-04	5.09E-04	1.007	16	3.58E-05	3.58E-05	15	18	1.7	c.2.1.3
1e5q_6	116	0.998	15	5.68E-04	5.68E-04	0.998	15	5.68E-04	5.68E-04	15	17	1.3	c.2.1.3
1e5q_7	120	1.161	16	3.98E-01	5.08E-01	0.899	14	9.21E-05	9.21E-05	12	20	5.0	c.2.1.3
1f06_0	40	0.818	16	7.62E-13	7.62E-13	0.818	16	7.62E-13	7.62E-13	16	17	2.4	c.2.1.3
1f06_1	28	0.756	14	2.95E-11	2.95E-11	0.756	14	2.95E-11	2.95E-11	14	14	1.2	c.2.1.3
3dap_0	73	0.945	18	1.68E-11	1.68E-11	0.945	18	1.68E-11	1.68E-11	18	18	2.7	c.2.1.3
3dap_1	113	0.884	18	4.88E-13	4.88E-13	0.884	18	4.88E-13	4.88E-13	18	24	6.3	c.2.1.3
1dap_0	73	0.837	16	1.34E-11	1.34E-11	0.837	16	1.34E-11	1.34E-11	16	18	2.2	c.2.1.3
1dap_1	112	1.178	18	5.78E-04	5.78E-04	1.178	18	5.78E-04	5.78E-04	18	24	5.6	c.2.1.3
1dih_0	61	2.197	14	1.00E+00	9.63E+16	2.197	14	1.00E+00	9.63E+16	14	15	0.7	c.2.1.3
1arz_0	33	1.359	11	1.00E+00	2.34E+07	1.341	12	1.00E+00	4.35E+05	11	13	1.1	c.2.1.3
1arz_1	32	1.189	11	1.00E+00	5.66E+04	1.263	13	1.00E+00	6.57E+02	11	15	1.2	c.2.1.3
1arz_2	35	2.172	11	1.00E+00	2.63E+14	2.172	11	1.00E+00	2.63E+14	11	14	0.9	c.2.1.3
1p9l_0	36	1.048	14	3.28E-03	3.28E-03	1.048	14	3.28E-03	3.28E-03	14	16	4.1	c.2.1.3

Table 8: Results for alcohol dehydrogenase matching against its own SCOP superfamily (but not family) without amino acid property.

Site	N	RMSD	q	Pvalue	Evalue	RMSD	q	Pvalue	Evalue	CG	Mean L	Var L	SCOP
1p9l_1	37	1.048	14	3.57E-03	3.57E-03	1.048	14	3.57E-03	3.57E-03	14	16	4.2	c.2.1.3
1c3v_0	37	1.307	14	1.00E+00	6.95E+02	0.561	8	6.29E-02	6.49E-02	7	16	3.9	c.2.1.3
1c3v_1	37	1.307	14	1.00E+00	6.95E+02	0.561	8	6.29E-02	6.49E-02	7	16	3.9	c.2.1.3
1vm6_0	35	1.528	13	1.00E+00	3.46E+08	0.816	9	1.00E+00	1.98E+01	6	15	4.0	c.2.1.3
1vm6_1	35	1.564	13	1.00E+00	1.22E+09	0.812	9	1.00E+00	1.69E+01	7	15	4.3	c.2.1.3
1vm6_2	36	1.618	14	1.00E+00	2.90E+09	0.801	9	1.00E+00	1.18E+01	7	15	3.2	c.2.1.3
1vm6_3	30	1.555	13	1.00E+00	5.54E+08	0.842	9	1.00E+00	3.40E+01	7	14	4.5	c.2.1.3
1gr0_0	44	1.122	17	3.94E-04	3.94E-04	0.893	13	1.58E-04	1.58E-04	12	14	2.4	c.2.1.3
1p1h_0	36	6.282	13	1.00E+00	7.24E+41	6.282	13	1.00E+00	7.24E+41	13	15	1.1	c.2.1.3
1p1h_1	38	6.334	13	1.00E+00	1.34E+42	6.334	13	1.00E+00	1.34E+42	13	15	1.2	c.2.1.3
1p1h_2	37	2.174	12	1.00E+00	5.53E+16	2.174	12	1.00E+00	5.53E+16	12	12	0.2	c.2.1.3
1p1h_3	38	5.532	12	1.00E+00	1.22E+33	0.985	11	1.00E+00	1.04E+01	0	13	2.4	c.2.1.3
1jkf_0	38	2.532	13	1.00E+00	1.64E+18	3.019	15	1.00E+00	1.48E+21	13	16	0.8	c.2.1.3
1jkf_1	35	1.580	12	1.00E+00	2.20E+11	1.580	12	1.00E+00	2.20E+11	12	12	0.2	c.2.1.3
1p1i_0	37	2.591	12	1.00E+00	2.07E+19	2.591	12	1.00E+00	2.07E+19	12	13	0.8	c.2.1.3
1p1i_1	37	1.734	12	1.00E+00	1.17E+11	1.734	12	1.00E+00	1.17E+11	12	13	0.7	c.2.1.3
1la2_32	39	5.393	12	1.00E+00	2.75E+34	5.393	12	1.00E+00	2.75E+34	12	13	0.9	c.2.1.3
1la2_33	37	1.325	12	1.00E+00	1.40E+06	0.855	9	1.00E+00	1.77E+02	8	11	2.9	c.2.1.3
1la2_34	37	1.729	12	1.00E+00	6.94E+11	1.729	12	1.00E+00	6.94E+11	12	12	0.1	c.2.1.3
1la2_35	39	0.872	12	1.83E-03	1.83E-03	0.872	12	1.83E-03	1.83E-03	12	11	2.4	c.2.1.3
1u1i_0	46	1.412	16	1.00E+00	6.14E+04	0.983	12	6.72E-01	1.11E+00	9	17	4.7	c.2.1.3
1u1i_1	47	1.597	12	1.00E+00	1.67E+11	1.597	12	1.00E+00	1.67E+11	12	13	0.5	c.2.1.3
1u1i_4	39	1.203	15	9.99E-01	7.30E+00	1.203	15	9.99E-01	7.30E+00	15	14	4.1	c.2.1.3
1u1i_5	36	1.219	12	1.00E+00	2.11E+04	1.219	12	1.00E+00	2.11E+04	12	12	0.3	c.2.1.3
1vko_0	52	1.356	13	1.00E+00	1.80E+06	1.037	12	1.00E+00	9.28E+00	7	17	4.1	c.2.1.3
1j5p_0	34	0.873	13	1.21E-05	1.21E-05	0.873	13	1.21E-05	1.21E-05	13	11	2.5	c.2.1.3
1h2h_0	70	1.572	15	1.00E+00	3.87E+07	1.572	15	1.00E+00	3.87E+07	15	15	0.5	c.2.1.3
1q0h_1	47	1.519	12	1.00E+00	8.96E+07	1.519	12	1.00E+00	8.96E+07	12	14	1.5	c.2.1.3
1q0q_0	43	1.379	12	1.00E+00	3.69E+06	1.399	13	1.00E+00	2.78E+05	12	15	1.1	c.2.1.3
1q0q_1	43	1.238	12	1.00E+00	7.81E+04	1.319	14	1.00E+00	1.83E+03	12	15	1.2	c.2.1.3
1jvs_2	33	1.355	12	1.00E+00	1.71E+05	1.355	12	1.00E+00	1.71E+05	12	13	0.8	c.2.1.3
1jvs_6	33	2.164	13	1.00E+00	3.99E+14	2.164	13	1.00E+00	3.99E+14	13	14	1.0	c.2.1.3
1q0l_0	43	1.355	12	1.00E+00	6.69E+06	1.383	13	1.00E+00	6.76E+05	12	14	0.6	c.2.1.3
1r0l_0	16	1.443	9	1.00E+00	4.17E+09	1.443	9	1.00E+00	4.17E+09	9	10	0.5	c.2.1.3
1r0l_1	16	4.296	9	1.00E+00	8.11E+26	4.296	9	1.00E+00	8.11E+26	9	9	0.1	c.2.1.3
1r0l_2	16	1.441	9	1.00E+00	3.96E+09	1.441	9	1.00E+00	3.96E+09	9	10	0.6	c.2.1.3
1r0l_3	17	4.284	9	1.00E+00	3.70E+28	4.284	9	1.00E+00	3.70E+28	9	10	0.6	c.2.1.3
1h6d_0	120	1.177	15	9.96E-01	5.61E+00	1.177	15	9.96E-01	5.61E+00	15	17	1.4	c.2.1.3
1h6d_1	116	1.953	15	1.00E+00	7.11E+14	1.953	15	1.00E+00	7.11E+14	15	16	0.7	c.2.1.3
1h6d_2	119	1.194	15	1.00E+00	1.29E+01	1.194	15	1.00E+00	1.29E+01	15	17	1.4	c.2.1.3
1h6d_3	120	1.538	15	1.00E+00	5.34E+07	1.540	16	1.00E+00	2.92E+06	15	17	0.6	c.2.1.3
1h6d_4	120	1.193	15	1.00E+00	1.26E+01	0.700	9	9.98E-01	6.47E+00	9	15	8.6	c.2.1.3
1h6d_5	119	1.204	15	1.00E+00	2.14E+01	1.204	15	1.00E+00	2.14E+01	15	17	1.4	c.2.1.3
1h6d_6	119	1.182	15	9.99E-01	7.06E+00	1.182	15	9.99E-01	7.06E+00	15	17	1.4	c.2.1.3
1h6d_7	118	8.558	15	1.00E+00	8.38E+56	8.558	15	1.00E+00	8.38E+56	15	19	2.1	c.2.1.3
1h6d_8	120	1.197	15	1.00E+00	1.54E+01	1.197	15	1.00E+00	1.54E+01	15	17	1.4	c.2.1.3
1h6d_9	120	1.188	15	1.00E+00	9.81E+00	1.188	15	1.00E+00	9.81E+00	15	17	1.3	c.2.1.3
1h6d_10	120	1.189	15	1.00E+00	1.03E+01	1.189	15	1.00E+00	1.03E+01	15	17	1.4	c.2.1.3
1h6d_11	120	1.185	15	1.00E+00	8.43E+00	1.185	15	1.00E+00	8.43E+00	15	15	6.2	c.2.1.3
1h6c_0	120	1.380	14	1.00E+00	1.16E+05	1.380	14	1.00E+00	1.16E+05	14	14	0.4	c.2.1.3
1h6c_1	115	1.210	15	1.00E+00	2.60E+01	1.210	15	1.00E+00	2.60E+01	15	16	1.2	c.2.1.3
1h6a_0	120	1.216	15	1.00E+00	3.97E+01	1.216	15	1.00E+00	3.97E+01	15	16	1.2	c.2.1.3
1h6a_1	108	1.220	15	1.00E+00	3.52E+01	1.220	15	1.00E+00	3.52E+01	15	17	1.3	c.2.1.3
1h6b_0	120	1.653	14	1.00E+00	1.06E+10	1.653	14	1.00E+00	1.06E+10	14	14	0.2	c.2.1.3
1h6b_1	120	1.236	15	1.00E+00	1.06E+02	1.236	15	1.00E+00	1.06E+02	15	17	1.5	c.2.1.3
1ofg_0	41	1.238	14	1.00E+00	1.66E+02	1.238	14	1.00E+00	1.66E+02	14	15	0.5	c.2.1.3
1ofg_1	41	1.238	14	1.00E+00	1.66E+02	1.238	14	1.00E+00	1.66E+02	14	15	0.5	c.2.1.3
1ofg_2	41	1.238	14	1.00E+00	1.66E+02	1.238	14	1.00E+00	1.66E+02	14	15	0.4	c.2.1.3
1ofg_3	41	1.238	14	1.00E+00	1.66E+02	1.238	14	1.00E+00	1.66E+02	14	15	0.5	c.2.1.3
1ofg_4	31	1.238	14	1.00E+00	7.01E+01	1.238	14	1.00E+00	7.01E+01	14	15	0.5	c.2.1.3
1ofg_5	31	1.238	14	1.00E+00	7.01E+01	0.689	9	5.82E-02	6.00E-02	8	11	1.7	c.2.1.3
1evj_0	25	1.882	12	1.00E+00	1.08E+14	1.882	12	1.00E+00	1.08E+14	12	12	0.3	c.2.1.3
1evj_1	25	1.881	12	1.00E+00	1.05E+14	1.881	12	1.00E+00	1.05E+14	12	12	0.2	c.2.1.3
1evj_2	25	1.857	11	1.00E+00	7.94E+13	1.857	11	1.00E+00	7.94E+13	11	11	0.4	c.2.1.3
1evj_3	25	1.882	12	1.00E+00	1.08E+14	1.882	12	1.00E+00	1.08E+14	12	12	0.3	c.2.1.3

Table 9: Results for alcohol dehydrogenase matching against its own SCOP superfamily (but not family) without amino acid property.

Site	N	RMSD	q	Pvalue	Evalue	RMSD	q	Pvalue	Evalue	CG	Mean L	Var L	SCOP
2dpg_0	17	1.717	10	1.00E+00	3.00E+10	1.717	10	1.00E+00	3.00E+10	10	10	0.1	c.2.1.3
1e7y_2	35	1.571	12	1.00E+00	2.91E+10	1.571	12	1.00E+00	2.91E+10	12	12	0.1	c.2.1.3
1h9a_1	41	1.560	13	1.00E+00	9.55E+09	1.560	13	1.00E+00	9.55E+09	13	14	0.6	c.2.1.3
1h94_0	44	1.499	12	1.00E+00	2.70E+10	1.482	13	1.00E+00	6.64E+08	12	14	0.7	c.2.1.3
1qki_5	24	1.920	9	1.00E+00	4.23E+15	1.920	9	1.00E+00	4.23E+15	9	9	0.2	c.2.1.3
1qki_7	24	6.955	9	1.00E+00	1.39E+37	6.955	9	1.00E+00	1.39E+37	9	9	0.4	c.2.1.3
1qki_9	24	1.892	10	1.00E+00	1.80E+15	1.892	10	1.00E+00	1.80E+15	10	10	0.0	c.2.1.3
1qki_11	25	4.740	10	1.00E+00	4.25E+32	4.740	10	1.00E+00	4.25E+32	10	10	0.3	c.2.1.3
1qki_13	23	2.313	9	1.00E+00	4.80E+18	2.313	9	1.00E+00	4.80E+18	9	9	0.2	c.2.1.3
1qki_15	24	1.920	9	1.00E+00	4.23E+15	1.920	9	1.00E+00	4.23E+15	9	9	0.2	c.2.1.3
1qki_17	24	1.905	10	1.00E+00	2.42E+15	1.905	10	1.00E+00	2.42E+15	10	10	0.2	c.2.1.3
1qki_19	25	1.890	10	1.00E+00	1.95E+15	1.890	10	1.00E+00	1.95E+15	10	10	0.1	c.2.1.3
2nad_0	40	1.112	14	2.84E-01	3.34E-01	1.112	14	2.84E-01	3.34E-01	14	15	1.1	c.2.1.4
2nad_1	40	1.121	15	2.96E-02	3.01E-02	1.121	15	2.96E-02	3.01E-02	15	16	1.0	c.2.1.4
1qp8_10	34	1.253	15	1.00E+00	1.82E+01	1.056	12	9.04E-01	2.34E+00	9	16	2.8	c.2.1.4
1mx3_0	37	1.004	15	1.11E-05	1.11E-05	1.004	15	1.11E-05	1.11E-05	15	17	2.2	c.2.1.4
1hku_0	115	1.308	17	1.00E+00	1.06E+01	0.816	10	9.97E-01	5.86E+00	8	21	4.4	c.2.1.4
1hl3_0	80	1.516	17	1.00E+00	7.97E+04	0.935	11	9.96E-01	5.62E+00	9	20	4.2	c.2.1.4
1dxy_0	73	1.395	17	1.00E+00	9.35E+02	0.554	8	3.08E-01	3.68E-01	8	15	6.1	c.2.1.4
1psd_0	76	1.259	16	8.94E-01	2.25E+00	1.259	16	8.94E-01	2.25E+00	16	16	2.9	c.2.1.4
1j49_0	37	1.019	15	6.34E-05	6.34E-05	1.019	15	6.34E-05	6.34E-05	15	13	3.3	c.2.1.4
1j49_1	29	1.438	16	1.00E+00	1.11E+04	0.527	9	7.48E-06	7.48E-06	9	12	1.3	c.2.1.4
2dld_0	29	1.463	14	1.00E+00	7.94E+05	0.560	9	5.38E-05	5.38E-05	9	13	2.8	c.2.1.4
2dld_1	27	1.088	14	2.81E-02	2.85E-02	0.622	9	1.31E-03	1.31E-03	9	12	3.0	c.2.1.4
1pjc_0	32	0.713	16	0.00E+00	5.06E-17	0.713	16	0.00E+00	5.06E-17	16	14	1.7	c.2.1.4
1f8g_11	38	0.913	13	3.35E-04	3.35E-04	0.913	13	3.35E-04	3.35E-04	13	13	0.2	c.2.1.4
1f8g_32	29	0.821	13	4.58E-07	4.58E-07	0.821	13	4.58E-07	4.58E-07	13	13	0.2	c.2.1.4
1f8g_33	26	0.880	12	8.15E-04	8.15E-04	0.880	12	8.15E-04	8.15E-04	12	13	0.2	c.2.1.4
1f8g_34	25	0.996	12	2.47E-01	2.84E-01	0.996	12	2.47E-01	2.84E-01	12	12	0.0	c.2.1.4
1nm5_0	30	0.779	13	2.95E-08	2.95E-08	0.779	13	2.95E-08	2.95E-08	13	9	1.2	c.2.1.4
1nm5_1	16	0.625	13	2.65E-14	2.66E-14	0.625	13	2.65E-14	2.66E-14	13	9	0.5	c.2.1.4
1nm5_2	34	1.556	13	1.00E+00	4.64E+09	1.614	14	1.00E+00	2.10E+09	13	14	0.3	c.2.1.4
1ptj_1	33	6.269	13	1.00E+00	3.92E+45	6.269	13	1.00E+00	3.92E+45	13	15	1.1	c.2.1.4
1hzz_0	30	1.724	12	1.00E+00	3.14E+11	0.892	10	9.99E-01	7.35E+00	4	10	1.0	c.2.1.4
1hzz_1	32	4.845	12	1.00E+00	1.77E+30	4.845	12	1.00E+00	1.77E+30	12	13	0.7	c.2.1.4
1d4f_0	52	1.099	20	2.58E-09	2.58E-09	1.099	20	2.58E-09	2.58E-09	20	21	3.5	c.2.1.4
1d4f_1	51	0.923	19	4.24E-14	4.24E-14	0.923	19	4.24E-14	4.24E-14	19	20	3.6	c.2.1.4
1d4f_2	52	1.103	19	1.85E-07	1.85E-07	0.959	17	1.38E-09	1.38E-09	15	20	3.5	c.2.1.4
1d4f_3	50	1.138	18	3.46E-05	3.46E-05	0.904	16	1.00E-09	1.00E-09	13	20	3.7	c.2.1.4
1b3r_0	38	1.523	16	1.00E+00	1.20E+06	0.937	14	4.06E-06	4.06E-06	8	16	2.0	c.2.1.4
1b3r_1	39	1.485	17	1.00E+00	5.44E+04	0.876	13	7.32E-06	7.32E-06	8	17	2.1	c.2.1.4
1b3r_2	39	1.490	17	1.00E+00	6.93E+04	0.869	13	4.97E-06	4.97E-06	8	17	2.3	c.2.1.4
1b3r_3	38	1.479	17	1.00E+00	3.75E+04	0.923	14	1.83E-06	1.83E-06	8	16	3.5	c.2.1.4
1k0u_0	54	0.928	19	7.77E-14	7.77E-14	0.928	19	7.77E-14	7.77E-14	19	20	3.3	c.2.1.4
1k0u_1	53	1.124	20	1.84E-08	1.84E-08	1.014	18	1.07E-09	1.07E-09	13	20	3.9	c.2.1.4
1k0u_2	53	0.971	18	2.08E-10	2.08E-10	0.971	18	2.08E-10	2.08E-10	18	20	3.3	c.2.1.4
1k0u_3	53	0.944	20	6.99E-15	6.98E-15	0.944	20	6.99E-15	6.98E-15	20	21	3.6	c.2.1.4
1k0u_4	52	1.035	18	6.45E-08	6.45E-08	1.100	19	1.97E-08	1.97E-08	15	21	3.1	c.2.1.4
1k0u_5	55	1.333	18	8.88E-01	2.19E+00	1.114	18	1.14E-06	1.14E-06	12	21	3.4	c.2.1.4
1k0u_6	56	0.808	19	0.00E+00	1.38E-18	0.808	19	0.00E+00	1.38E-18	19	20	3.0	c.2.1.4
1k0u_7	55	1.171	17	5.91E-03	5.92E-03	1.041	18	8.15E-09	8.15E-09	12	21	3.4	c.2.1.4
1d4g_0	52	0.873	18	3.20E-14	3.20E-14	0.873	18	3.20E-14	3.20E-14	18	21	2.8	c.2.1.4
1d4g_1	54	0.969	19	2.45E-12	2.45E-12	0.969	19	2.45E-12	2.45E-12	19	21	3.0	c.2.1.4
1d4g_2	52	1.172	17	5.29E-03	5.31E-03	1.093	18	2.39E-07	2.39E-07	12	21	3.8	c.2.1.4
1d4g_3	54	1.011	18	4.91E-09	4.91E-09	1.011	18	4.91E-09	4.91E-09	18	21	3.6	c.2.1.4
1d4g_4	52	1.081	17	5.98E-06	5.98E-06	1.127	19	1.30E-07	1.30E-07	13	21	3.0	c.2.1.4
1d4g_5	55	1.003	18	2.82E-09	2.82E-09	1.003	18	2.82E-09	2.82E-09	18	20	3.9	c.2.1.4
1d4g_6	54	1.155	18	4.52E-05	4.52E-05	1.017	16	2.68E-06	2.68E-06	13	20	5.2	c.2.1.4
1d4g_7	53	1.191	18	4.25E-04	4.25E-04	1.061	18	2.91E-08	2.91E-08	14	20	3.1	c.2.1.4
1ky4_0	35	1.488	17	1.00E+00	4.50E+04	0.854	13	1.54E-06	1.54E-06	8	16	2.0	c.2.1.4
1ky4_1	36	1.497	17	1.00E+00	7.58E+04	0.859	13	2.22E-06	2.22E-06	8	16	2.3	c.2.1.4
1ky4_2	35	1.491	17	1.00E+00	5.21E+04	0.860	13	2.16E-06	2.16E-06	8	16	2.2	c.2.1.4
1ky4_3	35	1.493	17	1.00E+00	5.73E+04	0.852	13	1.37E-06	1.37E-06	8	16	2.0	c.2.1.4
1pjs_4	21	1.690	10	1.00E+00	2.10E+11	1.797	11	1.00E+00	1.87E+11	10	11	0.3	c.2.1.11
1pjs_6	23	2.647	11	1.00E+00	5.25E+19	2.647	11	1.00E+00	5.25E+19	11	13	0.5	c.2.1.11

Table 10: Results for alcohol dehydrogenase matching against its own SCOP superfamily (but not family) without amino acid property.

Site	N	RMSD	q	Pvalue	Evalue	RMSD	q	Pvalue	Evalue	CG	Mean L	Var L	SCOP
1kyq_18	20	0.621	10	1.70E-06	1.70E-06	0.621	10	1.70E-06	1.70E-06	10	8	0.5	c.2.1.11
1kyq_19	22	0.711	11	1.67E-06	1.67E-06	0.711	11	1.67E-06	1.67E-06	11	9	0.9	c.2.1.11
1kyq_20	23	0.720	11	3.19E-06	3.19E-06	0.720	11	3.19E-06	3.19E-06	11	9	1.1	c.2.1.11
4mdh_0	35	4.306	12	1.00E+00	4.70E+29	0.606	10	3.43E-06	3.43E-06	4	13	2.5	c.2.1.5
4mdh_1	31	0.803	13	1.16E-07	1.16E-07	0.803	13	1.16E-07	1.16E-07	13	12	1.5	c.2.1.5
5mdh_0	35	1.627	12	1.00E+00	4.87E+09	0.503	9	2.95E-06	2.95E-06	9	13	2.5	c.2.1.5
5mdh_1	37	1.024	13	6.31E-02	6.52E-02	0.680	11	1.46E-06	1.46E-06	9	13	1.8	c.2.1.5
1civ_0	32	1.147	13	9.95E-01	5.21E+00	1.131	14	5.12E-02	5.26E-02	13	14	0.2	c.2.1.5
1emd_0	67	1.280	17	8.27E-01	1.76E+00	0.748	12	3.11E-06	3.11E-06	11	20	4.7	c.2.1.5
1ib6_0	34	0.992	14	1.33E-04	1.33E-04	0.992	14	1.33E-04	1.33E-04	14	15	3.0	c.2.1.5
1ib6_1	36	0.960	14	5.44E-05	5.44E-05	0.960	14	5.44E-05	5.44E-05	14	16	3.3	c.2.1.5
1ib6_2	35	1.426	14	1.00E+00	7.14E+04	0.939	11	4.12E-01	5.32E-01	9	15	2.6	c.2.1.5
1ie3_0	30	0.771	14	8.33E-11	8.33E-11	0.771	14	8.33E-11	8.33E-11	14	14	1.6	c.2.1.5
1ie3_1	30	0.834	13	4.80E-07	4.80E-07	0.834	13	4.80E-07	4.80E-07	13	14	1.8	c.2.1.5
1ie3_2	31	0.807	16	1.44E-13	1.44E-13	0.807	16	1.44E-13	1.44E-13	16	14	1.7	c.2.1.5
1ie3_3	32	0.866	14	6.17E-08	6.17E-08	0.866	14	6.17E-08	6.17E-08	14	15	2.4	c.2.1.5
1bmd_0	27	0.987	12	2.31E-01	2.62E-01	1.014	12	1.67E-01	1.82E-01	9	13	1.4	c.2.1.5
1bmd_1	26	0.851	11	1.07E-02	1.07E-02	0.851	11	1.07E-02	1.07E-02	11	13	1.3	c.2.1.5
1o6z_1	120	5.505	14	1.00E+00	4.68E+40	5.505	14	1.00E+00	4.68E+40	14	19	2.4	c.2.1.5
1o6z_3	62	0.729	14	2.93E-11	2.93E-11	0.717	15	7.61E-14	7.61E-14	14	15	0.6	c.2.1.5
1hlp_0	31	0.715	13	2.33E-10	2.33E-10	0.715	13	2.33E-10	2.33E-10	13	14	2.0	c.2.1.5
1hlp_1	30	0.740	13	1.47E-09	1.47E-09	0.740	13	1.47E-09	1.47E-09	13	14	2.1	c.2.1.5
1gt2_0	106	1.142	15	1.92E-01	2.13E-01	1.142	15	1.92E-01	2.13E-01	15	17	1.8	c.2.1.5
1gt2_3	102	1.265	15	1.00E+00	2.61E+02	1.066	14	1.83E-01	2.02E-01	8	18	6.3	c.2.1.5
1b8u_0	38	0.927	13	3.92E-04	3.92E-04	0.523	9	1.35E-05	1.35E-05	9	13	3.0	c.2.1.5
1uxj_0	120	1.101	18	5.05E-06	5.05E-06	0.924	18	5.38E-11	5.38E-11	14	21	2.8	c.2.1.5
1uxk_0	120	1.097	17	7.79E-05	7.79E-05	0.968	18	1.97E-09	1.97E-09	14	20	3.0	c.2.1.5
1uxk_1	84	1.044	18	1.48E-08	1.48E-08	1.023	19	7.12E-10	7.12E-10	14	20	3.2	c.2.1.5
1ur5_0	120	0.918	18	9.16E-12	9.16E-12	0.918	18	9.16E-12	9.16E-12	18	20	3.0	c.2.1.5
1uxg_0	119	1.022	17	6.18E-07	6.18E-07	1.022	17	6.18E-07	6.18E-07	17	23	4.8	c.2.1.5
1uxg_1	40	0.842	16	8.02E-12	8.02E-12	0.842	16	8.02E-12	8.02E-12	16	18	3.2	c.2.1.5
1guy_0	117	1.698	16	1.00E+00	8.30E+09	0.974	18	1.36E-09	1.36E-09	12	19	2.0	c.2.1.5
1guy_2	108	1.295	15	1.00E+00	1.27E+03	0.847	16	1.51E-10	1.51E-10	12	17	2.2	c.2.1.5
1uxh_0	117	1.069	18	5.59E-07	5.59E-07	1.069	18	5.59E-07	5.59E-07	18	23	3.5	c.2.1.5
1uxh_1	40	0.849	16	1.40E-11	1.40E-11	0.849	16	1.40E-11	1.40E-11	16	18	3.1	c.2.1.5
1uxi_0	118	1.046	18	1.18E-07	1.18E-07	1.046	18	1.18E-07	1.18E-07	18	24	5.2	c.2.1.5
1uxi_1	41	0.882	17	3.37E-12	3.37E-12	0.882	17	3.37E-12	3.37E-12	17	19	3.1	c.2.1.5
1gv0_0	99	1.434	19	1.00E+00	1.27E+02	1.059	17	2.56E-05	2.56E-05	13	20	3.8	c.2.1.5
1gv0_1	95	1.772	17	1.00E+00	5.38E+09	1.772	17	1.00E+00	5.38E+09	17	19	1.4	c.2.1.5
1guz_0	33	1.111	17	2.80E-05	2.80E-05	0.892	15	1.15E-08	1.15E-08	12	16	4.0	c.2.1.5
1guz_1	32	0.938	17	1.31E-10	1.31E-10	0.938	17	1.31E-10	1.31E-10	17	16	2.2	c.2.1.5
1guz_2	33	1.102	17	1.56E-05	1.56E-05	0.887	15	8.10E-09	8.10E-09	12	16	2.2	c.2.1.5
1guz_3	32	0.905	17	9.99E-12	9.99E-12	0.905	17	9.99E-12	9.99E-12	17	16	2.0	c.2.1.5
1ojs_0	97	0.720	18	0.00E+00	8.41E-20	0.720	18	0.00E+00	8.41E-20	18	19	3.0	c.2.1.5
1hyh_0	37	1.103	17	2.37E-05	2.37E-05	1.103	17	2.37E-05	2.37E-05	17	13	2.0	c.2.1.5
1hyh_1	38	0.886	17	2.08E-12	2.08E-12	0.886	17	2.08E-12	2.08E-12	17	12	1.5	c.2.1.5
1hyh_2	31	1.077	16	2.02E-05	2.02E-05	0.608	11	8.83E-09	8.83E-09	8	12	1.1	c.2.1.5
1hyh_3	31	1.064	16	9.16E-06	9.16E-06	0.661	11	2.57E-07	2.57E-07	8	12	1.4	c.2.1.5
9ldt_0	39	1.175	16	1.09E-01	1.15E-01	0.479	11	6.18E-13	6.18E-13	9	13	2.8	c.2.1.5
9ldt_1	38	0.682	16	0.00E+00	5.05E-18	0.682	16	0.00E+00	5.05E-18	16	13	2.4	c.2.1.5
9ldb_0	38	1.158	16	3.82E-02	3.90E-02	0.479	11	5.70E-13	5.70E-13	9	13	2.5	c.2.1.5
9ldb_1	38	0.677	16	0.00E+00	3.08E-18	0.677	16	0.00E+00	3.08E-18	16	13	2.0	c.2.1.5
1t2f_0	36	0.660	16	0.00E+00	4.75E-19	0.660	16	0.00E+00	4.75E-19	16	12	0.8	c.2.1.5
1t2f_1	36	0.675	15	4.44E-16	4.60E-16	0.675	15	4.44E-16	4.60E-16	15	13	2.8	c.2.1.5
1t2f_2	35	0.698	15	3.33E-15	3.37E-15	0.698	15	3.33E-15	3.37E-15	15	12	2.6	c.2.1.5
1t2f_3	38	0.668	16	0.00E+00	1.26E-18	0.668	16	0.00E+00	1.26E-18	16	13	1.9	c.2.1.5
1ldm_0	36	0.683	16	0.00E+00	4.71E-18	0.683	16	0.00E+00	4.71E-18	16	12	1.7	c.2.1.5
1t2d_0	40	0.911	15	1.45E-07	1.45E-07	0.911	15	1.45E-07	1.45E-07	15	15	2.8	c.2.1.5
1t24_0	41	0.909	15	1.36E-07	1.36E-07	0.909	15	1.36E-07	1.36E-07	15	15	2.5	c.2.1.5
1ldg_0	41	0.919	15	2.73E-07	2.73E-07	0.919	15	2.73E-07	2.73E-07	15	15	3.4	c.2.1.5
1oc4_0	95	1.315	16	1.00E+00	2.69E+02	1.353	18	8.70E-01	2.04E+00	16	19	0.9	c.2.1.5
1oc4_1	40	0.919	14	1.19E-05	1.19E-05	0.919	14	1.19E-05	1.19E-05	14	15	4.0	c.2.1.5
1pzh_0	39	1.211	16	2.43E-01	2.79E-01	0.990	13	1.31E-02	1.32E-02	11	14	2.1	c.2.1.5
1pzh_1	40	0.909	16	2.54E-09	2.54E-09	0.909	16	2.54E-09	2.54E-09	16	15	1.8	c.2.1.5
1pzh_2	40	1.006	16	1.21E-06	1.21E-06	1.006	16	1.21E-06	1.21E-06	16	15	2.1	c.2.1.5

Table 11: Results for alcohol dehydrogenase matching against its own SCOP superfamily (but not family) without amino acid property.

Site	N	RMSD	q	Pvalue	Evalue	RMSD	q	Pvalue	Evalue	CG	Mean L	Var L	SCOP
1pzh_3	40	0.941	16	2.76E-08	2.76E-08	0.941	16	2.76E-08	2.76E-08	16	15	2.0	c.2.1.5
1ldn_0	38	0.888	16	4.32E-10	4.32E-10	0.888	16	4.32E-10	4.32E-10	16	13	1.8	c.2.1.5
1ldn_1	37	0.876	16	8.94E-11	8.94E-11	0.876	16	8.94E-11	8.94E-11	16	13	1.3	c.2.1.5
1ldn_2	40	0.996	16	1.39E-06	1.39E-06	0.958	18	3.12E-11	3.12E-11	16	18	0.4	c.2.1.5
1ldn_3	39	0.839	16	5.84E-12	5.84E-12	0.839	16	5.84E-12	5.84E-12	16	13	2.3	c.2.1.5
1ldn_4	37	0.807	17	4.11E-15	4.11E-15	0.807	17	4.11E-15	4.11E-15	17	12	1.6	c.2.1.5
1ldn_5	38	1.112	15	1.54E-02	1.56E-02	0.715	11	1.33E-05	1.33E-05	10	13	1.9	c.2.1.5
1ldn_6	37	0.750	16	2.66E-15	2.71E-15	0.750	16	2.66E-15	2.71E-15	16	13	2.0	c.2.1.5
1ldn_7	36	0.809	16	3.98E-13	3.98E-13	0.809	16	3.98E-13	3.98E-13	16	12	1.5	c.2.1.5
2ldb_0	115	0.774	16	2.84E-13	2.84E-13	0.784	17	6.00E-15	6.04E-15	16	17	0.3	c.2.1.5
1ez4_0	31	0.663	16	0.00E+00	4.05E-19	0.663	16	0.00E+00	4.05E-19	16	12	0.7	c.2.1.5
1ez4_1	32	0.629	16	0.00E+00	1.31E-20	0.629	16	0.00E+00	1.31E-20	16	11	1.7	c.2.1.5
1ez4_2	31	0.680	16	0.00E+00	2.21E-18	0.680	16	0.00E+00	2.21E-18	16	12	0.9	c.2.1.5
1ez4_3	30	0.494	14	0.00E+00	1.01E-21	0.494	14	0.00E+00	1.01E-21	14	11	0.9	c.2.1.5
1lld_0	77	0.843	18	4.77E-15	4.80E-15	0.843	18	4.77E-15	4.80E-15	18	16	3.5	c.2.1.5
1lth_0	84	0.850	19	1.11E-16	1.65E-16	0.850	19	1.11E-16	1.65E-16	19	18	4.0	c.2.1.5
1lth_2	119	1.189	17	5.62E-03	5.63E-03	1.189	17	5.62E-03	5.63E-03	17	21	7.2	c.2.1.5
1a5z_0	31	0.786	15	2.63E-12	2.63E-12	0.786	15	2.63E-12	2.63E-12	15	10	1.4	c.2.1.5
1hye_0	30	0.868	12	3.79E-04	3.79E-04	0.887	13	1.06E-05	1.06E-05	12	13	0.3	c.2.1.5
1hyg_0	32	0.653	13	2.71E-12	2.71E-12	0.653	13	2.71E-12	2.71E-12	13	13	1.2	c.2.1.5
1hyg_1	32	2.122	13	1.00E+00	1.36E+14	0.712	9	1.64E-01	1.79E-01	7	11	1.8	c.2.1.5
1obb_0	120	5.233	13	1.00E+00	5.95E+33	5.233	13	1.00E+00	5.95E+33	13	17	2.6	c.2.1.5
1obb_1	105	1.163	14	1.00E+00	8.23E+01	1.163	14	1.00E+00	8.23E+01	14	15	0.6	c.2.1.5
1vjt_0	40	1.447	12	1.00E+00	1.36E+08	1.447	12	1.00E+00	1.36E+08	12	12	0.1	c.2.1.5
1u8x_1	47	1.418	15	1.00E+00	2.22E+06	1.418	15	1.00E+00	2.22E+06	15	15	0.2	c.2.1.5
1up6_0	102	1.531	13	1.00E+00	1.90E+09	1.531	13	1.00E+00	1.90E+09	13	13	0.4	c.2.1.5
1up6_1	112	2.045	14	1.00E+00	1.19E+13	2.045	14	1.00E+00	1.19E+13	14	16	1.0	c.2.1.5
1up6_9	104	1.869	13	1.00E+00	5.32E+14	1.869	13	1.00E+00	5.32E+14	13	14	0.8	c.2.1.5
1up6_11	41	1.711	14	1.00E+00	1.18E+11	1.711	14	1.00E+00	1.18E+11	14	14	0.4	c.2.1.5
1up6_13	39	1.304	13	1.00E+00	8.94E+04	1.150	11	1.00E+00	7.12E+04	8	15	3.7	c.2.1.5
1up6_14	33	1.525	12	1.00E+00	5.28E+09	1.525	12	1.00E+00	5.28E+09	12	14	1.4	c.2.1.5
1yve_0	41	1.728	15	1.00E+00	1.50E+10	1.168	12	1.00E+00	1.24E+03	8	18	4.9	c.2.1.6
1yve_2	42	1.508	14	1.00E+00	1.41E+07	0.884	9	1.00E+00	4.68E+02	8	18	5.3	c.2.1.6
1yve_3	41	1.510	14	1.00E+00	1.41E+07	1.053	10	1.00E+00	5.32E+03	8	19	5.1	c.2.1.6
1yve_4	42	1.578	15	1.00E+00	5.76E+07	1.090	10	1.00E+00	2.09E+04	8	19	5.0	c.2.1.6
1pgo_0	24	1.133	13	1.00E+00	9.71E+00	0.679	8	9.67E-01	3.41E+00	8	10	1.4	c.2.1.6
1j3v_0	40	1.159	16	1.58E-02	1.59E-02	1.159	16	1.58E-02	1.59E-02	16	16	2.9	c.2.1.6
1j3v_2	40	1.110	16	3.15E-04	3.15E-04	1.110	16	3.15E-04	3.15E-04	16	16	2.9	c.2.1.6
1j3v_4	40	0.864	15	1.70E-09	1.70E-09	0.864	15	1.70E-09	1.70E-09	15	16	2.8	c.2.1.6
1lj8_8	27	1.139	11	1.00E+00	1.69E+03	1.104	9	1.00E+00	5.90E+04	6	11	1.8	c.2.1.6
1m2w_4	35	3.485	11	1.00E+00	3.85E+25	3.485	11	1.00E+00	3.85E+25	11	13	0.6	c.2.1.6
1m2w_5	34	3.480	11	1.00E+00	3.31E+25	3.480	11	1.00E+00	3.31E+25	11	13	0.6	c.2.1.6
1f0y_0	50	1.328	14	1.00E+00	4.22E+03	0.671	11	2.10E-06	2.10E-06	9	14	2.8	c.2.1.6
1f0y_1	50	1.527	15	1.00E+00	2.42E+06	0.439	7	2.89E-01	3.41E-01	7	14	4.5	c.2.1.6
3had_0	37	1.260	15	1.00E+00	9.30E+00	1.260	15	1.00E+00	9.30E+00	15	15	0.1	c.2.1.6
3had_1	28	1.269	15	9.98E-01	6.01E+00	0.713	10	7.53E-04	7.53E-04	9	11	1.1	c.2.1.6
1m76_0	49	1.544	15	1.00E+00	4.43E+06	0.650	11	5.13E-07	5.13E-07	9	14	3.1	c.2.1.6
1m76_1	50	1.526	15	1.00E+00	2.33E+06	0.495	10	5.29E-09	5.29E-09	9	14	3.7	c.2.1.6
2hdh_0	32	1.229	14	1.00E+00	1.49E+01	0.451	8	1.01E-04	1.01E-04	8	12	3.9	c.2.1.6
2hdh_1	33	1.227	14	1.00E+00	1.50E+01	0.480	8	6.20E-04	6.20E-04	8	12	4.4	c.2.1.6
1il0_0	50	1.515	15	1.00E+00	1.51E+06	0.679	11	3.47E-06	3.47E-06	9	13	2.8	c.2.1.6
1il0_1	49	1.558	15	1.00E+00	7.62E+06	0.575	10	1.35E-06	1.35E-06	9	15	4.0	c.2.1.6
1m75_0	51	1.543	15	1.00E+00	4.82E+06	0.557	10	4.65E-07	4.65E-07	9	13	3.7	c.2.1.6
1m75_1	51	1.529	15	1.00E+00	2.79E+06	0.654	11	7.52E-07	7.52E-07	9	14	3.0	c.2.1.6
1lsj_0	25	1.194	14	7.55E-01	1.40E+00	0.810	10	6.06E-02	6.25E-02	9	11	0.9	c.2.1.6
1lsj_1	27	1.090	14	1.17E-02	1.17E-02	0.460	9	7.22E-08	7.22E-08	9	11	1.1	c.2.1.6
1lso_0	28	1.095	14	1.68E-02	1.69E-02	0.542	9	1.67E-05	1.67E-05	9	11	1.4	c.2.1.6
1lso_1	27	1.261	14	1.00E+00	3.63E+01	0.644	10	1.49E-05	1.49E-05	9	11	1.3	c.2.1.6
3hdh_0	28	1.303	14	1.00E+00	2.47E+02	0.473	9	2.00E-07	2.00E-07	9	11	1.9	c.2.1.6
3hdh_1	25	1.477	15	1.00E+00	3.85E+04	0.553	9	2.25E-05	2.25E-05	9	10	1.1	c.2.1.6
3hdh_2	26	1.301	14	1.00E+00	1.80E+02	0.520	9	3.45E-06	3.45E-06	9	11	2.5	c.2.1.6
1i36_0	28	1.657	12	1.00E+00	3.60E+10	1.657	12	1.00E+00	3.60E+10	12	13	0.5	c.2.1.6
1i36_1	27	2.608	11	1.00E+00	2.91E+18	2.608	11	1.00E+00	2.91E+18	11	11	0.1	c.2.1.6
1dli_0	39	1.190	15	6.78E-01	1.13E+00	0.726	14	8.13E-12	8.13E-12	11	15	1.7	c.2.1.6
1dli_1	52	1.247	17	3.69E-01	4.60E-01	0.726	14	1.97E-11	1.97E-11	10	16	2.9	c.2.1.6

Table 12: Results for alcohol dehydrogenase matching against its own SCOP superfamily (but not family) without amino acid property.

Site	N	RMSD	q	Pvalue	Evalue	RMSD	q	Pvalue	Evalue	CG	Mean L	Var L	SCOP
1mv8_3	30	0.545	15	0.00E+00	4.48E-22	0.545	15	0.00E+00	4.48E-22	15	12	2.0	c.2.1.6
1mv8_4	30	0.544	15	0.00E+00	3.99E-22	0.544	15	0.00E+00	3.99E-22	15	12	1.7	c.2.1.6
1mv8_5	30	0.693	15	1.33E-15	1.34E-15	0.693	15	1.33E-15	1.34E-15	15	12	2.2	c.2.1.6
1mv8_6	30	0.517	15	0.00E+00	1.69E-23	0.517	15	0.00E+00	1.69E-23	15	12	1.3	c.2.1.6
1muu_31	30	0.454	15	0.00E+00	5.32E-27	0.454	15	0.00E+00	5.32E-27	15	12	1.2	c.2.1.6
1muu_32	30	0.568	15	0.00E+00	5.82E-21	0.568	15	0.00E+00	5.82E-21	15	12	1.0	c.2.1.6
1muu_33	30	0.555	15	0.00E+00	1.38E-21	0.555	15	0.00E+00	1.38E-21	15	11	0.3	c.2.1.6
1muu_34	30	0.552	15	0.00E+00	9.88E-22	0.552	15	0.00E+00	9.88E-22	15	12	2.7	c.2.1.6
1jay_0	39	5.328	13	1.00E+00	1.91E+36	0.942	11	8.17E-01	1.70E+00	3	14	2.3	c.2.1.6
1jay_1	37	4.787	13	1.00E+00	3.15E+35	0.962	11	9.70E-01	3.51E+00	2	14	2.6	c.2.1.6
1wdk_3	28	1.174	13	1.00E+00	1.08E+02	1.210	14	1.00E+00	1.38E+01	13	15	0.5	c.2.1.6
1wdk_6	28	1.170	13	1.00E+00	8.98E+01	0.889	12	9.47E-04	9.47E-04	9	13	1.2	c.2.1.6
1wdm_4	28	1.096	13	9.26E-01	2.60E+00	0.906	12	2.32E-03	2.32E-03	10	13	1.4	c.2.1.6
1wdm_5	28	1.097	13	9.35E-01	2.73E+00	0.915	12	3.69E-03	3.70E-03	10	13	1.3	c.2.1.6
1wdl_2	25	1.154	14	8.58E-01	1.95E+00	0.692	10	1.73E-04	1.73E-04	9	11	2.0	c.2.1.6
1wdl_6	29	1.160	14	9.85E-01	4.22E+00	0.873	12	4.47E-04	4.48E-04	11	13	1.3	c.2.1.6
1hwz_0	42	1.075	14	2.12E-02	2.15E-02	1.075	14	2.12E-02	2.15E-02	14	15	0.5	c.2.1.7
1hwz_1	42	1.075	14	2.12E-02	2.15E-02	1.075	14	2.12E-02	2.15E-02	14	15	0.5	c.2.1.7
1hwz_2	42	1.382	15	1.00E+00	3.54E+03	1.382	15	1.00E+00	3.54E+03	15	16	0.6	c.2.1.7
1hwz_3	42	1.382	15	1.00E+00	3.54E+03	0.355	6	1.00E+00	8.35E+00	6	14	6.3	c.2.1.7
1hwz_4	42	1.382	15	1.00E+00	3.54E+03	1.382	15	1.00E+00	3.54E+03	15	16	0.6	c.2.1.7
1hwz_5	42	1.074	14	2.02E-02	2.04E-02	1.074	14	2.02E-02	2.04E-02	14	15	0.5	c.2.1.7
1hwy_7	38	1.797	14	1.00E+00	4.34E+09	1.797	14	1.00E+00	4.34E+09	14	15	0.6	c.2.1.7
1hwy_8	38	1.361	14	1.00E+00	2.95E+04	1.380	15	1.00E+00	2.39E+03	14	15	0.4	c.2.1.7
1hwy_9	38	1.361	14	1.00E+00	2.95E+04	1.380	15	1.00E+00	2.39E+03	14	15	0.4	c.2.1.7
1hwy_10	38	1.361	14	1.00E+00	2.95E+04	1.380	15	1.00E+00	2.39E+03	14	15	0.3	c.2.1.7
1hwy_11	38	1.797	14	1.00E+00	4.34E+09	1.797	14	1.00E+00	4.34E+09	14	15	0.6	c.2.1.7
1hwy_12	38	1.797	14	1.00E+00	4.34E+09	1.797	14	1.00E+00	4.34E+09	14	15	0.6	c.2.1.7
1hwy_13	33	1.952	12	1.00E+00	1.66E+15	1.952	12	1.00E+00	1.66E+15	12	13	1.0	c.2.1.7
1hwy_14	33	1.832	12	1.00E+00	6.42E+13	1.832	12	1.00E+00	6.42E+13	12	12	0.2	c.2.1.7
1hwy_15	33	1.976	12	1.00E+00	3.11E+15	1.976	12	1.00E+00	3.11E+15	12	13	1.0	c.2.1.7
1hwy_16	33	1.842	12	1.00E+00	8.48E+13	1.842	12	1.00E+00	8.48E+13	12	12	0.2	c.2.1.7
1hwy_17	33	1.975	13	1.00E+00	2.48E+16	1.975	13	1.00E+00	2.48E+16	13	13	0.2	c.2.1.7
1hwy_18	33	1.841	12	1.00E+00	8.25E+13	1.841	12	1.00E+00	8.25E+13	12	12	0.2	c.2.1.7
1c1d_0	43	1.285	18	2.19E-01	2.47E-01	1.081	15	1.49E-03	1.49E-03	12	17	2.7	c.2.1.7
1c1d_1	42	1.095	17	2.08E-05	2.08E-05	1.095	17	2.08E-05	2.08E-05	17	18	3.3	c.2.1.7
1c1x_0	42	1.060	17	2.01E-06	2.01E-06	1.060	17	2.01E-06	2.01E-06	17	17	2.9	c.2.1.7
1c1x_7	29	0.965	16	2.75E-08	2.75E-08	0.965	16	2.75E-08	2.75E-08	16	15	1.9	c.2.1.7
1bw9_0	45	1.343	18	1.00E+00	8.46E+00	0.938	13	3.07E-04	3.07E-04	11	18	3.2	c.2.1.7
1bw9_1	16	0.570	12	1.21E-13	1.21E-13	0.570	12	1.21E-13	1.21E-13	12	10	0.3	c.2.1.7
1bxg_0	42	1.001	16	1.01E-06	1.01E-06	1.001	16	1.01E-06	1.01E-06	16	18	3.4	c.2.1.7
1bxg_1	44	1.187	18	1.87E-04	1.87E-04	1.187	18	1.87E-04	1.87E-04	18	18	3.1	c.2.1.7
1a4i_0	26	0.843	14	7.33E-09	7.33E-09	0.843	14	7.33E-09	7.33E-09	14	13	3.4	c.2.1.7
1a4i_1	26	0.777	13	8.79E-09	8.79E-09	0.777	13	8.79E-09	8.79E-09	13	13	3.0	c.2.1.7
1dig_0	37	1.003	13	2.19E-02	2.21E-02	1.003	13	2.19E-02	2.21E-02	13	14	1.0	c.2.1.7
1dig_2	25	1.141	11	1.00E+00	1.25E+04	1.171	12	1.00E+00	9.39E+02	11	13	0.3	c.2.1.7
1dia_0	37	0.835	14	1.29E-08	1.29E-08	0.835	14	1.29E-08	1.29E-08	14	14	1.6	c.2.1.7
1dia_1	26	0.787	12	1.68E-06	1.68E-06	0.787	12	1.68E-06	1.68E-06	12	12	3.2	c.2.1.7
1dib_0	42	1.161	16	6.77E-03	6.79E-03	0.782	12	7.72E-06	7.72E-06	11	15	3.2	c.2.1.7
1dib_1	27	0.755	13	2.34E-09	2.34E-09	0.755	13	2.34E-09	2.34E-09	13	13	1.7	c.2.1.7
1ee9_0	23	1.156	13	1.00E+00	2.32E+02	1.156	13	1.00E+00	2.32E+02	13	13	0.2	c.2.1.7
1lua_0	26	1.002	15	1.44E-06	1.44E-06	1.002	15	1.44E-06	1.44E-06	15	10	3.3	c.2.1.7
1lua_1	25	1.345	13	1.00E+00	7.17E+03	0.734	8	1.00E+00	3.87E+01	7	9	2.9	c.2.1.7
1lua_2	25	1.315	14	1.00E+00	2.88E+02	1.315	14	1.00E+00	2.88E+02	14	15	0.3	c.2.1.7
1vi2_4	42	2.381	13	1.00E+00	1.31E+18	2.381	13	1.00E+00	1.31E+18	13	13	0.2	c.2.1.7
1vi2_8	42	2.080	11	1.00E+00	7.60E+16	2.033	12	1.00E+00	3.00E+15	11	13	0.6	c.2.1.7
1npd_3	44	3.476	13	1.00E+00	5.73E+26	3.476	13	1.00E+00	5.73E+26	13	14	0.6	c.2.1.7
1npd_5	43	2.436	12	1.00E+00	1.78E+18	2.436	12	1.00E+00	1.78E+18	12	12	0.3	c.2.1.7
1o9b_0	104	1.574	13	1.00E+00	8.55E+09	1.574	13	1.00E+00	8.55E+09	13	15	1.0	c.2.1.7
1o9b_1	118	1.395	14	1.00E+00	3.82E+06	1.420	15	1.00E+00	4.20E+05	14	16	0.7	c.2.1.7
1o9b_3	44	1.090	14	5.18E-02	5.32E-02	1.090	14	5.18E-02	5.32E-02	14	16	1.2	c.2.1.7
1o9b_6	36	1.200	13	1.00E+00	7.25E+01	1.200	13	1.00E+00	7.25E+01	13	14	0.6	c.2.1.7
1o9b_7	42	2.040	12	1.00E+00	4.24E+12	2.040	12	1.00E+00	4.24E+12	12	12	0.1	c.2.1.7
1nyt_0	40	1.192	14	1.00E+00	1.77E+01	0.907	13	1.47E-04	1.47E-04	11	15	2.6	c.2.1.7
1nyt_1	32	1.238	14	1.00E+00	7.74E+01	0.676	11	7.27E-07	7.27E-07	9	12	1.3	c.2.1.7

Table 13: Results for alcohol dehydrogenase matching against its own SCOP superfamily (but not family) without amino acid property.

Site	N	RMSD	q	Pvalue	Evalue	RMSD	q	Pvalue	Evalue	CG	Mean L	Var L	SCOP
1nyt_2	32	1.007	14	5.80E-04	5.80E-04	0.750	11	5.92E-05	5.92E-05	10	13	2.8	c.2.1.7
1nyt_14	32	1.288	14	1.00E+00	7.43E+02	0.904	12	3.16E-03	3.16E-03	10	13	2.0	c.2.1.7
1nvt_2	25	0.832	14	4.93E-09	4.93E-09	0.832	14	4.93E-09	4.93E-09	14	11	0.6	c.2.1.7
1nvt_3	26	1.122	13	9.24E-01	2.58E+00	1.122	13	9.24E-01	2.58E+00	13	11	1.2	c.2.1.7
1pj3_10	35	0.995	15	2.73E-05	2.73E-05	0.995	15	2.73E-05	2.73E-05	15	16	3.0	c.2.1.7
1pj3_11	36	1.007	14	1.92E-03	1.92E-03	1.069	16	5.11E-05	5.11E-05	10	16	2.0	c.2.1.7
1pj3_12	35	0.969	15	5.00E-06	5.00E-06	0.969	15	5.00E-06	5.00E-06	15	16	2.0	c.2.1.7
1pj3_13	34	1.113	15	1.16E-02	1.17E-02	1.101	16	3.09E-04	3.09E-04	15	18	1.2	c.2.1.7
1pj3_50	23	3.721	9	1.00E+00	1.35E+25	3.721	9	1.00E+00	1.35E+25	9	10	0.5	c.2.1.7
1pj3_51	23	1.963	10	1.00E+00	7.79E+15	1.963	10	1.00E+00	7.79E+15	10	10	0.3	c.2.1.7
1pj3_52	22	3.714	9	1.00E+00	1.10E+25	3.714	9	1.00E+00	1.10E+25	9	10	0.5	c.2.1.7
1pj3_53	23	4.935	9	1.00E+00	2.23E+31	4.935	9	1.00E+00	2.23E+31	9	10	0.4	c.2.1.7
1qr6_24	30	1.025	14	1.31E-03	1.31E-03	1.025	14	1.31E-03	1.31E-03	14	16	0.9	c.2.1.7
1qr6_25	16	1.471	9	1.00E+00	8.41E+09	1.471	9	1.00E+00	8.41E+09	9	9	0.1	c.2.1.7
1qr6_26	29	1.053	14	5.47E-03	5.49E-03	1.053	14	5.47E-03	5.49E-03	14	14	4.6	c.2.1.7
1qr6_27	16	1.889	9	1.00E+00	7.73E+13	1.889	9	1.00E+00	7.73E+13	9	9	0.1	c.2.1.7
1do8_10	36	0.963	14	1.37E-04	1.37E-04	1.071	16	5.79E-05	5.79E-05	10	16	1.5	c.2.1.7
1do8_11	35	0.934	15	4.74E-07	4.74E-07	0.934	15	4.74E-07	4.74E-07	15	16	1.9	c.2.1.7
1do8_12	35	0.989	14	6.06E-04	6.06E-04	1.087	16	1.43E-04	1.43E-04	10	16	1.7	c.2.1.7
1do8_13	34	1.112	14	4.33E-01	5.68E-01	1.073	16	5.50E-05	5.50E-05	9	16	2.2	c.2.1.7
1do8_50	21	1.375	10	1.00E+00	2.64E+08	1.375	10	1.00E+00	2.64E+08	10	10	0.2	c.2.1.7
1do8_51	21	7.946	9	1.00E+00	1.07E+37	7.946	9	1.00E+00	1.07E+37	9	10	0.5	c.2.1.7
1do8_52	20	1.534	9	1.00E+00	7.91E+10	1.534	9	1.00E+00	7.91E+10	9	9	0.1	c.2.1.7
1do8_53	22	1.356	10	1.00E+00	1.72E+08	1.356	10	1.00E+00	1.72E+08	10	10	0.2	c.2.1.7
1efl_10	36	1.109	15	3.06E-02	3.10E-02	1.055	16	2.11E-05	2.11E-05	12	16	2.4	c.2.1.7
1efl_11	37	0.930	14	1.89E-05	1.89E-05	1.033	16	5.60E-06	5.60E-06	10	16	3.2	c.2.1.7
1efl_12	35	1.062	14	4.00E-02	4.09E-02	1.062	15	6.94E-04	6.95E-04	10	16	2.2	c.2.1.7
1efl_13	36	0.931	14	1.85E-05	1.85E-05	1.015	16	1.59E-06	1.59E-06	10	16	1.7	c.2.1.7
1efl_50	21	2.296	9	1.00E+00	2.27E+17	2.296	9	1.00E+00	2.27E+17	9	9	0.3	c.2.1.7
1efl_51	21	2.292	9	1.00E+00	2.54E+18	2.298	10	1.00E+00	4.58E+17	9	11	0.5	c.2.1.7
1efl_52	22	2.284	9	1.00E+00	2.57E+18	2.284	9	1.00E+00	2.57E+18	9	10	0.7	c.2.1.7
1efl_53	21	2.298	9	1.00E+00	2.80E+18	2.298	9	1.00E+00	2.80E+18	9	10	0.7	c.2.1.7
1pjl_100	28	1.060	12	9.79E-01	3.88E+00	1.060	12	9.79E-01	3.88E+00	12	13	2.4	c.2.1.7
1pjl_101	15	2.270	9	1.00E+00	5.13E+16	2.270	9	1.00E+00	5.13E+16	9	9	0.1	c.2.1.7
1pjl_102	28	1.328	13	1.00E+00	2.16E+04	0.895	11	6.73E-02	6.97E-02	8	13	1.8	c.2.1.7
1pjl_103	15	1.614	9	1.00E+00	2.02E+11	1.614	9	1.00E+00	2.02E+11	9	9	0.0	c.2.1.7
1pjl_104	29	1.608	12	1.00E+00	1.56E+09	1.070	12	9.99E-01	6.75E+00	6	14	2.2	c.2.1.7
1pjl_105	15	2.453	9	1.00E+00	8.68E+17	2.453	9	1.00E+00	8.68E+17	9	9	0.4	c.2.1.7
1pjl_106	29	1.211	14	1.00E+00	1.62E+01	1.200	16	1.83E-02	1.85E-02	14	16	0.6	c.2.1.7
1pjl_107	15	2.770	9	1.00E+00	7.32E+19	2.770	9	1.00E+00	7.32E+19	9	9	0.3	c.2.1.7
1pjl_108	29	1.772	12	1.00E+00	1.09E+12	1.030	13	4.07E-02	4.16E-02	7	14	1.6	c.2.1.7
1pjl_109	15	3.292	9	1.00E+00	3.99E+22	3.292	9	1.00E+00	3.99E+22	9	9	0.1	c.2.1.7
1pjl_110	28	1.101	13	7.02E-01	1.21E+00	1.101	13	7.02E-01	1.21E+00	13	14	3.7	c.2.1.7
1pjl_111	16	1.986	9	1.00E+00	4.81E+14	1.986	9	1.00E+00	4.81E+14	9	9	0.3	c.2.1.7
1pjl_112	27	0.883	13	1.07E-05	1.07E-05	0.883	13	1.07E-05	1.07E-05	13	13	3.0	c.2.1.7
1pjl_113	16	2.689	10	1.00E+00	2.12E+21	2.689	10	1.00E+00	2.12E+21	10	10	0.0	c.2.1.7
1pjl_114	27	1.110	13	8.09E-01	1.65E+00	0.944	12	1.43E-02	1.44E-02	8	13	1.7	c.2.1.7
1pjl_115	16	2.174	9	1.00E+00	1.40E+17	2.174	9	1.00E+00	1.40E+17	9	9	0.1	c.2.1.7
1efk_10	37	1.041	15	2.39E-04	2.39E-04	1.081	16	1.17E-04	1.18E-04	7	16	1.8	c.2.1.7
1efk_11	36	1.450	14	1.00E+00	4.44E+06	1.085	16	1.38E-04	1.38E-04	7	16	1.8	c.2.1.7
1efk_12	35	0.967	15	4.38E-06	4.38E-06	0.967	15	4.38E-06	4.38E-06	15	16	1.9	c.2.1.7
1efk_13	35	1.167	15	5.26E-01	7.46E-01	1.057	16	2.20E-05	2.20E-05	9	17	2.0	c.2.1.7
1efk_50	20	2.370	9	1.00E+00	7.88E+18	2.422	10	1.00E+00	3.46E+18	9	10	0.3	c.2.1.7
1efk_51	20	1.827	9	1.00E+00	4.66E+13	1.827	9	1.00E+00	4.66E+13	9	10	0.6	c.2.1.7
1efk_52	21	3.084	9	1.00E+00	1.08E+22	3.084	9	1.00E+00	1.08E+22	9	10	0.4	c.2.1.7
1efk_53	20	2.354	9	1.00E+00	6.07E+18	2.401	10	1.00E+00	2.41E+18	9	10	0.4	c.2.1.7
1gq2_0	120	3.346	14	1.00E+00	3.21E+25	3.346	14	1.00E+00	3.21E+25	14	17	1.9	c.2.1.7
1gq2_1	120	1.444	17	1.00E+00	4.72E+04	1.444	17	1.00E+00	4.72E+04	17	19	1.9	c.2.1.7
1gq2_4	38	1.416	15	1.00E+00	2.31E+05	1.075	14	1.03E-01	1.08E-01	9	15	1.9	c.2.1.7
1gq2_6	120	2.088	13	1.00E+00	3.33E+17	2.088	13	1.00E+00	3.33E+17	13	16	2.4	c.2.1.7
1gq2_7	120	1.715	15	1.00E+00	5.57E+09	1.715	15	1.00E+00	5.57E+09	15	16	1.1	c.2.1.7
1gq2_10	38	1.386	14	1.00E+00	3.63E+05	1.084	14	1.62E-01	1.77E-01	7	15	2.2	c.2.1.7
1gq2_12	118	1.646	13	1.00E+00	2.11E+10	1.722	14	1.00E+00	1.42E+10	13	15	1.0	c.2.1.7
1gq2_13	120	1.514	16	1.00E+00	5.18E+06	1.514	16	1.00E+00	5.18E+06	16	19	2.4	c.2.1.7
1gq2_17	36	1.411	14	1.00E+00	8.85E+05	1.411	14	1.00E+00	8.85E+05	14	16	1.5	c.2.1.7

Table 14: Results for alcohol dehydrogenase matching against its own SCOP superfamily (but not family) without amino acid property.

Site	N	RMSD	q	Pvalue	Evalue	RMSD	q	Pvalue	Evalue	CG	Mean L	Var L	SCOP
1gq2_19	120	2.038	14	1.00E+00	1.03E+16	2.003	16	1.00E+00	4.54E+13	14	16	0.5	c.2.1.7
1gq2_20	120	1.533	15	1.00E+00	4.39E+07	1.533	15	1.00E+00	4.39E+07	15	18	2.2	c.2.1.7
1gq2_23	37	1.403	15	1.00E+00	2.65E+04	1.403	15	1.00E+00	2.65E+04	15	17	1.5	c.2.1.7
1gq2_25	120	9.772	14	1.00E+00	3.82E+59	9.772	14	1.00E+00	3.82E+59	14	19	2.8	c.2.1.7
1gq2_26	120	1.492	16	1.00E+00	2.00E+06	1.442	17	1.00E+00	4.29E+04	16	19	2.3	c.2.1.7
1gq2_29	39	1.132	15	4.97E-02	5.10E-02	0.890	13	9.09E-05	9.09E-05	9	14	2.2	c.2.1.7
1gq2_31	120	7.643	15	1.00E+00	3.59E+49	7.643	15	1.00E+00	3.59E+49	15	19	2.7	c.2.1.7
1gq2_32	120	1.451	16	1.00E+00	1.44E+04	1.451	16	1.00E+00	1.44E+04	16	19	1.9	c.2.1.7
1gq2_36	39	1.431	16	1.00E+00	9.30E+04	0.928	13	8.77E-04	8.77E-04	8	14	2.9	c.2.1.7
1gq2_38	120	1.504	14	1.00E+00	1.13E+07	1.504	14	1.00E+00	1.13E+07	14	16	1.2	c.2.1.7
1gq2_39	120	1.492	16	1.00E+00	3.97E+05	1.208	16	1.00E+00	2.40E+01	7	20	7.4	c.2.1.7
1gq2_42	37	1.007	14	2.09E-03	2.09E-03	1.007	14	2.09E-03	2.09E-03	14	15	2.0	c.2.1.7
1gq2_44	120	1.749	15	1.00E+00	5.80E+12	1.765	17	1.00E+00	5.89E+10	15	18	1.2	c.2.1.7
1gq2_45	120	1.503	16	1.00E+00	3.23E+06	1.503	16	1.00E+00	3.23E+06	16	18	1.6	c.2.1.7
1gq2_49	39	1.432	15	1.00E+00	5.15E+05	0.869	12	1.54E-03	1.54E-03	8	14	3.2	c.2.1.7
1gq2_51	111	2.092	13	1.00E+00	2.92E+17	2.092	13	1.00E+00	2.92E+17	13	15	1.1	c.2.1.7
1gq2_52	120	1.513	15	1.00E+00	2.00E+07	1.513	15	1.00E+00	2.00E+07	15	18	2.6	c.2.1.7
1gq2_56	36	1.425	14	1.00E+00	1.59E+06	1.041	14	1.36E-02	1.37E-02	7	14	2.7	c.2.1.7
1gq2_58	120	9.379	14	1.00E+00	7.81E+53	9.379	14	1.00E+00	7.81E+53	14	18	2.4	c.2.1.7
1gq2_59	120	1.568	14	1.00E+00	1.03E+08	1.568	14	1.00E+00	1.03E+08	14	16	1.1	c.2.1.7
1gq2_62	37	1.421	15	1.00E+00	2.67E+05	0.925	13	6.25E-04	6.26E-04	8	14	1.7	c.2.1.7
1gq2_63	120	9.357	14	1.00E+00	6.83E+53	9.357	14	1.00E+00	6.83E+53	14	18	2.6	c.2.1.7
1gq2_64	120	1.508	15	1.00E+00	1.64E+07	1.451	16	1.00E+00	3.27E+05	15	17	1.6	c.2.1.7
1gq2_67	36	1.318	14	1.00E+00	1.57E+04	1.089	14	1.79E-01	1.97E-01	9	14	1.7	c.2.1.7
1gq2_68	120	3.192	14	1.00E+00	7.46E+29	3.192	14	1.00E+00	7.46E+29	14	18	2.1	c.2.1.7
1gq2_69	120	0.956	14	1.90E-04	1.90E-04	0.968	15	9.88E-06	9.88E-06	14	19	2.8	c.2.1.7
1gq2_72	37	1.107	15	1.08E-02	1.08E-02	1.107	15	1.08E-02	1.08E-02	15	14	1.9	c.2.1.7
1gq2_75	120	1.969	14	1.00E+00	1.43E+15	1.969	14	1.00E+00	1.43E+15	14	15	0.7	c.2.1.7
1gq2_76	120	1.509	15	1.00E+00	3.30E+06	1.194	16	1.00E+00	1.07E+01	7	19	5.8	c.2.1.7
1gq2_79	36	1.428	15	1.00E+00	3.36E+05	1.033	14	8.63E-03	8.67E-03	9	14	1.5	c.2.1.7
1gq2_81	120	1.700	14	1.00E+00	2.13E+12	1.700	14	1.00E+00	2.13E+12	14	15	0.4	c.2.1.7
1gq2_82	120	1.559	15	1.00E+00	2.19E+07	1.559	15	1.00E+00	2.19E+07	15	19	2.3	c.2.1.7
1gq2_86	38	1.134	15	5.11E-02	5.25E-02	1.134	15	5.11E-02	5.25E-02	15	14	1.7	c.2.1.7
1gq2_87	120	2.095	14	1.00E+00	4.94E+17	2.080	15	1.00E+00	3.93E+16	14	18	2.3	c.2.1.7
1gq2_88	120	1.598	14	1.00E+00	1.63E+09	1.598	14	1.00E+00	1.63E+09	14	18	2.1	c.2.1.7
1gq2_91	36	1.429	15	1.00E+00	7.60E+04	1.053	14	2.66E-02	2.70E-02	7	14	1.4	c.2.1.7
1gq2_94	120	7.113	14	1.00E+00	1.41E+51	7.113	14	1.00E+00	1.41E+51	14	18	2.7	c.2.1.7
1gq2_95	120	2.094	14	1.00E+00	4.86E+15	2.111	15	1.00E+00	9.66E+14	14	16	1.4	c.2.1.7
1gq2_98	37	1.000	14	1.38E-03	1.38E-03	1.000	14	1.38E-03	1.38E-03	14	14	1.9	c.2.1.7
1llq_0	29	1.253	12	1.00E+00	1.18E+04	1.253	12	1.00E+00	1.18E+04	12	14	1.1	c.2.1.7
1llq_1	28	1.104	12	1.00E+00	7.33E+01	1.104	12	1.00E+00	7.33E+01	12	13	0.5	c.2.1.7
1lss_0	25	0.744	15	6.23E-14	6.23E-14	0.744	15	6.23E-14	6.23E-14	15	10	1.6	c.2.1.9
1lss_1	25	1.020	13	3.64E-02	3.71E-02	1.020	13	3.64E-02	3.71E-02	13	9	2.1	c.2.1.9
1xcb_0	35	1.578	14	1.00E+00	1.07E+08	0.815	11	2.63E-03	2.64E-03	9	13	2.5	c.2.1.12
1xcb_1	34	0.760	13	8.72E-09	8.72E-09	0.760	13	8.72E-09	8.72E-09	13	12	1.5	c.2.1.12
1xcb_2	37	0.906	15	4.30E-08	4.30E-08	0.906	15	4.30E-08	4.30E-08	15	14	1.6	c.2.1.12
1xcb_3	35	0.879	15	5.54E-09	5.54E-09	0.879	15	5.54E-09	5.54E-09	15	13	1.4	c.2.1.12
1xcb_4	37	0.755	14	6.48E-11	6.48E-11	0.755	14	6.48E-11	6.48E-11	14	14	1.8	c.2.1.12
1xcb_5	35	0.973	14	1.08E-04	1.08E-04	0.973	14	1.08E-04	1.08E-04	14	13	1.5	c.2.1.12
1xcb_6	35	1.128	13	1.00E+00	8.57E+00	0.917	14	3.64E-06	3.64E-06	9	14	1.2	c.2.1.12
1omo_2	28	1.020	14	1.88E-03	1.88E-03	0.879	13	9.49E-06	9.49E-06	10	14	2.0	c.2.1.13
1omo_3	27	1.069	15	1.80E-04	1.80E-04	1.069	15	1.80E-04	1.80E-04	15	14	2.2	c.2.1.13

Table 15: Results for alcohol dehydrogenase matching against its own SCOP superfamily (but not family) with amino acid property.

Site	N	RMSD	q	Pvalue	Evalue	RMSD	q	Pvalue	Evalue	CG	Mean L	Var L	SCOP
1udb_0	38	5.650	7	1.00E+00	2.73E+25	5.650	7	1.00E+00	2.73E+25	7	9	1.3	c.2.1.2
1udc_0	58	2.105	8	1.00E+00	1.18E+15	2.105	8	1.00E+00	1.18E+15	8	8	0.4	c.2.1.2
1xel_0	57	2.084	8	1.00E+00	8.02E+14	2.084	8	1.00E+00	8.02E+14	8	8	0.4	c.2.1.2
1lrk_2	34	4.480	7	1.00E+00	4.02E+22	4.480	7	1.00E+00	4.02E+22	7	9	0.8	c.2.1.2
1nah_0	39	3.751	8	1.00E+00	2.10E+20	3.751	8	1.00E+00	2.10E+20	8	9	0.7	c.2.1.2
2udp_0	54	2.635	9	1.00E+00	6.42E+16	0.570	7	9.54E-01	3.09E+00	2	8	1.6	c.2.1.2
2udp_1	52	6.453	8	1.00E+00	1.90E+29	6.453	8	1.00E+00	1.90E+29	8	10	0.8	c.2.1.2
1uda_0	37	5.668	7	1.00E+00	2.74E+25	5.668	7	1.00E+00	2.74E+25	7	9	1.2	c.2.1.2
1a9y_0	53	1.625	8	1.00E+00	6.52E+10	1.625	8	1.00E+00	6.52E+10	8	8	0.1	c.2.1.2
1lrl_2	33	2.219	7	1.00E+00	9.85E+14	2.219	7	1.00E+00	9.85E+14	7	9	0.8	c.2.1.2
1kvu_0	58	2.091	8	1.00E+00	9.46E+14	2.091	8	1.00E+00	9.46E+14	8	8	0.4	c.2.1.2
1kvr_0	38	5.632	8	1.00E+00	1.75E+27	5.632	8	1.00E+00	1.75E+27	8	11	0.9	c.2.1.2
1a9z_0	33	3.928	7	1.00E+00	6.17E+20	3.928	7	1.00E+00	6.17E+20	7	9	0.8	c.2.1.2
1lrj_0	53	2.139	8	1.00E+00	1.54E+15	2.139	8	1.00E+00	1.54E+15	8	8	0.1	c.2.1.2
1nai_0	37	1.235	8	1.00E+00	9.65E+05	0.882	7	1.00E+00	4.39E+04	5	10	1.7	c.2.1.2
1kvt_0	52	3.439	8	1.00E+00	5.15E+19	3.439	8	1.00E+00	5.15E+19	8	9	0.6	c.2.1.2
1kvq_0	52	7.528	8	1.00E+00	3.17E+33	7.528	8	1.00E+00	3.17E+33	8	11	1.4	c.2.1.2
1kvs_0	52	2.249	8	1.00E+00	7.81E+15	2.249	8	1.00E+00	7.81E+15	8	8	0.4	c.2.1.2
1i3k_0	52	2.439	8	1.00E+00	9.00E+16	2.439	8	1.00E+00	9.00E+16	8	9	0.5	c.2.1.2
1i3k_1	52	2.877	8	1.00E+00	2.85E+18	2.877	8	1.00E+00	2.85E+18	8	10	0.7	c.2.1.2
1i3l_6	32	3.536	7	1.00E+00	1.06E+20	3.536	7	1.00E+00	1.06E+20	7	8	1.0	c.2.1.2
1i3l_7	33	2.195	7	1.00E+00	5.77E+13	2.195	7	1.00E+00	5.77E+13	7	7	0.1	c.2.1.2
1i3n_0	54	6.610	8	1.00E+00	4.50E+29	6.610	8	1.00E+00	4.50E+29	8	11	1.1	c.2.1.2
1i3n_6	33	3.545	7	1.00E+00	1.25E+20	3.545	7	1.00E+00	1.25E+20	7	8	0.9	c.2.1.2
1i3m_0	54	6.629	8	1.00E+00	4.93E+29	6.629	8	1.00E+00	4.93E+29	8	11	1.1	c.2.1.2
1i3m_6	32	3.544	7	1.00E+00	1.13E+20	3.544	7	1.00E+00	1.13E+20	7	8	0.9	c.2.1.2
1hzj_0	54	5.092	8	1.00E+00	9.41E+27	5.092	8	1.00E+00	9.41E+27	8	10	1.2	c.2.1.2
1hzj_1	55	5.622	8	1.00E+00	6.79E+27	5.622	8	1.00E+00	6.79E+27	8	10	1.1	c.2.1.2
1ek5_0	36	1.313	8	1.00E+00	7.25E+06	1.008	8	1.00E+00	5.20E+03	6	10	1.5	c.2.1.2
1gy8_0	120	6.429	9	1.00E+00	8.86E+32	6.429	9	1.00E+00	8.86E+32	9	13	1.9	c.2.1.2
1bxk_0	33	1.245	9	1.00E+00	4.71E+04	0.919	9	9.40E-01	2.82E+00	7	10	1.2	c.2.1.2
1bxk_1	36	1.271	9	1.00E+00	1.26E+05	0.640	8	1.86E-02	1.88E-02	7	10	1.3	c.2.1.2
1g1a_0	38	1.204	8	1.00E+00	1.17E+06	0.577	8	1.28E-03	1.28E-03	6	10	1.1	c.2.1.2
1g1a_1	37	1.210	8	1.00E+00	1.24E+06	0.570	8	8.41E-04	8.41E-04	6	10	1.2	c.2.1.2
1g1a_2	37	1.222	8	1.00E+00	1.67E+06	0.920	9	9.84E-01	4.16E+00	6	10	1.2	c.2.1.2
1g1a_3	37	1.230	8	1.00E+00	2.02E+06	0.590	8	2.17E-03	2.18E-03	6	10	1.4	c.2.1.2
1kep_0	45	1.315	8	1.00E+00	1.51E+07	0.598	7	9.95E-01	5.23E+00	6	9	1.9	c.2.1.2
1kep_1	58	1.670	8	1.00E+00	2.87E+10	1.670	8	1.00E+00	2.87E+10	8	8	0.1	c.2.1.2
1ket_2	38	1.331	8	1.00E+00	1.28E+07	1.322	9	1.00E+00	3.38E+05	8	9	0.4	c.2.1.2
1ket_3	39	1.328	8	1.00E+00	1.30E+07	1.323	9	1.00E+00	3.76E+05	8	9	0.4	c.2.1.2
1kew_2	39	1.223	8	1.00E+00	2.01E+06	0.565	8	7.76E-04	7.76E-04	6	10	2.1	c.2.1.2
1kew_3	38	1.203	8	1.00E+00	1.14E+06	0.546	8	2.79E-04	2.79E-04	6	10	1.4	c.2.1.2
1ker_0	59	2.356	8	1.00E+00	1.78E+16	2.356	8	1.00E+00	1.78E+16	8	9	0.6	c.2.1.2
1ker_1	61	2.157	8	1.00E+00	1.52E+14	2.145	9	1.00E+00	1.72E+13	8	9	0.3	c.2.1.2
1keu_0	58	3.548	8	1.00E+00	1.54E+22	0.566	8	2.75E-03	2.75E-03	0	9	1.8	c.2.1.2
1keu_1	57	3.557	8	1.00E+00	1.60E+22	0.572	8	3.48E-03	3.49E-03	0	10	1.7	c.2.1.2
1r6d_0	57	1.265	9	1.00E+00	4.37E+05	0.624	8	3.77E-02	3.84E-02	7	10	1.9	c.2.1.2
1r66_1	38	1.236	9	1.00E+00	5.67E+04	0.607	8	5.16E-03	5.17E-03	7	10	2.2	c.2.1.2
1kc1_2	27	3.952	7	1.00E+00	7.96E+19	3.952	7	1.00E+00	7.96E+19	7	8	0.7	c.2.1.2
1kc3_0	39	8.455	8	1.00E+00	1.01E+31	8.455	8	1.00E+00	1.01E+31	8	9	0.6	c.2.1.2
1e6u_0	120	2.864	9	1.00E+00	1.27E+19	2.864	9	1.00E+00	1.27E+19	9	11	0.8	c.2.1.2
1e7s_0	120	1.211	9	1.00E+00	1.14E+06	1.211	9	1.00E+00	1.14E+06	9	9	0.1	c.2.1.2
1e7q_0	120	0.942	9	1.00E+00	3.24E+02	0.942	9	1.00E+00	3.24E+02	9	9	0.3	c.2.1.2
1e7r_0	120	1.188	9	1.00E+00	6.09E+05	1.188	9	1.00E+00	6.09E+05	9	10	0.3	c.2.1.2
1bsv_0	29	2.304	7	1.00E+00	2.83E+14	2.304	7	1.00E+00	2.83E+14	7	8	0.7	c.2.1.2
1fxs_0	29	3.920	7	1.00E+00	6.17E+19	3.920	7	1.00E+00	6.17E+19	7	8	0.4	c.2.1.2
1bws_0	29	1.134	7	1.00E+00	3.50E+06	0.524	6	1.00E+00	2.08E+02	6	6	0.6	c.2.1.2
1rpn_0	40	3.089	8	1.00E+00	1.12E+20	3.089	8	1.00E+00	1.12E+20	8	9	0.4	c.2.1.2
1rpn_1	40	3.080	8	1.00E+00	1.01E+20	3.080	8	1.00E+00	1.01E+20	8	9	0.4	c.2.1.2
1rpn_2	39	1.495	8	1.00E+00	1.25E+09	1.540	9	1.00E+00	1.62E+08	8	9	0.5	c.2.1.2
1rpn_3	39	3.101	8	1.00E+00	1.18E+20	3.101	8	1.00E+00	1.18E+20	8	9	0.4	c.2.1.2
1t2a_0	40	2.552	8	1.00E+00	3.41E+15	2.552	8	1.00E+00	3.41E+15	8	9	0.6	c.2.1.2
1t2a_2	39	1.555	9	1.00E+00	2.28E+08	1.555	9	1.00E+00	2.28E+08	9	10	0.3	c.2.1.2
1t2a_4	39	1.561	9	1.00E+00	2.63E+08	1.561	9	1.00E+00	2.63E+08	9	10	0.4	c.2.1.2
1t2a_6	39	1.313	7	1.00E+00	3.74E+08	1.493	8	1.00E+00	6.82E+08	7	9	0.7	c.2.1.2

Table 16: Results for alcohol dehydrogenase matching against its own SCOP superfamily (but not family) with amino acid property.

Site	N	RMSD	q	Pvalue	Evalue	RMSD	q	Pvalue	Evalue	CG	Mean L	Var L	SCOP
1n7h_0	35	1.894	8	1.00E+00	3.07E+12	1.894	8	1.00E+00	3.07E+12	8	9	0.5	c.2.1.2
1n7h_2	35	1.896	8	1.00E+00	3.18E+12	1.896	8	1.00E+00	3.18E+12	8	9	0.5	c.2.1.2
1n7g_0	58	1.690	9	1.00E+00	4.42E+10	1.690	9	1.00E+00	4.42E+10	9	10	0.4	c.2.1.2
1n7g_1	57	1.693	9	1.00E+00	4.49E+10	1.693	9	1.00E+00	4.49E+10	9	10	0.4	c.2.1.2
1n7g_2	58	1.690	9	1.00E+00	4.42E+10	1.690	9	1.00E+00	4.42E+10	9	10	0.4	c.2.1.2
1n7g_3	37	1.885	8	1.00E+00	3.11E+12	1.885	8	1.00E+00	3.11E+12	8	9	0.5	c.2.1.2
1eq2_0	36	3.094	7	1.00E+00	3.31E+18	3.114	8	1.00E+00	1.56E+18	7	10	0.7	c.2.1.2
1eq2_1	54	4.568	7	1.00E+00	2.79E+23	4.568	7	1.00E+00	2.79E+23	7	10	0.9	c.2.1.2
1eq2_2	36	1.736	8	1.00E+00	6.21E+10	1.736	8	1.00E+00	6.21E+10	8	8	0.2	c.2.1.2
1eq2_3	54	1.708	9	1.00E+00	6.69E+09	1.718	10	1.00E+00	5.70E+08	9	10	0.3	c.2.1.2
1eq2_4	36	2.155	7	1.00E+00	9.33E+13	2.155	7	1.00E+00	9.33E+13	7	8	0.6	c.2.1.2
1eq2_5	36	1.985	7	1.00E+00	1.05E+13	1.985	7	1.00E+00	1.05E+13	7	8	0.6	c.2.1.2
1eq2_6	36	1.749	8	1.00E+00	7.86E+10	1.749	8	1.00E+00	7.86E+10	8	8	0.2	c.2.1.2
1eq2_7	36	1.981	7	1.00E+00	9.91E+12	1.981	7	1.00E+00	9.91E+12	7	8	0.6	c.2.1.2
1eq2_8	36	1.728	8	1.00E+00	5.37E+10	1.728	8	1.00E+00	5.37E+10	8	8	0.2	c.2.1.2
1eq2_9	36	4.936	7	1.00E+00	3.59E+23	4.936	7	1.00E+00	3.59E+23	7	9	1.0	c.2.1.2
1orr_0	35	2.895	8	1.00E+00	1.66E+17	2.895	8	1.00E+00	1.66E+17	8	9	0.5	c.2.1.2
1orr_2	34	1.669	8	1.00E+00	2.65E+10	1.677	9	1.00E+00	2.00E+09	8	9	0.2	c.2.1.2
1orr_4	34	1.710	8	1.00E+00	5.71E+10	1.713	9	1.00E+00	4.16E+09	8	9	0.2	c.2.1.2
1orr_6	35	2.906	8	1.00E+00	1.85E+17	0.608	7	9.70E-01	3.51E+00	4	8	1.1	c.2.1.2
1i24_0	58	2.288	7	1.00E+00	1.34E+16	2.234	8	1.00E+00	1.01E+15	7	10	1.4	c.2.1.2
1qrr_0	58	4.086	8	1.00E+00	1.18E+22	4.086	8	1.00E+00	1.18E+22	8	10	1.1	c.2.1.2
1i2c_0	58	3.151	8	1.00E+00	5.42E+18	3.151	8	1.00E+00	5.42E+18	8	10	1.0	c.2.1.2
1i2b_0	59	4.031	9	1.00E+00	6.30E+22	4.031	9	1.00E+00	6.30E+22	9	11	1.3	c.2.1.2
1k6x_0	27	2.979	7	1.00E+00	3.20E+18	2.979	7	1.00E+00	3.20E+18	7	8	0.5	c.2.1.2
1ti7_0	33	6.495	7	1.00E+00	7.13E+26	6.495	7	1.00E+00	7.13E+26	7	8	0.5	c.2.1.2
1cyd_0	40	5.082	8	1.00E+00	1.13E+27	0.623	8	9.35E-03	9.40E-03	0	10	1.8	c.2.1.2
1cyd_1	40	5.757	8	1.00E+00	9.90E+28	0.574	7	6.64E-01	1.09E+00	0	10	2.0	c.2.1.2
1cyd_2	40	5.758	8	1.00E+00	9.96E+28	5.758	8	1.00E+00	9.96E+28	8	12	1.4	c.2.1.2
1cyd_3	40	2.120	8	1.00E+00	6.22E+13	0.598	7	9.37E-01	2.76E+00	0	10	2.6	c.2.1.2
1pr9_0	41	2.673	8	1.00E+00	1.35E+17	2.644	9	1.00E+00	2.35E+16	8	9	0.3	c.2.1.2
1pr9_1	42	1.956	8	1.00E+00	7.51E+12	1.959	9	1.00E+00	8.06E+11	8	10	0.4	c.2.1.2
1oaa_0	39	7.327	8	1.00E+00	3.00E+32	7.327	8	1.00E+00	3.00E+32	8	11	1.2	c.2.1.2
1sep_0	43	1.555	9	1.00E+00	5.37E+08	1.555	9	1.00E+00	5.37E+08	9	9	0.3	c.2.1.2
1nas_0	46	1.551	9	1.00E+00	6.02E+08	1.551	9	1.00E+00	6.02E+08	9	9	0.4	c.2.1.2
1dhr_0	31	1.655	9	1.00E+00	1.24E+09	0.701	9	2.66E-04	2.66E-04	5	11	2.4	c.2.1.2
1dir_0	33	1.698	9	1.00E+00	3.70E+09	1.006	11	8.34E-03	8.38E-03	7	11	1.2	c.2.1.2
1dir_1	36	1.471	9	1.00E+00	1.62E+07	0.949	11	1.04E-03	1.04E-03	7	11	0.9	c.2.1.2
1dir_2	33	1.704	9	1.00E+00	4.17E+09	0.942	11	5.90E-04	5.91E-04	7	11	0.7	c.2.1.2
1dir_3	32	0.955	9	1.00E+00	9.06E+00	0.920	10	2.22E-02	2.25E-02	8	11	1.2	c.2.1.2
1hdr_0	30	2.136	10	1.00E+00	3.30E+12	0.721	9	5.99E-04	5.99E-04	8	11	1.8	c.2.1.2
1e7w_0	44	1.557	7	1.00E+00	9.53E+10	1.557	7	1.00E+00	9.53E+10	7	7	0.1	c.2.1.2
1e7w_1	43	0.984	8	1.00E+00	3.32E+03	0.984	8	1.00E+00	3.32E+03	8	8	0.0	c.2.1.2
1e92_0	38	6.588	8	1.00E+00	3.25E+29	6.588	8	1.00E+00	3.25E+29	8	9	0.8	c.2.1.2
1e92_1	38	1.508	7	1.00E+00	2.43E+10	1.508	7	1.00E+00	2.43E+10	7	7	0.1	c.2.1.2
1e92_2	37	5.666	7	1.00E+00	2.03E+25	5.666	7	1.00E+00	2.03E+25	7	8	0.6	c.2.1.2
1e92_3	38	2.236	8	1.00E+00	5.97E+14	2.213	9	1.00E+00	6.17E+13	8	9	0.4	c.2.1.2
1p33_0	42	1.730	8	1.00E+00	2.61E+11	1.730	8	1.00E+00	2.61E+11	8	8	0.3	c.2.1.2
1p33_1	42	1.687	7	1.00E+00	8.20E+11	1.687	7	1.00E+00	8.20E+11	7	7	0.1	c.2.1.2
1p33_2	42	1.730	7	1.00E+00	1.69E+12	1.730	7	1.00E+00	1.69E+12	7	7	0.0	c.2.1.2
1p33_3	42	2.406	8	1.00E+00	1.68E+15	2.406	8	1.00E+00	1.68E+15	8	8	0.3	c.2.1.2
1mxh_0	42	5.684	8	1.00E+00	5.61E+28	5.684	8	1.00E+00	5.61E+28	8	10	1.2	c.2.1.2
1mxh_1	42	5.662	8	1.00E+00	4.92E+28	5.662	8	1.00E+00	4.92E+28	8	10	1.1	c.2.1.2
1mxh_2	42	2.583	8	1.00E+00	3.68E+16	2.583	8	1.00E+00	3.68E+16	8	8	0.3	c.2.1.2
1mxh_3	42	2.573	8	1.00E+00	3.26E+16	2.573	8	1.00E+00	3.26E+16	8	8	0.3	c.2.1.2
1mxf_0	47	5.681	8	1.00E+00	7.78E+28	5.681	8	1.00E+00	7.78E+28	8	10	1.2	c.2.1.2
1mxf_1	47	2.225	8	1.00E+00	7.37E+13	2.225	8	1.00E+00	7.37E+13	8	8	0.2	c.2.1.2
1mxf_2	46	3.528	8	1.00E+00	9.94E+19	3.528	8	1.00E+00	9.94E+19	8	9	0.5	c.2.1.2
1mxf_3	46	2.299	7	1.00E+00	1.96E+15	2.299	7	1.00E+00	1.96E+15	7	8	0.8	c.2.1.2
1qyw_2	20	7.767	7	1.00E+00	2.33E+28	7.767	7	1.00E+00	2.33E+28	7	8	0.6	c.2.1.2
1qyx_2	19	7.761	7	1.00E+00	1.94E+28	7.761	7	1.00E+00	1.94E+28	7	8	0.6	c.2.1.2
1qyv_0	33	3.107	7	1.00E+00	1.70E+17	3.107	7	1.00E+00	1.70E+17	7	7	0.4	c.2.1.2
1a27_0	63	4.698	9	1.00E+00	2.00E+25	4.698	9	1.00E+00	2.00E+25	9	12	1.7	c.2.1.2
1fdt_0	50	6.288	8	1.00E+00	9.84E+28	6.288	8	1.00E+00	9.84E+28	8	11	1.4	c.2.1.2
1fdu_0	60	4.704	9	1.00E+00	6.94E+26	4.704	9	1.00E+00	6.94E+26	9	11	1.3	c.2.1.2

Table 17: Results for alcohol dehydrogenase matching against its own SCOP superfamily (but not family) with amino acid property.

Site	N	RMSD	q	Pvalue	Evalue	RMSD	q	Pvalue	Evalue	CG	Mean L	Var L	SCOP
1fdu_1	49	1.928	8	1.00E+00	7.75E+12	1.928	8	1.00E+00	7.75E+12	8	10	1.8	c.2.1.2
1fdu_2	45	1.750	8	1.00E+00	2.77E+11	1.750	8	1.00E+00	2.77E+11	8	8	0.6	c.2.1.2
1fdu_3	47	6.001	8	1.00E+00	4.96E+29	6.001	8	1.00E+00	4.96E+29	8	11	1.4	c.2.1.2
1equ_0	43	6.128	8	1.00E+00	3.63E+28	6.128	8	1.00E+00	3.63E+28	8	11	1.7	c.2.1.2
1equ_1	33	3.793	7	1.00E+00	1.76E+19	3.793	7	1.00E+00	1.76E+19	7	9	0.9	c.2.1.2
1fdv_0	52	6.454	8	1.00E+00	8.32E+27	6.454	8	1.00E+00	8.32E+27	8	11	1.5	c.2.1.2
1fdv_1	34	2.449	8	1.00E+00	1.42E+15	2.449	8	1.00E+00	1.42E+15	8	9	0.5	c.2.1.2
1fdv_3	32	3.147	7	1.00E+00	2.12E+17	3.147	7	1.00E+00	2.12E+17	7	7	0.2	c.2.1.2
1fdv_5	30	2.063	7	1.00E+00	2.93E+13	2.063	7	1.00E+00	2.93E+13	7	8	0.9	c.2.1.2
1fmc_0	47	2.972	8	1.00E+00	1.37E+17	2.972	8	1.00E+00	1.37E+17	8	11	1.1	c.2.1.2
1fmc_1	47	4.727	8	1.00E+00	4.90E+22	0.720	8	5.65E-01	8.32E-01	2	11	1.9	c.2.1.2
1ahi_0	48	1.964	8	1.00E+00	1.72E+13	1.964	8	1.00E+00	1.72E+13	8	10	1.8	c.2.1.2
1ahi_1	46	6.172	8	1.00E+00	4.11E+25	6.172	8	1.00E+00	4.11E+25	8	11	1.5	c.2.1.2
1ahh_0	32	2.190	7	1.00E+00	2.32E+14	2.190	7	1.00E+00	2.32E+14	7	9	0.7	c.2.1.2
1ahh_1	29	2.570	8	1.00E+00	2.33E+16	2.471	10	1.00E+00	2.74E+14	8	10	0.1	c.2.1.2
2hsd_0	35	4.142	7	1.00E+00	9.10E+22	0.628	8	1.02E-02	1.02E-02	0	9	1.7	c.2.1.2
2hsd_1	32	6.251	7	1.00E+00	5.12E+27	0.693	8	1.11E-01	1.17E-01	0	9	1.9	c.2.1.2
2hsd_2	29	7.081	7	1.00E+00	1.72E+26	0.665	7	1.00E+00	1.49E+01	0	8	1.3	c.2.1.2
2hsd_3	32	1.584	8	1.00E+00	1.23E+09	0.692	8	1.06E-01	1.13E-01	4	10	1.9	c.2.1.2
1fk8_0	30	6.540	8	1.00E+00	1.30E+27	6.540	8	1.00E+00	1.30E+27	8	9	0.8	c.2.1.2
1fk8_1	28	6.711	7	1.00E+00	4.11E+25	6.711	7	1.00E+00	4.11E+25	7	9	0.9	c.2.1.2
1nff_0	35	1.928	9	1.00E+00	9.78E+10	1.928	9	1.00E+00	9.78E+10	9	10	0.4	c.2.1.2
1nff_1	35	2.842	8	1.00E+00	1.22E+16	2.842	8	1.00E+00	1.22E+16	8	8	0.3	c.2.1.2
1nfr_0	43	7.414	9	1.00E+00	1.72E+33	7.414	9	1.00E+00	1.72E+33	9	12	1.3	c.2.1.2
1nfr_1	44	7.442	9	1.00E+00	2.12E+33	7.442	9	1.00E+00	2.12E+33	9	12	1.4	c.2.1.2
1nfr_2	43	7.435	9	1.00E+00	1.91E+33	7.435	9	1.00E+00	1.91E+33	9	12	1.3	c.2.1.2
1nfr_3	43	7.442	9	1.00E+00	1.97E+33	7.442	9	1.00E+00	1.97E+33	9	12	1.2	c.2.1.2
1bdb_0	54	1.978	8	1.00E+00	1.59E+12	1.978	8	1.00E+00	1.59E+12	8	9	0.7	c.2.1.2
1b14_0	50	2.060	8	1.00E+00	4.99E+13	2.124	9	1.00E+00	1.70E+13	8	9	0.5	c.2.1.2
1b14_1	35	8.377	9	1.00E+00	2.67E+33	8.377	9	1.00E+00	2.67E+33	9	11	1.1	c.2.1.2
1gee_0	32	2.588	7	1.00E+00	4.00E+15	2.588	7	1.00E+00	4.00E+15	7	8	0.6	c.2.1.2
1gee_1	32	4.021	7	1.00E+00	1.19E+20	4.021	7	1.00E+00	1.19E+20	7	9	0.8	c.2.1.2
1gee_2	31	2.587	7	1.00E+00	3.59E+15	2.587	7	1.00E+00	3.59E+15	7	8	0.6	c.2.1.2
1gee_3	33	2.586	7	1.00E+00	4.31E+15	2.586	7	1.00E+00	4.31E+15	7	8	0.6	c.2.1.2
1gco_0	33	2.596	7	1.00E+00	4.74E+15	2.596	7	1.00E+00	4.74E+15	7	8	0.7	c.2.1.2
1gco_1	33	4.015	7	1.00E+00	1.26E+20	4.015	7	1.00E+00	1.26E+20	7	9	0.7	c.2.1.2
1gco_2	33	4.014	7	1.00E+00	1.25E+20	4.014	7	1.00E+00	1.25E+20	7	9	0.8	c.2.1.2
1gco_3	34	4.015	7	1.00E+00	1.38E+20	4.015	7	1.00E+00	1.38E+20	7	9	0.8	c.2.1.2
1g6k_0	31	2.002	7	1.00E+00	6.50E+12	2.002	7	1.00E+00	6.50E+12	7	7	0.0	c.2.1.2
1g6k_1	31	2.003	7	1.00E+00	6.58E+12	2.003	7	1.00E+00	6.58E+12	7	7	0.0	c.2.1.2
1g6k_2	30	2.001	7	1.00E+00	5.80E+12	2.001	7	1.00E+00	5.80E+12	7	7	0.1	c.2.1.2
1g6k_3	32	2.001	7	1.00E+00	7.08E+12	2.001	7	1.00E+00	7.08E+12	7	7	0.0	c.2.1.2
1rwb_0	34	2.554	7	1.00E+00	3.48E+15	2.554	7	1.00E+00	3.48E+15	7	8	0.7	c.2.1.2
1rwb_1	32	4.028	7	1.00E+00	1.24E+20	4.028	7	1.00E+00	1.24E+20	7	9	0.8	c.2.1.2
1rwb_2	32	2.609	7	1.00E+00	4.88E+15	2.609	7	1.00E+00	4.88E+15	7	8	0.7	c.2.1.2
1rwb_3	33	1.881	7	1.00E+00	4.44E+12	1.881	7	1.00E+00	4.44E+12	7	8	0.8	c.2.1.2
1geg_0	38	7.081	8	1.00E+00	1.16E+32	7.081	8	1.00E+00	1.16E+32	8	11	1.5	c.2.1.2
1geg_1	40	7.029	8	1.00E+00	8.07E+28	7.029	8	1.00E+00	8.07E+28	8	10	1.1	c.2.1.2
1geg_2	38	4.644	8	1.00E+00	1.43E+23	4.644	8	1.00E+00	1.43E+23	8	10	0.8	c.2.1.2
1geg_3	39	7.061	8	1.00E+00	1.14E+32	7.061	8	1.00E+00	1.14E+32	8	11	1.5	c.2.1.2
1geg_4	40	4.689	8	1.00E+00	2.23E+23	4.689	8	1.00E+00	2.23E+23	8	10	0.9	c.2.1.2
1geg_5	39	7.080	8	1.00E+00	1.25E+32	7.080	8	1.00E+00	1.25E+32	8	11	1.5	c.2.1.2
1geg_6	40	7.078	8	1.00E+00	1.34E+32	7.078	8	1.00E+00	1.34E+32	8	11	1.5	c.2.1.2
1geg_7	39	2.217	7	1.00E+00	3.35E+14	2.217	7	1.00E+00	3.35E+14	7	8	0.4	c.2.1.2
1iy8_0	41	5.878	8	1.00E+00	8.41E+27	5.878	8	1.00E+00	8.41E+27	8	11	1.4	c.2.1.2
1iy8_1	40	3.804	8	1.00E+00	7.50E+19	3.705	9	1.00E+00	2.40E+19	8	10	0.8	c.2.1.2
1iy8_2	40	5.897	8	1.00E+00	8.63E+27	0.575	8	1.03E-03	1.03E-03	0	10	2.2	c.2.1.2
1iy8_3	40	5.887	8	1.00E+00	8.18E+27	5.887	8	1.00E+00	8.18E+27	8	11	1.4	c.2.1.2
1iy8_4	41	3.839	8	1.00E+00	1.04E+20	3.738	9	1.00E+00	3.39E+19	8	11	1.0	c.2.1.2
1iy8_5	40	3.846	8	1.00E+00	1.01E+20	3.744	9	1.00E+00	3.30E+19	8	10	0.8	c.2.1.2
1iy8_6	40	3.807	8	1.00E+00	7.66E+19	3.708	9	1.00E+00	2.46E+19	8	10	0.7	c.2.1.2
1iy8_7	40	5.894	8	1.00E+00	8.49E+27	5.894	8	1.00E+00	8.49E+27	8	11	1.3	c.2.1.2
1h5q_0	88	9.281	9	1.00E+00	2.06E+36	9.281	9	1.00E+00	2.06E+36	9	11	1.3	c.2.1.2
1h5q_2	95	2.262	9	1.00E+00	3.97E+15	2.262	9	1.00E+00	3.97E+15	9	10	1.2	c.2.1.2
1h5q_4	89	4.281	10	1.00E+00	5.73E+26	4.281	10	1.00E+00	5.73E+26	10	12	1.5	c.2.1.2

Table 18: Results for alcohol dehydrogenase matching against its own SCOP superfamily (but not family) with amino acid property.

Site	N	RMSD	q	Pvalue	Evalue	RMSD	q	Pvalue	Evalue	CG	Mean L	Var L	SCOP
1h5q_5	91	8.128	10	1.00E+00	5.73E+36	8.128	10	1.00E+00	5.73E+36	10	11	1.0	c.2.1.2
1h5q_6	116	2.245	9	1.00E+00	2.62E+15	2.245	9	1.00E+00	2.62E+15	9	10	0.7	c.2.1.2
1h5q_7	117	1.790	9	1.00E+00	1.89E+12	1.790	9	1.00E+00	1.89E+12	9	9	0.4	c.2.1.2
1h5q_9	91	2.057	9	1.00E+00	1.09E+14	2.057	9	1.00E+00	1.09E+14	9	11	1.1	c.2.1.2
1h5q_10	91	2.276	9	1.00E+00	4.36E+15	2.276	9	1.00E+00	4.36E+15	9	10	1.2	c.2.1.2
1h5q_11	92	5.856	10	1.00E+00	2.70E+32	5.856	10	1.00E+00	2.70E+32	10	13	1.5	c.2.1.2
1h5q_13	90	3.059	10	1.00E+00	1.36E+20	3.059	10	1.00E+00	1.36E+20	10	11	0.8	c.2.1.2
1edo_0	35	2.708	7	1.00E+00	3.75E+16	2.708	7	1.00E+00	3.75E+16	7	8	0.6	c.2.1.2
1q7c_0	19	2.112	7	1.00E+00	2.96E+12	2.112	7	1.00E+00	2.96E+12	7	7	0.0	c.2.1.2
1q7c_1	20	4.626	7	1.00E+00	8.50E+20	4.626	7	1.00E+00	8.50E+20	7	7	0.2	c.2.1.2
1q7b_8	30	2.070	8	1.00E+00	3.80E+12	2.070	8	1.00E+00	3.80E+12	8	8	0.2	c.2.1.2
1q7b_9	30	2.943	7	1.00E+00	5.88E+16	2.943	7	1.00E+00	5.88E+16	7	8	0.7	c.2.1.2
1q7b_10	30	5.305	7	1.00E+00	1.39E+24	5.305	7	1.00E+00	1.39E+24	7	8	0.6	c.2.1.2
1q7b_11	29	4.364	7	1.00E+00	1.61E+22	4.364	7	1.00E+00	1.61E+22	7	7	0.2	c.2.1.2
1o5i_0	29	1.731	7	1.00E+00	6.31E+10	1.731	7	1.00E+00	6.31E+10	7	8	0.4	c.2.1.2
1eno_0	30	2.944	7	1.00E+00	5.01E+17	2.963	8	1.00E+00	2.04E+17	7	8	0.4	c.2.1.2
1d7o_0	32	4.146	8	1.00E+00	8.93E+21	4.146	8	1.00E+00	8.93E+21	8	9	0.5	c.2.1.2
1cwu_0	40	2.068	8	1.00E+00	5.01E+13	2.068	8	1.00E+00	5.01E+13	8	9	0.7	c.2.1.2
1cwu_1	40	3.713	8	1.00E+00	6.82E+20	3.659	9	1.00E+00	2.82E+20	8	9	0.6	c.2.1.2
1nhd_0	34	8.654	8	1.00E+00	1.26E+33	8.654	8	1.00E+00	1.26E+33	8	10	1.1	c.2.1.2
1nhd_1	34	4.204	8	1.00E+00	8.90E+23	4.204	8	1.00E+00	8.90E+23	8	11	1.0	c.2.1.2
1nhg_0	38	8.682	8	1.00E+00	1.97E+33	8.682	8	1.00E+00	1.97E+33	8	10	1.2	c.2.1.2
1nhg_1	39	2.732	7	1.00E+00	8.74E+16	2.678	8	1.00E+00	1.31E+16	7	9	0.9	c.2.1.2
1nhw_0	37	3.917	7	1.00E+00	1.09E+21	3.917	7	1.00E+00	1.09E+21	7	7	0.5	c.2.1.2
1nhw_1	37	2.780	7	1.00E+00	2.75E+17	2.780	7	1.00E+00	2.75E+17	7	9	0.7	c.2.1.2
1nnu_0	38	3.163	7	1.00E+00	3.02E+18	3.163	7	1.00E+00	3.02E+18	7	10	1.0	c.2.1.2
1nnu_1	41	1.533	8	1.00E+00	3.19E+09	1.533	8	1.00E+00	3.19E+09	8	10	1.0	c.2.1.2
1uh5_0	38	2.318	7	1.00E+00	1.36E+15	2.272	8	1.00E+00	1.25E+14	7	9	0.6	c.2.1.2
1uh5_1	38	2.019	8	1.00E+00	4.52E+13	2.019	8	1.00E+00	4.52E+13	8	9	0.6	c.2.1.2
1eny_0	28	1.485	8	1.00E+00	1.36E+09	1.485	8	1.00E+00	1.36E+09	8	8	0.3	c.2.1.2
1p44_0	39	4.134	8	1.00E+00	1.91E+23	4.134	8	1.00E+00	1.91E+23	8	11	1.0	c.2.1.2
1p44_1	43	8.369	8	1.00E+00	6.78E+32	8.369	8	1.00E+00	6.78E+32	8	9	1.0	c.2.1.2
1p44_2	41	3.390	8	1.00E+00	4.24E+20	3.390	8	1.00E+00	4.24E+20	8	10	0.7	c.2.1.2
1p44_3	44	1.887	8	1.00E+00	4.92E+12	1.887	8	1.00E+00	4.92E+12	8	8	0.2	c.2.1.2
1p44_4	29	1.488	7	1.00E+00	1.65E+10	1.488	7	1.00E+00	1.65E+10	7	9	0.9	c.2.1.2
1p44_5	28	1.599	7	1.00E+00	1.16E+11	1.599	7	1.00E+00	1.16E+11	7	9	1.0	c.2.1.2
1enz_0	28	9.328	7	1.00E+00	1.30E+33	9.328	7	1.00E+00	1.30E+33	7	9	0.9	c.2.1.2
1p45_0	43	4.392	8	1.00E+00	9.25E+22	4.392	8	1.00E+00	9.25E+22	8	8	0.4	c.2.1.2
1p45_1	34	1.720	7	1.00E+00	1.71E+12	1.720	7	1.00E+00	1.71E+12	7	9	1.5	c.2.1.2
1bvr_0	39	4.315	8	1.00E+00	4.06E+22	4.315	8	1.00E+00	4.06E+22	8	8	0.4	c.2.1.2
1bvr_1	39	1.894	8	1.00E+00	2.86E+12	1.902	9	1.00E+00	3.06E+11	8	9	0.2	c.2.1.2
1bvr_2	39	2.305	8	1.00E+00	1.40E+15	2.295	9	1.00E+00	1.99E+14	8	9	0.2	c.2.1.2
1bvr_3	39	4.324	8	1.00E+00	4.32E+22	4.324	8	1.00E+00	4.32E+22	8	8	0.4	c.2.1.2
1bvr_4	28	4.667	7	1.00E+00	5.36E+21	4.667	7	1.00E+00	5.36E+21	7	7	0.3	c.2.1.2
1bvr_5	30	5.473	8	1.00E+00	2.03E+25	5.473	8	1.00E+00	2.03E+25	8	9	0.4	c.2.1.2
1qsg_0	37	2.269	8	1.00E+00	4.88E+15	2.269	8	1.00E+00	4.88E+15	8	9	0.8	c.2.1.2
1qsg_1	36	2.240	8	1.00E+00	2.91E+15	2.240	8	1.00E+00	2.91E+15	8	9	0.8	c.2.1.2
1qsg_2	36	6.979	8	1.00E+00	2.05E+28	6.979	8	1.00E+00	2.05E+28	8	8	0.4	c.2.1.2
1qsg_3	36	6.941	8	1.00E+00	1.75E+28	6.941	8	1.00E+00	1.75E+28	8	8	0.4	c.2.1.2
1qsg_4	37	2.245	8	1.00E+00	3.41E+15	2.245	8	1.00E+00	3.41E+15	8	9	0.8	c.2.1.2
1qsg_5	36	2.248	8	1.00E+00	3.28E+15	2.248	8	1.00E+00	3.28E+15	8	9	0.8	c.2.1.2
1qsg_6	36	2.271	8	1.00E+00	4.62E+15	2.271	8	1.00E+00	4.62E+15	8	9	0.8	c.2.1.2
1qsg_7	36	2.228	8	1.00E+00	2.43E+15	2.228	8	1.00E+00	2.43E+15	8	9	0.8	c.2.1.2
1qg6_0	37	3.097	7	1.00E+00	2.59E+19	3.097	7	1.00E+00	2.59E+19	7	9	0.7	c.2.1.2
1qg6_1	37	2.608	8	1.00E+00	7.87E+16	2.608	8	1.00E+00	7.87E+16	8	9	0.6	c.2.1.2
1qg6_2	37	2.608	8	1.00E+00	7.87E+16	2.608	8	1.00E+00	7.87E+16	8	9	0.7	c.2.1.2
1qg6_3	37	2.608	8	1.00E+00	7.87E+16	2.608	8	1.00E+00	7.87E+16	8	9	0.7	c.2.1.2
1dfi_0	28	4.830	7	1.00E+00	7.00E+22	4.830	7	1.00E+00	7.00E+22	7	8	0.5	c.2.1.2
1dfi_1	27	6.025	8	1.00E+00	2.19E+27	6.025	8	1.00E+00	2.19E+27	8	9	0.4	c.2.1.2
1dfi_2	26	6.499	7	1.00E+00	1.98E+26	6.499	7	1.00E+00	1.98E+26	7	8	0.4	c.2.1.2
1dfi_3	29	4.599	8	1.00E+00	9.47E+23	4.599	8	1.00E+00	9.47E+23	8	9	0.2	c.2.1.2
1c14_0	38	1.600	8	1.00E+00	4.29E+10	1.600	8	1.00E+00	4.29E+10	8	8	0.1	c.2.1.2
1c14_1	38	2.669	8	1.00E+00	1.77E+17	2.669	8	1.00E+00	1.77E+17	8	9	0.5	c.2.1.2
1dfh_0	34	3.238	8	1.00E+00	5.48E+18	3.238	8	1.00E+00	5.48E+18	8	9	0.5	c.2.1.2
1dfh_1	33	3.244	8	1.00E+00	5.28E+18	3.244	8	1.00E+00	5.28E+18	8	9	0.5	c.2.1.2

Table 19: Results for alcohol dehydrogenase matching against its own SCOP superfamily (but not family) with amino acid property.

Site	N	RMSD	q	Pvalue	Evalue	RMSD	q	Pvalue	Evalue	CG	Mean L	Var L	SCOP
1dfg_0	34	2.837	8	1.00E+00	1.10E+17	2.837	8	1.00E+00	1.10E+17	8	9	0.5	c.2.1.2
1dfg_1	38	3.308	8	1.00E+00	1.18E+20	3.308	8	1.00E+00	1.18E+20	8	9	0.7	c.2.1.2
1mfp_0	43	7.226	8	1.00E+00	1.16E+31	7.226	8	1.00E+00	1.16E+31	8	10	1.1	c.2.1.2
1mfp_1	42	1.644	8	1.00E+00	8.22E+10	1.644	8	1.00E+00	8.22E+10	8	9	0.4	c.2.1.2
1d8a_0	35	1.902	8	1.00E+00	3.12E+12	1.902	8	1.00E+00	3.12E+12	8	9	0.6	c.2.1.2
1d8a_1	36	2.754	8	1.00E+00	4.03E+17	2.754	8	1.00E+00	4.03E+17	8	9	0.6	c.2.1.2
1lx6_0	32	4.034	7	1.00E+00	4.27E+22	4.034	7	1.00E+00	4.27E+22	7	9	0.7	c.2.1.2
1lx6_1	33	2.560	8	1.00E+00	2.33E+16	2.560	8	1.00E+00	2.33E+16	8	9	0.6	c.2.1.2
1lxc_0	39	1.446	8	1.00E+00	1.56E+09	1.446	8	1.00E+00	1.56E+09	8	8	0.1	c.2.1.2
1lxc_1	38	1.938	8	1.00E+00	7.28E+12	1.938	8	1.00E+00	7.28E+12	8	8	0.3	c.2.1.2
1i30_0	31	4.998	8	1.00E+00	5.24E+26	4.998	8	1.00E+00	5.24E+26	8	10	0.8	c.2.1.2
1i30_1	31	5.013	8	1.00E+00	5.79E+26	5.013	8	1.00E+00	5.79E+26	8	10	0.8	c.2.1.2
1i2z_0	39	2.113	8	1.00E+00	2.77E+13	2.104	9	1.00E+00	3.08E+12	8	10	1.0	c.2.1.2
1i2z_1	38	2.385	8	1.00E+00	3.86E+15	2.385	8	1.00E+00	3.86E+15	8	8	0.4	c.2.1.2
1jw7_0	36	1.745	8	1.00E+00	1.27E+11	1.745	8	1.00E+00	1.27E+11	8	9	0.3	c.2.1.2
1jw7_1	37	5.363	8	1.00E+00	1.68E+28	5.363	8	1.00E+00	1.68E+28	8	9	0.7	c.2.1.2
1jw7_2	37	1.747	8	1.00E+00	1.44E+11	1.747	8	1.00E+00	1.44E+11	8	9	0.3	c.2.1.2
1jw7_3	37	1.742	8	1.00E+00	1.31E+11	1.742	8	1.00E+00	1.31E+11	8	9	0.3	c.2.1.2
1jvf_0	35	6.645	7	1.00E+00	1.59E+27	6.645	7	1.00E+00	1.59E+27	7	8	0.6	c.2.1.2
1jvf_1	36	2.583	8	1.00E+00	1.13E+17	2.583	8	1.00E+00	1.13E+17	8	9	0.6	c.2.1.2
1jvf_2	36	2.582	8	1.00E+00	1.12E+17	2.582	8	1.00E+00	1.12E+17	8	9	0.5	c.2.1.2
1jvf_3	35	2.583	8	1.00E+00	1.04E+17	2.583	8	1.00E+00	1.04E+17	8	9	0.5	c.2.1.2
1ae1_0	33	1.627	7	1.00E+00	2.27E+10	1.740	8	1.00E+00	9.80E+09	7	9	0.6	c.2.1.2
1ae1_1	35	1.469	7	1.00E+00	2.37E+09	1.492	8	1.00E+00	1.65E+08	7	8	0.4	c.2.1.2
2ae2_0	43	1.832	8	1.00E+00	3.33E+11	1.879	9	1.00E+00	6.69E+10	8	9	0.6	c.2.1.2
2ae2_1	44	5.347	7	1.00E+00	1.56E+26	5.347	7	1.00E+00	1.56E+26	7	10	1.3	c.2.1.2
1ipe_0	35	1.944	7	1.00E+00	5.50E+12	1.944	7	1.00E+00	5.50E+12	7	9	0.6	c.2.1.2
1ipe_1	37	4.408	7	1.00E+00	3.39E+22	4.408	7	1.00E+00	3.39E+22	7	9	1.0	c.2.1.2
1ipf_0	45	1.835	8	1.00E+00	5.04E+11	1.804	9	1.00E+00	2.44E+10	8	10	0.3	c.2.1.2
1ipf_1	45	1.828	7	1.00E+00	2.32E+12	1.918	8	1.00E+00	8.00E+11	7	9	0.9	c.2.1.2
1g0o_0	42	1.677	7	1.00E+00	6.92E+11	1.775	9	1.00E+00	2.06E+10	7	9	0.9	c.2.1.2
1g0o_1	42	4.045	7	1.00E+00	8.10E+22	4.045	7	1.00E+00	8.10E+22	7	10	1.9	c.2.1.2
1g0o_2	43	7.478	8	1.00E+00	1.97E+31	7.478	8	1.00E+00	1.97E+31	8	11	1.6	c.2.1.2
1g0o_3	43	1.704	7	1.00E+00	1.18E+12	1.797	9	1.00E+00	3.39E+10	7	10	0.8	c.2.1.2
1g0n_0	43	5.138	7	1.00E+00	2.38E+24	5.138	7	1.00E+00	2.38E+24	7	10	1.2	c.2.1.2
1g0n_1	35	5.095	7	1.00E+00	1.01E+24	5.095	7	1.00E+00	1.01E+24	7	9	1.2	c.2.1.2
1doh_0	43	1.504	7	1.00E+00	3.25E+10	1.504	7	1.00E+00	3.25E+10	7	9	1.2	c.2.1.2
1doh_1	42	1.825	8	1.00E+00	4.84E+11	1.842	9	1.00E+00	5.53E+10	8	9	0.4	c.2.1.2
1ybv_0	113	2.744	10	1.00E+00	2.05E+19	2.730	12	1.00E+00	1.21E+18	10	13	1.0	c.2.1.2
1ybv_1	113	2.744	10	1.00E+00	2.05E+19	2.730	12	1.00E+00	1.21E+18	10	13	0.9	c.2.1.2
1ja9_0	43	4.331	7	1.00E+00	2.55E+22	4.331	7	1.00E+00	2.55E+22	7	11	1.5	c.2.1.2
1hdo_0	109	2.380	10	1.00E+00	2.14E+16	2.380	10	1.00E+00	2.14E+16	10	11	0.9	c.2.1.2
1he2_0	120	2.670	9	1.00E+00	3.42E+18	2.651	10	1.00E+00	6.53E+17	9	12	1.9	c.2.1.2
1he3_0	120	1.787	10	1.00E+00	1.53E+11	1.787	10	1.00E+00	1.53E+11	10	10	0.3	c.2.1.2
1he4_0	115	2.091	9	1.00E+00	9.36E+14	2.091	9	1.00E+00	9.36E+14	9	11	1.3	c.2.1.2
1he5_0	114	2.452	9	1.00E+00	3.05E+17	2.452	9	1.00E+00	3.05E+17	9	10	1.0	c.2.1.2
1e6w_0	111	2.691	11	1.00E+00	1.50E+20	2.671	12	1.00E+00	2.65E+19	11	15	1.6	c.2.1.2
1e6w_1	49	1.176	9	1.00E+00	2.87E+04	1.176	9	1.00E+00	2.87E+04	9	9	0.1	c.2.1.2
1e6w_2	51	6.373	9	1.00E+00	3.62E+31	6.373	9	1.00E+00	3.62E+31	9	11	1.2	c.2.1.2
1e6w_3	111	1.274	9	1.00E+00	4.66E+06	1.257	10	1.00E+00	5.44E+04	6	14	4.2	c.2.1.2
1e3s_0	47	6.431	9	1.00E+00	3.92E+31	6.431	9	1.00E+00	3.92E+31	9	11	1.1	c.2.1.2
1e3s_1	47	6.437	9	1.00E+00	4.06E+31	6.437	9	1.00E+00	4.06E+31	9	11	1.2	c.2.1.2
1e3s_2	46	6.425	9	1.00E+00	3.55E+31	6.425	9	1.00E+00	3.55E+31	9	11	1.0	c.2.1.2
1e3s_3	47	6.428	9	1.00E+00	3.85E+31	6.428	9	1.00E+00	3.85E+31	9	11	1.2	c.2.1.2
1e3w_0	49	1.492	8	1.00E+00	5.15E+09	1.492	8	1.00E+00	5.15E+09	8	8	0.2	c.2.1.2
1e3w_1	49	2.727	9	1.00E+00	2.12E+16	2.727	9	1.00E+00	2.12E+16	9	10	0.6	c.2.1.2
1e3w_2	53	1.547	8	1.00E+00	2.21E+10	1.547	8	1.00E+00	2.21E+10	8	8	0.3	c.2.1.2
1e3w_3	55	5.746	9	1.00E+00	7.78E+29	5.746	9	1.00E+00	7.78E+29	9	12	1.1	c.2.1.2
1n5d_1	33	1.565	7	1.00E+00	4.37E+10	1.628	9	1.00E+00	4.42E+08	7	9	0.1	c.2.1.2
1sb8_0	55	3.053	8	1.00E+00	1.65E+19	3.053	8	1.00E+00	1.65E+19	8	9	1.0	c.2.1.2
1sb9_0	53	1.743	7	1.00E+00	4.21E+12	1.743	7	1.00E+00	4.21E+12	7	9	0.8	c.2.1.2
1vl8_2	37	1.090	8	1.00E+00	6.47E+04	0.680	8	1.35E-01	1.46E-01	7	10	1.6	c.2.1.2
1vl8_3	37	1.093	8	1.00E+00	6.98E+04	0.910	6	1.00E+00	9.59E+06	5	11	2.1	c.2.1.2
1sny_1	40	6.102	8	1.00E+00	1.92E+28	6.102	8	1.00E+00	1.92E+28	8	12	1.5	c.2.1.2
1xhl_0	43	2.208	7	1.00E+00	1.96E+15	2.208	7	1.00E+00	1.96E+15	7	9	0.9	c.2.1.2

Table 20: Results for alcohol dehydrogenase matching against its own SCOP superfamily (but not family) with amino acid property.

Site	N	RMSD	q	Pvalue	Evalue	RMSD	q	Pvalue	Evalue	CG	Mean L	Var L	SCOP
1xhl_1	43	2.206	7	1.00E+00	1.91E+15	2.206	7	1.00E+00	1.91E+15	7	9	1.0	c.2.1.2
1gad_0	30	1.138	9	1.00E+00	1.58E+03	0.824	11	1.50E-06	1.50E-06	8	12	1.3	c.2.1.3
1gad_1	29	1.344	10	1.00E+00	1.51E+04	0.928	12	1.19E-06	1.19E-06	7	12	1.2	c.2.1.3
1dc6_0	29	1.329	10	1.00E+00	9.72E+03	0.996	13	3.03E-07	3.03E-07	7	12	0.8	c.2.1.3
1dc6_1	28	1.333	10	1.00E+00	9.81E+03	0.804	11	4.50E-07	4.50E-07	7	11	0.8	c.2.1.3
1gae_0	28	0.924	9	7.20E-01	1.27E+00	0.677	10	2.84E-07	2.84E-07	8	11	1.5	c.2.1.3
1gae_1	29	1.133	10	1.00E+00	3.14E+01	0.676	10	3.01E-07	3.01E-07	8	12	1.6	c.2.1.3
1gd1_0	69	2.300	9	1.00E+00	1.25E+15	1.001	13	4.27E-06	4.27E-06	0	16	1.9	c.2.1.3
1gd1_1	70	1.727	9	1.00E+00	2.02E+10	0.988	12	2.40E-04	2.40E-04	0	16	2.2	c.2.1.3
1gd1_2	69	1.373	10	1.00E+00	5.03E+05	1.034	13	2.04E-05	2.04E-05	7	16	2.1	c.2.1.3
1gd1_3	70	1.385	10	1.00E+00	7.41E+05	1.017	13	9.59E-06	9.59E-06	7	16	2.0	c.2.1.3
1nqo_0	34	1.355	10	1.00E+00	3.42E+04	0.996	12	2.72E-05	2.72E-05	7	12	1.2	c.2.1.3
1nqo_1	34	1.193	9	1.00E+00	8.98E+03	1.000	12	3.26E-05	3.26E-05	7	12	1.1	c.2.1.3
1nqo_2	33	1.353	10	1.00E+00	2.94E+04	0.936	11	1.96E-04	1.96E-04	7	12	1.5	c.2.1.3
1nqo_3	34	1.357	10	1.00E+00	3.62E+04	0.993	12	2.37E-05	2.37E-05	7	12	1.1	c.2.1.3
1nq5_0	32	1.378	10	1.00E+00	5.50E+04	0.983	12	1.24E-05	1.24E-05	7	12	1.1	c.2.1.3
1nq5_1	32	1.196	9	1.00E+00	8.12E+03	1.002	12	2.96E-05	2.96E-05	7	12	1.3	c.2.1.3
1nq5_2	31	1.376	10	1.00E+00	4.70E+04	1.059	12	3.29E-04	3.29E-04	7	12	1.3	c.2.1.3
1nq5_3	32	1.367	10	1.00E+00	4.01E+04	0.812	10	1.53E-04	1.53E-04	7	11	6.8	c.2.1.3
1npt_0	35	1.188	9	1.00E+00	8.50E+03	0.993	12	2.60E-05	2.60E-05	7	13	1.3	c.2.1.3
1npt_1	34	1.186	9	1.00E+00	7.33E+03	0.896	11	3.69E-05	3.69E-05	7	12	1.2	c.2.1.3
1npt_2	34	1.189	9	1.00E+00	8.00E+03	1.018	12	7.32E-05	7.32E-05	7	12	1.0	c.2.1.3
1npt_3	34	1.560	9	1.00E+00	7.09E+07	1.014	12	6.13E-05	6.13E-05	7	12	1.1	c.2.1.3
1nqa_0	36	1.198	9	1.00E+00	1.24E+04	0.996	12	3.25E-05	3.25E-05	7	13	1.4	c.2.1.3
1nqa_1	37	1.539	9	1.00E+00	5.77E+07	0.993	12	3.08E-05	3.08E-05	7	12	1.2	c.2.1.3
1nqa_2	36	1.556	9	1.00E+00	7.74E+07	0.973	12	1.13E-05	1.13E-05	7	12	1.0	c.2.1.3
1nqa_3	36	1.574	9	1.00E+00	1.15E+08	1.004	12	4.67E-05	4.67E-05	7	12	1.0	c.2.1.3
2dbv_0	33	1.374	9	1.00E+00	1.43E+06	1.084	12	1.53E-03	1.53E-03	6	12	1.4	c.2.1.3
2dbv_1	35	1.402	9	1.00E+00	3.44E+06	1.023	11	1.13E-02	1.14E-02	6	12	1.2	c.2.1.3
2dbv_2	33	1.258	8	1.00E+00	2.09E+06	0.988	11	2.32E-03	2.32E-03	6	11	1.0	c.2.1.3
2dbv_3	34	1.385	9	1.00E+00	2.07E+06	1.032	12	1.82E-04	1.82E-04	6	12	1.2	c.2.1.3
1dbv_0	31	1.398	9	1.00E+00	2.14E+06	0.988	11	1.91E-03	1.91E-03	6	12	1.2	c.2.1.3
1dbv_1	33	1.418	9	1.00E+00	4.24E+06	1.002	11	4.09E-03	4.10E-03	6	12	1.2	c.2.1.3
1dbv_2	39	1.243	8	1.00E+00	2.46E+06	0.960	11	1.79E-03	1.79E-03	6	13	1.9	c.2.1.3
1dbv_3	29	1.403	9	1.00E+00	1.97E+06	0.998	11	2.33E-03	2.34E-03	6	12	1.1	c.2.1.3
4dbv_0	32	1.152	8	1.00E+00	7.99E+04	0.926	11	8.75E-05	8.75E-05	6	12	1.2	c.2.1.3
4dbv_1	33	1.159	8	1.00E+00	1.05E+05	0.927	11	1.01E-04	1.01E-04	6	12	1.7	c.2.1.3
4dbv_2	32	1.163	8	1.00E+00	1.06E+05	0.937	11	1.41E-04	1.41E-04	6	12	1.1	c.2.1.3
4dbv_3	30	1.186	8	1.00E+00	1.54E+05	0.868	11	5.27E-06	5.27E-06	6	12	1.2	c.2.1.3
3dbv_0	32	1.381	9	1.00E+00	8.78E+05	0.951	11	2.56E-04	2.56E-04	6	12	1.6	c.2.1.3
3dbv_1	33	1.156	8	1.00E+00	9.73E+04	0.884	10	2.58E-03	2.58E-03	6	11	1.8	c.2.1.3
3dbv_2	30	1.298	9	1.00E+00	8.47E+04	0.938	11	1.20E-04	1.20E-04	6	11	1.3	c.2.1.3
3dbv_3	30	1.295	9	1.00E+00	7.82E+04	0.914	11	4.23E-05	4.23E-05	6	12	1.3	c.2.1.3
1cer_0	32	1.268	9	1.00E+00	6.10E+04	0.937	11	2.49E-04	2.49E-04	7	11	0.8	c.2.1.3
1cer_1	30	1.204	9	1.00E+00	8.36E+03	0.992	11	2.03E-03	2.03E-03	7	11	0.9	c.2.1.3
1cer_2	30	1.217	9	1.00E+00	1.21E+04	1.001	11	2.92E-03	2.93E-03	7	11	1.0	c.2.1.3
1cer_3	29	1.188	9	1.00E+00	4.75E+03	0.799	9	2.41E-02	2.44E-02	7	10	1.1	c.2.1.3
1vc2_0	29	1.177	8	1.00E+00	1.11E+05	1.011	10	2.98E-01	3.54E-01	6	11	2.0	c.2.1.3
1hdg_0	40	1.960	9	1.00E+00	4.05E+11	0.980	10	3.43E-01	4.19E-01	7	13	4.2	c.2.1.3
1hdg_1	41	1.958	9	1.00E+00	4.22E+11	1.006	10	5.80E-01	8.67E-01	7	13	2.7	c.2.1.3
1cf2_4	31	4.263	7	1.00E+00	6.01E+21	4.263	7	1.00E+00	6.01E+21	7	9	1.0	c.2.1.3
1cf2_5	31	4.223	7	1.00E+00	4.68E+21	4.223	7	1.00E+00	4.68E+21	7	9	1.0	c.2.1.3
1cf2_6	31	4.231	7	1.00E+00	4.92E+21	4.231	7	1.00E+00	4.92E+21	7	9	1.1	c.2.1.3
1cf2_7	31	4.248	7	1.00E+00	5.47E+21	4.248	7	1.00E+00	5.47E+21	7	9	1.0	c.2.1.3
1gga_0	32	2.410	9	1.00E+00	3.35E+14	0.755	8	7.11E-01	1.24E+00	7	11	2.0	c.2.1.3
1gga_1	32	2.010	9	1.00E+00	6.40E+11	0.772	9	1.52E-02	1.53E-02	7	11	2.1	c.2.1.3
1gga_2	32	2.001	9	1.00E+00	5.48E+11	1.011	9	1.00E+00	4.31E+01	7	11	2.1	c.2.1.3
1gga_3	32	2.009	9	1.00E+00	6.29E+11	0.777	9	1.85E-02	1.87E-02	7	11	2.2	c.2.1.3
1gga_4	31	2.005	9	1.00E+00	5.32E+11	0.766	9	1.09E-02	1.09E-02	7	11	2.2	c.2.1.3
1gga_5	31	2.005	9	1.00E+00	5.32E+11	1.026	10	6.20E-01	9.69E-01	7	11	2.1	c.2.1.3
1ml3_0	39	3.109	9	1.00E+00	2.88E+19	0.972	10	1.97E-01	2.20E-01	6	13	1.9	c.2.1.3
1ml3_1	34	1.311	10	1.00E+00	1.23E+04	1.113	11	3.37E-01	4.11E-01	8	12	1.5	c.2.1.3
1ml3_2	36	7.026	8	1.00E+00	3.29E+31	7.026	8	1.00E+00	3.29E+31	8	10	0.7	c.2.1.3
1qxs_0	40	2.667	9	1.00E+00	3.49E+17	2.555	12	1.00E+00	7.37E+14	9	13	0.7	c.2.1.3
1qxs_1	33	1.480	8	1.00E+00	3.09E+08	1.025	8	1.00E+00	4.73E+03	5	13	4.4	c.2.1.3

Table 21: Results for alcohol dehydrogenase matching against its own SCOP superfamily (but not family) with amino acid property.

Site	N	RMSD	q	Pvalue	Evalue	RMSD	q	Pvalue	Evalue	CG	Mean L	Var L	SCOP
1qxs_2	32	1.307	10	1.00E+00	9.02E+03	1.112	11	2.80E-01	3.29E-01	8	12	1.3	c.2.1.3
1qxs_3	31	2.271	8	1.00E+00	2.48E+14	0.930	8	1.00E+00	3.52E+02	6	12	2.5	c.2.1.3
1gyp_0	42	1.787	11	1.00E+00	8.71E+08	0.977	11	4.10E-03	4.10E-03	8	13	1.7	c.2.1.3
1gyp_1	44	1.762	11	1.00E+00	5.38E+08	0.941	10	1.25E-01	1.33E-01	8	13	1.8	c.2.1.3
1gyp_2	42	1.791	11	1.00E+00	9.62E+08	1.134	12	3.23E-02	3.28E-02	8	13	1.3	c.2.1.3
1gyp_3	42	1.787	11	1.00E+00	8.71E+08	0.981	11	4.83E-03	4.84E-03	8	13	1.6	c.2.1.3
1a7k_0	33	1.245	10	1.00E+00	1.46E+03	1.015	11	9.05E-03	9.10E-03	8	12	1.2	c.2.1.3
1a7k_1	34	1.259	10	1.00E+00	2.49E+03	0.895	10	1.02E-02	1.02E-02	8	13	1.7	c.2.1.3
1a7k_2	31	1.290	10	1.00E+00	4.88E+03	1.069	11	5.89E-02	6.07E-02	8	12	1.3	c.2.1.3
1a7k_3	39	1.745	11	1.00E+00	2.41E+08	0.864	10	4.47E-03	4.48E-03	8	13	1.5	c.2.1.3
1gpd_0	73	1.556	11	1.00E+00	1.33E+07	0.968	11	1.52E-02	1.53E-02	7	13	1.5	c.2.1.3
1gpd_1	78	1.402	10	1.00E+00	2.20E+06	1.184	9	1.00E+00	7.91E+04	6	15	3.4	c.2.1.3
1dss_0	45	2.011	10	1.00E+00	4.66E+11	0.835	10	1.57E-03	1.57E-03	7	12	1.6	c.2.1.3
1dss_1	46	2.009	10	1.00E+00	4.79E+11	0.814	10	9.00E-04	9.00E-04	7	13	1.6	c.2.1.3
1szj_0	44	1.429	10	1.00E+00	6.15E+05	0.828	10	2.15E-03	2.15E-03	7	14	2.2	c.2.1.3
1szj_1	33	1.333	9	1.00E+00	3.76E+05	0.743	9	3.97E-03	3.97E-03	7	10	1.3	c.2.1.3
3gpd_0	91	1.671	10	1.00E+00	4.68E+09	0.895	11	2.13E-03	2.13E-03	7	14	2.5	c.2.1.3
3gpd_1	35	1.480	9	1.00E+00	1.66E+07	0.945	10	5.64E-02	5.81E-02	7	11	1.2	c.2.1.3
1j0x_0	38	2.285	8	1.00E+00	1.11E+14	0.819	9	1.13E-01	1.20E-01	6	11	1.4	c.2.1.3
1j0x_1	39	2.261	9	1.00E+00	6.83E+13	0.928	10	4.17E-02	4.26E-02	6	12	2.1	c.2.1.3
1rm4_0	38	1.933	8	1.00E+00	8.01E+11	1.022	9	1.00E+00	1.34E+02	5	13	2.0	c.2.1.3
1rm4_1	30	1.266	8	1.00E+00	1.88E+06	0.552	5	1.00E+00	1.76E+05	5	10	3.0	c.2.1.3
1rm4_2	32	1.756	7	1.00E+00	2.78E+11	1.705	8	1.00E+00	8.62E+09	7	8	0.3	c.2.1.3
1rm5_0	27	1.187	7	1.00E+00	6.22E+06	0.755	7	1.00E+00	5.07E+02	5	10	2.0	c.2.1.3
1rm5_1	31	1.773	7	1.00E+00	2.32E+12	1.773	7	1.00E+00	2.32E+12	7	8	0.5	c.2.1.3
1rm5_2	27	4.841	7	1.00E+00	2.21E+20	4.841	7	1.00E+00	2.21E+20	7	8	0.5	c.2.1.3
1rm3_0	33	1.158	8	1.00E+00	1.02E+05	1.050	10	8.68E-01	2.02E+00	6	11	1.2	c.2.1.3
1rm3_1	36	1.164	8	1.00E+00	1.56E+05	1.013	9	1.00E+00	5.02E+01	6	11	1.4	c.2.1.3
1rm3_2	39	1.291	8	1.00E+00	7.53E+06	1.038	10	9.77E-01	3.79E+00	6	12	1.7	c.2.1.3
1nbo_0	40	1.944	9	1.00E+00	2.32E+11	1.099	10	1.00E+00	1.56E+01	6	14	2.3	c.2.1.3
1nbo_1	40	1.254	9	1.00E+00	8.28E+04	0.974	10	2.42E-01	2.76E-01	6	13	2.9	c.2.1.3
1nbo_2	41	1.287	9	1.00E+00	2.19E+05	0.924	9	9.99E-01	7.19E+00	6	13	2.9	c.2.1.3
1jn0_0	35	1.998	8	1.00E+00	9.24E+11	0.957	9	1.00E+00	8.12E+00	6	11	1.3	c.2.1.3
1jn0_1	45	1.267	9	1.00E+00	1.70E+05	1.057	10	9.98E-01	6.11E+00	6	12	2.4	c.2.1.3
1jn0_2	35	2.023	7	1.00E+00	5.25E+12	0.889	8	1.00E+00	1.95E+02	5	10	1.5	c.2.1.3
1gl3_0	120	1.769	10	1.00E+00	7.27E+11	1.769	10	1.00E+00	7.27E+11	10	10	0.4	c.2.1.3
1gl3_1	102	1.519	10	1.00E+00	1.04E+09	1.549	12	1.00E+00	6.44E+06	10	13	1.1	c.2.1.3
1mb4_0	37	2.888	8	1.00E+00	2.25E+16	2.888	8	1.00E+00	2.25E+16	8	10	1.0	c.2.1.3
1mb4_1	36	6.300	7	1.00E+00	1.22E+28	6.300	7	1.00E+00	1.22E+28	7	11	2.2	c.2.1.3
1pqu_0	40	5.053	8	1.00E+00	1.52E+24	5.053	8	1.00E+00	1.52E+24	8	10	1.2	c.2.1.3
1pqu_1	40	2.652	8	1.00E+00	1.42E+16	2.652	8	1.00E+00	1.42E+16	8	9	0.5	c.2.1.3
1pqu_3	42	4.359	8	1.00E+00	9.67E+23	4.359	8	1.00E+00	9.67E+23	8	11	1.1	c.2.1.3
1nvm_16	36	2.977	7	1.00E+00	1.56E+18	2.996	8	1.00E+00	6.58E+17	7	10	0.8	c.2.1.3
1nvm_17	36	2.347	7	1.00E+00	2.11E+15	2.347	7	1.00E+00	2.11E+15	7	9	1.1	c.2.1.3
1nvm_18	36	2.998	7	1.00E+00	1.89E+18	3.023	8	1.00E+00	8.58E+17	7	10	0.9	c.2.1.3
1ebf_2	26	0.903	8	1.00E+00	5.45E+01	0.747	9	1.21E-03	1.21E-03	7	10	1.1	c.2.1.3
1e5q_0	120	1.576	10	1.00E+00	1.88E+09	1.576	10	1.00E+00	1.88E+09	10	11	0.7	c.2.1.3
1e5q_1	120	1.616	9	1.00E+00	6.66E+10	1.616	10	1.00E+00	3.87E+09	9	10	0.4	c.2.1.3
1e5q_2	116	6.442	8	1.00E+00	4.82E+30	6.442	8	1.00E+00	4.82E+30	8	12	1.8	c.2.1.3
1e5q_3	120	7.284	9	1.00E+00	1.11E+35	7.284	9	1.00E+00	1.11E+35	9	11	1.5	c.2.1.3
1e5q_4	120	6.318	8	1.00E+00	5.56E+28	6.318	8	1.00E+00	5.56E+28	8	10	1.4	c.2.1.3
1e5q_5	119	4.940	8	1.00E+00	5.03E+25	4.940	8	1.00E+00	5.03E+25	8	11	1.6	c.2.1.3
1e5q_6	116	3.713	8	1.00E+00	5.70E+21	3.713	8	1.00E+00	5.70E+21	8	10	1.6	c.2.1.3
1e5q_7	120	1.611	9	1.00E+00	5.95E+10	1.611	10	1.00E+00	3.43E+09	9	10	0.4	c.2.1.3
1f06_0	40	1.078	9	1.00E+00	7.00E+02	1.005	10	5.40E-01	7.76E-01	8	10	0.8	c.2.1.3
1f06_1	28	0.691	8	6.98E-02	7.24E-02	0.691	8	6.98E-02	7.24E-02	8	10	0.5	c.2.1.3
3dap_0	73	2.094	10	1.00E+00	1.33E+13	2.101	11	1.00E+00	1.90E+12	10	12	0.9	c.2.1.3
3dap_1	113	1.504	10	1.00E+00	6.53E+07	1.504	10	1.00E+00	6.53E+07	10	10	0.4	c.2.1.3
1dap_0	73	3.332	9	1.00E+00	4.33E+21	3.271	10	1.00E+00	1.01E+21	9	12	1.3	c.2.1.3
1dap_1	112	1.885	9	1.00E+00	3.86E+13	1.885	9	1.00E+00	3.86E+13	9	12	1.6	c.2.1.3
1dih_0	61	1.657	9	1.00E+00	3.32E+09	1.657	9	1.00E+00	3.32E+09	9	11	1.2	c.2.1.3
1arz_0	33	5.797	7	1.00E+00	5.97E+23	5.797	7	1.00E+00	5.97E+23	7	9	1.1	c.2.1.3
1arz_1	32	1.417	8	1.00E+00	4.79E+07	1.417	8	1.00E+00	4.79E+07	8	9	1.1	c.2.1.3
1arz_2	35	1.855	8	1.00E+00	2.40E+11	1.876	9	1.00E+00	3.01E+10	8	10	0.6	c.2.1.3
1p9l_0	36	1.052	9	1.00E+00	3.02E+02	0.547	7	2.95E-01	3.50E-01	5	10	1.9	c.2.1.3

Table 22: Results for alcohol dehydrogenase matching against its own SCOP superfamily (but not family) with amino acid property.

Site	N	RMSD	q	Pvalue	Evalue	RMSD	q	Pvalue	Evalue	CG	Mean L	Var L	SCOP
1p9l_1	37	1.061	9	1.00E+00	4.34E+02	0.549	7	3.39E-01	4.14E-01	5	11	2.7	c.2.1.3
1c3v_0	37	1.588	10	1.00E+00	2.85E+07	0.511	7	7.84E-02	8.16E-02	7	11	1.8	c.2.1.3
1c3v_1	37	1.589	10	1.00E+00	2.91E+07	0.511	7	7.84E-02	8.16E-02	7	10	1.8	c.2.1.3
1vm6_0	35	1.838	9	1.00E+00	2.04E+10	1.103	9	1.00E+00	1.07E+03	8	10	1.4	c.2.1.3
1vm6_1	35	1.402	8	1.00E+00	7.40E+07	1.118	9	1.00E+00	1.62E+03	6	10	1.5	c.2.1.3
1vm6_2	36	1.302	8	1.00E+00	5.96E+06	1.153	9	1.00E+00	4.52E+03	7	9	1.1	c.2.1.3
1vm6_3	30	2.784	8	1.00E+00	2.42E+16	2.799	9	1.00E+00	8.25E+15	8	10	0.6	c.2.1.3
1gr0_0	44	1.262	9	1.00E+00	1.03E+05	0.259	5	9.98E-01	6.02E+00	4	10	2.3	c.2.1.3
1p1h_0	36	7.919	7	1.00E+00	2.45E+29	7.919	7	1.00E+00	2.45E+29	7	9	1.2	c.2.1.3
1p1h_1	38	1.831	7	1.00E+00	2.54E+12	1.835	8	1.00E+00	2.27E+11	7	9	0.7	c.2.1.3
1p1h_2	37	1.510	7	1.00E+00	2.32E+10	1.510	7	1.00E+00	2.32E+10	7	9	0.8	c.2.1.3
1p1h_3	38	1.748	7	1.00E+00	1.67E+12	1.748	7	1.00E+00	1.67E+12	7	9	1.1	c.2.1.3
1jkf_0	38	1.421	8	1.00E+00	9.89E+07	1.421	8	1.00E+00	9.89E+07	8	8	0.1	c.2.1.3
1jkf_1	35	4.127	7	1.00E+00	8.60E+21	4.127	7	1.00E+00	8.60E+21	7	9	0.8	c.2.1.3
1p1i_0	37	5.794	8	1.00E+00	2.99E+27	5.794	8	1.00E+00	2.99E+27	8	10	1.1	c.2.1.3
1p1i_1	37	9.233	7	1.00E+00	5.17E+30	9.233	7	1.00E+00	5.17E+30	7	9	1.1	c.2.1.3
1la2_32	39	1.736	7	1.00E+00	6.66E+11	1.841	9	1.00E+00	2.31E+10	7	10	1.0	c.2.1.3
1la2_33	37	7.991	7	1.00E+00	3.39E+29	7.991	7	1.00E+00	3.39E+29	7	9	0.9	c.2.1.3
1la2_34	37	1.466	7	1.00E+00	3.56E+09	1.466	7	1.00E+00	3.56E+09	7	7	0.2	c.2.1.3
1la2_35	39	7.976	7	1.00E+00	3.79E+29	7.976	7	1.00E+00	3.79E+29	7	9	0.9	c.2.1.3
1u1i_0	46	1.260	9	1.00E+00	8.39E+04	1.260	9	1.00E+00	8.39E+04	9	10	0.6	c.2.1.3
1u1i_1	47	1.785	9	1.00E+00	6.14E+10	1.785	9	1.00E+00	6.14E+10	9	9	0.4	c.2.1.3
1u1i_4	39	1.289	8	1.00E+00	4.02E+06	0.788	8	9.89E-01	4.51E+00	6	8	0.7	c.2.1.3
1u1i_5	36	7.240	8	1.00E+00	6.64E+31	7.240	8	1.00E+00	6.64E+31	8	10	0.8	c.2.1.3
1vko_0	52	2.528	8	1.00E+00	7.29E+16	2.528	8	1.00E+00	7.29E+16	8	9	1.5	c.2.1.3
1j5p_0	34	1.027	8	1.00E+00	1.86E+03	1.109	11	1.65E-01	1.81E-01	8	11	1.1	c.2.1.3
1h2h_0	70	2.882	8	1.00E+00	6.03E+19	2.907	9	1.00E+00	2.62E+19	8	10	0.9	c.2.1.3
1q0h_1	47	1.447	8	1.00E+00	2.52E+08	0.763	7	1.00E+00	2.62E+03	6	9	2.0	c.2.1.3
1q0q_0	43	4.436	8	1.00E+00	1.35E+24	4.436	8	1.00E+00	1.35E+24	8	11	0.9	c.2.1.3
1q0q_1	43	1.406	8	1.00E+00	2.21E+08	1.427	9	1.00E+00	4.62E+06	6	12	2.2	c.2.1.3
1jvs_2	33	2.155	7	1.00E+00	3.99E+13	2.155	7	1.00E+00	3.99E+13	7	9	0.8	c.2.1.3
1jvs_6	33	3.418	8	1.00E+00	1.38E+21	3.355	9	1.00E+00	3.65E+20	8	11	0.9	c.2.1.3
1q0l_0	43	3.510	8	1.00E+00	1.23E+20	1.351	9	1.00E+00	9.72E+05	5	12	2.0	c.2.1.3
1r0l_0	16	1.571	7	1.00E+00	3.89E+09	1.571	7	1.00E+00	3.89E+09	7	7	0.3	c.2.1.3
1r0l_1	16	1.955	7	1.00E+00	5.47E+11	1.955	7	1.00E+00	5.47E+11	7	7	0.2	c.2.1.3
1r0l_2	16	1.577	7	1.00E+00	4.34E+09	1.577	7	1.00E+00	4.34E+09	7	7	0.3	c.2.1.3
1r0l_3	17	1.914	7	1.00E+00	3.78E+11	1.914	7	1.00E+00	3.78E+11	7	7	0.0	c.2.1.3
1h6d_0	120	6.912	10	1.00E+00	7.76E+35	6.912	10	1.00E+00	7.76E+35	10	12	1.3	c.2.1.3
1h6d_1	116	1.267	9	1.00E+00	3.95E+06	1.267	9	1.00E+00	3.95E+06	9	10	0.7	c.2.1.3
1h6d_2	119	4.765	9	1.00E+00	1.97E+29	4.765	9	1.00E+00	1.97E+29	9	13	2.0	c.2.1.3
1h6d_3	120	6.871	10	1.00E+00	6.06E+35	6.871	10	1.00E+00	6.06E+35	10	12	1.3	c.2.1.3
1h6d_4	120	1.625	9	1.00E+00	4.18E+10	1.625	9	1.00E+00	4.18E+10	9	9	0.3	c.2.1.3
1h6d_5	119	6.933	10	1.00E+00	8.58E+35	6.933	10	1.00E+00	8.58E+35	10	12	1.2	c.2.1.3
1h6d_6	119	6.916	10	1.00E+00	7.75E+35	6.916	10	1.00E+00	7.75E+35	10	12	1.2	c.2.1.3
1h6d_7	118	6.919	10	1.00E+00	7.69E+35	6.919	10	1.00E+00	7.69E+35	10	12	1.3	c.2.1.3
1h6d_8	120	6.880	10	1.00E+00	6.40E+35	6.880	10	1.00E+00	6.40E+35	10	12	1.2	c.2.1.3
1h6d_9	120	1.589	9	1.00E+00	1.93E+10	1.589	9	1.00E+00	1.93E+10	9	9	0.3	c.2.1.3
1h6d_10	120	6.887	10	1.00E+00	6.68E+35	6.887	10	1.00E+00	6.68E+35	10	12	1.2	c.2.1.3
1h6d_11	120	4.751	9	1.00E+00	1.80E+29	4.751	9	1.00E+00	1.80E+29	9	12	1.9	c.2.1.3
1h6c_0	120	4.431	9	1.00E+00	8.48E+26	4.431	9	1.00E+00	8.48E+26	9	12	1.4	c.2.1.3
1h6c_1	115	1.699	8	1.00E+00	8.88E+11	1.699	8	1.00E+00	8.88E+11	8	8	0.2	c.2.1.3
1h6a_0	120	1.520	9	1.00E+00	7.03E+09	1.520	9	1.00E+00	7.03E+09	9	10	0.5	c.2.1.3
1h6a_1	108	1.599	9	1.00E+00	1.74E+10	1.599	9	1.00E+00	1.74E+10	9	9	0.3	c.2.1.3
1h6b_0	120	6.859	9	1.00E+00	3.27E+35	6.859	9	1.00E+00	3.27E+35	9	13	2.2	c.2.1.3
1h6b_1	120	1.580	9	1.00E+00	1.59E+10	1.580	9	1.00E+00	1.59E+10	9	9	0.3	c.2.1.3
1ofg_0	41	3.756	7	1.00E+00	8.28E+19	3.756	7	1.00E+00	8.28E+19	7	8	0.6	c.2.1.3
1ofg_1	41	5.256	7	1.00E+00	1.79E+26	5.256	7	1.00E+00	1.79E+26	7	9	1.1	c.2.1.3
1ofg_2	41	5.250	7	1.00E+00	1.73E+26	5.250	7	1.00E+00	1.73E+26	7	9	1.1	c.2.1.3
1ofg_3	41	1.957	7	1.00E+00	2.26E+13	2.019	8	1.00E+00	6.17E+12	7	8	0.6	c.2.1.3
1ofg_4	31	6.540	7	1.00E+00	2.20E+28	0.590	7	4.26E-01	5.54E-01	0	8	1.0	c.2.1.3
1ofg_5	31	6.540	7	1.00E+00	2.20E+28	0.590	7	4.26E-01	5.54E-01	0	8	1.0	c.2.1.3
1evj_0	25	2.905	6	1.00E+00	9.07E+15	2.905	6	1.00E+00	9.07E+15	6	6	0.1	c.2.1.3
1evj_1	25	2.162	6	1.00E+00	9.30E+13	2.162	6	1.00E+00	9.30E+13	6	6	0.2	c.2.1.3
1evj_2	25	4.700	6	1.00E+00	2.33E+22	4.700	6	1.00E+00	2.33E+22	6	8	0.4	c.2.1.3
1evj_3	25	2.160	6	1.00E+00	9.12E+13	2.160	6	1.00E+00	9.12E+13	6	6	0.1	c.2.1.3

Table 23: Results for alcohol dehydrogenase matching against its own SCOP superfamily (but not family) with amino acid property.

Site	N	RMSD	q	Pvalue	Evalue	RMSD	q	Pvalue	Evalue	CG	Mean L	Var L	SCOP
2dpg_0	17	1.904	6	1.00E+00	1.72E+12	1.925	7	1.00E+00	2.12E+11	6	7	0.2	c.2.1.3
1e7y_2	35	5.190	7	1.00E+00	7.65E+25	5.190	7	1.00E+00	7.65E+25	7	10	1.1	c.2.1.3
1h9a_1	41	4.609	8	1.00E+00	3.47E+25	4.609	8	1.00E+00	3.47E+25	8	11	0.6	c.2.1.3
1h94_0	44	2.383	7	1.00E+00	3.25E+16	2.383	7	1.00E+00	3.25E+16	7	8	0.9	c.2.1.3
1qki_5	24	2.630	6	1.00E+00	2.17E+16	2.630	6	1.00E+00	2.17E+16	6	6	0.0	c.2.1.3
1qki_7	24	2.630	6	1.00E+00	2.17E+16	2.630	6	1.00E+00	2.17E+16	6	6	0.1	c.2.1.3
1qki_9	24	2.624	6	1.00E+00	2.05E+16	2.624	6	1.00E+00	2.05E+16	6	6	0.1	c.2.1.3
1qki_11	25	2.639	6	1.00E+00	2.67E+16	2.639	6	1.00E+00	2.67E+16	6	6	0.0	c.2.1.3
1qki_13	23	2.631	6	1.00E+00	1.91E+16	2.631	6	1.00E+00	1.91E+16	6	6	0.0	c.2.1.3
1qki_15	24	2.624	6	1.00E+00	2.05E+16	2.624	6	1.00E+00	2.05E+16	6	6	0.0	c.2.1.3
1qki_17	24	6.161	6	1.00E+00	7.20E+24	6.161	6	1.00E+00	7.20E+24	6	6	0.1	c.2.1.3
1qki_19	25	2.621	6	1.00E+00	2.27E+16	2.621	6	1.00E+00	2.27E+16	6	6	0.0	c.2.1.3
2nad_0	40	1.349	8	1.00E+00	3.00E+07	1.381	9	1.00E+00	2.31E+06	8	10	0.5	c.2.1.4
2nad_1	40	1.321	8	1.00E+00	1.55E+07	1.355	9	1.00E+00	1.20E+06	8	10	0.5	c.2.1.4
1qp8_10	34	0.778	9	1.08E-02	1.09E-02	0.840	11	1.12E-05	1.12E-05	8	12	1.2	c.2.1.4
1mx3_0	37	1.330	10	1.00E+00	3.71E+04	1.355	12	1.00E+00	9.30E+01	10	12	0.4	c.2.1.4
1hku_0	115	1.458	11	1.00E+00	2.98E+06	0.593	7	1.00E+00	7.52E+01	6	12	3.5	c.2.1.4
1hl3_0	80	1.180	10	1.00E+00	2.66E+03	1.226	12	1.00E+00	8.03E+00	10	13	1.6	c.2.1.4
1dxy_0	73	1.532	9	1.00E+00	1.20E+09	1.532	9	1.00E+00	1.20E+09	9	9	0.3	c.2.1.4
1psd_0	76	1.934	9	1.00E+00	5.56E+13	2.024	11	1.00E+00	4.05E+12	9	13	2.1	c.2.1.4
1j49_0	37	1.648	8	1.00E+00	6.44E+09	0.570	7	6.20E-01	9.67E-01	6	9	1.2	c.2.1.4
1j49_1	29	6.379	8	1.00E+00	3.81E+28	6.379	8	1.00E+00	3.81E+28	8	9	0.7	c.2.1.4
2dld_0	29	2.463	7	1.00E+00	2.21E+15	2.558	9	1.00E+00	3.02E+14	7	9	0.5	c.2.1.4
2dld_1	27	1.640	7	1.00E+00	3.50E+10	1.640	7	1.00E+00	3.50E+10	7	8	0.6	c.2.1.4
1pjc_0	32	0.803	11	4.02E-07	4.02E-07	0.660	11	1.33E-10	1.33E-10	10	12	0.6	c.2.1.4
1f8g_11	38	1.062	7	1.00E+00	1.14E+06	1.062	7	1.00E+00	1.14E+06	7	7	0.2	c.2.1.4
1f8g_32	29	1.047	7	1.00E+00	3.49E+05	1.047	7	1.00E+00	3.49E+05	7	7	0.1	c.2.1.4
1f8g_33	26	1.714	7	1.00E+00	2.35E+11	1.714	7	1.00E+00	2.35E+11	7	8	0.3	c.2.1.4
1f8g_34	25	2.854	7	1.00E+00	1.09E+17	2.854	7	1.00E+00	1.09E+17	7	8	0.9	c.2.1.4
1nm5_0	30	5.270	8	1.00E+00	2.10E+27	0.562	7	1.07E-01	1.13E-01	0	7	0.3	c.2.1.4
1nm5_1	16	1.351	7	1.00E+00	2.85E+07	0.451	7	6.91E-05	6.91E-05	6	7	0.2	c.2.1.4
1nm5_2	34	4.013	8	1.00E+00	3.66E+22	4.013	8	1.00E+00	3.66E+22	8	10	1.1	c.2.1.4
1ptj_1	33	1.678	8	1.00E+00	2.87E+10	1.686	9	1.00E+00	2.19E+09	8	9	0.2	c.2.1.4
1hzz_0	30	1.709	7	1.00E+00	5.47E+11	1.709	7	1.00E+00	5.47E+11	7	8	0.4	c.2.1.4
1hzz_1	32	1.679	8	1.00E+00	2.66E+10	1.695	9	1.00E+00	2.40E+09	8	9	0.2	c.2.1.4
1d4f_0	52	1.489	9	1.00E+00	4.23E+07	1.095	12	1.10E-02	1.11E-02	7	13	1.4	c.2.1.4
1d4f_1	51	1.457	9	1.00E+00	1.97E+07	1.026	11	5.09E-02	5.23E-02	7	13	1.7	c.2.1.4
1d4f_2	52	2.422	9	1.00E+00	4.35E+15	2.422	9	1.00E+00	4.35E+15	9	11	1.5	c.2.1.4
1d4f_3	50	1.424	9	1.00E+00	8.80E+06	1.000	11	1.82E-02	1.84E-02	7	13	1.5	c.2.1.4
1b3r_0	38	1.007	8	1.00E+00	3.40E+03	0.944	10	1.04E-01	1.09E-01	7	10	0.7	c.2.1.4
1b3r_1	39	1.305	9	1.00E+00	1.36E+05	0.948	10	1.28E-01	1.37E-01	7	11	0.7	c.2.1.4
1b3r_2	39	1.294	9	1.00E+00	1.04E+05	0.937	10	8.83E-02	9.25E-02	7	11	0.7	c.2.1.4
1b3r_3	38	0.999	8	1.00E+00	2.73E+03	0.933	10	7.13E-02	7.40E-02	7	10	0.8	c.2.1.4
1k0u_0	54	1.975	9	1.00E+00	1.32E+12	1.062	12	3.30E-03	3.31E-03	4	12	1.2	c.2.1.4
1k0u_1	53	2.039	10	1.00E+00	1.33E+12	1.059	12	2.76E-03	2.76E-03	5	12	1.1	c.2.1.4
1k0u_2	53	1.981	9	1.00E+00	1.39E+12	1.078	12	5.95E-03	5.97E-03	4	13	1.2	c.2.1.4
1k0u_3	53	2.047	10	1.00E+00	1.55E+12	0.942	11	2.22E-03	2.22E-03	5	12	1.3	c.2.1.4
1k0u_4	52	1.405	9	1.00E+00	4.86E+06	1.100	12	1.34E-02	1.35E-02	7	12	1.4	c.2.1.4
1k0u_5	55	6.188	9	1.00E+00	1.76E+29	1.151	12	1.08E-01	1.14E-01	3	13	1.4	c.2.1.4
1k0u_6	56	1.581	9	1.00E+00	4.91E+08	0.953	11	4.09E-03	4.10E-03	6	12	1.3	c.2.1.4
1k0u_7	55	1.266	9	1.00E+00	1.95E+05	1.105	12	1.93E-02	1.95E-02	8	12	1.1	c.2.1.4
1d4g_0	52	3.349	9	1.00E+00	7.17E+19	1.090	12	9.05E-03	9.09E-03	4	12	1.1	c.2.1.4
1d4g_1	54	1.418	10	1.00E+00	4.33E+05	1.092	12	1.10E-02	1.10E-02	7	12	1.4	c.2.1.4
1d4g_2	52	6.630	8	1.00E+00	1.04E+28	1.162	12	1.35E-01	1.45E-01	1	13	1.5	c.2.1.4
1d4g_3	54	1.464	10	1.00E+00	1.43E+06	1.074	12	5.37E-03	5.38E-03	7	13	1.2	c.2.1.4
1d4g_4	52	1.416	9	1.00E+00	6.26E+06	1.065	11	2.07E-01	2.32E-01	7	12	1.5	c.2.1.4
1d4g_5	55	1.504	9	1.00E+00	5.20E+07	1.094	11	5.38E-01	7.71E-01	5	12	1.5	c.2.1.4
1d4g_6	54	2.617	9	1.00E+00	4.30E+15	1.072	11	2.84E-01	3.35E-01	7	13	1.8	c.2.1.4
1d4g_7	53	1.453	9	1.00E+00	2.02E+07	1.055	11	1.57E-01	1.71E-01	7	12	1.9	c.2.1.4
1ky4_0	35	0.972	8	1.00E+00	9.95E+02	0.922	10	3.79E-02	3.86E-02	7	11	0.7	c.2.1.4
1ky4_1	36	0.970	8	1.00E+00	1.03E+03	0.927	10	4.92E-02	5.05E-02	7	11	0.8	c.2.1.4
1ky4_2	35	0.969	8	1.00E+00	9.13E+02	0.930	10	5.02E-02	5.16E-02	7	11	0.8	c.2.1.4
1ky4_3	35	0.966	8	1.00E+00	8.39E+02	0.924	10	4.07E-02	4.15E-02	7	11	0.7	c.2.1.4
1pjs_4	21	3.377	7	1.00E+00	4.74E+18	3.377	7	1.00E+00	4.74E+18	7	9	0.8	c.2.1.11
1pjs_6	23	3.118	7	1.00E+00	7.54E+17	3.118	7	1.00E+00	7.54E+17	7	8	0.2	c.2.1.11

Table 24: Results for alcohol dehydrogenase matching against its own SCOP superfamily (but not family) with amino acid property.

Site	N	RMSD	q	Pvalue	Evalue	RMSD	q	Pvalue	Evalue	CG	Mean L	Var L	SCOP
1kyq_18	20	1.578	6	1.00E+00	2.66E+10	0.689	7	1.00E+00	1.83E+01	3	7	0.7	c.2.1.11
1kyq_19	22	2.045	6	1.00E+00	2.55E+12	2.131	7	1.00E+00	7.52E+11	6	7	0.2	c.2.1.11
1kyq_20	23	2.048	6	1.00E+00	3.02E+12	2.122	7	1.00E+00	7.85E+11	6	7	0.1	c.2.1.11
4mdh_0	35	0.833	9	6.88E-02	7.13E-02	0.544	9	1.35E-07	1.35E-07	8	10	0.6	c.2.1.5
4mdh_1	31	0.952	10	5.86E-02	6.04E-02	0.697	9	2.92E-04	2.92E-04	9	11	1.2	c.2.1.5
5mdh_0	35	0.860	8	1.00E+00	3.24E+01	0.904	10	1.66E-02	1.67E-02	7	10	0.7	c.2.1.5
5mdh_1	37	0.808	10	2.26E-04	2.26E-04	0.576	9	1.03E-06	1.03E-06	9	10	1.0	c.2.1.5
1civ_0	32	5.193	8	1.00E+00	1.38E+26	0.788	9	1.74E-02	1.76E-02	0	10	1.2	c.2.1.5
1emd_0	67	1.580	11	1.00E+00	1.46E+07	1.034	10	1.00E+00	1.36E+01	7	14	3.2	c.2.1.5
1ib6_0	34	1.562	8	1.00E+00	4.33E+09	1.037	10	9.17E-01	2.48E+00	0	11	1.8	c.2.1.5
1ib6_1	36	1.623	8	1.00E+00	1.73E+10	1.626	9	1.00E+00	1.09E+09	8	10	0.9	c.2.1.5
1ib6_2	35	1.927	8	1.00E+00	4.18E+11	1.099	10	1.00E+00	2.12E+01	5	11	2.1	c.2.1.5
1ie3_0	30	1.583	8	1.00E+00	4.47E+09	0.896	9	6.92E-01	1.18E+00	0	10	1.6	c.2.1.5
1ie3_1	30	1.577	8	1.00E+00	3.97E+09	0.917	9	9.08E-01	2.38E+00	0	10	1.3	c.2.1.5
1ie3_2	31	1.599	8	1.00E+00	6.80E+09	1.597	9	1.00E+00	3.67E+08	8	10	0.8	c.2.1.5
1ie3_3	32	3.074	8	1.00E+00	1.06E+17	1.085	10	1.00E+00	1.02E+01	5	10	1.4	c.2.1.5
1bmd_0	27	2.520	8	1.00E+00	2.62E+14	0.824	9	4.27E-02	4.36E-02	0	11	1.3	c.2.1.5
1bmd_1	26	1.629	8	1.00E+00	2.04E+09	0.703	9	4.62E-04	4.62E-04	6	11	0.8	c.2.1.5
1o6z_1	120	4.290	9	1.00E+00	1.09E+25	4.290	9	1.00E+00	1.09E+25	9	13	1.9	c.2.1.5
1o6z_3	62	2.915	9	1.00E+00	3.79E+17	2.915	9	1.00E+00	3.79E+17	9	11	1.2	c.2.1.5
1hlp_0	31	1.689	8	1.00E+00	1.03E+10	0.962	9	9.98E-01	6.45E+00	4	11	1.7	c.2.1.5
1hlp_1	30	1.693	8	1.00E+00	9.97E+09	0.956	9	9.92E-01	4.81E+00	4	11	2.0	c.2.1.5
1gt2_0	106	7.716	10	1.00E+00	8.64E+35	7.716	10	1.00E+00	8.64E+35	10	13	2.0	c.2.1.5
1gt2_3	102	3.901	10	1.00E+00	3.02E+22	3.901	10	1.00E+00	3.02E+22	10	11	0.8	c.2.1.5
1b8u_0	38	1.238	9	1.00E+00	1.18E+05	0.475	8	6.00E-06	6.00E-06	6	10	1.7	c.2.1.5
1uxj_0	120	2.511	10	1.00E+00	1.07E+18	2.511	10	1.00E+00	1.07E+18	10	12	1.2	c.2.1.5
1uxk_0	120	1.852	10	1.00E+00	1.45E+12	1.852	10	1.00E+00	1.45E+12	10	12	1.0	c.2.1.5
1uxk_1	84	1.172	9	1.00E+00	1.78E+05	0.866	10	6.47E-02	6.68E-02	7	12	1.0	c.2.1.5
1ur5_0	120	2.643	10	1.00E+00	6.97E+18	2.644	11	1.00E+00	1.74E+18	10	12	1.2	c.2.1.5
1uxg_0	119	5.733	9	1.00E+00	1.82E+32	5.733	9	1.00E+00	1.82E+32	9	14	2.4	c.2.1.5
1uxg_1	40	1.485	9	1.00E+00	4.92E+07	0.977	12	4.38E-05	4.38E-05	6	13	1.7	c.2.1.5
1guy_0	117	3.054	10	1.00E+00	2.57E+21	3.054	10	1.00E+00	2.57E+21	10	13	1.5	c.2.1.5
1guy_2	108	3.787	8	1.00E+00	2.84E+24	3.787	8	1.00E+00	2.84E+24	8	11	1.3	c.2.1.5
1uxh_0	117	0.832	10	1.51E-02	1.52E-02	1.187	14	5.90E-03	5.91E-03	10	15	2.5	c.2.1.5
1uxh_1	40	1.477	9	1.00E+00	4.08E+07	1.009	13	2.73E-06	2.73E-06	7	13	1.7	c.2.1.5
1uxi_0	118	3.009	9	1.00E+00	9.12E+21	3.009	9	1.00E+00	9.12E+21	9	11	1.0	c.2.1.5
1uxi_1	41	1.487	9	1.00E+00	5.56E+07	1.014	13	3.78E-06	3.78E-06	7	13	1.8	c.2.1.5
1gv0_0	99	2.508	10	1.00E+00	4.38E+17	2.454	11	1.00E+00	3.52E+16	10	13	1.6	c.2.1.5
1gv0_1	95	0.851	11	7.02E-05	7.02E-05	0.879	13	2.12E-08	2.12E-08	10	14	1.7	c.2.1.5
1guz_0	33	0.906	10	9.15E-03	9.20E-03	0.732	11	8.77E-09	8.77E-09	8	13	1.9	c.2.1.5
1guz_1	32	0.945	10	4.31E-02	4.40E-02	0.783	11	1.38E-07	1.38E-07	8	13	1.7	c.2.1.5
1guz_2	33	1.216	9	1.00E+00	3.64E+04	0.724	11	5.51E-09	5.51E-09	7	13	1.4	c.2.1.5
1guz_3	32	0.906	10	8.33E-03	8.36E-03	0.803	12	1.44E-09	1.44E-09	9	13	1.3	c.2.1.5
1ojs_0	97	2.062	10	1.00E+00	5.26E+13	2.025	12	1.00E+00	3.27E+11	10	12	0.4	c.2.1.5
1hyh_0	37	1.060	9	1.00E+00	3.44E+02	0.800	11	4.08E-07	4.08E-07	8	12	1.2	c.2.1.5
1hyh_1	38	1.048	9	1.00E+00	2.53E+02	0.652	10	8.00E-08	8.00E-08	7	11	2.0	c.2.1.5
1hyh_2	31	0.991	9	1.00E+00	1.96E+01	0.724	12	3.36E-11	3.36E-11	7	12	1.9	c.2.1.5
1hyh_3	31	0.994	9	1.00E+00	2.17E+01	0.752	12	1.87E-10	1.87E-10	7	13	0.7	c.2.1.5
9ldt_0	39	0.867	10	1.57E-03	1.57E-03	0.661	10	1.91E-07	1.91E-07	8	11	1.8	c.2.1.5
9ldt_1	38	0.991	10	1.96E-01	2.19E-01	0.728	10	6.53E-06	6.53E-06	7	12	2.3	c.2.1.5
9ldb_0	38	2.267	10	1.00E+00	2.52E+13	0.665	10	2.21E-07	2.21E-07	6	12	2.4	c.2.1.5
9ldb_1	38	1.004	10	3.00E-01	3.56E-01	0.703	10	1.77E-06	1.77E-06	7	12	1.9	c.2.1.5
1t2f_0	36	0.739	9	2.35E-03	2.35E-03	0.527	9	5.26E-08	5.26E-08	8	11	2.0	c.2.1.5
1t2f_1	36	2.952	8	1.00E+00	1.17E+18	2.933	10	1.00E+00	1.07E+17	8	11	1.1	c.2.1.5
1t2f_2	35	0.819	8	1.00E+00	8.87E+00	0.542	7	2.29E-01	2.61E-01	5	10	1.7	c.2.1.5
1t2f_3	38	0.748	9	4.10E-03	4.11E-03	0.774	10	6.47E-05	6.47E-05	8	12	1.8	c.2.1.5
1ldm_0	36	0.761	9	6.07E-03	6.09E-03	0.700	10	1.27E-06	1.27E-06	8	12	2.3	c.2.1.5
1t2d_0	40	2.267	8	1.00E+00	3.91E+14	2.226	9	1.00E+00	3.27E+13	8	10	0.8	c.2.1.5
1t24_0	41	2.273	8	1.00E+00	4.59E+14	2.231	9	1.00E+00	3.81E+13	8	10	0.9	c.2.1.5
1ldg_0	41	2.904	9	1.00E+00	2.78E+18	2.894	11	1.00E+00	2.52E+17	9	11	0.9	c.2.1.5
1oc4_0	95	5.100	9	1.00E+00	1.02E+30	5.100	9	1.00E+00	1.02E+30	9	12	1.6	c.2.1.5
1oc4_1	40	4.122	9	1.00E+00	9.76E+22	4.122	9	1.00E+00	9.76E+22	9	10	1.0	c.2.1.5
1pzh_0	39	2.072	8	1.00E+00	2.79E+13	2.075	9	1.00E+00	3.53E+12	8	10	0.9	c.2.1.5
1pzh_1	40	6.862	8	1.00E+00	4.80E+31	6.862	8	1.00E+00	4.80E+31	8	11	1.1	c.2.1.5
1pzh_2	40	2.196	8	1.00E+00	2.53E+14	0.958	9	1.00E+00	1.12E+01	0	10	2.0	c.2.1.5

Table 25: Results for alcohol dehydrogenase matching against its own SCOP superfamily (but not family) with amino acid property.

Site	N	RMSD	q	Pvalue	Evalue	RMSD	q	Pvalue	Evalue	CG	Mean L	Var L	SCOP
1pzh_3	40	2.591	8	1.00E+00	6.17E+16	2.591	8	1.00E+00	6.17E+16	8	9	1.0	c.2.1.5
1ldn_0	38	2.437	8	1.00E+00	5.77E+15	2.437	8	1.00E+00	5.77E+15	8	10	0.6	c.2.1.5
1ldn_1	37	2.067	8	1.00E+00	2.20E+13	2.067	8	1.00E+00	2.20E+13	8	10	1.0	c.2.1.5
1ldn_2	40	2.215	9	1.00E+00	1.32E+14	0.990	12	5.69E-05	5.69E-05	0	12	1.5	c.2.1.5
1ldn_3	39	2.188	9	1.00E+00	7.79E+13	2.232	10	1.00E+00	2.41E+13	9	11	1.0	c.2.1.5
1ldn_4	37	2.378	8	1.00E+00	2.45E+15	2.378	9	1.00E+00	4.42E+14	8	10	0.6	c.2.1.5
1ldn_5	38	2.457	8	1.00E+00	7.47E+15	2.469	9	1.00E+00	1.75E+15	8	10	0.6	c.2.1.5
1ldn_6	37	2.319	8	1.00E+00	6.29E+14	2.332	9	1.00E+00	1.28E+14	8	11	1.0	c.2.1.5
1ldn_7	36	3.541	8	1.00E+00	5.28E+19	0.374	6	4.45E-01	5.89E-01	1	10	3.5	c.2.1.5
2ldb_0	115	10.431	11	1.00E+00	3.12E+44	10.431	11	1.00E+00	3.12E+44	11	14	1.8	c.2.1.5
1ez4_0	31	1.107	9	1.00E+00	5.57E+02	0.838	10	3.82E-04	3.82E-04	8	10	0.5	c.2.1.5
1ez4_1	32	1.088	8	1.00E+00	1.66E+04	0.469	7	7.45E-03	7.48E-03	6	9	1.5	c.2.1.5
1ez4_2	31	1.127	9	1.00E+00	9.96E+02	0.848	10	5.95E-04	5.95E-04	8	10	0.6	c.2.1.5
1ez4_3	30	3.634	7	1.00E+00	1.06E+19	3.634	7	1.00E+00	1.06E+19	7	9	0.9	c.2.1.5
1lld_0	77	2.072	9	1.00E+00	2.01E+13	1.094	12	5.95E-02	6.14E-02	6	13	2.7	c.2.1.5
1lth_0	84	3.672	9	1.00E+00	7.38E+22	3.672	9	1.00E+00	7.38E+22	9	13	2.4	c.2.1.5
1lth_2	119	1.787	9	1.00E+00	4.50E+12	1.744	10	1.00E+00	1.28E+11	9	11	0.4	c.2.1.5
1a5z_0	31	0.758	9	4.44E-03	4.45E-03	0.999	12	5.38E-05	5.38E-05	9	12	0.8	c.2.1.5
1hye_0	30	3.952	7	1.00E+00	3.10E+20	3.952	7	1.00E+00	3.10E+20	7	9	0.9	c.2.1.5
1hyg_0	32	2.047	7	1.00E+00	1.65E+13	2.047	7	1.00E+00	1.65E+13	7	10	1.4	c.2.1.5
1hyg_1	32	3.342	7	1.00E+00	9.45E+17	3.342	7	1.00E+00	9.45E+17	7	9	1.2	c.2.1.5
1obb_0	120	1.782	9	1.00E+00	1.34E+12	1.782	9	1.00E+00	1.34E+12	9	9	0.3	c.2.1.5
1obb_1	105	2.832	8	1.00E+00	1.15E+20	2.786	9	1.00E+00	1.90E+19	8	12	1.1	c.2.1.5
1vjt_0	40	1.601	8	1.00E+00	1.16E+10	1.601	8	1.00E+00	1.16E+10	8	8	0.0	c.2.1.5
1u8x_1	47	2.757	7	1.00E+00	2.64E+17	2.757	7	1.00E+00	2.64E+17	7	7	0.2	c.2.1.5
1up6_0	102	1.780	9	1.00E+00	2.52E+11	1.780	9	1.00E+00	2.52E+11	9	10	1.0	c.2.1.5
1up6_1	112	2.680	9	1.00E+00	1.25E+19	2.713	11	1.00E+00	1.40E+18	9	11	0.9	c.2.1.5
1up6_9	104	1.876	9	1.00E+00	9.85E+12	1.876	9	1.00E+00	9.85E+12	9	9	0.3	c.2.1.5
1up6_11	41	1.670	7	1.00E+00	1.57E+11	1.670	7	1.00E+00	1.57E+11	7	9	1.3	c.2.1.5
1up6_13	39	4.892	7	1.00E+00	8.44E+24	4.892	7	1.00E+00	8.44E+24	7	10	1.2	c.2.1.5
1up6_14	33	4.634	7	1.00E+00	9.00E+22	4.634	7	1.00E+00	9.00E+22	7	8	0.7	c.2.1.5
1yve_0	41	1.409	8	1.00E+00	1.05E+08	1.182	8	1.00E+00	6.08E+05	6	10	2.4	c.2.1.6
1yve_2	42	2.276	8	1.00E+00	2.92E+14	2.276	8	1.00E+00	2.92E+14	8	9	0.7	c.2.1.6
1yve_3	41	4.127	8	1.00E+00	9.18E+22	4.127	8	1.00E+00	9.18E+22	8	10	0.8	c.2.1.6
1yve_4	42	1.227	8	1.00E+00	1.57E+06	0.888	6	1.00E+00	5.62E+06	5	10	2.4	c.2.1.6
1pgo_0	24	0.699	8	2.16E-02	2.19E-02	0.681	9	1.60E-05	1.60E-05	8	9	0.8	c.2.1.6
1j3v_0	40	1.253	10	1.00E+00	1.24E+03	0.569	8	7.72E-04	7.72E-04	6	10	1.7	c.2.1.6
1j3v_2	40	1.250	10	1.00E+00	1.14E+03	0.584	8	1.58E-03	1.58E-03	6	10	2.0	c.2.1.6
1j3v_4	40	1.109	9	1.00E+00	7.36E+02	0.590	8	2.09E-03	2.10E-03	6	11	1.6	c.2.1.6
1lj8_8	27	2.372	7	1.00E+00	2.76E+14	2.394	8	1.00E+00	6.36E+13	7	8	0.3	c.2.1.6
1m2w_4	35	2.452	7	1.00E+00	1.18E+16	2.406	8	1.00E+00	1.28E+15	7	9	0.5	c.2.1.6
1m2w_5	34	1.766	7	1.00E+00	3.90E+11	1.766	7	1.00E+00	3.90E+11	7	7	0.2	c.2.1.6
1f0y_0	50	4.096	9	1.00E+00	1.42E+24	0.466	9	1.50E-09	1.50E-09	5	11	1.1	c.2.1.6
1f0y_1	50	1.814	9	1.00E+00	1.76E+11	0.497	9	1.22E-08	1.22E-08	5	11	1.4	c.2.1.6
3had_0	37	3.343	8	1.00E+00	6.48E+19	3.343	8	1.00E+00	6.48E+19	8	10	0.8	c.2.1.6
3had_1	28	3.289	8	1.00E+00	1.63E+19	0.482	8	3.49E-06	3.49E-06	4	8	0.4	c.2.1.6
1m76_0	49	1.784	10	1.00E+00	2.17E+10	0.384	7	3.01E-04	3.01E-04	4	11	2.9	c.2.1.6
1m76_1	50	1.372	10	1.00E+00	2.38E+05	0.494	9	1.00E-08	1.00E-08	7	11	2.1	c.2.1.6
2hdh_0	32	4.407	7	1.00E+00	9.23E+21	0.438	7	1.59E-03	1.59E-03	0	8	2.5	c.2.1.6
2hdh_1	33	1.571	8	1.00E+00	2.59E+09	1.571	8	1.00E+00	2.59E+09	8	8	0.0	c.2.1.6
1il0_0	50	1.553	10	1.00E+00	4.16E+07	0.502	9	1.69E-08	1.69E-08	7	11	1.2	c.2.1.6
1il0_1	49	1.598	10	1.00E+00	1.20E+08	0.556	9	4.38E-07	4.38E-07	7	11	1.6	c.2.1.6
1m75_0	51	1.606	10	1.00E+00	1.66E+08	0.508	9	2.63E-08	2.63E-08	7	12	2.9	c.2.1.6
1m75_1	51	2.147	10	1.00E+00	5.27E+13	0.509	9	2.81E-08	2.81E-08	6	11	1.7	c.2.1.6
1lsj_0	25	3.957	7	1.00E+00	4.89E+19	3.957	7	1.00E+00	4.89E+19	7	9	0.6	c.2.1.6
1lsj_1	27	1.965	7	1.00E+00	5.78E+12	0.415	8	5.04E-08	5.04E-08	0	8	0.7	c.2.1.6
1lso_0	28	1.106	8	1.00E+00	2.06E+04	0.504	8	1.19E-05	1.19E-05	7	8	0.5	c.2.1.6
1lso_1	27	1.047	8	1.00E+00	3.64E+03	0.446	8	3.67E-07	3.67E-07	7	8	0.4	c.2.1.6
3hdh_0	28	3.323	8	1.00E+00	2.26E+19	0.452	8	5.94E-07	5.94E-07	4	8	0.5	c.2.1.6
3hdh_1	25	3.240	8	1.00E+00	7.14E+18	0.511	8	1.23E-05	1.23E-05	4	8	0.7	c.2.1.6
3hdh_2	26	1.714	8	1.00E+00	1.49E+10	0.480	8	2.47E-06	2.47E-06	6	8	0.4	c.2.1.6
1i36_0	28	6.657	7	1.00E+00	8.36E+26	0.432	6	6.72E-01	1.11E+00	0	8	1.2	c.2.1.6
1i36_1	27	1.177	7	1.00E+00	1.57E+07	1.007	7	1.00E+00	2.52E+05	5	9	1.8	c.2.1.6
1dli_0	39	1.413	9	1.00E+00	2.38E+06	0.587	9	1.27E-06	1.27E-06	8	9	0.8	c.2.1.6
1dli_1	52	2.388	11	1.00E+00	1.51E+14	0.587	9	3.06E-06	3.06E-06	9	10	1.8	c.2.1.6

Table 26: Results for alcohol dehydrogenase matching against its own SCOP superfamily (but not family) with amino acid property.

Site	N	RMSD	q	Pvalue	Evalue	RMSD	q	Pvalue	Evalue	CG	Mean L	Var L	SCOP
1mv8_3	30	1.308	10	1.00E+00	4.50E+03	0.475	11	0.00E+00	5.50E-17	9	11	0.3	c.2.1.6
1mv8_4	30	1.287	10	1.00E+00	2.45E+03	0.459	11	0.00E+00	1.29E-17	9	11	0.2	c.2.1.6
1mv8_5	30	1.301	10	1.00E+00	3.68E+03	0.487	11	1.11E-16	1.58E-16	9	11	0.3	c.2.1.6
1mv8_6	30	1.243	10	1.00E+00	6.68E+02	0.462	11	0.00E+00	1.70E-17	8	11	0.3	c.2.1.6
1muu_31	30	1.283	10	1.00E+00	2.18E+03	0.472	11	0.00E+00	4.21E-17	9	11	0.3	c.2.1.6
1muu_32	30	1.287	10	1.00E+00	2.45E+03	0.511	11	1.22E-15	1.21E-15	9	11	0.2	c.2.1.6
1muu_33	30	1.245	10	1.00E+00	7.09E+02	0.485	11	1.11E-16	1.33E-16	9	11	0.3	c.2.1.6
1muu_34	30	1.316	10	1.00E+00	5.65E+03	0.525	11	3.77E-15	3.81E-15	9	11	0.3	c.2.1.6
1jay_0	39	2.692	8	1.00E+00	1.53E+16	2.692	8	1.00E+00	1.53E+16	8	8	0.4	c.2.1.6
1jay_1	37	5.735	9	1.00E+00	8.96E+27	5.735	9	1.00E+00	8.96E+27	9	10	0.9	c.2.1.6
1wdk_3	28	2.716	8	1.00E+00	1.31E+15	0.854	10	7.55E-04	7.55E-04	3	10	3.1	c.2.1.6
1wdk_6	28	2.719	8	1.00E+00	1.35E+15	2.719	8	1.00E+00	1.35E+15	8	8	0.0	c.2.1.6
1wdm_4	28	1.079	8	1.00E+00	9.92E+03	1.001	11	3.04E-03	3.05E-03	7	11	1.0	c.2.1.6
1wdm_5	28	1.075	8	1.00E+00	8.89E+03	1.010	11	4.44E-03	4.45E-03	7	11	0.8	c.2.1.6
1wdl_2	25	1.111	8	1.00E+00	1.65E+04	0.910	10	5.70E-03	5.71E-03	7	10	0.5	c.2.1.6
1wdl_6	29	1.130	8	1.00E+00	4.33E+04	0.909	10	8.67E-03	8.71E-03	7	11	0.8	c.2.1.6
1hwz_0	42	1.179	9	1.00E+00	1.97E+04	0.370	5	1.00E+00	1.34E+04	5	13	3.5	c.2.1.7
1hwz_1	42	1.179	9	1.00E+00	1.97E+04	0.370	5	1.00E+00	1.34E+04	5	13	3.4	c.2.1.7
1hwz_2	42	1.179	9	1.00E+00	1.97E+04	0.370	5	1.00E+00	1.34E+04	5	13	3.6	c.2.1.7
1hwz_3	42	1.179	9	1.00E+00	1.97E+04	0.370	5	1.00E+00	1.34E+04	5	13	5.0	c.2.1.7
1hwz_4	42	1.179	9	1.00E+00	1.97E+04	0.370	5	1.00E+00	1.34E+04	5	13	3.3	c.2.1.7
1hwz_5	42	1.178	9	1.00E+00	1.92E+04	0.370	5	1.00E+00	1.34E+04	5	13	3.7	c.2.1.7
1hwy_7	38	1.197	9	1.00E+00	1.34E+04	0.763	6	1.00E+00	6.55E+05	5	13	3.4	c.2.1.7
1hwy_8	38	1.198	9	1.00E+00	1.38E+04	1.138	7	1.00E+00	9.16E+06	5	13	3.1	c.2.1.7
1hwy_9	38	1.198	9	1.00E+00	1.38E+04	1.096	6	1.00E+00	1.93E+08	5	13	3.2	c.2.1.7
1hwy_10	38	1.198	9	1.00E+00	1.38E+04	1.198	9	1.00E+00	1.38E+04	9	13	3.2	c.2.1.7
1hwy_11	38	1.197	9	1.00E+00	1.34E+04	1.096	6	1.00E+00	1.93E+08	5	13	3.5	c.2.1.7
1hwy_12	38	1.198	9	1.00E+00	1.38E+04	1.482	9	1.00E+00	7.03E+06	6	13	3.1	c.2.1.7
1hwy_13	33	2.935	7	1.00E+00	3.95E+18	2.935	7	1.00E+00	3.95E+18	7	7	0.4	c.2.1.7
1hwy_14	33	2.277	7	1.00E+00	1.56E+15	2.277	7	1.00E+00	1.56E+15	7	7	0.1	c.2.1.7
1hwy_15	33	2.934	7	1.00E+00	3.91E+18	2.934	7	1.00E+00	3.91E+18	7	7	0.4	c.2.1.7
1hwy_16	33	2.257	7	1.00E+00	1.21E+15	2.257	7	1.00E+00	1.21E+15	7	7	0.1	c.2.1.7
1hwy_17	33	2.251	7	1.00E+00	1.12E+15	2.251	7	1.00E+00	1.12E+15	7	7	0.1	c.2.1.7
1hwy_18	33	2.261	7	1.00E+00	1.28E+15	2.261	7	1.00E+00	1.28E+15	7	7	0.1	c.2.1.7
1c1d_0	43	0.899	11	6.86E-05	6.86E-05	0.899	11	6.86E-05	6.86E-05	11	13	2.2	c.2.1.7
1c1d_1	42	0.904	11	8.07E-05	8.07E-05	0.904	11	8.07E-05	8.07E-05	11	15	2.5	c.2.1.7
1c1x_0	42	0.946	12	5.84E-06	5.84E-06	0.946	12	5.84E-06	5.84E-06	12	14	2.0	c.2.1.7
1c1x_7	29	0.788	10	1.78E-05	1.78E-05	0.788	10	1.78E-05	1.78E-05	10	12	1.7	c.2.1.7
1bw9_0	45	0.929	12	3.06E-06	3.06E-06	0.929	12	3.06E-06	3.06E-06	12	14	2.3	c.2.1.7
1bw9_1	16	0.594	9	1.14E-07	1.14E-07	0.594	9	1.14E-07	1.14E-07	9	9	0.2	c.2.1.7
1bxg_0	42	0.920	11	1.70E-04	1.70E-04	0.920	11	1.70E-04	1.70E-04	11	15	2.8	c.2.1.7
1bxg_1	44	1.610	12	1.00E+00	2.92E+05	1.063	12	1.81E-03	1.82E-03	9	15	2.6	c.2.1.7
1a4i_0	26	2.728	7	1.00E+00	2.39E+16	2.675	8	1.00E+00	3.61E+15	7	9	0.6	c.2.1.7
1a4i_1	26	1.877	6	1.00E+00	1.41E+12	1.877	6	1.00E+00	1.41E+12	6	7	0.4	c.2.1.7
1dig_0	37	1.938	8	1.00E+00	1.41E+13	1.938	8	1.00E+00	1.41E+13	8	10	1.1	c.2.1.7
1dig_2	25	1.869	6	1.00E+00	1.14E+12	1.869	6	1.00E+00	1.14E+12	6	6	0.3	c.2.1.7
1dia_0	37	1.965	7	1.00E+00	1.15E+13	2.024	8	1.00E+00	2.84E+12	7	9	0.7	c.2.1.7
1dia_1	26	1.881	6	1.00E+00	1.47E+12	1.881	6	1.00E+00	1.47E+12	6	6	0.4	c.2.1.7
1dib_0	42	5.181	7	1.00E+00	2.46E+23	5.181	7	1.00E+00	2.46E+23	7	9	1.1	c.2.1.7
1dib_1	27	1.907	6	1.00E+00	2.16E+12	1.907	6	1.00E+00	2.16E+12	6	7	0.5	c.2.1.7
1ee9_0	23	2.792	7	1.00E+00	2.29E+16	2.792	7	1.00E+00	2.29E+16	7	7	0.3	c.2.1.7
1lua_0	26	2.973	6	1.00E+00	1.20E+18	2.973	6	1.00E+00	1.20E+18	6	7	0.9	c.2.1.7
1lua_1	25	2.091	7	1.00E+00	5.50E+12	2.091	7	1.00E+00	5.50E+12	7	7	0.1	c.2.1.7
1lua_2	25	1.496	6	1.00E+00	1.44E+10	1.582	7	1.00E+00	2.37E+09	6	7	0.3	c.2.1.7
1vi2_4	42	2.753	7	1.00E+00	9.94E+17	2.753	7	1.00E+00	9.94E+17	7	10	1.2	c.2.1.7
1vi2_8	42	2.956	7	1.00E+00	7.62E+18	2.956	7	1.00E+00	7.62E+18	7	10	1.4	c.2.1.7
1npd_3	44	6.309	8	1.00E+00	1.30E+29	6.309	8	1.00E+00	1.30E+29	8	11	1.6	c.2.1.7
1npd_5	43	1.607	8	1.00E+00	1.66E+10	1.607	8	1.00E+00	1.66E+10	8	9	0.4	c.2.1.7
1o9b_0	104	6.171	8	1.00E+00	6.67E+29	6.171	8	1.00E+00	6.67E+29	8	11	1.6	c.2.1.7
1o9b_1	118	1.521	9	1.00E+00	1.30E+09	1.576	10	1.00E+00	2.40E+08	9	10	0.2	c.2.1.7
1o9b_3	44	3.686	7	1.00E+00	3.68E+20	3.686	7	1.00E+00	3.68E+20	7	10	1.6	c.2.1.7
1o9b_6	36	1.699	7	1.00E+00	2.19E+11	1.699	7	1.00E+00	2.19E+11	7	8	0.3	c.2.1.7
1o9b_7	42	2.025	8	1.00E+00	1.06E+12	2.025	8	1.00E+00	1.06E+12	8	8	0.1	c.2.1.7
1nyt_0	40	1.374	11	1.00E+00	4.60E+03	0.785	10	9.74E-05	9.74E-05	8	11	1.8	c.2.1.7
1nyt_1	32	1.343	9	1.00E+00	5.78E+05	0.587	9	9.19E-07	9.19E-07	8	10	0.8	c.2.1.7

Table 27: Results for alcohol dehydrogenase matching against its own SCOP superfamily (but not family) with amino acid property.

Site	N	RMSD	q	Pvalue	Evalue	RMSD	q	Pvalue	Evalue	CG	Mean L	Var L	SCOP
1nyt_2	32	1.269	9	1.00E+00	6.95E+04	0.656	9	3.40E-05	3.40E-05	6	10	0.8	c.2.1.7
1nyt_14	32	1.637	11	1.00E+00	5.07E+06	0.789	10	5.91E-05	5.91E-05	8	10	0.8	c.2.1.7
1nvt_2	25	2.319	9	1.00E+00	7.17E+13	2.319	9	1.00E+00	7.17E+13	9	10	0.7	c.2.1.7
1nvt_3	26	4.264	8	1.00E+00	6.08E+21	4.264	8	1.00E+00	6.08E+21	8	9	0.6	c.2.1.7
1pj3_10	35	0.940	10	3.51E-02	3.57E-02	1.005	12	1.03E-04	1.03E-04	10	12	0.7	c.2.1.7
1pj3_11	36	0.913	10	1.23E-02	1.24E-02	0.841	11	5.49E-06	5.49E-06	9	12	0.8	c.2.1.7
1pj3_12	35	0.906	10	8.33E-03	8.36E-03	0.868	11	1.92E-05	1.92E-05	9	11	1.9	c.2.1.7
1pj3_13	34	0.878	10	2.22E-03	2.22E-03	0.810	11	9.37E-07	9.37E-07	9	11	2.6	c.2.1.7
1pj3_50	23	2.116	6	1.00E+00	3.36E+13	2.116	6	1.00E+00	3.36E+13	6	7	0.3	c.2.1.7
1pj3_51	23	2.207	6	1.00E+00	2.28E+14	2.207	6	1.00E+00	2.28E+14	6	6	0.3	c.2.1.7
1pj3_52	22	2.084	6	1.00E+00	2.10E+13	2.084	6	1.00E+00	2.10E+13	6	7	0.2	c.2.1.7
1pj3_53	23	8.803	6	1.00E+00	3.91E+28	8.803	6	1.00E+00	3.91E+28	6	7	0.5	c.2.1.7
1qr6_24	30	1.500	8	1.00E+00	3.51E+08	0.714	9	1.02E-03	1.02E-03	0	10	1.5	c.2.1.7
1qr6_25	16	6.227	5	1.00E+00	5.21E+22	6.227	5	1.00E+00	5.21E+22	5	5	0.4	c.2.1.7
1qr6_26	29	1.006	9	1.00E+00	2.00E+01	0.873	10	3.38E-03	3.39E-03	7	11	1.0	c.2.1.7
1qr6_27	16	6.231	5	1.00E+00	5.28E+22	6.231	5	1.00E+00	5.28E+22	5	5	0.4	c.2.1.7
1do8_10	36	0.943	10	4.32E-02	4.42E-02	0.835	11	4.05E-06	4.05E-06	9	12	0.8	c.2.1.7
1do8_11	35	1.010	10	5.51E-01	8.00E-01	0.866	11	1.74E-05	1.74E-05	9	12	0.9	c.2.1.7
1do8_12	35	0.925	10	1.88E-02	1.89E-02	0.816	10	3.63E-04	3.63E-04	8	11	2.5	c.2.1.7
1do8_13	34	0.919	10	1.33E-02	1.34E-02	0.829	11	2.50E-06	2.50E-06	9	12	0.9	c.2.1.7
1do8_50	21	8.862	6	1.00E+00	3.44E+28	8.862	6	1.00E+00	3.44E+28	6	7	0.5	c.2.1.7
1do8_51	21	5.477	6	1.00E+00	3.09E+22	5.477	6	1.00E+00	3.09E+22	6	7	0.3	c.2.1.7
1do8_52	20	1.503	6	1.00E+00	5.55E+09	1.503	6	1.00E+00	5.55E+09	6	6	0.0	c.2.1.7
1do8_53	22	8.874	6	1.00E+00	4.12E+28	8.874	6	1.00E+00	4.12E+28	6	7	0.5	c.2.1.7
1efl_10	36	0.939	10	3.67E-02	3.74E-02	0.847	11	7.41E-06	7.41E-06	9	12	0.9	c.2.1.7
1efl_11	37	0.905	10	9.46E-03	9.50E-03	0.819	11	1.94E-06	1.94E-06	9	12	0.8	c.2.1.7
1efl_12	35	1.559	9	1.00E+00	9.70E+07	0.874	11	2.57E-05	2.57E-05	6	11	1.0	c.2.1.7
1efl_13	36	0.870	10	1.84E-03	1.84E-03	0.755	11	5.69E-08	5.69E-08	9	12	0.8	c.2.1.7
1efl_50	21	2.143	6	1.00E+00	3.32E+13	2.143	6	1.00E+00	3.32E+13	6	7	0.2	c.2.1.7
1efl_51	21	8.948	6	1.00E+00	4.33E+28	8.948	6	1.00E+00	4.33E+28	6	7	0.6	c.2.1.7
1efl_52	22	8.944	6	1.00E+00	4.96E+28	8.944	6	1.00E+00	4.96E+28	6	7	0.5	c.2.1.7
1efl_53	21	1.921	6	1.00E+00	1.75E+12	1.921	6	1.00E+00	1.75E+12	6	6	0.1	c.2.1.7
1pjl_100	28	0.744	8	3.26E-01	3.95E-01	0.864	10	1.54E-03	1.54E-03	7	10	0.9	c.2.1.7
1pjl_101	15	2.429	5	1.00E+00	5.08E+14	2.429	5	1.00E+00	5.08E+14	5	6	0.3	c.2.1.7
1pjl_102	28	0.731	8	1.25E-01	1.33E-01	0.890	11	2.77E-05	2.77E-05	8	11	0.6	c.2.1.7
1pjl_103	15	3.094	5	1.00E+00	6.38E+16	3.094	5	1.00E+00	6.38E+16	5	5	0.2	c.2.1.7
1pjl_104	29	6.220	8	1.00E+00	4.62E+26	6.220	8	1.00E+00	4.62E+26	8	10	0.9	c.2.1.7
1pjl_105	15	4.284	6	1.00E+00	5.06E+19	4.284	6	1.00E+00	5.06E+19	6	6	0.0	c.2.1.7
1pjl_106	29	1.614	9	1.00E+00	3.22E+08	0.922	11	1.38E-04	1.38E-04	8	11	0.8	c.2.1.7
1pjl_107	15	2.079	5	1.00E+00	6.47E+13	2.079	5	1.00E+00	6.47E+13	5	5	0.0	c.2.1.7
1pjl_108	29	0.748	8	4.03E-01	5.17E-01	0.701	9	5.04E-04	5.04E-04	7	10	2.4	c.2.1.7
1pjl_109	15	2.542	5	1.00E+00	1.19E+15	2.542	5	1.00E+00	1.19E+15	5	6	0.3	c.2.1.7
1pjl_110	28	1.045	9	1.00E+00	6.64E+01	1.070	11	6.54E-02	6.77E-02	8	11	1.1	c.2.1.7
1pjl_111	16	2.084	5	1.00E+00	6.23E+13	2.084	5	1.00E+00	6.23E+13	5	6	0.3	c.2.1.7
1pjl_112	27	1.025	9	1.00E+00	3.05E+01	0.890	11	2.47E-05	2.47E-05	8	11	0.9	c.2.1.7
1pjl_113	16	2.565	5	1.00E+00	1.74E+15	2.565	5	1.00E+00	1.74E+15	5	6	0.3	c.2.1.7
1pjl_114	27	2.227	8	1.00E+00	1.35E+13	0.780	10	3.00E-05	3.00E-05	6	10	1.7	c.2.1.7
1pjl_115	16	2.461	5	1.00E+00	8.00E+14	2.461	5	1.00E+00	8.00E+14	5	6	0.3	c.2.1.7
1efk_10	37	1.497	9	1.00E+00	3.08E+07	0.886	11	5.43E-05	5.43E-05	6	11	0.9	c.2.1.7
1efk_11	36	1.485	9	1.00E+00	2.18E+07	0.877	11	3.24E-05	3.24E-05	6	12	1.0	c.2.1.7
1efk_12	35	0.991	10	3.15E-01	3.78E-01	0.894	11	6.69E-05	6.69E-05	9	12	0.9	c.2.1.7
1efk_13	35	1.496	9	1.00E+00	2.54E+07	0.828	11	2.60E-06	2.60E-06	6	12	0.9	c.2.1.7
1efk_50	20	1.586	5	1.00E+00	3.07E+11	1.586	5	1.00E+00	3.07E+11	5	5	0.0	c.2.1.7
1efk_51	20	2.344	5	1.00E+00	4.95E+14	2.264	6	1.00E+00	4.03E+13	5	6	0.1	c.2.1.7
1efk_52	21	1.557	6	1.00E+00	1.39E+10	1.557	6	1.00E+00	1.39E+10	6	6	0.0	c.2.1.7
1efk_53	20	2.718	5	1.00E+00	5.95E+15	2.718	5	1.00E+00	5.95E+15	5	6	0.3	c.2.1.7
1gq2_0	120	1.674	9	1.00E+00	1.54E+11	1.795	11	1.00E+00	1.07E+10	9	13	1.2	c.2.1.7
1gq2_1	120	1.349	12	1.00E+00	6.47E+03	1.349	12	1.00E+00	6.47E+03	12	13	0.5	c.2.1.7
1gq2_4	38	1.368	10	1.00E+00	9.14E+04	0.828	10	4.09E-04	4.09E-04	8	11	1.1	c.2.1.7
1gq2_6	120	1.682	9	1.00E+00	3.34E+10	1.768	10	1.00E+00	1.41E+10	9	11	1.1	c.2.1.7
1gq2_7	120	4.064	10	1.00E+00	3.09E+25	4.064	10	1.00E+00	3.09E+25	10	14	2.1	c.2.1.7
1gq2_10	38	1.238	10	1.00E+00	2.39E+03	0.736	9	1.69E-03	1.69E-03	7	11	1.4	c.2.1.7
1gq2_12	118	4.143	9	1.00E+00	4.09E+24	4.143	9	1.00E+00	4.09E+24	9	12	1.4	c.2.1.7
1gq2_13	120	1.671	10	1.00E+00	1.43E+10	1.671	10	1.00E+00	1.43E+10	10	13	2.3	c.2.1.7
1gq2_17	36	1.268	9	1.00E+00	1.14E+05	0.739	8	6.06E-01	9.31E-01	7	10	2.3	c.2.1.7

Table 28: Results for alcohol dehydrogenase matching against its own SCOP superfamily (but not family) with amino acid property.

Site	N	RMSD	q	Pvalue	Evalue	RMSD	q	Pvalue	Evalue	CG	Mean L	Var L	SCOP
1gq2_19	120	4.137	9	1.00E+00	4.10E+24	4.137	9	1.00E+00	4.10E+24	9	12	1.7	c.2.1.7
1gq2_20	120	1.686	10	1.00E+00	2.03E+10	1.686	10	1.00E+00	2.03E+10	10	13	2.1	c.2.1.7
1gq2_23	37	0.985	9	1.00E+00	2.08E+01	0.834	10	5.01E-04	5.01E-04	8	11	1.1	c.2.1.7
1gq2_25	120	9.568	9	1.00E+00	5.15E+40	9.568	9	1.00E+00	5.15E+40	9	14	2.5	c.2.1.7
1gq2_26	120	1.658	10	1.00E+00	1.05E+10	1.658	10	1.00E+00	1.05E+10	10	13	2.2	c.2.1.7
1gq2_29	39	1.212	10	1.00E+00	1.12E+03	0.805	10	1.46E-04	1.46E-04	8	10	0.9	c.2.1.7
1gq2_31	120	9.567	9	1.00E+00	5.12E+40	9.567	9	1.00E+00	5.12E+40	9	14	2.8	c.2.1.7
1gq2_32	120	4.065	10	1.00E+00	3.12E+25	4.065	10	1.00E+00	3.12E+25	10	14	2.1	c.2.1.7
1gq2_36	39	1.456	10	1.00E+00	8.76E+05	0.745	9	2.79E-03	2.79E-03	6	11	1.4	c.2.1.7
1gq2_38	120	5.477	9	1.00E+00	6.62E+28	5.477	9	1.00E+00	6.62E+28	9	13	1.5	c.2.1.7
1gq2_39	120	1.686	10	1.00E+00	2.03E+10	1.686	10	1.00E+00	2.03E+10	10	13	2.6	c.2.1.7
1gq2_42	37	0.956	9	9.99E-01	7.41E+00	0.830	10	4.14E-04	4.14E-04	8	11	1.1	c.2.1.7
1gq2_44	120	1.673	9	1.00E+00	2.02E+10	1.673	9	1.00E+00	2.02E+10	9	10	0.5	c.2.1.7
1gq2_45	120	4.059	10	1.00E+00	2.95E+25	4.059	10	1.00E+00	2.95E+25	10	14	2.0	c.2.1.7
1gq2_49	39	0.979	9	1.00E+00	1.98E+01	0.828	10	4.43E-04	4.43E-04	8	11	1.6	c.2.1.7
1gq2_51	111	4.107	9	1.00E+00	2.52E+24	4.107	9	1.00E+00	2.52E+24	9	12	1.6	c.2.1.7
1gq2_52	120	2.478	10	1.00E+00	1.05E+17	2.478	10	1.00E+00	1.05E+17	10	13	2.1	c.2.1.7
1gq2_56	36	0.926	9	8.96E-01	2.27E+00	0.854	10	1.17E-03	1.17E-03	8	10	1.1	c.2.1.7
1gq2_58	120	1.630	9	1.00E+00	2.63E+10	1.689	10	1.00E+00	5.94E+09	9	11	0.8	c.2.1.7
1gq2_59	120	1.572	10	1.00E+00	1.70E+09	1.572	10	1.00E+00	1.70E+09	10	12	2.3	c.2.1.7
1gq2_62	37	0.981	9	1.00E+00	1.81E+01	0.830	10	4.14E-04	4.14E-04	8	10	0.8	c.2.1.7
1gq2_63	120	4.155	9	1.00E+00	4.76E+24	4.155	9	1.00E+00	4.76E+24	9	12	1.6	c.2.1.7
1gq2_64	120	1.651	10	1.00E+00	8.89E+09	1.651	10	1.00E+00	8.89E+09	10	13	2.2	c.2.1.7
1gq2_67	36	0.991	9	1.00E+00	2.35E+01	0.438	5	1.00E+00	1.34E+04	5	10	2.7	c.2.1.7
1gq2_68	120	1.574	10	1.00E+00	4.24E+08	1.026	7	1.00E+00	2.73E+07	6	13	4.1	c.2.1.7
1gq2_69	120	1.695	10	1.00E+00	2.51E+10	1.695	10	1.00E+00	2.51E+10	10	13	2.3	c.2.1.7
1gq2_72	37	0.961	9	1.00E+00	8.87E+00	0.961	9	1.00E+00	8.87E+00	9	10	0.6	c.2.1.7
1gq2_75	120	3.062	9	1.00E+00	5.00E+20	3.062	9	1.00E+00	5.00E+20	9	14	2.5	c.2.1.7
1gq2_76	120	1.649	10	1.00E+00	8.47E+09	0.707	9	1.40E-02	1.41E-02	7	12	13.2	c.2.1.7
1gq2_79	36	1.219	10	1.00E+00	1.10E+03	1.219	10	1.00E+00	1.10E+03	10	11	0.6	c.2.1.7
1gq2_81	120	4.601	10	1.00E+00	1.77E+27	4.601	10	1.00E+00	1.77E+27	10	13	1.3	c.2.1.7
1gq2_87	120	1.632	9	1.00E+00	2.75E+10	1.645	11	1.00E+00	1.35E+08	9	11	0.7	c.2.1.7
1gq2_88	120	1.246	10	1.00E+00	1.35E+05	1.246	10	1.00E+00	1.35E+05	10	12	1.0	c.2.1.7
1gq2_91	36	1.268	10	1.00E+00	5.19E+03	0.860	10	1.54E-03	1.54E-03	8	10	0.9	c.2.1.7
1gq2_94	120	4.136	9	1.00E+00	4.06E+24	4.136	9	1.00E+00	4.06E+24	9	12	1.3	c.2.1.7
1gq2_95	120	4.037	10	1.00E+00	2.38E+25	4.037	10	1.00E+00	2.38E+25	10	14	1.9	c.2.1.7
1gq2_98	37	1.246	9	1.00E+00	6.80E+04	1.246	9	1.00E+00	6.80E+04	9	10	0.8	c.2.1.7
1llq_0	29	2.366	7	1.00E+00	1.01E+15	2.366	7	1.00E+00	1.01E+15	7	7	0.2	c.2.1.7
1llq_1	28	6.806	7	1.00E+00	1.99E+27	6.806	7	1.00E+00	1.99E+27	7	9	0.8	c.2.1.7
1lss_0	25	2.840	7	1.00E+00	1.04E+16	2.840	7	1.00E+00	1.04E+16	7	7	0.5	c.2.1.9
1lss_1	25	2.617	7	1.00E+00	1.23E+16	2.617	7	1.00E+00	1.23E+16	7	7	0.3	c.2.1.9
1xcb_0	35	1.307	10	1.00E+00	1.64E+04	0.711	9	8.10E-04	8.10E-04	9	11	1.6	c.2.1.12
1xcb_1	34	1.108	9	1.00E+00	1.02E+03	0.643	9	2.83E-05	2.83E-05	8	10	1.1	c.2.1.12
1xcb_2	37	1.055	10	9.37E-01	2.76E+00	0.833	10	5.28E-04	5.28E-04	9	12	1.3	c.2.1.12
1xcb_3	35	0.854	10	1.13E-03	1.13E-03	0.715	9	9.72E-04	9.72E-04	9	11	1.1	c.2.1.12
1xcb_4	37	0.848	9	2.00E-01	2.23E-01	0.674	9	1.69E-04	1.69E-04	8	11	1.3	c.2.1.12
1xcb_5	35	0.971	9	1.00E+00	1.53E+01	0.810	10	2.75E-04	2.75E-04	8	11	1.0	c.2.1.12
1xcb_6	35	0.711	9	8.10E-04	8.10E-04	0.711	9	8.10E-04	8.10E-04	9	12	1.3	c.2.1.12
1omo_2	28	3.734	7	1.00E+00	9.62E+19	3.679	8	1.00E+00	4.20E+19	7	8	0.5	c.2.1.13
1omo_3	27	3.412	6	1.00E+00	1.13E+19	3.412	6	1.00E+00	1.13E+19	6	8	0.7	c.2.1.13
