# Supplementary material for: Bayesian refinement of protein functional site matching
Source: BMC Bioinformatics. 2007 Jul 17;8:257. doi: 10.1186/1471-2105-8-257 (PMC1940029; doi:10.1186/1471-2105-8-257)
Supplement: Additional file 4 — Case 4 Results. Results for alcohol dehydrogenase and FAD/NAD(P)-binding domain. Tables 1–5: Without physico-chemistry. Tables 5–10: With physico-chemistry. [file 1471-2105-8-257-S4.pdf]

Results for alcohol dehydrogenase and FAD/NAD(P)-binding domain.

Table 1: Results for alcohol dehydrogenase matching against FAD/NAD(P)-binding domain without amino acid property.

Site	N	RMSD	q	Pvalue	Evalue	RMSD	q	Pvalue	Evalue	CG	Mean L	Var L	SCOP
1ps9_0	69	0.572	11	3.30E-09	3.30E-09	0.572	11	3.30E-09	3.30E-09	11	11	0.2	c.3.1.1
1cjc_0	42	0.963	10	1.00E+00	7.62E+01	0.963	11	8.71E-01	2.04E+00	10	11	0.4	c.3.1.1
1e1m_0	120	4.716	11	1.00E+00	4.18E+32	4.716	11	1.00E+00	4.18E+32	11	14	2.4	c.3.1.1
1e6e_0	120	2.832	10	1.00E+00	3.48E+19	2.832	10	1.00E+00	3.48E+19	10	14	2.6	c.3.1.1
1e1k_0	120	0.938	11	1.00E+00	1.64E+01	0.938	11	1.00E+00	1.64E+01	11	11	0.5	c.3.1.1
1e1n_0	92	0.970	10	1.00E+00	5.13E+02	0.970	10	1.00E+00	5.13E+02	10	12	1.2	c.3.1.1
1e1l_0	120	0.647	12	9.14E-09	9.14E-09	0.647	12	9.14E-09	9.14E-09	12	12	0.2	c.3.1.1
1lqt_0	82	1.742	12	1.00E+00	1.33E+10	1.742	12	1.00E+00	1.33E+10	12	14	1.2	c.3.1.1
1lqt_2	82	1.723	11	1.00E+00	1.17E+11	1.723	11	1.00E+00	1.17E+11	11	13	1.3	c.3.1.1
1lqu_0	81	1.749	12	1.00E+00	1.52E+10	1.749	12	1.00E+00	1.52E+10	12	14	1.2	c.3.1.1
1lqu_2	81	0.885	11	2.13E-01	2.39E-01	0.885	11	2.13E-01	2.39E-01	11	13	0.9	c.3.1.1
1h7w_0	120	2.235	10	1.00E+00	1.02E+18	2.241	11	1.00E+00	1.71E+17	10	12	0.8	c.3.1.1
1gte_0	120	1.464	11	1.00E+00	2.53E+09	1.397	12	1.00E+00	5.84E+07	11	13	1.4	c.3.1.1
1gte_1	120	5.768	10	1.00E+00	9.84E+37	5.768	10	1.00E+00	9.84E+37	10	14	2.6	c.3.1.1
1gte_5	120	1.493	11	1.00E+00	1.15E+09	1.493	11	1.00E+00	1.15E+09	11	14	1.9	c.3.1.1
1gte_6	120	5.764	10	1.00E+00	9.55E+37	5.764	10	1.00E+00	9.55E+37	10	14	2.5	c.3.1.1
1h7x_0	120	2.146	11	1.00E+00	2.98E+18	2.146	11	1.00E+00	2.98E+18	11	12	0.4	c.3.1.1
1h7x_3	67	3.524	10	1.00E+00	1.37E+22	3.524	10	1.00E+00	1.37E+22	10	13	2.1	c.3.1.1
1h7x_5	67	3.532	10	1.00E+00	1.48E+22	3.532	10	1.00E+00	1.48E+22	10	13	2.2	c.3.1.1
1h7x_8	65	3.532	10	1.00E+00	1.35E+22	3.532	10	1.00E+00	1.35E+22	10	13	2.2	c.3.1.1
1h7x_10	66	3.532	10	1.00E+00	1.42E+22	3.532	10	1.00E+00	1.42E+22	10	13	2.1	c.3.1.1
1gth_0	120	1.502	11	1.00E+00	1.46E+09	1.502	11	1.00E+00	1.46E+09	11	14	1.9	c.3.1.1
1gth_1	120	1.631	11	1.00E+00	1.48E+12	1.631	11	1.00E+00	1.48E+12	11	12	0.5	c.3.1.1
1gth_3	64	3.555	10	1.00E+00	1.62E+22	3.555	10	1.00E+00	1.62E+22	10	13	2.1	c.3.1.1
1gth_6	64	1.141	11	1.00E+00	9.77E+03	1.139	12	1.00E+00	1.89E+02	11	12	0.4	c.3.1.1
1gth_7	120	1.491	11	1.00E+00	1.09E+09	1.491	11	1.00E+00	1.09E+09	11	14	1.8	c.3.1.1
1gth_8	120	1.672	10	1.00E+00	1.09E+13	1.672	10	1.00E+00	1.09E+13	10	11	0.7	c.3.1.1
1gth_10	65	3.565	10	1.00E+00	1.88E+22	3.565	10	1.00E+00	1.88E+22	10	13	2.1	c.3.1.1
1gth_13	64	0.916	11	3.74E-01	4.69E-01	0.951	12	2.58E-02	2.62E-02	11	12	0.5	c.3.1.1
1gt8_0	120	1.413	11	1.00E+00	5.64E+08	1.413	11	1.00E+00	5.64E+08	11	13	1.4	c.3.1.1
1gt8_1	120	1.766	11	1.00E+00	7.12E+12	1.766	11	1.00E+00	7.12E+12	11	14	1.5	c.3.1.1
1gt8_3	65	2.004	10	1.00E+00	2.36E+14	2.004	10	1.00E+00	2.36E+14	10	13	1.7	c.3.1.1
1gt8_6	66	3.540	10	1.00E+00	1.53E+22	3.540	10	1.00E+00	1.53E+22	10	13	1.8	c.3.1.1
1gt8_7	120	1.431	11	1.00E+00	9.63E+08	1.431	11	1.00E+00	9.63E+08	11	13	1.5	c.3.1.1
1gt8_8	120	6.401	11	1.00E+00	3.44E+36	6.401	11	1.00E+00	3.44E+36	11	13	1.7	c.3.1.1
1gt8_10	66	1.973	10	1.00E+00	1.38E+14	1.973	10	1.00E+00	1.38E+14	10	14	1.7	c.3.1.1
1gt8_13	65	3.538	10	1.00E+00	1.44E+22	3.538	10	1.00E+00	1.44E+22	10	13	2.0	c.3.1.1
3cox_0	45	2.867	10	1.00E+00	5.06E+19	2.867	10	1.00E+00	5.06E+19	10	13	1.2	c.3.1.2
1coy_0	61	0.729	11	3.35E-05	3.35E-05	0.729	11	3.35E-05	3.35E-05	11	11	0.3	c.3.1.2
1n4w_1	54	5.091	10	1.00E+00	1.90E+29	5.091	10	1.00E+00	1.90E+29	10	16	2.2	c.3.1.2
1n1p_3	54	5.094	10	1.00E+00	1.94E+29	5.094	10	1.00E+00	1.94E+29	10	16	2.2	c.3.1.2
1n4v_0	55	5.094	10	1.00E+00	2.05E+29	5.094	10	1.00E+00	2.05E+29	10	16	2.2	c.3.1.2
1ijh_0	47	3.323	10	1.00E+00	5.78E+20	3.323	10	1.00E+00	5.78E+20	10	16	2.3	c.3.1.2
1b4v_0	72	2.635	11	1.00E+00	8.58E+21	2.635	11	1.00E+00	8.58E+21	11	17	2.8	c.3.1.2
1b8s_0	68	1.602	10	1.00E+00	3.61E+11	1.602	10	1.00E+00	3.61E+11	10	15	2.4	c.3.1.2
1cbo_0	71	0.780	11	7.11E-04	7.12E-04	0.780	11	7.11E-04	7.12E-04	11	12	0.6	c.3.1.2
1cc2_0	71	0.757	11	2.26E-04	2.26E-04	0.757	11	2.26E-04	2.26E-04	11	12	0.6	c.3.1.2
1cf3_4	47	2.237	9	1.00E+00	3.14E+15	2.237	9	1.00E+00	3.14E+15	9	13	2.3	c.3.1.2
1gal_0	48	2.045	10	1.00E+00	2.22E+12	2.045	10	1.00E+00	2.22E+12	10	14	2.0	c.3.1.2
1gpe_8	50	3.466	9	1.00E+00	5.72E+21	3.466	9	1.00E+00	5.72E+21	9	11	1.0	c.3.1.2
1gpe_9	50	5.384	10	1.00E+00	1.22E+30	5.384	10	1.00E+00	1.22E+30	10	16	2.2	c.3.1.2
1ju2_8	48	1.607	9	1.00E+00	4.21E+11	1.607	9	1.00E+00	4.21E+11	9	13	2.1	c.3.1.2
1ju2_9	48	1.597	9	1.00E+00	3.40E+11	1.597	9	1.00E+00	3.40E+11	9	13	2.0	c.3.1.2
1pbe_0	51	1.289	11	1.00E+00	3.22E+06	1.349	13	1.00E+00	2.84E+04	11	14	1.1	c.3.1.2
1bgn_0	51	1.292	11	1.00E+00	3.57E+06	1.285	12	1.00E+00	8.33E+04	11	14	1.2	c.3.1.2
1bkw_0	51	1.924	9	1.00E+00	3.01E+13	1.924	9	1.00E+00	3.01E+13	9	9	0.3	c.3.1.2
1pbd_0	50	0.933	10	1.00E+00	3.98E+01	0.933	10	1.00E+00	3.98E+01	10	12	0.9	c.3.1.2
1cc6_0	82	1.269	11	1.00E+00	6.84E+06	1.262	12	1.00E+00	1.51E+05	11	14	1.2	c.3.1.2
1cj4_0	83	1.270	11	1.00E+00	7.35E+06	1.265	12	1.00E+00	1.75E+05	11	14	1.4	c.3.1.2
1bf3_0	51	1.259	11	1.00E+00	1.13E+06	1.250	12	1.00E+00	2.26E+04	11	14	1.3	c.3.1.2
1cj3_0	82	0.572	10	3.66E-06	3.66E-06	0.572	10	3.66E-06	3.66E-06	10	11	0.7	c.3.1.2
1pbb_0	48	0.968	10	1.00E+00	1.39E+02	0.908	11	2.25E-01	2.55E-01	10	12	1.1	c.3.1.2
1pbc_0	48	1.443	9	1.00E+00	4.85E+10	1.574	13	1.00E+00	9.71E+06	9	15	1.4	c.3.1.2
1pbf_0	46	0.974	10	1.00E+00	1.54E+02	0.974	10	1.00E+00	1.54E+02	10	12	1.0	c.3.1.2
1cj2_0	81	0.578	10	5.00E-06	5.00E-06	0.645	11	7.26E-07	7.26E-07	10	11	0.6	c.3.1.2
1cc4_0	83	0.952	11	9.08E-01	2.38E+00	0.901	12	5.56E-03	5.57E-03	11	13	0.7	c.3.1.2
1bgj_0	51	1.139	10	1.00E+00	2.17E+05	1.114	11	1.00E+00	1.77E+03	10	13	1.2	c.3.1.2
1phh_0	52	1.353	11	1.00E+00	2.93E+07	1.383	12	1.00E+00	2.85E+06	11	14	1.5	c.3.1.2
1k0i_0	43	1.751	10	1.00E+00	2.06E+13	1.848	11	1.00E+00	1.66E+13	10	11	0.4	c.3.1.2

Table 2: Results for alcohol dehydrogenase matching against FAD/NAD(P)-binding domain without amino acid property.

Site	N	RMSD	q	Pvalue	Evalue	RMSD	q	Pvalue	Evalue	CG	Mean L	Var L	SCOP
1iut_0	49	1.285	11	1.00E+00	2.48E+06	1.278	12	1.00E+00	5.69E+04	11	14	1.3	c.3.1.2
1iux_0	50	1.209	11	1.00E+00	1.77E+05	1.272	13	1.00E+00	1.40E+03	11	15	1.5	c.3.1.2
1iuw_0	50	0.798	11	8.44E-04	8.45E-04	0.798	11	8.44E-04	8.45E-04	11	13	0.9	c.3.1.2
1doc_0	53	1.186	10	1.00E+00	1.20E+06	1.279	12	1.00E+00	2.03E+04	10	13	1.3	c.3.1.2
1iuu_0	50	1.272	11	1.00E+00	1.68E+06	1.266	12	1.00E+00	3.88E+04	11	14	1.2	c.3.1.2
1k0l_0	39	1.386	10	1.00E+00	9.51E+08	0.650	9	6.69E-03	6.72E-03	4	14	2.7	c.3.1.2
1dod_0	48	0.790	10	6.72E-02	6.95E-02	0.790	10	6.72E-02	6.95E-02	10	12	1.1	c.3.1.2
1pxc_0	49	1.326	11	1.00E+00	9.99E+06	1.266	13	1.00E+00	3.76E+03	11	15	1.4	c.3.1.2
1dob_0	50	0.794	10	9.08E-02	9.52E-02	0.794	10	9.08E-02	9.52E-02	10	12	1.1	c.3.1.2
1ius_0	50	1.293	11	1.00E+00	3.48E+06	1.286	12	1.00E+00	8.13E+04	11	15	1.4	c.3.1.2
1doe_0	52	0.807	10	1.79E-01	1.97E-01	0.807	10	1.79E-01	1.97E-01	10	12	0.9	c.3.1.2
1pxb_0	49	1.216	12	1.00E+00	1.82E+04	1.213	13	1.00E+00	4.04E+02	12	14	1.1	c.3.1.2
1pxa_0	49	1.431	11	1.00E+00	2.93E+08	1.432	12	1.00E+00	1.23E+07	11	15	1.4	c.3.1.2
1iuv_0	50	1.304	11	1.00E+00	5.06E+06	1.301	12	1.00E+00	1.41E+05	11	15	1.4	c.3.1.2
1k0j_0	49	1.394	9	1.00E+00	7.56E+08	1.583	11	1.00E+00	1.41E+08	9	12	1.2	c.3.1.2
1el5_0	50	1.271	10	1.00E+00	1.54E+07	1.257	11	1.00E+00	2.77E+05	10	12	0.8	c.3.1.2
1el5_1	51	2.528	9	1.00E+00	4.49E+19	2.528	9	1.00E+00	4.49E+19	9	12	1.8	c.3.1.2
1l9e_0	48	1.065	10	1.00E+00	4.97E+03	1.065	10	1.00E+00	4.97E+03	10	11	0.8	c.3.1.2
1l9e_1	49	1.229	10	1.00E+00	3.29E+05	1.173	11	1.00E+00	4.47E+03	10	12	0.6	c.3.1.2
1l9f_0	44	1.603	10	1.00E+00	1.68E+10	1.603	10	1.00E+00	1.68E+10	10	11	0.6	c.3.1.2
1l9f_1	45	2.459	9	1.00E+00	5.94E+16	2.623	10	1.00E+00	1.16E+17	9	13	1.8	c.3.1.2
1l9c_0	45	1.352	10	1.00E+00	1.27E+08	1.327	11	1.00E+00	1.99E+06	10	12	0.7	c.3.1.2
1l9c_1	44	1.353	9	1.00E+00	2.06E+08	1.353	9	1.00E+00	2.06E+08	9	11	1.5	c.3.1.2
1el7_0	50	2.515	9	1.00E+00	3.50E+19	2.515	9	1.00E+00	3.50E+19	9	12	1.7	c.3.1.2
1el7_1	51	2.512	9	1.00E+00	3.56E+19	2.512	9	1.00E+00	3.56E+19	9	12	1.8	c.3.1.2
1l9d_0	50	2.849	10	1.00E+00	1.02E+21	2.849	10	1.00E+00	1.02E+21	10	11	0.8	c.3.1.2
1l9d_1	51	1.770	9	1.00E+00	1.01E+14	1.770	9	1.00E+00	1.01E+14	9	10	1.0	c.3.1.2
1el8_0	50	1.428	9	1.00E+00	1.76E+09	1.428	9	1.00E+00	1.76E+09	9	10	0.4	c.3.1.2
1el8_1	51	2.521	9	1.00E+00	4.06E+19	2.521	9	1.00E+00	4.06E+19	9	12	1.8	c.3.1.2
1eli_0	73	4.754	10	1.00E+00	9.62E+31	4.754	10	1.00E+00	9.62E+31	10	15	2.1	c.3.1.2
1eli_3	71	5.991	10	1.00E+00	1.57E+34	5.991	10	1.00E+00	1.57E+34	10	14	2.3	c.3.1.2
1el9_0	73	5.746	11	1.00E+00	5.90E+35	5.746	11	1.00E+00	5.90E+35	11	15	2.2	c.3.1.2
1el9_3	72	4.290	10	1.00E+00	3.14E+28	4.290	10	1.00E+00	3.14E+28	10	14	1.9	c.3.1.2
1ng4_0	52	0.558	10	3.99E-07	3.99E-07	0.558	10	3.99E-07	3.99E-07	10	12	1.2	c.3.1.2
1ng4_1	51	0.982	10	1.00E+00	6.01E+01	1.191	11	1.00E+00	9.04E+02	10	12	1.1	c.3.1.2
1ng3_0	53	1.525	10	1.00E+00	3.14E+07	1.824	11	1.00E+00	7.22E+08	10	13	1.3	c.3.1.2
1ng3_1	55	0.775	11	2.55E-04	2.55E-04	0.775	11	2.55E-04	2.55E-04	11	12	0.5	c.3.1.2
1pn0_0	53	1.148	10	1.00E+00	3.74E+04	1.501	13	1.00E+00	5.11E+04	10	14	2.4	c.3.1.2
1pn0_1	53	1.135	10	1.00E+00	2.50E+04	1.490	13	1.00E+00	3.69E+04	10	14	2.1	c.3.1.2
1pn0_6	39	0.826	9	1.00E+00	1.55E+01	0.819	10	8.89E-02	9.31E-02	9	13	1.4	c.3.1.2
1pn0_8	39	0.545	9	3.12E-05	3.12E-05	0.545	9	3.12E-05	3.12E-05	9	12	1.7	c.3.1.2
1foh_0	53	1.156	10	1.00E+00	4.78E+04	1.516	13	1.00E+00	7.93E+04	10	14	2.1	c.3.1.2
1foh_1	54	2.326	10	1.00E+00	4.33E+17	2.326	10	1.00E+00	4.33E+17	10	12	1.5	c.3.1.2
1foh_2	39	1.620	9	1.00E+00	4.94E+10	1.586	10	1.00E+00	1.37E+09	9	13	0.9	c.3.1.2
1foh_4	39	0.839	9	1.00E+00	2.57E+01	0.833	10	1.56E-01	1.70E-01	9	13	1.5	c.3.1.2
1h82_0	120	0.710	11	1.08E-04	1.08E-04	0.710	11	1.08E-04	1.08E-04	11	11	0.3	c.3.1.2
1h82_1	62	0.662	10	2.05E-04	2.05E-04	0.662	10	2.05E-04	2.05E-04	10	11	0.5	c.3.1.2
1h82_2	62	0.636	10	4.96E-05	4.96E-05	0.636	10	4.96E-05	4.96E-05	10	11	0.5	c.3.1.2
1h83_0	120	5.005	10	1.00E+00	1.14E+30	5.005	10	1.00E+00	1.14E+30	10	14	2.1	c.3.1.2
1h83_1	57	0.689	10	7.03E-04	7.04E-04	0.689	10	7.03E-04	7.04E-04	10	11	0.5	c.3.1.2
1h83_2	56	0.641	10	4.47E-05	4.47E-05	0.641	10	4.47E-05	4.47E-05	10	11	0.5	c.3.1.2
1b5q_0	53	0.717	11	1.57E-05	1.57E-05	0.717	11	1.57E-05	1.57E-05	11	11	0.2	c.3.1.2
1b5q_1	53	1.251	10	1.00E+00	9.86E+06	1.251	10	1.00E+00	9.86E+06	10	11	0.9	c.3.1.2
1b5q_2	55	1.329	11	1.00E+00	1.57E+07	1.387	12	1.00E+00	3.88E+06	11	14	1.2	c.3.1.2
1b37_3	45	0.709	11	5.90E-06	5.90E-06	0.709	11	5.90E-06	5.90E-06	11	11	0.2	c.3.1.2
1b37_4	46	0.676	10	1.79E-04	1.79E-04	0.676	10	1.79E-04	1.79E-04	10	11	0.4	c.3.1.2
1b37_5	46	0.642	10	2.60E-05	2.60E-05	0.642	10	2.60E-05	2.60E-05	10	11	0.4	c.3.1.2
1h86_0	120	0.650	10	7.97E-04	7.97E-04	0.650	10	7.97E-04	7.97E-04	10	11	1.0	c.3.1.2
1h86_1	52	0.664	10	1.33E-04	1.33E-04	0.664	10	1.33E-04	1.33E-04	10	11	0.4	c.3.1.2
1h86_2	52	0.648	10	5.35E-05	5.35E-05	0.648	10	5.35E-05	5.35E-05	10	11	0.3	c.3.1.2
1h84_0	120	2.267	10	1.00E+00	1.53E+17	2.267	10	1.00E+00	1.53E+17	10	14	2.2	c.3.1.2
1h84_1	52	0.922	10	1.00E+00	2.88E+01	0.922	10	1.00E+00	2.88E+01	10	11	0.7	c.3.1.2
1h84_2	54	0.631	10	2.22E-05	2.22E-05	0.631	10	2.22E-05	2.22E-05	10	11	0.4	c.3.1.2
1h81_0	120	0.949	11	1.00E+00	1.32E+01	0.949	11	1.00E+00	1.32E+01	11	13	1.1	c.3.1.2
1h81_1	120	0.648	10	7.14E-04	7.15E-04	0.648	10	7.14E-04	7.15E-04	10	11	0.6	c.3.1.2
1f8r_4	52	0.669	11	7.86E-07	7.86E-07	0.669	11	7.86E-07	7.86E-07	11	12	0.6	c.3.1.2
1f8r_5	53	0.670	11	8.87E-07	8.87E-07	0.670	11	8.87E-07	8.87E-07	11	12	0.6	c.3.1.2
1f8r_6	52	0.664	11	5.72E-07	5.72E-07	0.664	11	5.72E-07	5.72E-07	11	12	0.5	c.3.1.2
1f8r_7	52	0.672	11	9.49E-07	9.49E-07	0.672	11	9.49E-07	9.49E-07	11	12	0.5	c.3.1.2

Table 3: Results for alcohol dehydrogenase matching against FAD/NAD(P)-binding domain without amino acid property.

Site	N	RMSD	q	Pvalue	Evalue	RMSD	q	Pvalue	Evalue	CG	Mean L	Var L	SCOP
1f8s_0	59	1.089	12	1.00E+00	5.20E+01	1.089	12	1.00E+00	5.20E+01	12	13	0.4	c.3.1.2
1f8s_1	59	0.956	12	1.04E-01	1.10E-01	0.956	12	1.04E-01	1.10E-01	12	13	0.6	c.3.1.2
1f8s_2	59	0.961	12	1.31E-01	1.41E-01	0.961	12	1.31E-01	1.41E-01	12	13	0.6	c.3.1.2
1f8s_3	59	0.969	12	1.88E-01	2.08E-01	0.969	12	1.88E-01	2.08E-01	12	13	0.6	c.3.1.2
1f8s_4	59	0.959	12	1.20E-01	1.27E-01	0.959	12	1.20E-01	1.27E-01	12	13	0.6	c.3.1.2
1f8s_5	58	0.950	12	7.46E-02	7.75E-02	0.950	12	7.46E-02	7.75E-02	12	13	0.6	c.3.1.2
1f8s_6	59	0.657	11	5.37E-07	5.37E-07	0.657	11	5.37E-07	5.37E-07	11	12	0.6	c.3.1.2
1f8s_7	59	0.976	12	2.54E-01	2.93E-01	0.976	12	2.54E-01	2.93E-01	12	13	0.6	c.3.1.2
1reo_0	49	0.590	11	3.20E-09	3.20E-09	0.590	11	3.20E-09	3.20E-09	11	12	0.4	c.3.1.2
1tdn_0	49	0.574	10	4.77E-07	4.77E-07	0.574	10	4.77E-07	4.77E-07	10	11	0.4	c.3.1.2
1tdk_0	53	0.611	10	6.28E-06	6.28E-06	0.611	10	6.28E-06	6.28E-06	10	11	0.5	c.3.1.2
1tdo_0	51	0.675	11	1.08E-06	1.08E-06	0.675	11	1.08E-06	1.08E-06	11	11	0.3	c.3.1.2
1oja_0	120	1.113	12	1.00E+00	4.48E+02	1.113	12	1.00E+00	4.48E+02	12	13	0.6	c.3.1.2
1oja_1	57	1.054	11	1.00E+00	9.47E+01	1.054	11	1.00E+00	9.47E+01	11	12	0.7	c.3.1.2
1ojb_0	120	1.104	12	1.00E+00	3.10E+02	1.104	12	1.00E+00	3.10E+02	12	13	0.4	c.3.1.2
1ojb_1	116	1.015	11	1.00E+00	1.79E+02	1.015	11	1.00E+00	1.79E+02	11	12	0.6	c.3.1.2
1oj9_0	120	1.213	13	1.00E+00	1.84E+03	1.213	13	1.00E+00	1.84E+03	13	14	0.4	c.3.1.2
1ojc_0	120	1.115	12	1.00E+00	4.86E+02	1.115	12	1.00E+00	4.86E+02	12	13	0.5	c.3.1.2
1ojc_1	54	1.045	11	1.00E+00	5.68E+01	1.045	11	1.00E+00	5.68E+01	11	12	0.7	c.3.1.2
1gos_0	120	1.316	11	1.00E+00	2.77E+07	1.311	13	1.00E+00	2.34E+04	11	15	1.5	c.3.1.2
1gos_1	120	1.293	11	1.00E+00	1.31E+07	1.295	13	1.00E+00	1.30E+04	11	15	1.5	c.3.1.2
1ojd_0	120	1.215	14	1.00E+00	1.69E+02	1.215	14	1.00E+00	1.69E+02	14	15	0.4	c.3.1.2
1ojd_1	120	1.286	13	1.00E+00	3.47E+04	1.286	13	1.00E+00	3.47E+04	13	14	1.0	c.3.1.2
1ojd_2	120	1.135	12	1.00E+00	1.09E+03	1.135	12	1.00E+00	1.09E+03	12	14	1.2	c.3.1.2
1ojd_3	120	1.294	13	1.00E+00	4.74E+04	1.294	13	1.00E+00	4.74E+04	13	14	0.9	c.3.1.2
1ojd_4	120	1.134	13	1.00E+00	6.27E+01	1.134	13	1.00E+00	6.27E+01	13	14	0.6	c.3.1.2
1ojd_5	120	1.214	14	1.00E+00	1.62E+02	1.167	15	7.18E-01	1.27E+00	14	15	0.3	c.3.1.2
1ojd_6	120	1.333	14	1.00E+00	2.80E+04	1.333	14	1.00E+00	2.80E+04	14	15	0.6	c.3.1.2
1ojd_7	120	1.288	13	1.00E+00	3.75E+04	1.288	13	1.00E+00	3.75E+04	13	14	0.9	c.3.1.2
1ojd_8	120	1.128	13	1.00E+00	4.81E+01	1.087	14	3.06E-01	3.65E-01	13	14	0.4	c.3.1.2
1ojd_9	120	1.139	12	1.00E+00	1.28E+03	1.139	12	1.00E+00	1.28E+03	12	13	0.9	c.3.1.2
1o5w_0	64	1.608	10	1.00E+00	5.94E+10	1.608	10	1.00E+00	5.94E+10	10	11	0.7	c.3.1.2
1o5w_1	62	1.941	10	1.00E+00	8.47E+11	2.044	11	1.00E+00	5.52E+11	10	12	0.9	c.3.1.2
1o5w_2	63	1.607	10	1.00E+00	5.53E+10	1.607	10	1.00E+00	5.53E+10	10	11	0.9	c.3.1.2
1o5w_3	64	1.941	10	1.00E+00	9.33E+11	2.049	11	1.00E+00	6.64E+11	10	12	0.9	c.3.1.2
1pj5_0	49	1.048	11	1.00E+00	4.74E+01	1.048	11	1.00E+00	4.74E+01	11	12	0.6	c.3.1.2
1pj6_1	45	0.998	11	9.94E-01	5.08E+00	0.998	11	9.94E-01	5.08E+00	11	12	0.8	c.3.1.2
1pj7_2	46	1.059	12	9.22E-01	2.55E+00	1.059	12	9.22E-01	2.55E+00	12	14	0.9	c.3.1.2
1sez_0	92	0.898	11	4.70E-01	6.34E-01	0.898	11	4.70E-01	6.34E-01	11	12	0.9	c.3.1.2
1sez_3	80	3.535	11	1.00E+00	3.43E+26	3.508	12	1.00E+00	1.37E+26	11	15	2.3	c.3.1.2
1knr_0	52	1.172	9	1.00E+00	1.01E+07	1.172	9	1.00E+00	1.01E+07	9	9	0.3	c.3.1.4
1knp_0	59	5.100	11	1.00E+00	2.64E+31	5.100	11	1.00E+00	2.64E+31	11	14	1.4	c.3.1.4
1nek_0	56	0.939	11	5.72E-01	8.48E-01	0.501	9	7.27E-06	7.27E-06	9	17	18.8	c.3.1.4
1nen_0	57	0.941	11	6.23E-01	9.76E-01	0.502	8	7.52E-03	7.55E-03	7	18	15.1	c.3.1.4
1kf6_0	56	0.535	11	1.18E-10	1.18E-10	0.535	11	1.18E-10	1.18E-10	11	12	0.4	c.3.1.4
1kf6_1	56	1.440	10	1.00E+00	1.35E+08	1.440	10	1.00E+00	1.35E+08	10	12	1.7	c.3.1.4
1l0v_0	56	1.147	10	1.00E+00	4.29E+04	1.147	10	1.00E+00	4.29E+04	10	12	1.2	c.3.1.4
1l0v_1	50	1.101	10	1.00E+00	1.95E+04	1.081	11	1.00E+00	1.76E+02	10	12	0.6	c.3.1.4
1kfy_0	55	2.307	11	1.00E+00	4.53E+15	2.290	12	1.00E+00	5.43E+14	11	15	1.9	c.3.1.4
1kfy_1	54	1.176	10	1.00E+00	9.29E+04	1.137	11	1.00E+00	1.71E+03	10	12	1.2	c.3.1.4
1qla_5	50	0.971	11	6.62E-01	1.09E+00	0.971	12	1.41E-02	1.42E-02	11	13	0.3	c.3.1.4
1qla_7	50	0.972	11	6.77E-01	1.13E+00	0.971	12	1.41E-02	1.42E-02	11	13	0.3	c.3.1.4
1qlb_2	53	0.946	11	6.20E-01	9.67E-01	0.941	13	1.11E-04	1.11E-04	11	13	0.1	c.3.1.4
1qlb_6	53	0.946	11	6.20E-01	9.67E-01	0.941	13	1.11E-04	1.11E-04	11	13	0.1	c.3.1.4
1e7p_1	103	2.384	12	1.00E+00	5.75E+20	2.384	12	1.00E+00	5.75E+20	12	14	1.6	c.3.1.4
1e7p_2	55	0.872	11	2.32E-02	2.35E-02	0.894	12	6.27E-04	6.27E-04	11	13	0.4	c.3.1.4
1e7p_5	103	2.384	12	1.00E+00	5.75E+20	2.384	12	1.00E+00	5.75E+20	12	14	1.6	c.3.1.4
1e7p_6	55	0.872	11	2.32E-02	2.35E-02	0.893	12	5.98E-04	5.99E-04	11	13	0.4	c.3.1.4
1e7p_8	103	2.384	12	1.00E+00	5.75E+20	2.384	12	1.00E+00	5.75E+20	12	14	1.5	c.3.1.4
1e7p_9	55	0.872	11	2.32E-02	2.35E-02	0.894	12	6.27E-04	6.27E-04	11	13	0.4	c.3.1.4
1e7p_12	103	2.384	12	1.00E+00	5.75E+20	2.384	12	1.00E+00	5.75E+20	12	14	1.6	c.3.1.4
1e7p_13	55	1.371	11	1.00E+00	1.47E+07	1.387	12	1.00E+00	8.91E+05	11	13	0.7	c.3.1.4
1q9i_1	59	1.706	12	1.00E+00	1.30E+10	1.706	12	1.00E+00	1.30E+10	12	15	1.4	c.3.1.4
1kss_1	58	1.251	11	1.00E+00	2.83E+04	1.491	12	1.00E+00	1.09E+06	11	15	1.8	c.3.1.4
1m64_1	57	1.272	11	1.00E+00	5.09E+04	1.513	12	1.00E+00	1.89E+06	11	15	1.8	c.3.1.4
1m64_3	56	1.281	11	1.00E+00	6.32E+04	1.515	12	1.00E+00	1.89E+06	11	15	1.7	c.3.1.4
1e39_1	113	1.969	11	1.00E+00	6.78E+13	1.969	11	1.00E+00	6.78E+13	11	15	3.1	c.3.1.4
1e39_2	58	1.716	12	1.00E+00	1.61E+10	1.716	12	1.00E+00	1.61E+10	12	15	1.5	c.3.1.4
1ksu_1	59	1.728	12	1.00E+00	2.32E+10	1.787	13	1.00E+00	8.29E+09	12	15	1.4	c.3.1.4

Table 4: Results for alcohol dehydrogenase matching against FAD/NAD(P)-binding domain without amino acid property.

Site	N	RMSD	q	Pvalue	Evalue	RMSD	q	Pvalue	Evalue	CG	Mean L	Var L	SCOP
1ksu_3	59	1.765	12	1.00E+00	6.06E+10	1.823	13	1.00E+00	2.17E+10	12	15	1.5	c.3.1.4
1jry_2	59	1.664	11	1.00E+00	1.06E+10	1.760	12	1.00E+00	7.59E+09	11	14	1.4	c.3.1.4
1jry_3	61	1.820	11	1.00E+00	4.35E+11	1.820	11	1.00E+00	4.35E+11	11	15	1.9	c.3.1.4
1lj1_1	58	1.383	12	1.00E+00	2.12E+05	1.383	13	1.00E+00	7.82E+03	12	15	1.4	c.3.1.4
1lj1_3	57	1.091	12	1.00E+00	1.89E+01	1.218	14	8.32E-01	1.78E+00	12	16	1.1	c.3.1.4
1jrz_2	59	1.716	12	1.00E+00	1.69E+10	1.716	12	1.00E+00	1.69E+10	12	15	1.3	c.3.1.4
1jrz_3	58	1.674	11	1.00E+00	1.28E+10	1.674	11	1.00E+00	1.28E+10	11	14	1.5	c.3.1.4
1jrx_2	59	2.305	10	1.00E+00	4.78E+18	2.230	14	1.00E+00	6.05E+14	10	15	1.3	c.3.1.4
1jrx_3	60	1.360	10	1.00E+00	1.63E+09	1.423	12	1.00E+00	1.70E+07	10	13	0.6	c.3.1.4
1p2e_2	60	1.805	12	1.00E+00	1.76E+11	1.844	13	1.00E+00	3.96E+10	12	16	1.7	c.3.1.4
1p2h_1	59	1.653	11	1.00E+00	8.09E+09	1.653	11	1.00E+00	8.09E+09	11	14	1.6	c.3.1.4
1qjd_1	112	1.594	10	1.00E+00	2.34E+11	1.611	11	1.00E+00	2.01E+10	10	16	3.2	c.3.1.4
1qjd_2	58	1.684	11	1.00E+00	1.63E+10	1.684	11	1.00E+00	1.63E+10	11	14	1.5	c.3.1.4
1qo8_0	120	1.441	10	1.00E+00	6.62E+09	1.478	11	1.00E+00	7.65E+08	10	11	0.5	c.3.1.4
1qo8_2	120	2.058	10	1.00E+00	3.78E+17	2.058	10	1.00E+00	3.78E+17	10	12	1.2	c.3.1.4
1d4d_1	57	1.742	12	1.00E+00	3.01E+10	1.742	12	1.00E+00	3.01E+10	12	15	1.7	c.3.1.4
1d4e_1	58	1.522	13	1.00E+00	1.80E+07	1.522	13	1.00E+00	1.80E+07	13	15	0.9	c.3.1.4
1d4c_0	52	1.040	11	1.00E+00	1.70E+01	1.322	12	1.00E+00	5.44E+03	11	15	1.8	c.3.1.4
1d4c_1	55	1.586	11	1.00E+00	1.23E+09	1.586	11	1.00E+00	1.23E+09	11	13	1.2	c.3.1.4
1d4c_2	51	1.703	11	1.00E+00	1.13E+11	0.667	10	1.46E-04	1.46E-04	7	15	7.9	c.3.1.4
1d4c_9	53	1.439	10	1.00E+00	5.24E+08	1.454	12	1.00E+00	1.41E+06	10	14	1.0	c.3.1.4
1jnr_0	50	1.428	11	1.00E+00	1.33E+07	1.428	11	1.00E+00	1.33E+07	11	13	1.3	c.3.1.4
1jnr_1	50	1.413	10	1.00E+00	4.90E+07	1.413	10	1.00E+00	4.90E+07	10	12	1.3	c.3.1.4
1jnz_0	52	1.426	11	1.00E+00	1.42E+07	1.426	11	1.00E+00	1.42E+07	11	13	1.3	c.3.1.4
1jnz_1	53	1.404	11	1.00E+00	8.03E+06	1.404	11	1.00E+00	8.03E+06	11	13	1.1	c.3.1.4
3grs_0	45	1.271	10	1.00E+00	8.33E+05	1.271	10	1.00E+00	8.33E+05	10	11	0.8	c.3.1.5
1dnc_0	47	1.203	11	1.00E+00	3.32E+03	1.203	11	1.00E+00	3.32E+03	11	11	0.4	c.3.1.5
1gsn_0	47	1.922	11	1.00E+00	2.11E+11	1.926	13	1.00E+00	2.17E+09	11	14	1.1	c.3.1.5
1xan_0	45	1.036	9	1.00E+00	1.55E+04	1.036	9	1.00E+00	1.55E+04	9	12	1.3	c.3.1.5
1gre_1	45	1.263	9	1.00E+00	6.52E+06	1.263	9	1.00E+00	6.52E+06	9	10	1.0	c.3.1.5
1gra_1	45	1.289	9	1.00E+00	1.21E+07	1.289	9	1.00E+00	1.21E+07	9	10	1.0	c.3.1.5
1gra_2	12	1.015	6	1.00E+00	1.67E+07	1.015	6	1.00E+00	1.67E+07	6	6	0.0	c.3.1.5
1grg_0	45	1.267	9	1.00E+00	7.18E+06	1.267	9	1.00E+00	7.18E+06	9	10	1.2	c.3.1.5
1grf_0	45	0.821	10	1.46E-01	1.58E-01	0.821	10	1.46E-01	1.58E-01	10	10	0.1	c.3.1.5
1bwc_1	45	1.235	10	1.00E+00	3.01E+05	1.235	10	1.00E+00	3.01E+05	10	11	0.8	c.3.1.5
4gr1_0	45	1.683	9	1.00E+00	1.09E+13	1.638	10	1.00E+00	2.46E+11	9	10	0.3	c.3.1.5
1grb_0	60	1.429	9	1.00E+00	1.47E+08	1.417	10	1.00E+00	4.56E+06	9	12	1.9	c.3.1.5
1k4q_2	46	1.003	9	1.00E+00	6.19E+03	1.003	9	1.00E+00	6.19E+03	9	12	1.3	c.3.1.5
1grt_0	45	1.622	9	1.00E+00	2.84E+12	1.589	10	1.00E+00	7.42E+10	9	10	0.4	c.3.1.5
5grt_0	46	1.207	10	1.00E+00	1.43E+05	1.207	10	1.00E+00	1.43E+05	10	11	0.9	c.3.1.5
3grt_0	45	5.951	9	1.00E+00	1.13E+33	5.951	9	1.00E+00	1.13E+33	9	14	2.3	c.3.1.5
2grt_0	46	1.996	9	1.00E+00	7.23E+13	2.036	10	1.00E+00	1.58E+13	9	12	1.4	c.3.1.5
4grt_0	44	1.967	9	1.00E+00	3.02E+15	1.967	9	1.00E+00	3.02E+15	9	10	1.0	c.3.1.5
1onf_0	46	1.346	11	1.00E+00	2.31E+05	1.346	11	1.00E+00	2.31E+05	11	12	0.9	c.3.1.5
1ges_0	47	0.922	9	1.00E+00	9.77E+02	0.922	9	1.00E+00	9.77E+02	9	11	1.2	c.3.1.5
1ges_1	47	4.062	9	1.00E+00	8.20E+23	4.062	9	1.00E+00	8.20E+23	9	13	2.1	c.3.1.5
1ger_0	47	0.602	10	2.05E-06	2.05E-06	0.602	10	2.05E-06	2.05E-06	10	11	0.7	c.3.1.5
1ger_1	47	4.070	9	1.00E+00	8.74E+23	4.070	9	1.00E+00	8.74E+23	9	13	2.2	c.3.1.5
1get_0	65	1.877	9	1.00E+00	2.82E+13	1.911	10	1.00E+00	4.81E+12	9	12	1.3	c.3.1.5
1get_1	65	5.541	9	1.00E+00	3.70E+30	5.541	9	1.00E+00	3.70E+30	9	14	2.4	c.3.1.5
1geu_0	68	4.808	10	1.00E+00	2.36E+30	4.808	10	1.00E+00	2.36E+30	10	15	2.2	c.3.1.5
1geu_1	67	0.976	10	1.00E+00	5.25E+02	0.976	10	1.00E+00	5.25E+02	10	11	1.0	c.3.1.5
1fec_0	50	5.578	10	1.00E+00	3.23E+32	5.578	10	1.00E+00	3.23E+32	10	13	1.3	c.3.1.5
1fec_1	50	5.575	10	1.00E+00	3.16E+32	5.575	10	1.00E+00	3.16E+32	10	13	1.3	c.3.1.5
1feb_0	50	0.755	10	1.11E-02	1.12E-02	0.755	10	1.11E-02	1.12E-02	10	11	0.5	c.3.1.5
1feb_1	48	0.906	10	1.00E+00	1.17E+01	0.926	11	2.60E-01	3.02E-01	10	12	0.4	c.3.1.5
1fea_0	49	5.533	9	1.00E+00	7.14E+33	5.533	9	1.00E+00	7.14E+33	9	14	2.3	c.3.1.5
1fea_1	50	5.599	10	1.00E+00	3.74E+32	5.599	10	1.00E+00	3.74E+32	10	13	1.2	c.3.1.5
1fea_2	49	1.439	9	1.00E+00	4.52E+08	1.439	9	1.00E+00	4.52E+08	9	10	0.5	c.3.1.5
1fea_3	50	5.545	9	1.00E+00	8.26E+33	5.545	9	1.00E+00	8.26E+33	9	14	2.4	c.3.1.5
2tpr_0	49	0.743	10	5.94E-03	5.96E-03	0.743	10	5.94E-03	5.96E-03	10	11	0.6	c.3.1.5
2tpr_1	50	5.622	10	1.00E+00	4.40E+32	5.622	10	1.00E+00	4.40E+32	10	13	1.4	c.3.1.5
1tyt_0	49	0.911	10	1.00E+00	1.53E+01	0.911	10	1.00E+00	1.53E+01	10	11	0.6	c.3.1.5
1tyt_1	50	1.581	9	1.00E+00	4.80E+10	1.581	9	1.00E+00	4.80E+10	9	9	0.2	c.3.1.5
1typ_1	65	0.555	11	8.15E-10	8.15E-10	0.555	11	8.15E-10	8.15E-10	11	11	0.1	c.3.1.5
1typ_3	48	0.590	11	3.81E-09	3.81E-09	0.594	12	1.24E-11	1.24E-11	11	12	0.2	c.3.1.5
1typ_4	21	0.469	8	3.01E-05	3.01E-05	0.469	8	3.01E-05	3.01E-05	8	8	0.2	c.3.1.5
1aog_0	49	5.593	10	1.00E+00	3.37E+32	5.593	10	1.00E+00	3.37E+32	10	13	1.3	c.3.1.5
1aog_3	49	0.529	11	4.96E-11	4.96E-11	0.529	11	4.96E-11	4.96E-11	11	11	0.5	c.3.1.5

Table 5: Results for alcohol dehydrogenase matching against FAD/NAD(P)-binding domain without amino acid property.

Site	N	RMSD	q	Pvalue	Evalue	RMSD	q	Pvalue	Evalue	CG	Mean L	Var L	SCOP
1bzl_0	49	0.481	10	1.22E-09	1.22E-09	0.481	10	1.22E-09	1.22E-09	10	11	0.5	c.3.1.5
1bzl_1	49	5.586	10	1.00E+00	3.21E+32	5.586	10	1.00E+00	3.21E+32	10	13	1.3	c.3.1.5
1gxf_0	120	1.433	11	1.00E+00	4.76E+09	1.433	11	1.00E+00	4.76E+09	11	12	1.0	c.3.1.5
1gxf_2	120	5.329	11	1.00E+00	1.47E+33	5.329	11	1.00E+00	1.47E+33	11	15	2.5	c.3.1.5
1nda_0	49	5.743	10	1.00E+00	9.57E+32	5.743	10	1.00E+00	9.57E+32	10	13	1.3	c.3.1.5
1nda_1	49	5.743	10	1.00E+00	9.57E+32	5.743	10	1.00E+00	9.57E+32	10	13	1.3	c.3.1.5
1h6v_0	120	1.307	11	1.00E+00	8.03E+07	1.307	11	1.00E+00	8.03E+07	11	12	1.0	c.3.1.5
1h6v_1	120	2.162	11	1.00E+00	3.97E+17	2.162	11	1.00E+00	3.97E+17	11	17	2.8	c.3.1.5
1h6v_2	120	1.875	11	1.00E+00	7.17E+14	1.852	12	1.00E+00	3.59E+13	11	14	1.4	c.3.1.5
1h6v_3	120	1.392	10	1.00E+00	7.96E+09	1.392	10	1.00E+00	7.96E+09	10	12	1.4	c.3.1.5
1h6v_4	120	1.341	11	1.00E+00	2.51E+08	1.341	11	1.00E+00	2.51E+08	11	12	0.7	c.3.1.5
1h6v_5	120	1.260	10	1.00E+00	1.21E+07	1.377	11	1.00E+00	1.02E+07	10	12	0.9	c.3.1.5
1trb_0	49	1.537	9	1.00E+00	5.17E+11	2.164	10	1.00E+00	1.87E+16	9	12	1.2	c.3.1.5
1cl0_0	47	1.560	9	1.00E+00	7.83E+11	2.382	10	1.00E+00	7.23E+17	9	12	1.3	c.3.1.5
1tde_0	52	0.648	10	5.35E-05	5.35E-05	0.648	10	5.35E-05	5.35E-05	10	10	0.3	c.3.1.5
1tdf_0	52	1.122	10	1.00E+00	3.64E+05	1.122	10	1.00E+00	3.64E+05	10	10	0.1	c.3.1.5
1tdf_1	26	0.722	11	2.76E-06	2.76E-06	0.722	11	2.76E-06	2.76E-06	11	12	0.8	c.3.1.5
1f6m_0	62	0.771	10	4.44E-02	4.54E-02	0.771	10	4.44E-02	4.54E-02	10	10	0.1	c.3.1.5
1f6m_1	63	1.306	11	1.00E+00	7.34E+05	1.306	11	1.00E+00	7.34E+05	11	14	1.7	c.3.1.5
1f6m_2	62	0.776	10	5.54E-02	5.70E-02	0.776	10	5.54E-02	5.70E-02	10	10	0.1	c.3.1.5
1f6m_3	63	1.304	11	1.00E+00	6.90E+05	1.304	11	1.00E+00	6.90E+05	11	14	1.6	c.3.1.5
1vdc_0	95	1.447	10	1.00E+00	3.81E+09	1.447	10	1.00E+00	3.81E+09	10	12	1.4	c.3.1.5
1m6i_0	43	2.951	10	1.00E+00	2.56E+21	2.951	10	1.00E+00	2.56E+21	10	12	1.3	c.3.1.5
1gv4_0	109	1.447	11	1.00E+00	2.43E+08	1.447	11	1.00E+00	2.43E+08	11	13	2.5	c.3.1.5
1hyu_0	44	2.112	9	1.00E+00	3.96E+14	2.112	9	1.00E+00	3.96E+14	9	12	1.3	c.3.1.5
1fl2_5	43	4.126	10	1.00E+00	1.40E+27	4.126	10	1.00E+00	1.40E+27	10	13	1.3	c.3.1.5
1nhq_0	37	0.990	10	1.00E+00	6.54E+01	0.990	10	1.00E+00	6.54E+01	10	12	2.1	c.3.1.5
1nhp_0	37	1.002	10	1.00E+00	1.00E+02	1.002	10	1.00E+00	1.00E+02	10	12	2.2	c.3.1.5
1nhr_0	37	1.140	10	1.00E+00	2.85E+04	1.140	10	1.00E+00	2.85E+04	10	12	2.3	c.3.1.5
1nhs_0	39	0.976	10	1.00E+00	4.64E+01	0.976	10	1.00E+00	4.64E+01	10	12	2.4	c.3.1.5
1npx_0	40	0.990	10	1.00E+00	8.31E+01	0.990	10	1.00E+00	8.31E+01	10	12	2.4	c.3.1.5
1f8w_0	39	1.095	10	1.00E+00	7.42E+03	1.095	10	1.00E+00	7.42E+03	10	12	1.9	c.3.1.5
1joa_0	57	0.703	11	6.77E-06	6.77E-06	0.703	11	6.77E-06	6.77E-06	11	13	1.1	c.3.1.5
2npx_0	59	0.383	12	0.00E+00	1.64E-19	0.383	12	0.00E+00	1.64E-19	12	14	1.1	c.3.1.5
1d7y_0	42	2.254	9	1.00E+00	3.76E+17	2.254	9	1.00E+00	3.76E+17	9	10	1.1	c.3.1.5
1f3p_0	63	0.523	11	4.18E-11	4.18E-11	0.523	12	8.93E-14	8.92E-14	11	13	0.7	c.3.1.5
1q1r_0	41	2.263	10	1.00E+00	6.30E+16	2.263	10	1.00E+00	6.30E+16	10	11	0.9	c.3.1.5
1q1r_1	42	2.247	10	1.00E+00	5.13E+16	2.247	10	1.00E+00	5.13E+16	10	11	0.9	c.3.1.5
1q1w_0	41	3.234	10	1.00E+00	1.94E+24	3.234	10	1.00E+00	1.94E+24	10	12	1.1	c.3.1.5
1q1w_1	42	2.060	9	1.00E+00	1.47E+15	2.060	9	1.00E+00	1.47E+15	9	10	0.9	c.3.1.5
1lvl_0	66	1.051	13	2.01E-01	2.25E-01	1.009	14	9.79E-04	9.79E-04	13	14	0.5	c.3.1.5
1lpf_0	47	0.676	11	8.65E-07	8.65E-07	0.676	11	8.65E-07	8.65E-07	11	12	0.8	c.3.1.5
1lpf_1	47	0.840	11	5.52E-03	5.54E-03	0.840	11	5.52E-03	5.54E-03	11	11	0.0	c.3.1.5
3lad_0	48	0.742	12	2.93E-07	2.93E-07	0.742	12	2.93E-07	2.93E-07	12	13	0.5	c.3.1.5
3lad_1	46	0.630	11	4.71E-08	4.71E-08	0.630	11	4.71E-08	4.71E-08	11	12	0.7	c.3.1.5
1ebd_0	46	0.786	12	2.50E-06	2.50E-06	0.786	12	2.50E-06	2.50E-06	12	12	0.0	c.3.1.5
1ebd_1	49	0.815	12	1.45E-05	1.45E-05	0.815	12	1.45E-05	1.45E-05	12	12	0.0	c.3.1.5
1ojt_0	48	0.804	10	8.75E-02	9.16E-02	0.823	11	2.58E-03	2.59E-03	10	11	0.4	c.3.1.5
1bhy_0	47	0.821	11	2.20E-03	2.20E-03	0.821	11	2.20E-03	2.20E-03	11	11	0.2	c.3.1.5
1jeh_0	48	0.717	11	9.93E-06	9.93E-06	0.717	11	9.93E-06	9.93E-06	11	12	0.6	c.3.1.5
1jeh_1	49	0.726	11	1.75E-05	1.75E-05	0.802	12	1.06E-05	1.06E-05	11	12	0.7	c.3.1.5
1dxl_0	90	1.086	10	1.00E+00	2.62E+04	1.086	10	1.00E+00	2.62E+04	10	13	1.6	c.3.1.5
1dxl_1	86	0.920	11	5.11E-01	7.16E-01	1.020	12	4.32E-01	5.65E-01	11	13	0.6	c.3.1.5
1mo9_0	44	1.247	9	1.00E+00	4.13E+06	1.247	9	1.00E+00	4.13E+06	9	11	1.2	c.3.1.5
1mo9_1	46	1.381	10	1.00E+00	3.15E+08	1.500	12	1.00E+00	1.79E+07	10	12	0.7	c.3.1.5
1mok_0	47	1.704	10	1.00E+00	2.03E+11	1.704	10	1.00E+00	2.03E+11	10	11	0.7	c.3.1.5
1mok_1	46	1.461	10	1.00E+00	5.99E+08	1.466	11	1.00E+00	2.97E+07	10	13	1.8	c.3.1.5
1mok_2	46	1.696	10	1.00E+00	1.04E+12	1.697	11	1.00E+00	7.12E+10	10	12	0.6	c.3.1.5
1mok_3	45	1.707	10	1.00E+00	1.89E+11	1.707	10	1.00E+00	1.89E+11	10	11	0.6	c.3.1.5
1fcd_2	48	0.811	13	1.27E-07	1.27E-07	0.811	13	1.27E-07	1.27E-07	13	14	0.2	c.3.1.5
1fcd_3	46	0.685	11	1.47E-06	1.47E-06	0.685	11	1.47E-06	1.47E-06	11	12	0.4	c.3.1.5
1w4x_0	120	0.662	11	6.39E-06	6.39E-06	0.662	11	6.39E-06	6.39E-06	11	12	0.5	c.3.1.5

Table 6: Results for alcohol dehydrogenase matching against FAD/NAD(P)-binding domain with amino acid property.

Site	N	RMSD	q	Pvalue	Evalue	RMSD	q	Pvalue	Evalue	CG	Mean L	Var L	SCOP
1ps9_0	69	0.592	9	1.03E-05	1.03E-05	0.592	9	1.03E-05	1.03E-05	9	9	0.2	c.3.1.1
1cjc_0	42	1.424	6	1.00E+00	6.10E+09	1.424	6	1.00E+00	6.10E+09	6	7	0.7	c.3.1.1
1e1m_0	120	3.912	7	1.00E+00	7.80E+21	3.912	7	1.00E+00	7.80E+21	7	11	2.3	c.3.1.1
1e6e_0	120	1.808	7	1.00E+00	1.66E+14	1.885	9	1.00E+00	4.56E+12	7	13	2.2	c.3.1.1
1e1k_0	120	1.564	7	1.00E+00	1.51E+12	1.564	7	1.00E+00	1.51E+12	7	8	2.5	c.3.1.1
1e1n_0	92	8.920	7	1.00E+00	3.00E+33	8.920	7	1.00E+00	3.00E+33	7	12	2.5	c.3.1.1
1e1l_0	120	2.241	7	1.00E+00	7.74E+16	2.189	8	1.00E+00	5.59E+15	7	10	1.1	c.3.1.1
1lqt_0	82	2.005	7	1.00E+00	1.90E+14	2.031	8	1.00E+00	3.11E+13	7	9	0.8	c.3.1.1
1lqt_2	82	5.609	7	1.00E+00	1.71E+23	5.609	7	1.00E+00	1.71E+23	7	10	1.7	c.3.1.1
1lqu_0	81	6.624	7	1.00E+00	1.52E+28	6.624	7	1.00E+00	1.52E+28	7	11	1.9	c.3.1.1
1lqu_2	81	6.489	7	1.00E+00	8.79E+27	6.489	7	1.00E+00	8.79E+27	7	11	1.9	c.3.1.1
1h7w_0	120	5.706	7	1.00E+00	1.07E+28	5.706	7	1.00E+00	1.07E+28	7	11	1.9	c.3.1.1
1gte_0	120	6.875	7	1.00E+00	4.42E+28	6.875	7	1.00E+00	4.42E+28	7	10	1.6	c.3.1.1
1gte_1	120	1.457	8	1.00E+00	1.08E+10	1.521	9	1.00E+00	2.23E+09	8	9	0.3	c.3.1.1
1gte_5	120	1.287	7	1.00E+00	3.23E+09	1.287	7	1.00E+00	3.23E+09	7	9	1.4	c.3.1.1
1gte_6	120	1.461	8	1.00E+00	1.18E+10	1.461	8	1.00E+00	1.18E+10	8	9	0.3	c.3.1.1
1h7x_0	120	5.722	7	1.00E+00	1.16E+28	5.722	7	1.00E+00	1.16E+28	7	11	1.7	c.3.1.1
1h7x_3	67	1.048	7	1.00E+00	3.50E+06	1.048	7	1.00E+00	3.50E+06	7	9	1.7	c.3.1.1
1h7x_5	67	1.055	7	1.00E+00	4.12E+06	1.055	7	1.00E+00	4.12E+06	7	9	1.7	c.3.1.1
1h7x_8	65	1.058	7	1.00E+00	4.03E+06	1.058	7	1.00E+00	4.03E+06	7	9	2.0	c.3.1.1
1h7x_10	66	1.058	7	1.00E+00	4.22E+06	1.058	7	1.00E+00	4.22E+06	7	9	2.2	c.3.1.1
1gth_0	120	6.890	7	1.00E+00	4.68E+28	6.890	7	1.00E+00	4.68E+28	7	10	1.5	c.3.1.1
1gth_1	120	1.677	8	1.00E+00	1.21E+12	1.677	8	1.00E+00	1.21E+12	8	9	1.0	c.3.1.1
1gth_3	64	3.237	8	1.00E+00	1.73E+19	3.237	8	1.00E+00	1.73E+19	8	10	0.9	c.3.1.1
1gth_6	64	3.232	8	1.00E+00	1.65E+19	3.232	8	1.00E+00	1.65E+19	8	10	0.9	c.3.1.1
1gth_7	120	6.898	7	1.00E+00	4.83E+28	6.898	7	1.00E+00	4.83E+28	7	10	1.9	c.3.1.1
1gth_8	120	1.675	8	1.00E+00	1.16E+12	1.675	8	1.00E+00	1.16E+12	8	9	1.2	c.3.1.1
1gth_10	65	3.231	8	1.00E+00	1.72E+19	3.231	8	1.00E+00	1.72E+19	8	10	1.0	c.3.1.1
1gth_13	64	1.600	8	1.00E+00	4.19E+10	1.722	11	1.00E+00	1.47E+08	8	11	0.8	c.3.1.1
1gt8_0	120	4.670	7	1.00E+00	7.84E+24	4.670	7	1.00E+00	7.84E+24	7	9	1.6	c.3.1.1
1gt8_1	120	1.626	7	1.00E+00	2.61E+12	1.626	7	1.00E+00	2.61E+12	7	9	1.6	c.3.1.1
1gt8_3	65	1.550	7	1.00E+00	8.46E+10	1.589	8	1.00E+00	8.64E+09	7	9	0.9	c.3.1.1
1gt8_6	66	1.546	7	1.00E+00	8.32E+10	1.588	8	1.00E+00	8.89E+09	7	9	1.0	c.3.1.1
1gt8_7	120	6.797	8	1.00E+00	5.22E+32	6.797	8	1.00E+00	5.22E+32	8	11	1.7	c.3.1.1
1gt8_8	120	1.608	7	1.00E+00	1.89E+12	1.608	7	1.00E+00	1.89E+12	7	9	1.3	c.3.1.1
1gt8_10	66	2.106	7	1.00E+00	1.68E+14	2.171	9	1.00E+00	6.79E+12	7	11	2.0	c.3.1.1
1gt8_13	65	3.452	7	1.00E+00	2.83E+21	3.452	7	1.00E+00	2.83E+21	7	11	1.3	c.3.1.1
3cox_0	45	3.635	7	1.00E+00	6.77E+18	3.635	7	1.00E+00	6.77E+18	7	13	1.8	c.3.1.2
1coy_0	61	3.059	7	1.00E+00	7.58E+16	3.152	8	1.00E+00	6.14E+16	7	11	1.4	c.3.1.2
1n4w_1	54	4.302	7	1.00E+00	1.79E+17	4.302	7	1.00E+00	1.79E+17	7	10	1.5	c.3.1.2
1n1p_3	54	4.301	7	1.00E+00	1.78E+17	4.301	7	1.00E+00	1.78E+17	7	10	1.7	c.3.1.2
1n4v_0	55	4.313	7	1.00E+00	1.98E+17	4.313	7	1.00E+00	1.98E+17	7	10	1.7	c.3.1.2
1ijh_0	47	4.295	7	1.00E+00	1.13E+17	4.295	7	1.00E+00	1.13E+17	7	10	1.5	c.3.1.2
1b4v_0	72	1.513	7	1.00E+00	6.37E+10	1.658	8	1.00E+00	3.80E+10	7	12	2.1	c.3.1.2
1b8s_0	68	1.501	7	1.00E+00	4.40E+10	1.761	9	1.00E+00	1.26E+10	7	12	2.1	c.3.1.2
1cbo_0	71	1.219	7	1.00E+00	6.97E+07	1.219	7	1.00E+00	6.97E+07	7	10	2.2	c.3.1.2
1cc2_0	71	1.236	7	1.00E+00	9.28E+07	1.236	7	1.00E+00	9.28E+07	7	11	2.7	c.3.1.2
1cf3_4	47	1.817	7	1.00E+00	1.48E+11	1.922	8	1.00E+00	5.12E+10	7	10	1.3	c.3.1.2
1gal_0	48	2.189	7	1.00E+00	9.55E+13	2.244	8	1.00E+00	2.68E+13	7	8	1.0	c.3.1.2
1gpe_8	50	1.572	6	1.00E+00	7.23E+10	1.771	8	1.00E+00	4.38E+09	6	10	1.1	c.3.1.2
1gpe_9	50	4.781	7	1.00E+00	7.98E+21	4.781	7	1.00E+00	7.98E+21	7	12	1.9	c.3.1.2
1ju2_8	48	1.696	7	1.00E+00	1.01E+11	1.696	7	1.00E+00	1.01E+11	7	10	2.3	c.3.1.2
1ju2_9	48	1.695	7	1.00E+00	9.94E+10	1.695	7	1.00E+00	9.94E+10	7	10	2.3	c.3.1.2
1pbe_0	51	2.688	7	1.00E+00	4.52E+16	2.962	12	1.00E+00	2.85E+15	7	12	1.4	c.3.1.2
1bgn_0	51	1.152	7	1.00E+00	1.25E+07	1.124	9	1.00E+00	2.62E+03	7	11	1.2	c.3.1.2
1bkw_0	51	1.975	6	1.00E+00	2.86E+13	2.142	8	1.00E+00	2.93E+12	6	11	1.9	c.3.1.2
1pbd_0	50	1.979	6	1.00E+00	2.81E+13	2.039	7	1.00E+00	6.18E+12	6	10	2.1	c.3.1.2
1cc6_0	82	1.137	7	1.00E+00	6.56E+07	0.407	7	2.83E-03	2.83E-03	4	14	3.5	c.3.1.2
1cj4_0	83	1.125	7	1.00E+00	5.13E+07	1.108	9	1.00E+00	1.27E+04	7	11	1.4	c.3.1.2
1bf3_0	51	1.143	7	1.00E+00	1.02E+07	1.117	9	1.00E+00	2.14E+03	7	11	1.3	c.3.1.2
1cj3_0	82	2.432	7	1.00E+00	4.07E+16	2.432	7	1.00E+00	4.07E+16	7	8	0.7	c.3.1.2
1pbb_0	48	2.889	7	1.00E+00	1.48E+17	0.821	10	6.42E-04	6.42E-04	4	13	2.6	c.3.1.2
1pbc_0	48	2.887	7	1.00E+00	1.45E+17	2.887	7	1.00E+00	1.45E+17	7	9	3.3	c.3.1.2
1pbf_0	46	2.987	6	1.00E+00	1.67E+17	2.987	6	1.00E+00	1.67E+17	6	10	4.0	c.3.1.2
1cj2_0	81	2.832	7	1.00E+00	7.51E+17	2.832	7	1.00E+00	7.51E+17	7	9	1.2	c.3.1.2
1cc4_0	83	1.142	7	1.00E+00	7.65E+07	1.117	9	1.00E+00	1.65E+04	7	11	1.4	c.3.1.2
1bgj_0	51	2.471	6	1.00E+00	6.46E+15	2.471	6	1.00E+00	6.46E+15	6	8	0.9	c.3.1.2
1phh_0	52	1.303	7	1.00E+00	1.14E+08	1.303	7	1.00E+00	1.14E+08	7	8	0.7	c.3.1.2
1k0i_0	43	2.896	6	1.00E+00	1.85E+18	2.823	7	1.00E+00	3.01E+17	6	8	1.0	c.3.1.2

Table 7: Results for alcohol dehydrogenase matching against FAD/NAD(P)-binding domain with amino acid property.

Site	N	RMSD	q	Pvalue	Evalue	RMSD	q	Pvalue	Evalue	CG	Mean L	Var L	SCOP
1iut_0	49	1.126	7	1.00E+00	6.03E+06	0.875	10	6.51E-03	6.53E-03	5	13	3.0	c.3.1.2
1iux_0	50	1.156	7	1.00E+00	1.29E+07	1.086	10	1.00E+00	1.82E+01	7	11	1.2	c.3.1.2
1iuw_0	50	1.144	7	1.00E+00	9.79E+06	1.119	9	1.00E+00	2.13E+03	7	11	1.3	c.3.1.2
1doc_0	53	1.147	7	1.00E+00	1.25E+07	1.122	9	1.00E+00	2.78E+03	7	11	1.3	c.3.1.2
1iuu_0	50	1.135	7	1.00E+00	7.93E+06	1.112	9	1.00E+00	1.74E+03	7	11	1.2	c.3.1.2
1k0l_0	39	2.923	6	1.00E+00	1.71E+18	2.849	7	1.00E+00	2.84E+17	6	8	1.0	c.3.1.2
1dod_0	48	1.996	6	1.00E+00	2.99E+13	0.805	10	3.20E-04	3.20E-04	4	13	2.5	c.3.1.2
1pxc_0	49	1.123	7	1.00E+00	5.62E+06	1.098	9	1.00E+00	1.08E+03	7	11	1.2	c.3.1.2
1dob_0	50	1.124	7	1.00E+00	6.12E+06	1.164	11	9.96E-01	5.42E+00	7	11	0.9	c.3.1.2
1ius_0	50	1.091	7	1.00E+00	2.77E+06	0.879	10	8.14E-03	8.17E-03	5	13	3.9	c.3.1.2
1doe_0	52	1.936	6	1.00E+00	1.97E+13	2.003	7	1.00E+00	4.49E+12	6	10	2.2	c.3.1.2
1pxb_0	49	2.708	7	1.00E+00	1.92E+16	2.562	9	1.00E+00	2.47E+14	7	11	1.3	c.3.1.2
1pxa_0	49	1.218	7	1.00E+00	4.88E+07	1.170	10	1.00E+00	2.78E+02	7	11	1.0	c.3.1.2
1iuv_0	50	1.974	6	1.00E+00	2.66E+13	2.037	7	1.00E+00	6.03E+12	6	10	2.4	c.3.1.2
1k0j_0	49	3.240	6	1.00E+00	3.57E+18	3.240	6	1.00E+00	3.57E+18	6	10	1.6	c.3.1.2
1el5_0	50	1.747	7	1.00E+00	2.48E+12	1.747	7	1.00E+00	2.48E+12	7	8	1.0	c.3.1.2
1el5_1	51	1.714	7	1.00E+00	1.53E+12	1.714	7	1.00E+00	1.53E+12	7	8	1.0	c.3.1.2
1l9e_0	48	0.918	7	1.00E+00	5.39E+04	0.918	7	1.00E+00	5.39E+04	7	8	0.8	c.3.1.2
1l9e_1	49	0.916	7	1.00E+00	5.47E+04	0.916	7	1.00E+00	5.47E+04	7	8	0.9	c.3.1.2
1l9f_0	44	0.916	7	1.00E+00	3.93E+04	0.916	7	1.00E+00	3.93E+04	7	9	1.1	c.3.1.2
1l9f_1	45	0.915	7	1.00E+00	4.11E+04	0.915	7	1.00E+00	4.11E+04	7	8	1.0	c.3.1.2
1l9c_0	45	0.883	7	1.00E+00	8.73E+03	0.932	8	1.00E+00	3.83E+02	7	9	0.7	c.3.1.2
1l9c_1	44	3.699	6	1.00E+00	2.60E+19	3.699	6	1.00E+00	2.60E+19	6	9	1.5	c.3.1.2
1el7_0	50	2.733	6	1.00E+00	2.41E+17	2.715	7	1.00E+00	5.49E+16	6	9	0.8	c.3.1.2
1el7_1	51	2.729	6	1.00E+00	2.48E+17	2.712	7	1.00E+00	5.66E+16	6	9	0.8	c.3.1.2
1l9d_0	50	1.776	7	1.00E+00	3.97E+12	1.776	7	1.00E+00	3.97E+12	7	9	1.0	c.3.1.2
1l9d_1	51	1.734	7	1.00E+00	2.13E+12	1.734	7	1.00E+00	2.13E+12	7	9	1.1	c.3.1.2
1el8_0	50	1.776	7	1.00E+00	3.97E+12	1.776	7	1.00E+00	3.97E+12	7	8	1.0	c.3.1.2
1el8_1	51	1.735	7	1.00E+00	2.16E+12	1.735	7	1.00E+00	2.16E+12	7	8	1.0	c.3.1.2
1eli_0	73	6.592	7	1.00E+00	9.64E+27	6.592	7	1.00E+00	9.64E+27	7	10	1.6	c.3.1.2
1eli_3	71	1.859	6	1.00E+00	6.44E+13	1.840	7	1.00E+00	4.38E+12	6	8	1.1	c.3.1.2
1el9_0	73	6.319	7	1.00E+00	4.35E+25	6.319	7	1.00E+00	4.35E+25	7	11	1.9	c.3.1.2
1el9_3	72	1.993	7	1.00E+00	1.35E+14	2.067	8	1.00E+00	4.27E+13	7	9	0.8	c.3.1.2
1ng4_0	52	2.783	7	1.00E+00	2.11E+17	2.752	8	1.00E+00	4.33E+16	7	13	1.6	c.3.1.2
1ng4_1	51	2.123	7	1.00E+00	3.10E+13	2.123	7	1.00E+00	3.10E+13	7	12	1.9	c.3.1.2
1ng3_0	53	2.994	7	1.00E+00	8.97E+17	2.903	9	1.00E+00	4.13E+16	7	12	1.7	c.3.1.2
1ng3_1	55	2.110	7	1.00E+00	3.36E+13	2.110	7	1.00E+00	3.36E+13	7	12	1.9	c.3.1.2
1pn0_0	53	8.109	7	1.00E+00	5.22E+29	8.109	7	1.00E+00	5.22E+29	7	10	1.7	c.3.1.2
1pn0_1	53	2.247	7	1.00E+00	8.00E+13	2.507	8	1.00E+00	2.01E+14	7	10	1.3	c.3.1.2
1pn0_6	39	4.329	6	1.00E+00	1.56E+19	4.329	6	1.00E+00	1.56E+19	6	9	1.3	c.3.1.2
1pn0_8	39	3.086	6	1.00E+00	1.06E+18	3.086	6	1.00E+00	1.06E+18	6	10	1.0	c.3.1.2
1foh_0	53	2.136	7	1.00E+00	1.93E+14	2.186	9	1.00E+00	7.08E+12	7	10	0.8	c.3.1.2
1foh_1	54	2.136	7	1.00E+00	2.04E+14	2.189	9	1.00E+00	7.84E+12	7	10	0.8	c.3.1.2
1foh_2	39	6.696	6	1.00E+00	4.49E+23	6.696	6	1.00E+00	4.49E+23	6	9	1.6	c.3.1.2
1foh_4	39	3.061	6	1.00E+00	8.91E+17	3.061	6	1.00E+00	8.91E+17	6	10	1.0	c.3.1.2
1h82_0	120	1.539	8	1.00E+00	3.32E+10	1.581	9	1.00E+00	4.02E+09	8	11	1.6	c.3.1.2
1h82_1	62	0.559	7	7.96E-01	1.59E+00	0.559	7	7.96E-01	1.59E+00	7	8	0.6	c.3.1.2
1h82_2	62	0.506	7	1.54E-01	1.67E-01	0.506	7	1.54E-01	1.67E-01	7	8	0.7	c.3.1.2
1h83_0	120	1.524	8	1.00E+00	2.49E+10	1.620	10	1.00E+00	5.19E+08	8	12	1.5	c.3.1.2
1h83_1	57	1.251	7	1.00E+00	9.59E+07	1.351	8	1.00E+00	2.19E+07	7	9	1.7	c.3.1.2
1h83_2	56	1.272	7	1.00E+00	1.37E+08	1.356	8	1.00E+00	2.30E+07	7	9	1.7	c.3.1.2
1b5q_0	53	2.166	7	1.00E+00	6.55E+12	2.262	9	1.00E+00	4.24E+11	7	10	1.2	c.3.1.2
1b5q_1	53	1.571	7	1.00E+00	3.66E+10	1.558	8	1.00E+00	1.56E+09	7	10	1.7	c.3.1.2
1b5q_2	55	1.756	7	1.00E+00	3.60E+11	1.727	9	1.00E+00	1.19E+09	7	9	0.3	c.3.1.2
1b37_3	45	1.259	7	1.00E+00	5.44E+07	1.355	8	1.00E+00	1.15E+07	7	9	1.7	c.3.1.2
1b37_4	46	1.073	7	1.00E+00	9.46E+05	1.073	7	1.00E+00	9.46E+05	7	9	1.4	c.3.1.2
1b37_5	46	1.596	7	1.00E+00	3.50E+10	1.573	8	1.00E+00	1.31E+09	7	10	1.6	c.3.1.2
1h86_0	120	2.344	8	1.00E+00	1.45E+16	2.291	9	1.00E+00	1.19E+15	8	11	1.5	c.3.1.2
1h86_1	52	1.561	7	1.00E+00	2.95E+10	1.554	8	1.00E+00	1.37E+09	7	10	1.4	c.3.1.2
1h86_2	52	1.065	7	1.00E+00	1.16E+06	1.065	7	1.00E+00	1.16E+06	7	9	1.4	c.3.1.2
1h84_0	120	1.168	7	1.00E+00	2.04E+08	1.168	7	1.00E+00	2.04E+08	7	9	0.8	c.3.1.2
1h84_1	52	1.609	7	1.00E+00	6.22E+10	1.593	8	1.00E+00	2.71E+09	7	10	1.5	c.3.1.2
1h84_2	54	1.640	7	1.00E+00	1.12E+11	1.614	8	1.00E+00	4.37E+09	7	10	1.6	c.3.1.2
1h81_0	120	6.679	8	1.00E+00	2.96E+32	6.679	8	1.00E+00	2.96E+32	8	13	2.2	c.3.1.2
1h81_1	120	1.147	7	1.00E+00	2.63E+08	1.291	8	1.00E+00	1.84E+08	7	8	0.6	c.3.1.2
1f8r_4	52	1.090	8	1.00E+00	6.23E+04	1.090	8	1.00E+00	6.23E+04	8	9	0.6	c.3.1.2
1f8r_5	53	1.080	8	1.00E+00	5.21E+04	1.080	8	1.00E+00	5.21E+04	8	9	0.6	c.3.1.2
1f8r_6	52	1.089	8	1.00E+00	6.08E+04	1.089	8	1.00E+00	6.08E+04	8	9	0.6	c.3.1.2
1f8r_7	52	1.086	8	1.00E+00	5.67E+04	1.086	8	1.00E+00	5.67E+04	8	9	0.7	c.3.1.2

Table 8: Results for alcohol dehydrogenase matching against FAD/NAD(P)-binding domain with amino acid property.

Site	N	RMSD	q	Pvalue	Evalue	RMSD	q	Pvalue	Evalue	CG	Mean L	Var L	SCOP
1f8s_0	59	1.054	7	1.00E+00	1.35E+06	1.054	7	1.00E+00	1.35E+06	7	9	1.0	c.3.1.2
1f8s_1	59	1.058	7	1.00E+00	1.47E+06	1.058	7	1.00E+00	1.47E+06	7	9	0.9	c.3.1.2
1f8s_2	59	7.459	7	1.00E+00	2.31E+27	7.459	7	1.00E+00	2.31E+27	7	10	1.7	c.3.1.2
1f8s_3	59	1.082	8	1.00E+00	7.59E+04	1.082	8	1.00E+00	7.59E+04	8	9	0.6	c.3.1.2
1f8s_4	59	1.084	8	1.00E+00	7.95E+04	1.084	8	1.00E+00	7.95E+04	8	9	0.6	c.3.1.2
1f8s_5	58	1.053	7	1.00E+00	1.26E+06	1.053	7	1.00E+00	1.26E+06	7	9	1.0	c.3.1.2
1f8s_6	59	1.054	7	1.00E+00	1.35E+06	1.054	7	1.00E+00	1.35E+06	7	9	1.0	c.3.1.2
1f8s_7	59	1.053	7	1.00E+00	1.32E+06	1.053	7	1.00E+00	1.32E+06	7	9	1.0	c.3.1.2
1reo_0	49	2.659	7	1.00E+00	7.02E+15	2.620	8	1.00E+00	1.17E+15	7	8	0.6	c.3.1.2
1tdn_0	49	1.291	8	1.00E+00	3.99E+06	1.291	8	1.00E+00	3.99E+06	8	9	0.7	c.3.1.2
1tdk_0	53	1.249	8	1.00E+00	3.35E+06	1.255	9	1.00E+00	1.07E+05	8	10	0.4	c.3.1.2
1tdo_0	51	1.295	8	1.00E+00	4.91E+06	0.431	7	2.43E-03	2.43E-03	6	10	5.1	c.3.1.2
1oja_0	120	0.670	8	7.73E-01	1.48E+00	0.670	8	7.73E-01	1.48E+00	8	9	0.4	c.3.1.2
1oja_1	57	0.996	8	1.00E+00	2.98E+03	0.996	9	1.00E+00	4.19E+01	8	9	0.1	c.3.1.2
1ojb_0	120	0.662	8	6.55E-01	1.06E+00	0.662	8	6.55E-01	1.06E+00	8	9	0.3	c.3.1.2
1ojb_1	116	0.443	7	5.36E-02	5.51E-02	0.443	7	5.36E-02	5.51E-02	7	8	0.4	c.3.1.2
1oj9_0	120	1.046	9	1.00E+00	3.45E+03	1.046	9	1.00E+00	3.45E+03	9	9	0.3	c.3.1.2
1ojc_0	120	0.673	8	8.13E-01	1.68E+00	0.673	8	8.13E-01	1.68E+00	8	9	0.4	c.3.1.2
1ojc_1	54	0.452	7	8.47E-03	8.51E-03	0.452	7	8.47E-03	8.51E-03	7	8	0.4	c.3.1.2
1gos_0	120	3.637	8	1.00E+00	4.52E+20	3.500	10	1.00E+00	4.92E+19	8	15	2.6	c.3.1.2
1gos_1	120	5.134	9	1.00E+00	2.89E+26	5.134	9	1.00E+00	2.89E+26	9	14	2.8	c.3.1.2
1ojd_0	120	1.067	9	1.00E+00	6.58E+03	1.067	9	1.00E+00	6.58E+03	9	9	0.3	c.3.1.2
1ojd_1	120	0.662	8	6.55E-01	1.06E+00	0.662	8	6.55E-01	1.06E+00	8	9	0.3	c.3.1.2
1ojd_2	120	0.667	8	7.30E-01	1.31E+00	0.667	8	7.30E-01	1.31E+00	8	9	0.5	c.3.1.2
1ojd_3	120	0.662	8	6.55E-01	1.06E+00	0.662	8	6.55E-01	1.06E+00	8	9	0.2	c.3.1.2
1ojd_4	120	0.662	8	6.55E-01	1.06E+00	0.662	8	6.55E-01	1.06E+00	8	9	0.3	c.3.1.2
1ojd_5	120	1.064	9	1.00E+00	6.01E+03	1.064	9	1.00E+00	6.01E+03	9	9	0.3	c.3.1.2
1ojd_6	120	1.071	9	1.00E+00	7.43E+03	1.071	9	1.00E+00	7.43E+03	9	9	0.3	c.3.1.2
1ojd_7	120	0.655	8	5.48E-01	7.94E-01	0.655	8	5.48E-01	7.94E-01	8	9	0.3	c.3.1.2
1ojd_8	120	1.063	9	1.00E+00	5.83E+03	1.063	9	1.00E+00	5.83E+03	9	9	0.3	c.3.1.2
1ojd_9	120	0.657	8	5.78E-01	8.64E-01	0.657	8	5.78E-01	8.64E-01	8	9	0.3	c.3.1.2
1o5w_0	64	1.153	7	1.00E+00	2.59E+07	1.131	9	1.00E+00	6.48E+03	7	9	0.4	c.3.1.2
1o5w_1	62	1.155	7	1.00E+00	2.46E+07	1.133	9	1.00E+00	6.23E+03	7	9	0.4	c.3.1.2
1o5w_2	63	2.254	7	1.00E+00	8.33E+13	2.254	7	1.00E+00	8.33E+13	7	10	1.0	c.3.1.2
1o5w_3	64	1.157	7	1.00E+00	2.84E+07	1.136	9	1.00E+00	7.48E+03	7	9	0.3	c.3.1.2
1pj5_0	49	1.604	6	1.00E+00	2.26E+12	1.604	6	1.00E+00	2.26E+12	6	8	1.3	c.3.1.2
1pj6_1	45	0.976	7	1.00E+00	1.80E+05	0.812	11	3.35E-06	3.35E-06	6	11	0.7	c.3.1.2
1pj7_2	46	1.704	6	1.00E+00	7.91E+12	1.704	6	1.00E+00	7.91E+12	6	9	1.2	c.3.1.2
1sez_0	92	2.731	7	1.00E+00	9.23E+16	2.731	7	1.00E+00	9.23E+16	7	10	2.1	c.3.1.2
1sez_3	80	1.736	7	1.00E+00	1.51E+13	1.785	8	1.00E+00	2.61E+12	7	10	1.2	c.3.1.2
1knr_0	52	2.515	6	1.00E+00	2.20E+16	2.515	6	1.00E+00	2.20E+16	6	9	1.4	c.3.1.4
1knp_0	59	2.995	7	1.00E+00	7.27E+17	2.995	7	1.00E+00	7.27E+17	7	10	1.9	c.3.1.4
1nek_0	56	1.468	8	1.00E+00	8.03E+08	1.493	10	1.00E+00	2.81E+06	8	11	1.2	c.3.1.4
1nen_0	57	1.584	7	1.00E+00	5.54E+10	1.604	9	1.00E+00	2.44E+08	7	11	1.5	c.3.1.4
1kf6_0	56	1.239	9	1.00E+00	8.82E+04	0.575	9	3.02E-05	3.02E-05	8	12	7.8	c.3.1.4
1kf6_1	56	1.290	9	1.00E+00	1.77E+05	1.341	10	1.00E+00	1.94E+04	9	11	0.7	c.3.1.4
1l0v_0	56	1.214	8	1.00E+00	1.08E+06	0.649	9	9.50E-04	9.50E-04	5	13	8.0	c.3.1.4
1l0v_1	50	0.914	7	1.00E+00	5.60E+04	0.908	8	1.00E+00	5.18E+02	7	8	0.1	c.3.1.4
1kfy_0	55	2.348	10	1.00E+00	2.46E+13	2.349	12	1.00E+00	7.54E+11	10	13	1.4	c.3.1.4
1kfy_1	54	1.178	9	1.00E+00	1.71E+04	1.178	9	1.00E+00	1.71E+04	9	10	0.7	c.3.1.4
1qla_5	50	1.618	6	1.00E+00	9.81E+11	1.618	6	1.00E+00	9.81E+11	6	6	0.2	c.3.1.4
1qla_7	50	1.616	6	1.00E+00	9.53E+11	1.616	6	1.00E+00	9.53E+11	6	6	0.3	c.3.1.4
1qlb_2	53	0.976	7	1.00E+00	2.93E+05	0.970	9	1.00E+00	4.28E+01	7	9	0.3	c.3.1.4
1qlb_6	53	0.976	7	1.00E+00	2.93E+05	0.970	9	1.00E+00	4.28E+01	7	9	0.3	c.3.1.4
1e7p_1	103	1.507	7	1.00E+00	1.08E+11	1.507	7	1.00E+00	1.08E+11	7	8	0.8	c.3.1.4
1e7p_2	55	1.606	7	1.00E+00	6.28E+10	1.606	7	1.00E+00	6.28E+10	7	8	0.3	c.3.1.4
1e7p_5	103	1.507	7	1.00E+00	1.08E+11	1.507	7	1.00E+00	1.08E+11	7	8	0.7	c.3.1.4
1e7p_6	55	1.606	7	1.00E+00	6.28E+10	1.606	7	1.00E+00	6.28E+10	7	8	0.3	c.3.1.4
1e7p_8	103	7.553	8	1.00E+00	3.66E+31	7.553	8	1.00E+00	3.66E+31	8	12	2.2	c.3.1.4
1e7p_9	55	1.606	7	1.00E+00	6.28E+10	1.606	7	1.00E+00	6.28E+10	7	8	0.3	c.3.1.4
1e7p_12	103	7.607	8	1.00E+00	3.91E+30	7.607	8	1.00E+00	3.91E+30	8	11	1.6	c.3.1.4
1e7p_13	55	1.501	7	1.00E+00	3.22E+09	1.501	7	1.00E+00	3.22E+09	7	7	0.3	c.3.1.4
1q9i_1	59	1.511	8	1.00E+00	3.87E+08	1.511	8	1.00E+00	3.87E+08	8	11	1.5	c.3.1.4
1kss_1	58	0.817	7	1.00E+00	9.89E+03	0.817	7	1.00E+00	9.89E+03	7	8	0.8	c.3.1.4
1m64_1	57	1.310	7	1.00E+00	2.75E+08	1.310	7	1.00E+00	2.75E+08	7	10	1.8	c.3.1.4
1m64_3	56	1.084	7	1.00E+00	3.76E+06	1.125	8	1.00E+00	1.75E+05	7	9	0.6	c.3.1.4
1e39_1	113	1.518	8	1.00E+00	3.13E+09	1.518	8	1.00E+00	3.13E+09	8	11	1.7	c.3.1.4
1e39_2	58	1.518	8	1.00E+00	4.13E+08	1.518	8	1.00E+00	4.13E+08	8	11	1.4	c.3.1.4
1ksu_1	59	1.546	8	1.00E+00	6.86E+08	1.546	8	1.00E+00	6.86E+08	8	11	1.7	c.3.1.4

Table 9: Results for alcohol dehydrogenase matching against FAD/NAD(P)-binding domain with amino acid property.

Site	N	RMSD	q	Pvalue	Evalue	RMSD	q	Pvalue	Evalue	CG	Mean L	Var L	SCOP
1ksu_3	59	1.558	8	1.00E+00	8.36E+08	1.558	8	1.00E+00	8.36E+08	8	11	1.9	c.3.1.4
1jry_2	59	1.520	8	1.00E+00	4.50E+08	1.520	8	1.00E+00	4.50E+08	8	11	1.7	c.3.1.4
1jry_3	61	1.318	7	1.00E+00	2.22E+08	1.318	7	1.00E+00	2.22E+08	7	10	2.3	c.3.1.4
1lj1_1	58	1.568	8	1.00E+00	9.45E+08	1.568	8	1.00E+00	9.45E+08	8	11	1.7	c.3.1.4
1lj1_3	57	1.551	8	1.00E+00	6.78E+08	1.551	8	1.00E+00	6.78E+08	8	11	1.6	c.3.1.4
1jrz_2	59	1.532	8	1.00E+00	5.50E+08	1.532	8	1.00E+00	5.50E+08	8	11	1.8	c.3.1.4
1jrz_3	58	1.536	8	1.00E+00	5.58E+08	0.531	8	9.03E-04	9.03E-04	6	13	5.3	c.3.1.4
1jrx_2	59	2.030	7	1.00E+00	3.99E+13	2.030	7	1.00E+00	3.99E+13	7	10	1.8	c.3.1.4
1jrx_3	60	1.772	7	1.00E+00	3.72E+12	1.768	8	1.00E+00	2.64E+11	7	9	1.3	c.3.1.4
1p2e_2	60	2.466	7	1.00E+00	8.45E+16	2.466	7	1.00E+00	8.45E+16	7	11	1.5	c.3.1.4
1p2h_1	59	1.525	8	1.00E+00	4.89E+08	1.525	8	1.00E+00	4.89E+08	8	11	1.8	c.3.1.4
1qjd_1	112	1.514	8	1.00E+00	2.85E+09	1.514	8	1.00E+00	2.85E+09	8	11	1.6	c.3.1.4
1qjd_2	58	1.514	8	1.00E+00	3.86E+08	1.514	8	1.00E+00	3.86E+08	8	11	1.7	c.3.1.4
1qo8_0	120	4.130	6	1.00E+00	1.08E+23	4.130	6	1.00E+00	1.08E+23	6	8	1.0	c.3.1.4
1qo8_2	120	3.843	7	1.00E+00	4.37E+22	3.763	8	1.00E+00	1.73E+22	7	9	1.1	c.3.1.4
1d4d_1	57	1.479	8	1.00E+00	2.69E+08	0.487	9	4.06E-08	4.06E-08	6	15	3.6	c.3.1.4
1d4e_1	58	1.377	9	1.00E+00	2.87E+06	1.342	10	1.00E+00	4.20E+04	9	12	0.9	c.3.1.4
1d4c_0	52	1.505	8	1.00E+00	3.28E+08	1.462	9	1.00E+00	6.40E+06	8	11	1.3	c.3.1.4
1d4c_1	55	1.510	8	1.00E+00	4.27E+08	1.463	9	1.00E+00	7.75E+06	8	11	1.2	c.3.1.4
1d4c_2	51	1.104	8	1.00E+00	1.04E+05	0.681	10	3.68E-06	3.68E-06	7	12	3.6	c.3.1.4
1d4c_9	53	0.947	7	1.00E+00	8.64E+04	0.914	8	1.00E+00	4.24E+02	7	10	0.9	c.3.1.4
1jnr_0	50	1.929	7	1.00E+00	6.11E+12	2.020	9	1.00E+00	2.61E+11	7	9	0.7	c.3.1.4
1jnr_1	50	1.922	7	1.00E+00	5.55E+12	2.010	9	1.00E+00	2.22E+11	7	9	0.8	c.3.1.4
1jnz_0	52	5.081	7	1.00E+00	1.93E+24	5.081	7	1.00E+00	1.93E+24	7	11	1.3	c.3.1.4
1jnz_1	53	5.139	7	1.00E+00	2.77E+24	5.139	7	1.00E+00	2.77E+24	7	11	1.5	c.3.1.4
3grs_0	45	1.032	6	1.00E+00	2.32E+07	0.998	7	1.00E+00	1.70E+05	6	9	1.4	c.3.1.5
1dnc_0	47	0.996	6	1.00E+00	1.32E+07	0.966	7	1.00E+00	9.27E+04	6	8	1.4	c.3.1.5
1gsn_0	47	3.861	6	1.00E+00	3.24E+17	3.861	6	1.00E+00	3.24E+17	6	11	1.8	c.3.1.5
1xan_0	45	3.978	6	1.00E+00	7.83E+19	3.978	6	1.00E+00	7.83E+19	6	10	1.8	c.3.1.5
1gre_1	45	1.027	6	1.00E+00	2.11E+07	0.996	7	1.00E+00	1.62E+05	6	8	1.2	c.3.1.5
1gra_1	45	1.057	6	1.00E+00	3.71E+07	1.021	7	1.00E+00	2.84E+05	6	9	1.3	c.3.1.5
1gra_2	12	3.719	5	1.00E+00	8.62E+16	3.719	5	1.00E+00	8.62E+16	5	6	0.4	c.3.1.5
1grg_0	45	1.016	6	1.00E+00	1.70E+07	0.986	7	1.00E+00	1.29E+05	6	8	1.3	c.3.1.5
1grf_0	45	1.003	6	1.00E+00	1.32E+07	0.972	7	1.00E+00	9.34E+04	6	8	1.4	c.3.1.5
1bwc_1	45	1.011	6	1.00E+00	1.55E+07	0.979	7	1.00E+00	1.10E+05	6	9	1.4	c.3.1.5
4gr1_0	45	1.037	6	1.00E+00	2.55E+07	1.003	7	1.00E+00	1.90E+05	6	8	1.3	c.3.1.5
1grb_0	60	0.982	7	1.00E+00	4.98E+05	0.982	7	1.00E+00	4.98E+05	7	8	0.6	c.3.1.5
1k4q_2	46	1.030	6	1.00E+00	2.39E+07	0.996	7	1.00E+00	1.73E+05	6	9	1.3	c.3.1.5
1grt_0	45	3.972	6	1.00E+00	7.57E+19	3.972	6	1.00E+00	7.57E+19	6	10	1.6	c.3.1.5
5grt_0	46	4.056	6	1.00E+00	1.27E+18	4.056	6	1.00E+00	1.27E+18	6	10	1.6	c.3.1.5
3grt_0	45	3.218	6	1.00E+00	7.86E+17	3.218	6	1.00E+00	7.86E+17	6	11	1.7	c.3.1.5
2grt_0	46	1.433	6	1.00E+00	1.98E+10	1.522	7	1.00E+00	3.56E+09	6	9	1.7	c.3.1.5
4grt_0	44	1.610	6	1.00E+00	6.58E+11	1.610	6	1.00E+00	6.58E+11	6	8	2.0	c.3.1.5
1onf_0	46	1.191	6	1.00E+00	4.13E+08	1.191	6	1.00E+00	4.13E+08	6	10	1.8	c.3.1.5
1ges_0	47	5.177	6	1.00E+00	8.11E+22	5.177	6	1.00E+00	8.11E+22	6	11	1.7	c.3.1.5
1ges_1	47	5.370	6	1.00E+00	5.45E+24	5.370	6	1.00E+00	5.45E+24	6	10	2.3	c.3.1.5
1ger_0	47	4.848	6	1.00E+00	7.88E+20	4.848	6	1.00E+00	7.88E+20	6	10	1.7	c.3.1.5
1ger_1	47	4.846	6	1.00E+00	7.81E+20	4.846	6	1.00E+00	7.81E+20	6	10	1.8	c.3.1.5
1get_0	65	10.626	7	1.00E+00	9.31E+28	10.626	7	1.00E+00	9.31E+28	7	10	2.1	c.3.1.5
1get_1	65	2.057	6	1.00E+00	4.37E+14	2.057	6	1.00E+00	4.37E+14	6	8	1.0	c.3.1.5
1geu_0	68	9.493	7	1.00E+00	4.82E+27	9.493	7	1.00E+00	4.82E+27	7	11	2.2	c.3.1.5
1geu_1	67	0.983	8	1.00E+00	5.61E+03	0.983	8	1.00E+00	5.61E+03	8	10	1.5	c.3.1.5
1fec_0	50	5.041	6	1.00E+00	6.74E+20	5.041	6	1.00E+00	6.74E+20	6	11	1.9	c.3.1.5
1fec_1	50	7.723	7	1.00E+00	8.40E+23	7.723	7	1.00E+00	8.40E+23	7	10	1.6	c.3.1.5
1feb_0	50	4.322	6	1.00E+00	2.20E+22	4.322	6	1.00E+00	2.20E+22	6	11	1.7	c.3.1.5
1feb_1	48	0.851	7	1.00E+00	3.27E+03	0.867	10	2.03E-03	2.03E-03	7	10	0.6	c.3.1.5
1fea_0	49	5.467	6	1.00E+00	5.47E+21	5.467	6	1.00E+00	5.47E+21	6	10	1.9	c.3.1.5
1fea_1	50	1.122	7	1.00E+00	7.60E+06	1.221	8	1.00E+00	1.56E+06	7	9	0.8	c.3.1.5
1fea_2	49	5.467	6	1.00E+00	5.47E+21	5.467	6	1.00E+00	5.47E+21	6	10	1.8	c.3.1.5
1fea_3	50	2.191	7	1.00E+00	2.83E+15	2.191	7	1.00E+00	2.83E+15	7	8	0.8	c.3.1.5
2tpr_0	49	0.911	7	1.00E+00	2.14E+04	0.910	10	1.31E-02	1.32E-02	7	10	0.7	c.3.1.5
2tpr_1	50	5.478	6	1.00E+00	6.05E+21	5.478	6	1.00E+00	6.05E+21	6	10	1.7	c.3.1.5
1tyt_0	49	2.365	7	1.00E+00	3.98E+15	2.380	8	1.00E+00	8.52E+14	7	10	1.2	c.3.1.5
1tyt_1	50	4.259	6	1.00E+00	1.55E+22	4.259	6	1.00E+00	1.55E+22	6	11	1.7	c.3.1.5
1typ_1	65	0.938	7	1.00E+00	3.59E+05	0.877	9	9.51E-01	3.02E+00	7	10	1.1	c.3.1.5
1typ_3	48	0.932	7	1.00E+00	6.95E+04	0.856	9	2.68E-01	3.12E-01	7	10	0.6	c.3.1.5
1typ_4	21	1.535	5	1.00E+00	1.89E+11	1.535	5	1.00E+00	1.89E+11	5	7	0.8	c.3.1.5
1aog_0	49	0.854	7	1.00E+00	3.82E+03	0.849	10	9.86E-04	9.87E-04	7	10	0.5	c.3.1.5
1aog_3	49	0.854	8	1.00E+00	3.43E+01	0.809	10	1.62E-04	1.62E-04	8	10	0.6	c.3.1.5

Table 10: Results for alcohol dehydrogenase matching against FAD/NAD(P)-binding domain with amino acid property.

Site	N	RMSD	q	Pvalue	Evalue	RMSD	q	Pvalue	Evalue	CG	Mean L	Var L	SCOP
1bzl_0	49	1.573	6	1.00E+00	1.04E+11	1.601	7	1.00E+00	8.62E+09	6	10	1.6	c.3.1.5
1bzl_1	49	1.582	6	1.00E+00	1.18E+11	1.619	7	1.00E+00	1.13E+10	6	10	1.8	c.3.1.5
1gxf_0	120	5.637	7	1.00E+00	6.81E+26	5.637	7	1.00E+00	6.81E+26	7	11	2.8	c.3.1.5
1gxf_2	120	2.265	7	1.00E+00	3.48E+16	2.265	7	1.00E+00	3.48E+16	7	10	2.0	c.3.1.5
1nda_0	49	4.559	6	1.00E+00	1.28E+23	4.559	6	1.00E+00	1.28E+23	6	10	1.6	c.3.1.5
1nda_1	49	4.559	6	1.00E+00	1.28E+23	4.559	6	1.00E+00	1.28E+23	6	10	1.5	c.3.1.5
1h6v_0	120	1.426	7	1.00E+00	1.07E+11	1.523	8	1.00E+00	3.45E+10	7	9	0.9	c.3.1.5
1h6v_1	120	1.706	8	1.00E+00	2.15E+12	1.706	8	1.00E+00	2.15E+12	8	10	1.2	c.3.1.5
1h6v_2	120	1.216	7	1.00E+00	7.90E+08	1.216	7	1.00E+00	7.90E+08	7	7	0.1	c.3.1.5
1h6v_3	120	5.250	7	1.00E+00	1.72E+27	5.250	7	1.00E+00	1.72E+27	7	13	2.4	c.3.1.5
1h6v_4	120	1.493	7	1.00E+00	4.00E+11	1.563	8	1.00E+00	7.82E+10	7	9	0.8	c.3.1.5
1h6v_5	120	1.438	7	1.00E+00	8.58E+10	1.535	9	1.00E+00	1.07E+09	7	9	0.6	c.3.1.5
1trb_0	49	1.794	7	1.00E+00	1.45E+12	1.794	7	1.00E+00	1.45E+12	7	8	0.7	c.3.1.5
1cl0_0	47	1.580	7	1.00E+00	4.39E+10	1.618	8	1.00E+00	4.83E+09	7	8	0.4	c.3.1.5
1tde_0	52	1.631	7	1.00E+00	8.60E+10	1.631	7	1.00E+00	8.60E+10	7	8	0.4	c.3.1.5
1tdf_0	52	1.620	7	1.00E+00	7.28E+10	1.620	7	1.00E+00	7.28E+10	7	8	0.5	c.3.1.5
1tdf_1	26	3.430	6	1.00E+00	7.20E+18	3.430	6	1.00E+00	7.20E+18	6	9	0.8	c.3.1.5
1f6m_0	62	1.598	7	1.00E+00	8.89E+10	1.598	7	1.00E+00	8.89E+10	7	7	0.3	c.3.1.5
1f6m_1	63	1.614	7	1.00E+00	1.19E+11	1.614	7	1.00E+00	1.19E+11	7	7	0.5	c.3.1.5
1f6m_2	62	1.602	7	1.00E+00	9.46E+10	1.602	7	1.00E+00	9.46E+10	7	7	0.3	c.3.1.5
1f6m_3	63	1.615	7	1.00E+00	1.21E+11	1.615	7	1.00E+00	1.21E+11	7	7	0.3	c.3.1.5
1vdc_0	95	1.576	7	1.00E+00	6.12E+11	1.576	7	1.00E+00	6.12E+11	7	8	1.2	c.3.1.5
1m6i_0	43	2.027	6	1.00E+00	5.20E+13	2.010	7	1.00E+00	4.78E+12	6	8	1.0	c.3.1.5
1gv4_0	109	1.415	7	1.00E+00	1.01E+10	1.415	7	1.00E+00	1.01E+10	7	8	1.1	c.3.1.5
1hyu_0	44	2.518	6	1.00E+00	1.07E+16	2.518	6	1.00E+00	1.07E+16	6	9	1.0	c.3.1.5
1fl2_5	43	2.616	6	1.00E+00	1.05E+15	2.540	7	1.00E+00	1.28E+14	6	9	1.2	c.3.1.5
1nhq_0	37	3.028	6	1.00E+00	1.96E+17	3.028	6	1.00E+00	1.96E+17	6	8	1.1	c.3.1.5
1nhp_0	37	1.357	6	1.00E+00	1.81E+10	1.387	7	1.00E+00	1.13E+09	6	8	0.9	c.3.1.5
1nhr_0	37	2.888	6	1.00E+00	6.24E+17	2.888	6	1.00E+00	6.24E+17	6	9	1.1	c.3.1.5
1nhs_0	39	1.366	6	1.00E+00	2.50E+10	1.391	7	1.00E+00	1.44E+09	6	8	1.1	c.3.1.5
1npx_0	40	3.026	6	1.00E+00	2.46E+17	3.026	6	1.00E+00	2.46E+17	6	8	1.2	c.3.1.5
1f8w_0	39	5.380	6	1.00E+00	6.10E+22	5.380	6	1.00E+00	6.10E+22	6	9	1.0	c.3.1.5
1joa_0	57	6.459	6	1.00E+00	1.02E+25	6.459	6	1.00E+00	1.02E+25	6	9	1.7	c.3.1.5
2npx_0	59	0.931	7	1.00E+00	2.22E+05	0.551	9	6.22E-07	6.22E-07	6	13	5.0	c.3.1.5
1d7y_0	42	2.095	7	1.00E+00	4.60E+12	2.068	8	1.00E+00	4.11E+11	7	9	0.7	c.3.1.5
1f3p_0	63	1.146	8	1.00E+00	5.57E+05	1.146	8	1.00E+00	5.57E+05	8	11	1.4	c.3.1.5
1q1r_0	41	1.639	7	1.00E+00	2.89E+10	1.639	7	1.00E+00	2.89E+10	7	9	1.1	c.3.1.5
1q1r_1	42	1.866	7	1.00E+00	4.56E+12	1.914	9	1.00E+00	8.17E+10	7	9	0.6	c.3.1.5
1q1w_0	41	2.285	6	1.00E+00	1.04E+15	2.285	6	1.00E+00	1.04E+15	6	8	1.2	c.3.1.5
1q1w_1	42	1.694	7	1.00E+00	6.56E+10	1.694	7	1.00E+00	6.56E+10	7	9	1.2	c.3.1.5
1lvl_0	66	0.919	9	1.00E+00	1.45E+01	0.609	9	2.26E-05	2.26E-05	8	11	2.6	c.3.1.5
1lpf_0	47	5.936	7	1.00E+00	3.28E+27	5.936	7	1.00E+00	3.28E+27	7	11	2.1	c.3.1.5
1lpf_1	47	4.051	7	1.00E+00	3.40E+20	4.051	7	1.00E+00	3.40E+20	7	8	0.8	c.3.1.5
3lad_0	48	1.720	6	1.00E+00	2.07E+12	1.720	6	1.00E+00	2.07E+12	6	9	2.2	c.3.1.5
3lad_1	46	2.293	6	1.00E+00	5.15E+15	2.311	8	1.00E+00	1.70E+14	6	10	1.2	c.3.1.5
1ebd_0	46	1.723	8	1.00E+00	9.37E+09	1.723	8	1.00E+00	9.37E+09	8	8	0.4	c.3.1.5
1ebd_1	49	2.544	7	1.00E+00	2.81E+16	2.544	7	1.00E+00	2.81E+16	7	11	2.1	c.3.1.5
1ojt_0	48	1.333	7	1.00E+00	1.68E+08	1.333	7	1.00E+00	1.68E+08	7	9	1.2	c.3.1.5
1bhy_0	47	1.335	7	1.00E+00	1.64E+08	1.335	7	1.00E+00	1.64E+08	7	8	1.0	c.3.1.5
1jeh_0	48	1.702	6	1.00E+00	1.63E+12	1.693	7	1.00E+00	9.66E+10	6	11	2.0	c.3.1.5
1jeh_1	49	1.616	6	1.00E+00	5.62E+11	1.878	10	1.00E+00	9.74E+08	6	11	0.9	c.3.1.5
1dxl_0	90	2.136	7	1.00E+00	1.28E+14	2.182	8	1.00E+00	2.89E+13	7	10	1.4	c.3.1.5
1dxl_1	86	1.720	7	1.00E+00	1.49E+12	1.805	8	1.00E+00	3.93E+11	7	11	2.3	c.3.1.5
1mo9_0	44	1.896	7	1.00E+00	3.67E+12	1.873	8	1.00E+00	2.43E+11	7	9	0.4	c.3.1.5
1mo9_1	46	6.243	6	1.00E+00	1.05E+26	6.243	6	1.00E+00	1.05E+26	6	11	1.8	c.3.1.5
1mok_0	47	1.889	7	1.00E+00	8.92E+12	1.827	8	1.00E+00	3.06E+11	7	10	0.9	c.3.1.5
1mok_1	46	1.935	7	1.00E+00	1.59E+13	1.872	8	1.00E+00	5.87E+11	7	10	1.0	c.3.1.5
1mok_2	46	1.946	7	1.00E+00	1.84E+13	1.882	8	1.00E+00	6.88E+11	7	10	1.0	c.3.1.5
1mok_3	45	1.923	7	1.00E+00	1.26E+13	1.867	8	1.00E+00	5.07E+11	7	10	1.2	c.3.1.5
1fcd_2	48	0.968	7	1.00E+00	1.79E+05	0.701	9	4.73E-04	4.74E-04	6	12	5.9	c.3.1.5
1fcd_3	46	1.594	6	1.00E+00	1.13E+11	1.627	8	1.00E+00	6.19E+08	6	9	0.9	c.3.1.5
1w4x_0	120	1.596	8	1.00E+00	6.27E+10	1.724	9	1.00E+00	3.69E+10	8	9	0.6	c.3.1.5
